# The Values and Preferences of People Living With Motor Neurone Disease (MND): A Systematic Review and Meta‐Analysis

**DOI:** 10.1002/gin2.70080

**Published:** 2026-07-15

**Authors:** Timothy Hugh Barker, Lemma N. Bulto, Grace Holland, Camille Schubert, Tracy Merlin, Samer G. Karam, Wojtek Wiercioch, Danielle Pollock, Cindy Stern, Jay Beasley‐Hall, Nipun Shrestha, Steve Vucic, Lynne Giles, Zachary Munn

**Affiliations:** ^1^ Health Evidence Synthesis, Recommendations and Impact (HESRI) School of Public Health, College of Health, Adelaide University Adelaide South Australia Australia; ^2^ Adelaide Health Technology Assessment (AHTA) School of Public Health, College of Health, Adelaide University Adelaide South Australia Australia; ^3^ Michael G. DeGroote Cochrane Canada & McMaster GRADE Centres McMaster University Hamilton Ontario Canada; ^4^ Department of Health Research Methods, Evidence, and Impact McMaster University Hamilton Ontario Canada; ^5^ University Library, Adelaide University Adelaide South Australia Australia; ^6^ Brain and Nerve Research Centre Concord Clinical School, The University of Sydney Sydney New South Wales Australia; ^7^ School of Public Health, College of Health, Adelaide University Adelaide South Australia Australia

**Keywords:** motor neurone disease, preferences, valuation of outcomes, values

## Abstract

**Aim:**

To synthesise quantitative evidence on the values and preferences of people living with Motor Neurone Disease (MND), caregivers, and genetic carriers regarding health‐related outcomes to inform the Australian MND Guideline.

**Methods:**

A systematic review was conducted following Cochrane and GRADE guidance, informed by an *a priori* protocol. Major electronic databases (including MEDLINE, Embase, CENTRAL) and trial registries were searched to identify studies that met the eligibility criteria. Risk of bias of the studies that met the eligibility criteria was assessed using the Risk of Bias in Studies of Values and Utilities (ROBVALU) tool. Data on health state utility values were synthesised using meta‐analysis where appropriate, while other quantitative data deemed inappropriate for meta‐analysis were synthesised narratively. The certainty of the evidence for each outcome was assessed using the GRADE approach.

**Results:**

Twenty‐four studies (*n* = 10,397) were included. Overall health‐related quality of life (hrQoL) utility values for adults with MND varied significantly based on the regional preference set utilised (mean EQ‐5D utility ranging from 0.57 in UK cohort (high certainty in the evidence) to 0.72 in Chinese cohorts (low certainty in the evidence). Utility values declined consistently with increasing disease severity across multiple staging systems, such as King's and MiToS. Narrative synthesis identified clear preferences across physical, psychosocial, and healthcare domains regarding both current and hypothetical treatment strategies.

**Conclusion:**

This review provides a comprehensive synthesis of the values and preferences of the broad MND community. This review has been conducted following rigorous best‐practice methodology, to directly inform the selection and prioritisation of outcomes for the development of the Australian MND Guideline, ensuring the guideline adheres to a patient‐centred approach. Standardisation of preference elicitation methods from the MND community, and the development of a core outcome set for future MND research are key future priorities.

## Summary

- **What We Already Know**
  ∘ People living with MND face deeply personal trade‐offs between prolonging survival, preserving comfort, and maintaining autonomy, and their values and preferences are central to patient‐centred, trustworthy guideline development.
  ∘ No systematic review had previously synthesised the quantitative evidence on the outcomes that matter most to people living with MND and their caregivers.
- **What This Adds to What Was Known?**
  ∘ This is the first comprehensive systematic review and meta‐analysis of the values and preferences of people living with MND, synthesising 24 studies (*n* = 10,397) using GRADE methods and the ROBVALU risk‐of‐bias tool.
  ∘ Health‐related quality‐of‐life utilities declined consistently with disease progression across multiple staging systems.
  ∘ Narrative synthesis across 14 domains mapped preferences spanning physical, psychosocial, and healthcare outcomes, though the certainty of evidence was frequently low or very low.
- **What Is the Implication and What Should Change Now?**
  ∘ These findings directly inform the selection and prioritisation of outcomes for the Australian MND Guideline, supporting a patient‐centred approach, and can help guide clinical decision‐making and future research.
  ∘ Standardising preference‐elicitation methods and developing a core outcome set for MND research are key priorities to improve the certainty and comparability of future evidence.

## 1 Background

Motor Neurone Disease (MND) is a progressive neurodegenerative disorder affecting upper and lower motor neurones, leading to paralysis and respiratory failure [1, 2, 3, 4]. Types include amyotrophic lateral sclerosis (ALS); progressive muscular atrophy (PMA); primary lateral sclerosis (PLS); progressive bulbar palsy (PBP); and hereditary spastic paraparesis (HSP) [5, 6]. In Australia, MND affects approximately 2000 people at any given time, with two deaths occurring daily [4, 7, 8]. The disease severely impacts physical functions and the emotional well‐being of both patients and their families [9, 10, 11].

Individuals living with MND (and their families and caregivers) face complex decisions care decisions, which need to be balanced across the connected constructs of independent health, social well‐being, and emotional safety. There is well documented diversity among individuals with lived experienced of MND regarding their priorities and preferences with respect to their health outcomes, with some patients opting for strategies that prolong survival, while others may opt for comfort or maintenance of their autonomy [12, 13]. The preference for a specific provision of care that a patient may opt to engage with is therefore an implicit result of the importance of health outcomes that an individual connects with that specific provision of care [13]. The GRADE (Grading of Recommendations Assessment, Development and Evaluation) Working Group operationalise patient values and preferences as “the relative importance patients (and other populations of interest) place on outcomes” [14, 15].

Understanding these preferences is vital for patient‐centred care and the development of trustworthy guidelines [16]. Evidence towards the values and preferences of those with a lived experience of a condition directly informs the importance of outcomes, the trade‐off between benefits and harms, the direction of any recommendations made, dissemination strategies, and any decision aids and implementation tools associated with the guidelines [13].

As a preliminary search identified no existing systematic reviews on this topic, this review aims to synthesise evidence regarding the health‐related outcome preferences of people with lived experienced of MND.

### 1.1 Review Question

What are the values and preferences of the Australian MND Community regarding their health‐related outcomes associated with any intervention for MND care?

## 2 Methods

This review is part of the overall project coordinated by Health Evidence Synthesis, Recommendations, and Impact (HESRI) and the Adelaide GRADE Centre to develop the Australian MND Guideline. This review has been conducted following guidance from Cochrane [17] and the GRADE Working Group [13, 14, 15] and utilises adapted methodology from previous systematic reviews investigating the values and preferences of the target population [18, 19, 20, 21, 22, 23]. This review has been reported in line with PRISMA (Preferred Reporting Items for Systematic Reviews and Meta‐Analyses) 2020 [24] (Supporting Information S1: Material 1) This review has been registered within PROSPERO, ID: CRD420250653287 and a protocol has been published [25].

### 2.1 Eligibility Criteria

#### 2.1.1 Participants

Studies were eligible for this review where they have reported data from any of the following sub‐populations of people:

1. Adults (18+) diagnosed with any type of MND, such as amyotrophic lateral sclerosis (ALS), progressive bulbar palsy (PBP), progressive muscular atrophy (PMA), primary lateral sclerosis (PLS), hereditary spastic paraparesis (HSP), MND with frontal temporal dementia (MND/FTD), Hirayama disease/monomelic amyotrophy (MMA) and adult onset (type VI) spinal muscular atrophy (SMA). Regarding SMA, studies were only eligible for inclusion where the data is specific to adult‐onset SMA (e.g., type IV).
2. Individuals with lived experience of MND, including caregivers and family members directly involved in care decisions. There were no restrictions placed on the mode of caregiving (e.g., live‐in caregiver, full time caregiver, paid vs. unpaid etc.).
3. Clinicians involved in the direct provision of treatment and care to people diagnosed with MND, or its subtypes as listed above (e.g., palliative care physicians to manage quality of life and end of life care or pulmonologists involved in the management of breathing difficulties and non‐invasive ventilation).
4. Asymptomatic carriers (also known as genetic carriers) of MND. Defined as individuals that carry a mutation in a gene (C9orf72, SOD1, FUS, and TARDBP genes) associated with MND, but do not exhibit symptoms of the disease themselves.

Studies were ineligible for this review where they focused exclusively on other neurological conditions unrelated to MND, however should studies investigated both MND and other unrelated neurological conditions, attempts were made to extract only the data related to MND. Studies were also ineligible for this review where they involved only populations without direct lived experience of MND (e.g., general public, or healthcare professionals without firsthand interaction).

For the sub‐population of clinicians, studies were only eligible where they had investigated outcome valuations by clinicians as a proxy to the outcome values of a person diagnosed with MND. Studies that investigated outcome valuations by clinicians, in relation to clinician‐specific outcomes (e.g., burnout) were excluded.

#### 2.1.2 Phenomena of Interest

Studies were eligible for this review if they explored the values and/or preferences of the eligible population regarding their satisfaction or health and quality of life related outcomes. These needed to include specific valuations of the eligible population regarding their satisfaction or health and quality of life related outcomes. This included (but is not limited to) preferences related to symptom management, end‐of‐life care decisions, treatment trade‐offs, or other health or healthcare related experiences of living with MND.

#### 2.1.3 Context

There were no limitations regarding study eligibility for this review based on context. Studies were eligible for this review that have been conducted in any country and in any healthcare setting (e.g., hospitals, outpatient clinics, home care etc.).

There were no exclusions based on language and no date limitations. The DeepL Translator (DeepL, Cologne, Germany) was used to translate any titles and abstracts in languages authors were not fluent in. We reached out to experts from the Australian MND guideline for suggestions of references to include where appropriate, however none were identified.

#### 2.1.4 Outcomes

An exhaustive list of outcomes could not be pre‐determined due to the expected diversity of the available data. However, studies were eligible for this review where they reported data on health outcomes such as (but not limited to) quality of life, satisfaction, survival, autonomy and so forth.

Studies were ineligible for this review when the reported outcomes focused exclusively on the objective measurement of clinical indicators or biomarkers (e.g., spirometry results, grip strength, or biomarker levels). To be eligible, studies must have explicitly measured the relative importance, utility, or preference assigned to those clinical outcomes (e.g., asking participants to rank the importance of preserving fine motor skills vs. speech, or deriving a utility valuation for specific functional states).

#### 2.1.5 Study Design

Studies were eligible for this review only if they were peer‐reviewed research papers that followed an established and appropriate methodology (described below). Eligible studies were any that have reported eligible populations' preferences and/or values regarding their MND related health outcomes associated with MND care or living with MND. Quantitative studies with the following characteristics were eligible where they reported the relative importance of the health‐related outcomes [13]:

1. Patient reported measures (PRMs) and quality of life studies
  Studies that quantitatively measure the lived experience of the population(s) of interest, in one or multiple domains that were directly relevant to patient satisfaction or quality of life; typically referred to as patient‐reported experience measures (PREMs) or patient‐reported outcome measures (PROMs). Quality of life measuring tools were disease/topic specific questionnaires (such as the ALSAQ‐40 for MND [26]), or generic multi‐attribute utility instruments (MAUIs) such as the EQ‐5D [27], Short Form‐36 Health Survey (SF‐36) [28], Health Utilities Index (HUI) [29] or Quality of Wellbeing (QWB) [30]. Where quality of life data were captured using a validated tool and subsequently translated to a utility estimate (described below) using an external mapping or preference algorithm, both the raw and translated measures were relevant.
2. Direct utility and health state value studies
  Studies that directly measured how the population of interest perceive the value or quality of different health states by use of assigned numerical values (utility values) that reflect the desirability of living in those states [31]. Utility values are typically reported on a scale where 1 represents perfect health, and 0 represents a state equivalent to death [31]. In some instances, negative values can be used to indicate a state that is considered worse than death. Study methodologies that elicit direct measurement or valuation include survey techniques such as the Standard Gamble (SG), Time Trade‐Off (TTO) or Visual Analogue Scale (VAS). Although representing the same concept and nominally the same, utility values estimated by different methods of direct elicitation are poorly comparable to one another and to the indirect estimation of utility captured through MAUIs.
3. Direct choice studies
  Studies that quantitatively examined the population of interest's choice, after presenting them with a description of hypothetical states or during decision‐making for their own actual health states [20]. Studies that utilised a willingness to pay approach or conduct a randomised controlled trial of preferences (whereby there was a randomised group [participants randomised to treatment groups as per normal] and a preferences group [participants with strong preference for a treatment were allocated to their preferred treatment]). They also utilised a direct/forced choice exercise where the population of interest is prompted to make a choice from a set of options or rank a set of alternatives to enable best‐worst scaling [32].
4. Other studies utilising quantitative methodology to assess outcome importance
  Studies that employed any other quantitative methodology (e.g., bespoke questionnaires or other non‐utility measurement techniques) whereby the patients values and preferences regarding their health‐related outcomes were examined.

### 2.2 Search Strategy

Comprehensive search strategies (Supporting Information S1: Material 2) were developed by a research librarian (JBH) and peer‐reviewed against using the Peer Review of Electronic Search Strategies Guideline Statement [33]. Following an initial PubMed pilot search, a full strategy was developed for MEDLINE and adapted for Embase, CENTRAL and clinical trial registries (ClinicalTrials.gov and WHO ICTRP). References lists and citations of included studies were screened using citationchaser [34] and/or a related citations search [35].

### 2.3 Study Selection and Screening

**Figure 1:**
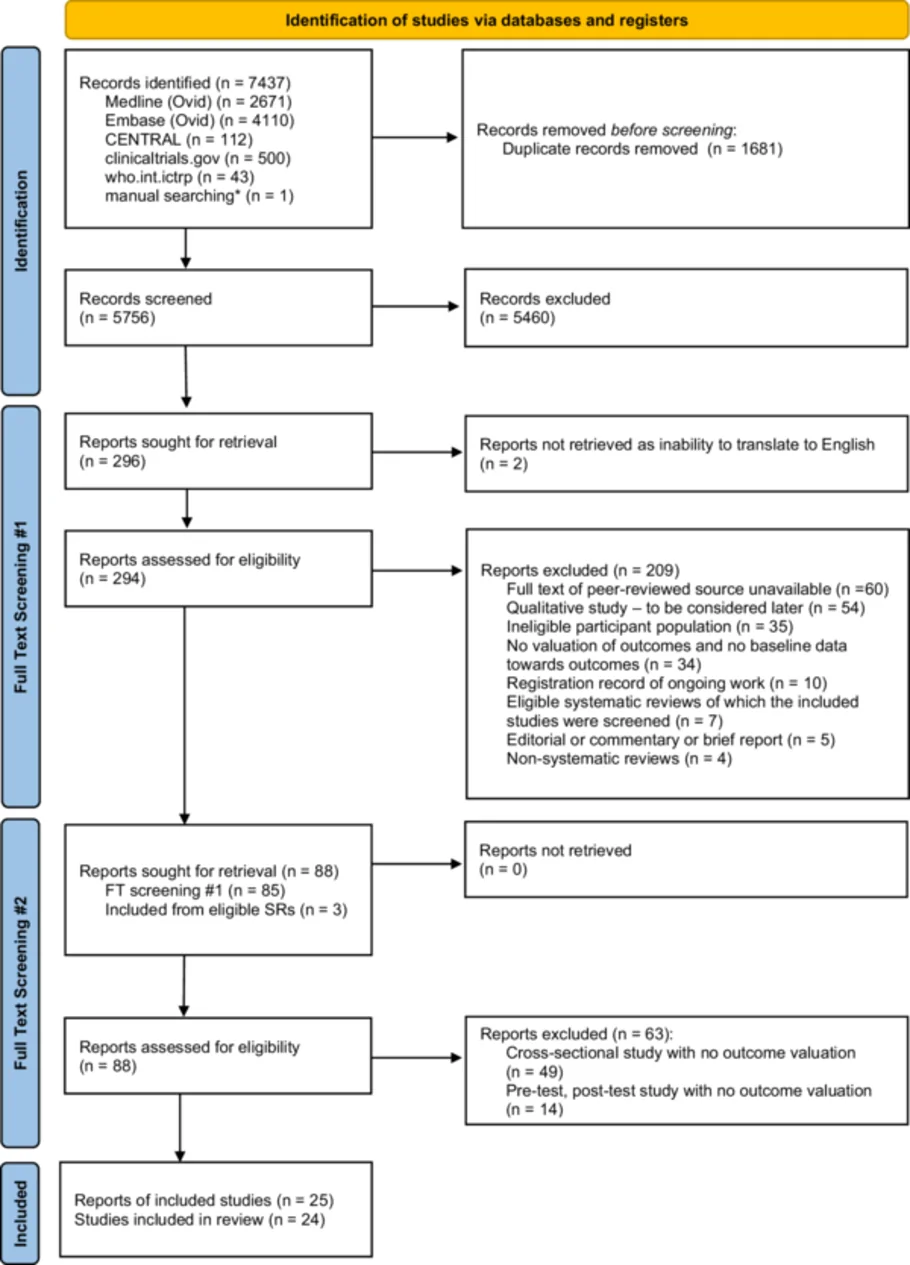
PRISMA Flow Diagram, depicting the movement of studies throughout the review. *1 record was identified through manual searching that was not returned from the search strategy. This work is licensed under CC BY 4.0. To view a copy of this license, visit https://creativecommons.org/licenses/by/4.0/ Source: Page MJ, et al. BMJ 2021;372:n71. doi: 10.1136/bmj.n71.

Records were managed in Covidence, with duplicates removed automatically. Two or more independent reviewers (T.H.B., Z.M., G.M.) screened titles and abstracts against eligibility criteria, following a pilot phase (5% of records) that established > 70% agreement between all reviewers. Full‐text reports were retrieved of eligible records and assessed independently by two reviewers, following the same pilot methods as above. (T.H.B., L.B., G.M.). Reasons for exclusion at this stage were recorded (Supporting Information S1: Material 3). Disagreements were resolved via discussion. Search results are detailed as a PRISMA 2020 flow diagram (Figure 1) [24]. More detailed methods of this study selection process are provided as Supporting Information S1: Material 5.

### 2.4 Assessment of Risk of Bias

Two review authors (T.H.B. and L.B.) independently assessed the risk of bias for each study using the Risk of Bias in Studies of Values and Utilities (ROBVALU) tool [36].

ROBVALU considers four subdomains: selection of participants, completeness of data, measurement instrument and data analysis. Across these subdomains, seven key signalling questions were presented, the answers to these being yes, probably yes, probably no and no. Studies were ascribed an overall risk of bias (no serious, serious, very serious, or extremely serious) based on the seven‐step ROBVALU framework, which evaluates participant selection, data completeness, measurement instruments, and analysis plans. Detailed assessment criteria are provided in Supporting Information S1: Material 5. The assessment of risk of bias was piloted on two studies, and all assessments were conducted by two independent reviewers (T.H.B. and L.B.). Any disagreements that arose between the reviewers were resolved through discussion. Where required, authors of papers were contacted to request missing or additional data. Results of risk of bias assessment are presented narratively in this report and are supported by figures and tables. All studies, regardless of their assessment of risk of bias were included in the review.

### 2.5 Data Extraction

Data from all included studies were extracted independently and in duplicate (T.H.B. and L.B.), using structured extraction forms relevant to the type of data. The instrument was adapted from a previous patient values and preferences systematic review [18]. The data extraction form was piloted on two studies included in the review following study selection (as such, the final extraction tool was modified and appears differently to the tool presented in the protocol) this is available as Supporting Information S1: Material 4. Data extraction was supported where appropriate by a dictionary of terms to ensure uniformity of extraction items between members of the review team. Disagreements and inconsistencies in extraction were resolved through discussion and consensus. Where required, data reported in figures but not in the text of included studies was digitised using software. This included the GetData Graph Digitizer [37] and ChatGPT 4.0 [38]. Each instance for which digitisation was required, has been reported in Supporting Information S1: Material 9.

### 2.6 Data Synthesis

Meta‐analyses were performed using the *metafor* package in R (version 4.2.1) [39] when studies reported identical health states and instruments to measure these states. We used DerSimonian‐Laird random‐effects models when four or more studies contributed to a meta‐analysis, and inverse variance fixed‐effect models when three of fewer studies contributed, due to unreliable variance estimation [23, 40]. Sensitivity was assessed by comparing random‐ and fixed‐effect results. Relative and absolute heterogeneity were evaluated using *I* ^2^, Cochrans *Q*, tau^2^and prediction intervals. A global Wald‐type test was used to assess the statistical significance of the pooled interactions, providing a single overall test for subgroup effect. When meta‐analysis was precluded by instrument validity, utility ranges or narrative descriptions of central tendency and variability were reported [18]. Non‐utility data were narratively synthesised by population subgroup. Missing data were sought from authors without imputation. Comprehensive details of these statistical methods are provided in Supporting Information S1: Material 5.

### 2.7 Patient and Public Involvement

This systematic review was conducted for the purposes of informing the Australian MND guideline. Guideline development panel members. Many diverse interest holders including clinicians, potential end‐users of the guideline, experts and other decision‐makers have shaped the review question and focus and guided the interpretation of the results.

### 2.8 Assessment of Certainty of the Evidence

The GRADE approach for grading the certainty of evidence in the importance of outcomes or values and/or preferences was followed [14, 15]. Each GRADE Summary of Findings table (presented above) detailed the following information where appropriate: study design, measurement instrument (or methodology where appropriate), number of participants, and estimate of outcome importance. Additionally, an overall certainty assessment was provided, which was informed through individual assessments of the domains of risk of bias, inconsistency, indirectness, imprecision, and publication bias of the overall body of evidence for each outcome. The overall certainty of evidence started as high and was downgraded according to the domains listed above. Where appropriate, the certainty of evidence was upgraded due to the presence of a dose‐response gradient.

### 2.9 Deviations From Protocol

Originally, we aimed to include qualitative studies, however due to the vast number of quantitative studies available which provided a substantial amount of diverse outcome valuation data, a pragmatic decision was made to limit our inclusion to quantitative studies for this review. Qualitative studies exploring values and preferences among MND patients were identified during this review process but will be presented separately in additional forthcoming work. Populations of people with Kennedy's disease/spinal and bulbar muscular atrophy (SBMA) were identified in the protocol to be included, however upon feedback received from multiple parties with extensive experience in MND management, it was determined that this population of people were not appropriate for inclusion into the review. Finally, exploration of the data through meta‐regression was planned in the protocol where common study characteristics existed between studies. However, due to the considerable heterogeneity observed, this was not possible.

## 3 Results

### 3.1 Results of the Search

The PRISMA flow diagram of the entire screening process is presented below (Figure 1). There were 7436 citation records identified in the initial database search (Medline, Embase and CENTRAL) and from trial registries (ClinicalTrials.gov and who.int.ictrp). One additional study was identified from manual searching of databases that appeared to meet eligibility criteria but was not identified from the search strategy (discussed below). Of these, 1681 were removed as duplicates. This left 5756 unique citation records to be screened at the title and abstract level.

All records were screened by title and abstract, and 5460 records were excluded for failing to meet the eligibility criteria and two records were irretrievable. This left 296 records in which the full‐text reports were sought and screened against the eligibility criteria. Of the 296 reports screened at full‐text, 209 were excluded; primary reasons included unavailability of peer‐reviewed text, qualitative design, or lack of direct outcome valuation. A full list of exclusions is provided in the PRISMA diagram (Figure 1) and Supporting Information S1: Material 3.

This left 85 reports of studies eligible for inclusion. Additionally, three studies were identified from screening the reference lists of the identified eligible systematic reviews, resulting in 88 studies eligible. From this list of 88 reports, a second screen was required, where all studies that did not provide a direct outcome valuation were excluded. Of these, 63 records were excluded for one of two reasons: cross‐sectional study with no outcome valuation (*n* = 49); or pre‐test, post‐test study with no outcome valuation (*n* = 14). Finally, two reports [41, 42] belonging to the same overall study were merged. This left 24 studies which met the eligibility criteria and have been included in the final review. The PRISMA flow diagram of the entire screening process is presented below (Figure 1).

### 3.2 Characteristics of the Included Studies

A total of 24 studies involving 10,397 participants were included in this review, comprising 6246 adults with MND, 399 caregivers, and 152 clinicians providing proxy outcome valuations. Studies were conducted across 14 countries, including Australia, China, the USA, Canada, and several European nations. The evidence base primarily consists of cross‐sectional designs [41, 43, 44, 45, 46, 47, 48, 49, 50, 51, 52, 53, 54] (*n* = 13), with additional data from retrospective trial analyses [55, 56, 57, 58] (*n* = 4), pre‐test post‐test studies [59, 60, 61] (*n* = 3), and specialised preference‐elicitation methods such as discrete choice experiments [62, 63] (*n* = 2) and standard gamble exercises [41, 55] (*n* = 2). Outcomes under investigation spanned physical, psychological, and healthcare domains, with a focus on health‐related quality of life measured via generic (e.g., EQ‐5D) and disease‐specific (e.g., ALSAQ‐40) instruments. Most studies (*n* = 10) were conducted since 2015 [43, 44, 45, 46, 47, 49, 53, 54, 60, 63]. Comprehensive study characteristics, including specific recruitment strategies and eligibility criteria, are detailed in Supporting Information S1: Materials 4 and 5.

### 3.3 Assessment of Risk of Bias

Risk of bias was evaluated across four domains, participant selection, data completeness, measurement instruments, and data analysis, using the ROBVALU tool. Overall, 12 included studies [41, 44, 45, 50, 53, 54, 55, 58, 62, 63, 64, 65] demonstrated no serious risk of bias, while 11 [43, 46, 47, 48, 49, 51, 52, 57, 59, 60, 61] were found to have extremely serious risk, and one was judged to have serious risk [56]. Bias was least frequent in the selection of participants and completeness of data domains. Methodological concerns were most prominent regarding measurement instruments and data analysis. Detailed risk of bias judgements for each study across all domains are summarized in Supporting Information S1: Material 4. The summarised risk of bias judgements using ROBVALU and the complete risk of bias assessments, with detailed responses to each signalling question are also provided as Supporting Information S1: Material 4. Finally, detailed narrative for each domain assessed is provided as Supporting Information S1: Material 6.

### 3.4 Outcome Data Synthesis and Meta‐Analysis

#### 3.4.1 Meta‐Analyses

To support meta‐analysis (and narrative synthesis) several studies [43, 44, 45, 55] required their extracted data to be ‘transformed’. All these transformations have been transparently documented and justified and are available in Supporting Information S1: Material 9.

#### 3.4.2 Overall Health‐Related Quality for Adults Diagnosed With ALS

Two studies utilised the EQ‐5D‐5L instrument to provide utility‐based valuations of overall hrQoL [43, 55]. One study utilised a UK preference set [55], and reported an overall hrQoL of 0.57 (95%CI: 0.55 to 0.59). This finding was associated with high certainty evidence (Table 1). A second study utilised a Chinese preference set [43] and reported an overall hrQoL of 0.72 (95%CI: 0.70 to 0.74).

**Table 1:** Outcomes that have been meta‐analysed or presented in a forest‐plot.

Summary of findings			
Outcomes that have been meta‐analysed or presented in a forest‐plot			
Health state/outcome valuation	Estimate of health state/outcome valuation	Certainty in evidence	Interpretation of findings
	No. of participants/studies		
*Health‐related quality of life (UK preference set)*			
Overall health‐related quality of life	Mean EQ. 5D Health State Utility = 0.57 (95%CI: 0.55 to 0.59) [55]. (Supporting Information S1: Material 6)	⊕⊕⊕⊕ High certainty	Adults with ALS from the UK may have a mean valuation of their health‐related quality of life of approximately 0.57.
	595 participants/1 study		
*Health‐related quality of life (Chinese preference set)*			
Overall health‐related quality of life	Mean EQ. 5D Health State Utility = 0.72 (95%CI: 0.70 to 0.74) [43]. (Supporting Information S1: Material 6)	⊕⊕◯◯^a^ Low certainty	Adults with ALS from China may have a mean valuation of their health‐related quality of life of approximately 0.72.
	441 participants/1 study		
*Health‐related quality of life (King's staging)*			
Health‐related quality of life [King's Staging 1]	Mean EQ. 5D Health State Utility = 0.71 (95%CI: 0.68 to 0.74) [44, 55]. (Figure 2)	⊕◯◯◯^b^^,^ c Very low certainty	Adults with MND at King's stage 1 may have a mean valuation of their health‐related quality of life of approximately 0.71, but the evidence is very uncertain.
	118 participants/2 studies		
Health‐related quality [King's Staging 2]	Mean EQ. 5D Health State Utility = 0.56 (95%CI: 0.54 to 0.59) [44, 55]. (Figure 2)	⊕◯◯◯^b^^,^ c Very low certainty	Adults with MND at King's stage 2 may have a mean valuation of their health‐related quality of life of approximately 0.56, but the evidence is very uncertain.
	162 participants/2 studies		
Health‐related quality of life [King's Staging 3]	Mean EQ. 5D Health State Utility = 0.49 (95%CI: 0.47 to 0.52) [44, 55]. (Figure 2)	⊕◯◯◯^b^^,^ c Very low certainty	Adults with MND at King's stage 3 may have a mean valuation of their health‐related quality of life of approximately 0.49, but the evidence is very uncertain.
	262 participants/2 studies		
Health‐related quality of life [King's Staging 4]	Mean EQ. 5D Health State Utility = 0.40 (95%CI: 0.36 to 0.44) [44, 55]. (Figure 2)	⊕◯◯◯^b^^,^ c ^,^ d Very low certainty	Adults with MND at King's stage 4 may have a mean valuation of their health‐related quality of life of approximately 0.40, but the evidence is very uncertain.
	212 participants/2 studies		
Health‐related quality of life [King's Staging Overall]	Mean EQ. 5D Health State Utility = 0.52 (95%CI: 0.44 to 0.60) [44, 55]. (Figure 2)	⊕⊕◯◯^b^^,^ c ^,^ e Low certainty	Adults with MND at any stage of disease progression (King's) may have a mean valuation of their health‐related quality of life of approximately 0.52.
	754 participants/2 studies		
*Health‐related quality of life (MiToS staging)*			
Health‐related quality of life [MiToS Staging 0]	Mean EQ. 5D Health State Utility = 0.62 (95%CI: 0.44 to 0.80) [44, 55]. (Figure 3)	⊕◯◯◯^b^^,^ c ^,^ d Very low certainty	Adults with MND at stage 0 (MiToS) may have a mean valuation of their health‐related quality of life of approximately 0.62, but the evidence is very uncertain.
	417 participants/2 studies		
Health‐related quality of life [MiToS Staging 1]	Mean EQ. 5D Health State Utility = 0.43 (95%CI: 0.29 to 0.56) [44, 55]. (Figure 3)	⊕◯◯◯^b^^,^ c ^,^ d Very low certainty	Adults with MND at stage 1 (MiToS) may have a mean valuation of their health‐related quality of life of approximately 0.43, but the evidence is very uncertain.
	215 participants/2 studies		
Health‐related quality of life [MiToS Staging 2]	Mean EQ. 5D Health State Utility = 0.24 (95%CI: −0.09 to 0.57) [44, 55]. (Figure 3)	⊕◯◯◯^b^^,^ c ^,^ d Very low certainty	Adults with MND at stage 2 (MiToS) may have a mean valuation of their health‐related quality of life of approximately 0.24, but the evidence is very uncertain.
	83 participants/2 studies		
Health‐related quality of life [MiToS Staging 3]	Mean EQ. 5D Health State Utility = 0.29 (95%CI: 0.10 to 0.49) [44, 55]. (Figure 3)	⊕◯◯◯^b^^,^ c ^,^ d Very low certainty	Adults with MND at stage 3 (MiToS) may have a mean valuation of their health‐related quality of life of approximately 0.29, but the evidence is very uncertain.
	26 participants/2 studies		
Health‐related quality of life [MiToS Staging 4]	Mean EQ. 5D Health State Utility = 0.12 (95%CI: −0.22 to 0.45) [44, 55]. (Figure 3)	⊕◯◯◯^b^^,^ c ^,^ d Very low certainty	Adults with MND at stage 4 (MiToS) may have a mean valuation of their health‐related quality of life of approximately 0.12, but the evidence is very uncertain.
	19 participants/2studies		
Health‐related quality of life [MiToS Staging Overall]	Mean EQ. 5D Health State Utility = 0.37 (95%CI: 0.27 to 0.47) [44, 55]. (Figure 3)	⊕◯◯◯^b^^,^ c Very low certainty	Adults with MND at any stage of disease progression (MiToS) may have a mean valuation of their health‐related quality of life of approximately 0.27, but the evidence is very uncertain.
	760 participants/2 studies		
*Health‐related quality of life (ALS Health State Scale)*			
Health‐related quality of life [ALS HSS Level 1]	Mean EQ. 5D Health State Utility = 0.74 (95%CI: 0.64 to 0.84) [41]. (Supporting Information S1: Material 6)	⊕⊕⊕◯^f^ Moderate certainty	Adults with MND at level 1 (ALS HSS) probably have a mean valuation of their health‐related quality of life of approximately 0.74.
	15 participants/1 study		
Health‐related quality of life [ALS HSS Level 2]	Mean EQ. 5D Health State Utility = 0.63 (95%CI: 0.56 to 0.70) [41]. (Supporting Information S1: Material 6)	⊕⊕⊕◯^f^ Moderate certainty	Adults with MND at level 2 (ALS HSS) probably have a mean valuation of their health‐related quality of life of approximately 0.63.
	21 participants/1 study		
Health‐related quality of life [ALS HSS Level 3]	Mean EQ. 5D Health State Utility = 0.51 (95%CI: 0.43 to 0.59) [41]. (Supporting Information S1: Material 6)	⊕⊕⊕◯^f^ Moderate certainty	Adults with MND at level 3 (ALS HSS) probably have a mean valuation of their health‐related quality of life of approximately 0.51.
	22 participants/1 study		
Health‐related quality of life [ALS HSS Level 4]	Mean EQ. 5D Health State Utility = 0.37 (95%CI: 0.29 to 0.45) [41]. (Supporting Information S1: Material 6)	⊕⊕⊕◯^f^ Moderate certainty	Adults with MND at level 4 (ALS HSS) probably have a mean valuation of their health‐related quality of life of approximately 0.37.
	19 participants/1 study		
Health‐related quality of life [ALS HSS Overall]	Mean EQ. 5D Health State Utility = 0.56 (95%CI: 0.41 to 0.71) [41]. (Supporting Information S1: Material 6)	⊕◯◯◯^c^^,^ f Very low certainty	Adults with MND at any stage of disease progression (ALS HSS) may have a mean valuation of their health‐related quality of life of approximately 0.56, but the evidence is very uncertain.
	77 participants/1 study		
*Health‐related quality of life (Functional Test 9)*			
Health‐related quality of life [FT9 0]	Mean EQ. 5D Health State Utility = 0.79 (95%CI: 0.76 to 0.81) [64]. (Supporting Information S1: Material 6)	⊕⊕⊕⊕ High certainty	Adults with ALS (FT9 0) have a mean valuation of their health‐related quality of life of approximately 0.79.
	160 participants/1 study		
Health‐related quality of life [FT9 1]	Mean EQ. 5D Health State Utility = 0.74 (95%CI: 0.73 to 0.76) [64]. (Supporting Information S1: Material 6)	⊕⊕⊕⊕ High certainty	Adults with ALS (FT9 1) have a mean valuation of their health‐related quality of life of approximately 0.74.
	494 participants/1 study		
Health‐related quality of life [FT9 2]	Mean EQ. 5D Health State Utility = 0.63 (95%CI: 0.61 to 0.65) [64]. (Supporting Information S1: Material 6)	⊕⊕⊕⊕ High certainty	Adults with ALS (FT9 2) have a mean valuation of their health‐related quality of life of approximately 0.63.
	542 participants/1 study		
Health‐related quality of life [FT9 3]	Mean EQ. 5D Health State Utility = 0.54 (95%CI: 0.52 to 0.56) [64]. (Supporting Information S1: Material 6)	⊕⊕⊕⊕ High certainty	Adults with ALS (FT9 3) have a mean valuation of their health‐related quality of life of approximately 0.54.
	381 participants/1 study		
Health‐related quality of life [FT9 4]	Mean EQ. 5D Health State Utility = 0.46 (95%CI: 0.43 to 0.49) [64]. (Supporting Information S1: Material 6)	⊕⊕⊕⊕ High certainty	Adults with ALS (FT9 4) have a mean valuation of their health‐related quality of life of approximately 0.46.
	291 participants/1 study		
Health‐related quality of life [FT9 Overall]	Mean EQ. 5D Health State Utility = 0.63 (95%CI: 0.53 to 0.74) [64]. (Supporting Information S1: Material 6)	⊕⊕⊕⊕ High certainty	Adults with ALS at any stage of disease progression (FT9) may have a mean valuation of their health‐related quality of life of approximately 0.63.
	1868 participants/1 study		

Outcomes that have been narrativley synthesised			
*Living with MND*			
Living with ALS (Appel Score = 40 to 59)	A standard gamble exercise was used to elicit preferences of clinicians serving as proxy to adults living with ALS, to living in specific health states [56]. Health state descriptions were derived from the 5 clinical stages that were mapped to the Appel ALS rating scale, a disease‐specific classification system. This health state (Appel score from 40 to 59) had the following health state description: normal to slightly slurred speech; general diet but need to eliminate hard foods that are difficult to swallow; normal breathing; dress and feed independently and can live independently; may require mobility aids such as a cane; able to work full time; medical procedures are strictly diagnostic. Mean valuation of 0.89 ± 0.04	⊕⊕◯◯^g^^,^ h Low certainty	According to clinicians serving as proxy to adults with ALS (with an Appel Score of 40–59) they may have a mean valuation of living with ALS of 0.89 ± 0.04.
	10 participants/1 study		
Living with ALS (Appel Score = 60 to 86)	A standard gamble exercise was used to elicit preferences of clinicians serving as proxy to adults living with ALS, to living in specific health states [56]. Health state descriptions were derived from the 5 clinical stages that were mapped to the Appel ALS rating scale, a disease‐specific classification system. This health state (Appel score from 60 to 86) had the following health state description: speech is intelligible at least three‐quarters of the time; diet limited to finely chopped or ground foods and thick liquids; breathing is moderately impaired, experience shortness of breath on exertion, but can cough effectively; require minimal care for dressing and feeding; require mobility aids such as crutches or occasionally a wheelchair; work limited to part time; medical procedures include chest physiotherapy and diagnostic spirometry. Mean valuation of 0.82 ± 0.04	⊕⊕◯◯^g^^,^ h Low certainty	According to clinicians serving as proxy to adults with ALS (with an Appel Score of 60‐86) they may have a mean valuation of living with ALS of 0.82 ± 0.04.
	10 participants/1 study		
Living with ALS (Appel Score = 87 to 109)	A standard gamble exercise was used to elicit preferences of clinicians serving as proxy to adults living with ALS, to living in specific health states [56]. Health state descriptions were derived from the 5 clinical stages that were mapped to the Appel ALS rating scale, a disease‐specific classification system. This health state (Appel score from 87 to 109) had the following health state description: speech is intelligible less than half of the time; diet limited to food of pudding consistency; breathing impaired to point at increased risk for infections; require caretaker to assist with transfers and to assist with dressing and feeding; require use of a wheelchair much of the time; most likely have stopped working; medical care includes PEG consultations, medications for pain, and many unexpected hospitalisations. Mean valuation of 0.41 ± 0.17	⊕⊕◯◯^g^^,^ h Low certainty	According to clinicians serving as proxy to adults with ALS (with an Appel Score of 87–109) they may have a mean valuation of living with ALS of 0.41 ± 0.17.
	10 participants/1 study		
Living with ALS (Appel Score = 110 to 128)	A standard gamble exercise was used to elicit preferences of clinicians serving as proxy to adults living with ALS, to living in specific health states [56]. Health state descriptions were derived from the 5 clinical stages that were mapped to the Appel ALS rating scale, a disease‐specific classification system. This health state (Appel score from 110 to 128) had the following health state description: speech is intelligible less than one‐quarter of the time or requires a device to communicate; diet is limited to pureed, blended, thick liquids and likely to need tube feedings; respiratory function is impaired to the extent that pressure support (CPAP, BIPAP) and home oxygen may be needed; fully dependent on a caregiver for dressing and feeding; wheelchair‐bound; not working; medical care includes consultations regarding need for tracheostomy/gastrostomy, as well as medications for pain. Mean valuation of 0.01 ± 0.20	⊕⊕◯◯^g^^,^ h Low certainty	According to clinicians serving as proxy to adults with ALS (with an Appel Score of 110–109) they may have a mean valuation of living with ALS of 0.01 ± 0.20.
	10 participants/1 study		
Living with ALS (Appel Score = 129 to 164)	A standard gamble exercise was used to elicit preferences of clinicians serving as proxy to adults living with ALS, to living in specific health states [56]. Health state descriptions were derived from the 5 clinical stages that were mapped to the Appel ALS rating scale, a disease‐specific classification system. This health state (Appel score from 129 to 164) had the following health state description: unable to speak; have had a gastrostomy; respiratory function severely impaired; have had a tracheostomy; fully dependent on a caregiver for dressing and feeding; quadriplegic; not working; most likely in a long‐term care facility; medical care includes medications for pain. Mean valuation of −0.53 ± 0.14	⊕⊕◯◯^g^^,^ h Moderate certainty	According to clinicians serving as proxy to adults with ALS (with an Appel Score of 129–164) they may have a mean valuation of living with ALS of −0.53 ± 0.14.
	10 participants/1 study		
Day to day living	Preferences were elicited through surveys and interviews. The outcome of daily activity received a normalised ranking score of 0.21 across all countries, with country‐specific scores ranging from 0.21 to 0.24 [45]. One study reported 9.8% of participants identified impairment of activities of daily living as the worst aspect of disease at baseline, and 3.2% at follow‐up [59]	⊕⊕◯◯^i^^,^ j Low certainty	Adults with ALS may slightly prefer interventions that support their day to day living.
	373 participants/2 studies		
*Physical functioning and mobility*			
Physical functioning and mobility	Preferences were elicited through surveys and interviews. Reported proportions identifying physical functioning and mobility as key concerns ranged from 16.7% to 29% [46, 59]. One study reported that 49 participants cited mobility as a treatment priority [47].	⊕⊕◯◯^i^^,^ j Very low certainty	Adults with ALS and their caregivers may prefer preservation of physical functioning and mobility, but the evidence is very uncertain
	1025 participants/3 studies		
*Genetic testing*			
Genetic testing	Preferences were elicited through an online survey. Agreement with the value of genetic testing ranged from 80.1% to 83.3%, with most participants indicating they would undergo testing again and recommend it to others [48].	⊕⊕◯◯^k^ Low certainty	Adults with ALS may prefer access to genetic testing.
	449 participants/1 study		
*Cognition*			
Cognition	Preferences were elicited through surveys and interviews. Reported proportions identifying cognition as a treatment priority ranged from 2.4% to 3.2% [47, 59]. One study reported that 6 participants cited cognitive improvement as a treatment priority.	⊕⊕◯◯^i^ Low certainty	Adults with ALS may slightly prefer interventions targeting cognitive function.
	898 participants/2 studies		
*Psychological and emotional wellbeing*			
Mental health and mood	Preferences were elicited through surveys and interviews. The importance of mood as a component of quality of life ranged from 36.4% to 38.1% [60] One study reported that 4 participants cited mental health as a treatment priority [47].	⊕⊕◯◯^i^ Low certainty	Adults with ALS may prefer support for mental health and mood.
	888 participants/2 studies		
Provision of emotional support	Preferences were elicited through a discrete choice experiment. Coefficients for emotional support varied by disease stage and delivery method, with no important differences in preferences identified [62].	⊕⊕⊕⊕ High certainty	Adults with ALS and their caregivers have no strong preferences regarding their access to emotional support.
	149 participants/1 study		
Emotional swings	Preferences were elicited through structured surveys. Emotional swings were identified as a top concern by 13.7% to 16.7% of participants in one study [46]. One study also reported that 1 participant cited uncontrollable laughing and/or crying as a treatment priority [47].	⊕◯◯◯^i^^,^ j Very low certainty	Adults with ALS and their caregivers may slightly prefer interventions address emotional swings.
	984 participants/2 studies		
Anxiety	Preferences were elicited through a structured survey. The mean importance scores for anxiety were 45.4% (gastronomy‐fed) and 28.6% (eating orally) [60].	⊕⊕◯◯^i^ Low certainty	Adults with ALS may prefer interventions that reduce anxiety.
	31 participants/1 study		
Depression	Preferences were elicited through a structured survey. Depression was identified as a top concern by 3% to 5.1% of participants [46].	⊕◯◯◯^i^^,^ l Very low certainty	Adults with ALS and their caregivers may slightly prefer interventions targeting depression, but the evidence is very uncertain.
	127 participants/1 study		
Loss of faith	Preferences were elicited through a structured survey. Loss of faith was identified as a top concern by 1.5% of participants [46].	⊕◯◯◯^i^^,^ l Very low certainty	Adults with ALS and their caregivers may slightly prefer support for spiritual wellbeing or interventions addressing loss of faith, but the evidence is very uncertain.
	127 participants/1 study		
Apathy	Preferences were elicited through a structured survey. Apathy was cited as a treatment priority by 3 participants [47].	⊕◯◯◯^i^^,^ m Very low certainty	Adults with ALS may slightly prefer interventions targeting apathy, but the evidence is very uncertain.
	857 participants/1 study		
Financial Burden	Preferences were elicited through a structured survey and a discrete choice experiment. Financial burden was identified as a top concern by 13.7% to 16.7% of participants in one study [46]; the second study reported no strong preferences regarding cost of services [62].	⊕⊕◯◯^n^^,^ o Low certainty	Adults with ALS and their caregivers may prefer support to reduce financial burden.
	276 participants/2 studies		
Concern for future	Preferences were elicited through structured and semi‐structured interviews. Concern for the future was identified as a top concern by 5% to 25.4% of participants [46, 49].	⊕◯◯◯^i^^,^ j ^,^ o Very low certainty	Adults with ALS and PLS may prefer support that address concerns about their future, but the evidence is very uncertain
	182 participants/2 studies		
*Swallowing and nutrition*			
Eating score	Preferences were elicited through a retrospective analysis of RCT data. One study reported eating score over time, with a mean eating score (ALSSQoL) of 0.91 (±1.54) at baseline and 2.54 (±3.09) at week 96 [50].	⊕⊕⊕⊕ High certainty	Adults with ALS prefer interventions that support their eating.
	82 participants/1 study		
Flavour perception	Preferences were elicited through surveys and interviews. 75% of participants reported flavour perception as being 75 or higher (on a scale of 100) in terms of importance [60].	⊕⊕◯◯^k^ Low certainty	Adults with ALS may have strong preferences for interventions that support their flavour perception.
	31 participants/1 study		
Taste	Preferences were elicited through surveys and interviews. Mean importance scores for taste were 76.19% for orally fed and 54.5% for gastronomy‐fed participants [60]	⊕⊕◯◯^k^ Low certainty	Adults with ALS may prefer interventions that support their retention of taste.
	31 participants/1 study		
Swallowing and chewing	Preferences were elicited through surveys and interviews. Participants that identified swallowing and chewing as key areas of importance ranged from 2.4% to 30.5% [46, 59]. Mean importance scores ranged from 9.52% to 28.57% for those that were orally fed and 90.91% to 100% for those that were gastronomy‐fed [60]. One study reported that 7 participants cited swallowing as a treatment priority [47].	⊕◯◯◯^i^^,^ j ^,^ o Very low certainty	Adults with ALS and their caregivers may have varying preferences for interventions that support their swallowing and chewing, but the evidence is very uncertain.
	1056 participants/4 studies		
Body weight	Preferences were elicited through a survey. The outcome of body weight received a normalised ranking score of 0.16 across all countries, with country‐specific scores ranging from 0.15 (Netherlands) to 0.21 (Australia) [45].	⊕⊕⊕⊕ High certainty	Adults with ALS slightly prefer interventions that support their body weight.
	332 participants/1 study		
*Personal care and daily activities*			
Independence	Preferences were elicited through surveys and interviews. Reported proportions identifying independence as a key concern ranged from 6.8% to 37.9% among participants [49, 59]. Another study reported that 12.2% of participants identified dependence on others as the worst aspect at baseline, and 9.7% at follow‐up [46].	⊕◯◯◯^i^^,^ j Very low certainty	Adults with ALS and their caregivers may have varying preferences of interventions that support their independence, but the evidence is very uncertain.
	223 participants/3 studies		
Recreation	Preferences were elicited through surveys and interviews. Mean importance scores for recreation were 45.4% for gastronomy‐fed participants and 52.4% for eating orally participants [60].	⊕◯◯◯^k^^,^ p Very low certainty	Adults with ALS may have varying preferences for interventions that support their recreation, but the evidence is very uncertain.
	31 participants/1 study		
Ability to work	Preferences were elicited through surveys and interviews. Reported proportions identifying ability to work as a key concern ranged from 1.7% to 22% among participants [49, 59].	⊕◯◯◯^i^^,^ j Very low certainty	Adults with ALS and their caregivers may have varying preferences of interventions that support their ability to work, but the evidence is very uncertain.
	96 participants/2 studies		
Daily self‐care	Preferences were elicited through surveys. One study found that 18.2% of adults with ALS and 22% of caregivers listed difficulty with self‐care as one of their top three concerns [46]. One study reported that 28 participants wanted their current ALS treatment to impact their daily self‐care [47].	⊕◯◯◯^i^^,^ j Very low certainty	Adults with ALS and their caregivers may have varying preferences of interventions that support their daily self‐care, but the evidence is very uncertain.
	984 participants/2 studies		
Ability to write	Preferences were elicited through interviews. Loss of writing ability was identified as the worst aspect of the disease by 2.4% of adults with ALS at baseline and by 3.2% at follow‐up [59].	⊕◯◯◯^i^^,^ p Very low certainty	Adults with ALS may have slight preferences of interventions that support their ability to write, but the evidence is very uncertain.
	41 participants/1 study		
Appearance	Preferences were elicited through a surveys and interviews. Mean importance scores for appearance was 54.5% for gastronomy‐fed participants and 4.76% for orally fed participants [60].	⊕◯◯◯^i^^,^ p Very low certainty	Adults with ALS may have varying preferences for interventions that support their appearance, but the evidence is very uncertain.
	31 participants/1 study		
Being able to travel	Preferences were elicited through interviews. One study reported that 10% of participants in pain and 7% of those not in pain identified the ability to travel as one of their top five most important concerns [49].	⊕◯◯◯^i^^,^ q Very low certainty	Adults with ALS and PLS slightly prefer interventions that support their ability to travel
	55 participants/1 study		
Personal care provision	Preferences were elicited through a discrete choice experiment. There were no important preferences observed for participants in terms of how their personal care was provided [62].	⊕⊕⊕◯^j^ Moderate certainty	Adults with ALS and their caregivers probably have no strong preference towards how their personal care is provided to them.
	149 participants/1 study		
*Symptom management*			
Pain	Preferences were elicited through surveys and interviews and the retrospective analysis of RCT data. One study found mean importance scores for pain of 9.1% (gastronomy‐fed) and 9.5% (eating orally) [60]. A second study reported mean pain scores of 1.6 (±2.27) at baseline and 2.5 (±2.37) at week 96 [50]. The final study reported that only a single participant wanted their current ALS treatments to impact their pain [47].	⊕⊕◯◯^r^ Low certainty	Adults with ALS may slightly prefer interventions that support their pain.
	970 participants/3 studies		
Breathing	Preferences were elicited through surveys and interviews and the retrospective analysis of RCT data. One study found that breathing was listed as a top three concern for 13.6% to 25.4% of participants [46]. One study reported a normalised ranking score for breathing of 0.63 across all countries (from 0.5 to 0.63) [45]. Another study found mean breathing scores of 0.67 (±1.33) at baseline and 2.83 (±2.77) at week 96 [50]. One study reported that 90 participants wanted current ALS treatments to impact their breathing [47].	⊕◯◯◯^i^^,^ r ^,^ s Very low certainty	Adults with ALS may have varying preferences for interventions that support their breathing, but the evidence is very uncertain.
	1398 participants/4 studies		
Muscle spasticity	Preferences were elicited through a structured survey. Muscle spasticity was cited as a treatment priority by 2 participants [47].	⊕◯◯◯^k^^,^ m Very low certainty	Adults with ALS may slightly prefer interventions targeting muscle spasticity, but the evidence is very uncertain.
	857 participants/1 study		
Muscle strength	Preferences were elicited through surveys and the retrospective analysis of RCT data. One study reported a normalised ranking score for muscle strength of 0.39 across all countries (from 0.34 to 0.39) [45]. 22% to 25.8% of participants listed muscle weakness as a top three concern [46]. Another study reported mean strength/ability to move scores of 3.96 (±2.57) at baseline and 6.46 (±2.36) at week 96 [50]. When presented with a hypothetical drug, 23.9% preferred the drug increased muscle strength (despite severe side effects), and 24.5% preferred it prevented further weakening (without improvement). The same study reported 23 participants wanted their current ALS treatments to impact muscle weakness [47].	⊕◯◯◯^j^^,^ r ^,^ s Very low certainty	Adults with ALS may have varying preferences for interventions that support their muscle strength, but the evidence is very uncertain.
	1398 participants/4 studies		
Improved symptoms	Preferences were elicited through surveys and a standard gamble utility elicitation exercise. The probability of choosing to gamble guaranteed survival for 2 years against a cure with risk of death, ranged from 0.45 to 0.79, however this changed depending on MND ‘level’ (level 1–4) [41]. One study reported that 28.9% of participants reported that slowing the spread of ALS/MND to be the most important outcome of a new drug and 14.2% of participants reported that reduction of symptoms to be the most important outcome. The same study reported that 20 participants wanted their current treatment to ease the overall burden of disease and 161 wanted it to stop progression [47].	⊕⊕⊕◯^n^ Moderate certainty	Adults with ALS and their caregivers probably have slight preference towards interventions that improve their symptoms of the disease.
	934 participants/2 studies		
Fatigue	Preferences were elicited through surveys and the retrospective analysis of RCT data. The mean fatigue scores were 3.24 (±2.24) at baseline and 4.38 (±2.22) at week 96 [50]. One study reported that 15 participants wanted their current ALS treatments to impact fatigue [47].	⊕⊕◯◯^n^^,^ s Low certainty	Adults with ALS may slightly prefer interventions that target their fatigue.
	939 participants/2 studies		
Sialorrhea	Preferences were elicited through surveys and the retrospective analysis of RCT data. One study reported mean importance scores for sialorrhea (saliva) of 18.18% (gastronomy‐fed) and 4.76% (eating orally) [60]. Another study reported mean scores of 1.12 (±1.92) at baseline and 2.54 (±2.93) at week 96 [50].	⊕⊕◯◯^n^^,^ s Low certainty	Adults with ALS may have varying preferences for interventions that target their salvia.
	113 participants/2 studies		
Bowel and bladder	Preferences were elicited through the retrospective analysis of RCT data. A mean bowel and bladder score were reported of 1.12 (±1.92) at baseline and 2.54 (±2.93) at week 96 [50].	⊕⊕⊕⊕ High certainty	Adults with ALS slightly prefer interventions that target their bowel and bladder.
	82 participants/1 study		
Mucus	Preferences were elicited through the retrospective analysis of RCT data. A mean mucus score was reported of 1.24 (±1.73) at baseline and 2.74 (±2.84) at week 96 [50].	⊕⊕⊕⊕ High certainty	Adults with ALS slightly prefer interventions that target their mucus.
	82 participants/1 study		
Sleep	Preferences were elicited through surveys and the retrospective analysis of RCT data. One study reported a normalised ranking score for sleep of 0.17 across all countries, ranging from 0.11 to 0.39 [45]. Another study reported mean sleep scores of 1.44 (±1.9) at baseline and 2.85 (±2.54) at week 96 [50].	⊕⊕⊕◯^s^ Moderate certainty	Adults with ALS probably have varying preferences towards interventions that target their sleep.
	414 participants/2 studies.		
*Disease modifying therapies*			
Riluzole	Preferences were elicited through interviews. Reported reasons included insufficient benefit (*n* = 14), high cost (*n* = 9), not wanting to prolong life (*n* = 8), perceived worsening condition (*n* = 2), potential side effects (*n* = 2), lack of quality of life improvement (*n* = 1), and participation in another study (*n* = 1) [51].	⊕◯◯◯^k^^,^ m Very low certainty	Adults with ALS may have varying preferences when considering using riluzole, but the evidence is very uncertain.
	46 participants/1 study		
Intrathecal drug delivery	Preferences were elicited through a discrete choice experiment and threshold technique. Implanted drug delivery devices (IDDD) were the preferred intervention over lumbar puncture in 69% to 82% of choices. In total, 78.7% of discrete choice experiment tasks favoured IDDD [63].	⊕⊕⊕⊕ High certainty	Adults with ALS prefer the outcomes associated with IDDD over lumbar puncture for intrathecal drug delivery.
	295 participants/1 study		
*Communication and expression*			
Speech	Preferences were elicited through surveys, interviews, and retrospective analysis of RCT data. One study reported a normalised ranking score for fine motor/speech of 0.22 across all countries (range: 0.19 to 0.25) [45]. 29.3% of adults with ALS identified speech reduction as the worst aspect of disease at baseline, and 19.4% at follow‐up [59]. Speech was rated 90.9% important among gastronomy‐fed adults and 42.9% among adults that were eating orally [60]. 27.3% of adults with ALS and 17% of caregivers ranked speaking among their top three concerns [46]. Mean speaking scores were 1.85 (±2.59) at baseline and 4.3 (±3.77) at week 96 [50]. 33 participants reported wanting their current ALS treatments to improve speech [47].	⊕◯◯◯^r^^,^ s Very low certainty	Adults with ALS may slightly prefer interventions that target their speech, but the evidence is very uncertain
	1470 participants/6 studies		
Use of communication technologies	Preferences were elicited through a discrete choice experiment. Female participants showed a stronger preference for using communication devices (coefficient: −0.25, 95% CI: −0.47 to −0.21), while males showed no clear preference for any attribute [62].	⊕⊕⊕⊕ High certainty	Female adults with ALS prefer the use of voice banking. Male adults with ALS have no strong preference towards the use of voice banking.
	93 participants/1 study		
*Healthcare experiences*			
Availability of phone advice	Preferences were elicited through a discrete choice experiment. There were no strong preferences regarding having phone advice available 24 h a day (stage 1–2 (coefficient: 0.03, 95% CI: −0.35 to 0.41)) (stage 3–4 ((coefficient: 0.05, 95% CI: −0.18 to 0.28)). There were no strong preferences regarding having phone advice available during business hours (stage 1–2 (coefficient: 0.14, 95% CI: −0.15 to 0.43)) (stage 3–4 (coefficient: −0.17, 95% CI: −0.33 to 0.00)) [62].	⊕⊕⊕⊕ High certainty	Adults with ALS have no strong preference regarding the availability or timing of phone advice.
	93 participants/1 study		
Dependence on healthcare providers	Preferences were elicited through a discrete choice experiment. There were no strong preferences regarding the need to depend on healthcare providers (stage 1–2 (coefficient: 0.36, 95% CI: −0.00 to 0.72)) (stage 3–4 (coefficient: 0.02, 95% CI: −0.20 to 0.25)) [62].	⊕⊕⊕⊕ High certainty	Adults with ALS have no strong preference regarding needing to depend on healthcare providers.
	93 participants/1 study		
Disease information	Preferences were elicited through a discrete choice experiment. Participants at stage 1–2 showed a strong preference for receiving information about MND at the time of diagnosis, rather than later when clinically relevant (coefficient: −0.51, 95% CI: −0.99 to −0.05), while no important differences in preferences were identified at stage 3–4 (coefficient: −0.12, 95% CI: −0.37 to 0.13). Receiving information at diagnosis was also strongly preferred over not receiving any information at all for those at stage 1–2 (coefficient: −0.57, 95% CI: −1.04 to −0.10), but no important differences were observed at stage 3–4 (coefficient: −0.05, 95% CI: −0.31 to 0.21) [62].	⊕⊕⊕⊕ High certainty	Adults with ALS (staged at 1 or 2 [King's]) prefer receiving information regarding MND at the time of diagnosis. Adults with ALS (staged at 3 or 4 [King's]) have no strong preference regarding when they receive information regarding MND.
	93 participants/1 study		
Timing of hospice care	Preferences were elicited through a discrete choice experiment. There were no strong preferences regarding how early in the illness to begin hospice care (stage 1–2 (coefficient: 0.24, 95% CI: −0.05 to 0.53)) (stage 3–4) (coefficient: 0.14, 95% CI: −0.03 to 0.30)).	⊕⊕⊕⊕ High certainty	Adults with ALS have no strong preference regarding the timing of their hospice care.
	93 participants/1 study		
Waiting times	Preferences were elicited through a discrete choice experiment. There were no strong preferences regarding attendance at multidisciplinary clinics with long waiting times. (stage 1–2 (coefficient: −0.05, 95% CI: −0.39 to 0.29)) (stage 3–4 (coefficient: 0.06, 95% CI: −0.14 to 0.27)) [62].	⊕⊕⊕⊕ High certainty	Adults with ALS have no strong preference regarding their waiting times when attending clinics.
	93 participants/1 study		
“Helplessness” of medicine	Preferences were elicited through interviews. 2.4% of adults with ALS identified helplessness of medicine as the worst aspect of the disease at baseline, and 3.2% at six‐month follow‐up [59].	⊕⊕◯◯^k^ Low certainty	Adults with ALS may have slight preferences towards interventions that target the feeling of helplessness.
	41 participants/1 study		
*Prognosis*			
Survival (prolonging life)	Preferences were elicited through surveys and interviews. One study reported mean scores for the importance of living at 7.8 (±3.1) for people with ALS and 8.0 (±3.0) for caregivers at first assessment, and 7.5 (±3.4) and 6.9 (±3.3), respectively, at last assessment [61]. 69.5% of caregivers supported the use of life‐prolonging measures for their relative with ALS [52]. 8.5% of adults with ALS identified survival prolongation, despite severe side effects as the most important outcome of a hypothetical new drug [47].	⊕◯◯◯^i^^,^ s Very low certainty	Adults with ALS and their caregivers may prefer interventions that increase their chance of survival, but the evidence is uncertain.
	1159 participants/3 studies		
Hastening death	Preferences were elicited through surveys and interviews. Mean scores for interest in hastening death were 3.2 (±3.3) for people with ALS and 2.7 (±2.8) for caregivers at first assessment, and 3.1 (±3.3) and 2.7 (±2.8), respectively, at last assessment [61]. 38.7% of caregivers supported the use of life‐shortening measures if legally permitted [52].	⊕◯◯◯^i^^,^ s Very low certainty	Adults with ALS and their caregivers may prefer to explore methods of hastening their own death, but the evidence is uncertain.
	302 participants/2 studies		
*Social and interpersonal relationships*			
Isolation and loneliness	Preferences were elicited through surveys and interviews. 6.5% of adults with ALS identified social isolation as the worst aspect of the disease at six‐month follow‐up, while none reported it at baseline [59]. Another study found that 3% of adults with ALS and 3% of caregivers listed loneliness as one of their top three concerns [46].	⊕◯◯◯^i^^,^ s Very low certainty	Adults with ALS may slightly prefer interventions that target social isolation and loneliness, but the evidence is uncertain
	168 participants/2 studies		
Who helps in the home	Preferences were elicited through a discrete choice experiment. It was narratively reported that adults with ALS and their caregivers showed no strong preference regarding whether non‐related individuals helped in the home [62].	⊕⊕⊕⊕ High certainty	Adults with ALS have no strong preference regarding who helps in the home.
	93 participants/1 study		
Sexual function	Preferences were elicited through a survey. One study reported that a single adult with ALS indicated they wanted their current ALS treatments to improve sexual function [47].	⊕⊕◯◯^k^ Low certainty	Adults with ALS may have slight preferences towards interventions that target their sexual function.
	857 participants/1 study		
*Domain importance*			
Domain importance	Preferences were elicited through surveys. 37.5% of adults with ALS ranked respiratory function as the most important domain, followed by bulbar (30.9%), gross motor (20.4%), and fine motor (11.2%) [53]. In a second study, 37% reported no domain preference, while the remaining participants prioritised respiratory (23%), bulbar (22%), gross motor (10%), and fine motor (8%) domains [54].	⊕⊕⊕⊕ High certainty	Most adults with ALS demonstrate no strong preference regarding domain importance. Where adults with ALS do demonstrate a preference regarding domain importance: Most adults prefer interventions that target the respiratory domain. Some adults prefer interventions that target the bulbar and gross motor domains. Few adults prefer interventions that target the fine motor domain.
	815 participants/2 studies		

^a^ Downgraded one level for risk of bias—this single study had extremely serious concerns with risk of bias.

^b^ Downgraded one level for indirectness—one study has included adults with a diagnosis of ‘MND’, the other study has included adults with a diagnosis of ‘ALS’.

^c^ Downgraded two levels for inconsistency—*I* ^2^ > 90%, significant *Q* test at the 0.001 significance level.

^d^ Downgraded one level for imprecision—the confidence intervals are very wide and may have crossed several important decision‐making thresholds.

^e^ Upgraded one level for dose‐response gradient—there were clear differences in the pooled estimates for EQ. 5D measurements dependent on King's staging. As per the GRADE guidance [14] upgrading your certainty when you haven't previously downgraded for risk of bias or imprecision can be appropriate, if applicable.

^f^ Downgraded two levels for imprecision—only one study with a small number of participants has contributed to this outcome.

^g^ Downgraded one level for indirectness—the single contributing study has provided outcome valuations provided by clinicians as proxy to adults with ALS.

^h^ Downgraded one level for imprecision—only the single study has provided data towards this outcome valuation. This single study only included 10 participants.

^i^ Downgraded two levels for risk of bias—all included studies had extremely serious concerns with risk of bias.

^j^ Downgraded one level for indirectness—one study has combined the responses of adults with ALS with those of caregivers.

^k^ Downgraded two levels for risk of bias—the single contributing study had extremely serious concerns with risk of bias.

^l^ Downgraded one level for indirectness—the single contributing study has combined the responses of adults with ALS with those of caregivers.

^m^ Downgraded two levels for imprecision—the single contributing study has only provided n with no totals.

^n^ Downgraded one level for risk of bias—one of the included studies had extremely serious concerns with risk of bias.

^o^ Downgraded one level for inconsistency—the included studies had inconsistent valuations regarding the outcome of interest which were unexplained.

^p^ Downgraded one level for imprecision—the single contributing study has produced valuations with wide variety of preference.

^q^ Downgraded one level for imprecision—the single study has only provided outcome data from 1 participant at each time point.

^r^ Downgraded two levels for risk of bias—several included studies had extremely serious concerns with risk of bias.

^s^ Downgraded one level for indirectness—the outcome was measured differently across all included studies.

Because utility scores for identical health states vary significantly across populations due to cultural, socio‐demographic, and methodological factors, combining datasets derived from different national preference sets is strongly advised against [66]. Consequently, we deemed it methodologically inappropriate to pool these data to offer a single summary estimate for adults with ALS.

For full transparency, an exploratory meta‐analysis of these two studies was performed. As anticipated, extreme heterogeneity was observed (*Q* = 99.64, *p* < .001, *I* ^2^ = 99%), driven by the divergent preference sets. Because a pooled estimator in this context lacks clinical usefulness and could be misleading, the combined mean valuation and its corresponding forest plot have been presented in Supporting Information S1: Material 6, but will not be discussed further.

#### 3.4.3 Health‐Related Quality for Adults Diagnosed With ALS at Different Stage of Disease Progression (King's)

**Figure 2:**
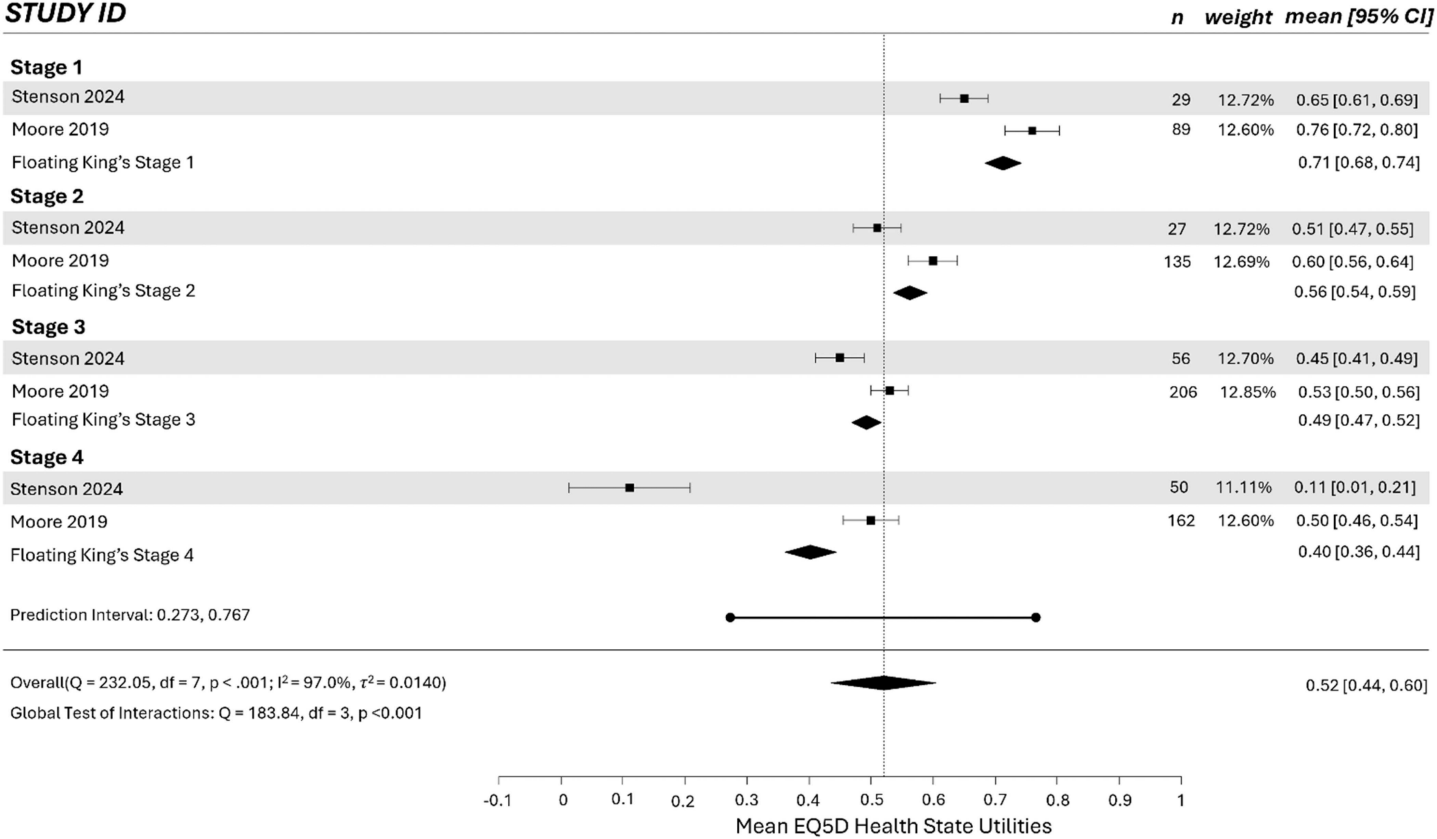
Forest‐plot detailing the meta‐analysis (DerSimonian‐Laird, random‐effects model, within‐trial interaction framework) of mean EQ. 5D health state valuation for adults diagnosed with ALS sub‐grouped according to disease progression using the King's staging system.

Two studies utilising a UK preference set provided valuations according to Kin's staging [44, 55]. Pooled hrQoL declined as disease progressed (Figure 2); stage 1 (0.70, 95%CI: 0.60–0.81), stage 2 (0.55, 95%CI: 0.47–0.64), stage 3 (0.49, 95%CI: 0.41–0.57) and stage 4 (0.31, 95%CI: −0.07–0.69). A global test of interaction confirmed that disease progression was a significant modifier of hrQoL (*Q* = 183.84, df = 3, *p* < 0.001, Figure 2). While the overall mean was 0.52 (95%CI: 0.52–0.6), this requires cautious interpretation due to the extreme heterogeneity (*Q* = 232.05, *p* < .0.001, *I* ^2^ = 97%, *τ* ^2^ = 0.0140). Sensitivity analysis (Supporting Information S1: Material 8) using a fixed‐effect model produced a result with narrower confidence intervals, however these may be artificially precise given the underlying heterogeneity. There was no other important difference between the two models.

#### 3.4.4 Health‐Related Quality for Adults Diagnosed With ALS at Different Stage of Disease Progression (MiToS)

**Figure 3:**
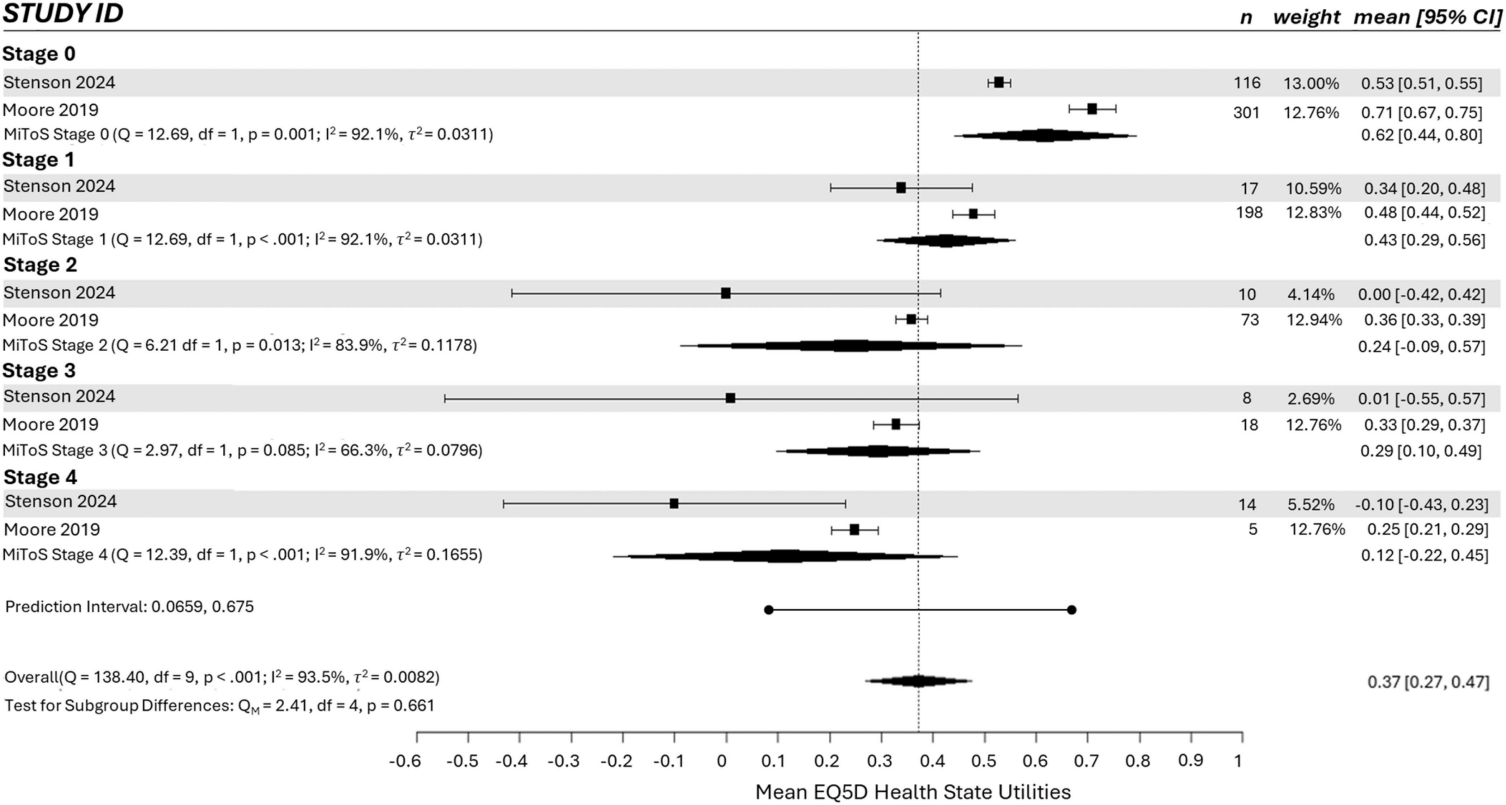
Forest‐plot detailing the meta‐analysis (DerSimonian‐Laird, random‐effects model) of mean EQ. 5D health state valuation for adults diagnosed with ALS sub‐grouped according to disease progression using the MiToS staging system.

The same two studies [44, 55] provided valuations staged with the MiToS system (Figure 3). The pooled mean hrQoL was 0.37 (95%CI: 0.27 to 0.47) though extreme heterogeneity was observed (*Q* = 138.4, *p* < 0.001, *I* ^2^ = 93.5%, *τ* ^2^ = 0.0082) and as with the above, this overall value should be interpreted with caution. For stage 0 the mean hrQoL was 0.62 (95%CI: 0.44–0.80), stage 1 (0.43, 95%CI: 0.29–0.56), stage 2 (0.24, 95%CI: −0.09–0.57), stage 3 (0.29, 95%CI: 0.10–0.49) and stage 4 (0.12, 95%CI%: −0.22–0.45). While a downward trend as disease severity increased was apparent, the global test of interaction was not statistically significant (*Q*_M_ = 2.41, *p* = 0.661) (Figure 3). As with the above, a fixed‐effect model (Supporting Information S1: Material 8) produced a result with narrower confidence intervals; however, these may be artificially precise given the underlying heterogeneity. There was no other important difference between the two models.

#### 3.4.5 Health‐Related Quality for Adults Diagnosed With ALS at Different Stage of Disease Progression (ALS Health State Scale (ALS HSS) and Functional Test 9 (FT9))

Health‐related quality of life was further evaluated by the single study (each) using the ALS HSS [41] and the FT9 staging systems [64]. For ALS HSS, stage‐specific valuations ranged from 0.74 (level 1) to 0.37 (level 4), with an overall mean of 0.56 (95%CI: 0.41–0.71). Data for FT9 showed a similar decline from 0.79 (FT9 0) to 0.46 (FT9 4), with an overall mean of 0.63 (95%CI: 0.53–0.74). While formal tests for subgroup differences were not calculable due to the limited data points, both systems demonstrated clear association between disease progression and reduced utility. High heterogeneity was observed in both analyses (*I* ^2^ > 92%). Detailed forest plots for both analyses have been provided as Supporting Information S1: Material 6. Sensitivity analyses using the fixed‐effect model did not yield any important differences (Supporting Information S1: Material 8).

#### 3.4.6 Narrative Syntheses

Beyond hrQoL, 108 unique health‐state valuations, 108 unique health‐state valuations were extracted. Extreme heterogeneity precluded formal meta‐analysis for these outcomes. Consequently, findings were synthesised narratively across 14 different domains. Detailed narrative syntheses, including domain and subdomain breakdowns, study contributions, and interpretive findings are provided as Supporting Information S1: Material 7.

## 4 Discussion

This systematic review has synthesised the values and preferences related to outcomes that matter most to people living with MND and their caregivers. While several outcomes were supported by high certainty evidence (Table 1), this was often due to the data coming from one study that was at low risk of bias and that had reported precise estimates. There currently exists high certainty evidence to indicate:

- Adults with ALS from the UK may have a mean valuation of their health‐related quality of life of 0.57, however this may change depending on the staging system utilised and the stage of disease progression.
- Adults with ALS and their caregivers have no strong preferences regarding their access to emotional support.
- Adults with ALS have strong preferences for interventions that support their ability to eat.
- Adults with ALS have a slight preference for interventions that support their body weight, mucus and bowel/bladder function.
- Adults with ALS prefer implanted drug delivery devices (IDDD) over lumbar puncture for intrathecal therapy.
- Female adults with ALS prefer voice banking, while males do not.
- Adults with ALS have no strong preferences regarding the availability of phone advice, dependence on healthcare providers, or hospice timing.
- Adults with ALS in earlier disease stages prefer to receive disease‐related information at the time of diagnosis.

There was moderate certainty evidence to indicate:

- Adults with ALS and their caregivers prefer interventions that improve their general symptoms and sleep quality.
- Adults with ALS have no strong preference regarding how their personal care is delivered.

There was low certainty evidence to indicate:

- Clinicians serving as proxy to adults living with may have a mean valuation of living with ALS with an Appel score of 0.89 ± 0.04 (Appel Score: 40–59); 0.82 ± 0.04 (Appel Score: 60–86); 0.41 ± 0.17 (Appel Score: 87–109); 0.01 ± 0.20 (Appel Score: 110–128); −0.53 ± 0.14 (Appel Score: 129–164).
- Adults with ALS from China may have a mean valuation of their health‐related quality of life of 0.72; however, this may change depending on the staging system utilised and the stage of disease progression.
- Adults with ALS may prefer interventions that address cognitive function, mental health, anxiety, financial burden, flavour perception, retention of taste, day to day living, pain, fatigue, sialorrhea
- Adults with ALS and PLS may slightly prefer interventions that support their ability to travel.
- Adults with ALS may prefer access to genetic testing.

Several outcomes were informed by very low certainty evidence, and it is difficult to draw firm conclusion from these.

EQ‐5D utility scores were highly stage‐dependent, declining from ~0.7 in early stages to ~0.12 in late stages (Figures 2 and 3, Supporting Information S1: Material 6). This trend provides face validity for using generic utility measures to track MND progression, though certainty was mostly low or very low (Table 1). Variability across stages likely stems from biologically plausible heterogeneity (*I* ^2^ > 90%), limited statistical power within subgroups, and the reduced sensitivity of generic instruments to MND‐specific changes.

### 4.1 Strengths and Limitations

This review is the most comprehensive quantitative synthesis of MND values and preferences to date. Strengths include the application of rigorous systematic review methodology, including GRADE methods, use of ROBVALU for bias assessment, and contemporary meta‐analytic techniques for synthesis of utility data.

However, the review is not without limitations. The underlying evidence base remains methodologically heterogeneous, with studies using different elicitation and valuation approaches and investigating different populations. Although the evidence is largely derived from ALS cohorts, ALS represents the most common form of MND; given the frequent interchangeability of these terms, the findings can reasonably be considered representative of the broader MND community. Additionally, although some studies reported on large sample sizes, many had small cohorts, often recruited from single centres that may reflect preferences and values of the country or time period in which the study was conducted, and the appropriateness of combining these studies together may be debated. Furthermore, while our search was comprehensive and exhaustive with broad eligibility criteria, it was limited in its consideration of only peer‐reviewed literature for inclusion. It remains possible that relevant grey literature or unpublished studies were not captured, which may increase the potential of publication bias.

A further limitation is the lack of integration of qualitative data with the quantitative findings. We acknowledge that the inclusion of qualitative data could provide important contextual insights into the motivations, reasoning and lived experiences underlying patient preferences, which is why qualitative studies were originally in scope of this review. By not integrating this data as stipulated in the *a priori* protocol we may have limited our understanding as to *why* some outcomes were prioritised. As discussed above, the overwhelming number of qualitative studies identified suggested that these studies should be considered as part of their own review at a later date. The findings of this second review can then be juxtaposed with the findings of this review as part of the ongoing development of the Australian MND Guideline.

One additional rapid review [67] was identified outside of our formal search strategy. While its inclusion did not alter the review's overall conclusions, these findings indicate a potential limitation of our search strategy. We acknowledge that our search may not have captured all relevant literature, though we believe this to be an isolated case.

Finally, although the review includes the values and preferences of patients, caregivers, and clinicians acting as proxies, the available data did not permit a formal comparison of convergence or divergence between these groups.

Despite these limitations, this review provides a comprehensive synthesis of the currently available evidence regarding MND values and preferences. While the certainty of this evidence is oftentimes low or very low, these findings will directly inform outcome prioritisation for the Australian MND guideline, future research, and clinical decision‐making.

## 5 Conclusion

The findings of our review have direct implications for the development of the Australian MND Guideline. The prioritised outcomes identified from this work can assist in the selection of outcomes prioritised as part of the guideline, and in terms of outcome importance following the GRADE approach. The clear stage‐dependent decline in quality of life across advancing disease stages highlights that stage‐specific utility estimates, rather than overall averages, are essential for this process. Future research should prioritise standardisation of methods for eliciting preferences in MND, the establishment of a core‐outcome set that may inform future MND research, and further exploration into patient preferences regarding specific treatment modalities.

## Author Contributions

**Timothy Hugh Barker:** conceptualization, methodology, software, data curation, investigation, validation, formal analysis, funding acquisition, visualization, project administration, resources, writing – original draft, writing – review and editing. **Lemma N. Bulto:** methodology, software, formal analysis. **Grace Holland:** conceptualization, methodology, software, formal analysis, funding acquisition, writing – original draft, writing – review and editing. **Camille Schubert:** conceptualization, methodology, funding acquisition, writing – original draft, writing – review and editing. **Tracy Merlin:** conceptualization, methodology, funding acquisition, writing – original draft, writing – review and editing. **Samer G. Karam:** methodology, writing – original draft, writing – review and editing. **Wojtek Wiercioch:** methodology, writing – original draft, writing – review and editing. **Danielle Pollock:** conceptualization, methodology, funding acquisition, writing – original draft, writing – review and editing. **Cindy Stern:** conceptualization, methodology, funding acquisition, writing – original draft, writing – review and editing. **Jay Beasley‐Hall:** methodology, funding acquisition, writing – original draft, writing – review and editing. **Nipun Shrestha:** methodology. **Steve Vucic:** conceptualization, methodology, writing – original draft, writing – review and editing. **Lynne Giles:** conceptualization, methodology, funding acquisition, writing – original draft, writing – review and editing. **Zachary Munn:** conceptualization, methodology, investigation, validation, supervision, funding acquisition, visualization, project administration, writing – original draft, writing – review and editing.

## Conflicts of Interest

Lemma Negesa Bulto, Grace Holland, Danielle Pollock, Cindy Stern and Nipun Shrestha are members of the Adelaide GRADE Centre. Samer Karam is a member of the McMaster GRADE Centre. Timothy Hugh Barker is Deputy Director of the Adelaide GRADE Centre. Wojtek Wiercioch is Director of the McMaster GRADE Centre. Zachary Munn is Director of the Adelaide GRADE Centre.

## Supporting information


Supporting File

## Data Availability

All data for this review, and for the entire Australian MND Guideline is available on the Open Science Framework (OSF) at https://osf.io/c2ezm/.
