# Corrigendum to “The Values and Preferences of People Living With Motor Neurone Disease (MND): A Systematic Review and Meta‐Analysis”

**DOI:** 10.1002/gin2.70090

**Published:** 2026-10-08

T. H. Barker, L. N. Bulto, G. Holland, et al., “The Values and Preferences of People Living With Motor Neurone Disease (MND): A Systematic Review and Meta‐Analysis,” Clinical and Public Health Guidelines 3 (2026): e70080.https://doi.org/10.1002/gin2.70080.

**The second line of the paragraph of Section 2.6 Data Synthesis states:** We used DerSimonian‐Laird random‐effects models when four or more studies contributed to a meta‐analysis, and inverse variance fixed‐effect models when three of fewer studies contributed, due to unreliable variance estimation [23, 40].

**The correct sentence should be:** We used DerSimonian‐Laird random‐effects models when four or more effect estimates contributed to a meta‐analysis, and inverse variance fixed‐effect models when three or fewer effect estimates contributed, due to unreliable variance estimation [23, 40].

**The paragraph of Section 3.4.3 Health‐Related Quality for Adults Diagnosed With ALS at Different Stage of Disease Progression (King's) states:** Two studies utilising a UK preference set provided valuations according to Kin's staging [44, 55]. Pooled hrQoL declined as disease progressed (Figure 2); stage 1 (0.70, 95%CI: 0.60–0.81), stage 2 (0.55, 95%CI: 0.47–0.64), stage 3 (0.49, 95%CI: 0.41–0.57) and stage 4 (0.31, 95%CI: −0.07–0.69). … While the overall mean was 0.52 (95%CI: 0.52–0.6), this requires cautious interpretation due to the extreme heterogeneity …

**The correct text should be:** Two studies utilising a UK preference set provided valuations according to King's staging [44, 55]. Pooled hrQoL declined as disease progressed (Figure 2); stage 1 (0.71, 95%CI: 0.68–0.74), stage 2 (0.56, 95%CI: 0.54–0.59), stage 3 (0.49, 95%CI: 0.47–0.52) and stage 4 (0.40, 95%CI: 0.36–0.44). … While the overall mean was 0.52 (95%CI: 0.44–0.60), this requires cautious interpretation due to the extreme heterogeneity …

Neither error affects the data extracted, the analyses conducted, the certainty of evidence assessments, or the conclusions of the review.

We apologize for this error.

