# Supplementary material for: The Values and Preferences of People Living With Motor Neurone Disease (MND): A Systematic Review and Meta‐Analysis
Source: Clin Public Health Guidel. 2026 Jul 15;3(3):e70080. doi: 10.1002/gin2.70080 (PMC13563401; doi:10.1002/gin2.70080)
Supplement: Supplementary file 1 [file GIN2-3-e70080-s001.docx]

**SUPPLEMENTARY MATERIAL**

**Supplementary material 1 - PRISMA Checklist**

**Supplementary table 1 –** PRISMA Checklist

| **Section and Topic** | **Item #** | **Checklist item** | **Location where item is reported** |
| --- | --- | --- | --- |
| **TITLE** | | |  |
| Title | 1 | Identify the report as a systematic review. | Title |
| **ABSTRACT** | | |  |
| Abstract | 2 | See the PRISMA 2020 for Abstracts checklist. | Page 2 |
| **INTRODUCTION** | | |  |
| Rationale | 3 | Describe the rationale for the review in the context of existing knowledge. | Background |
| Objectives | 4 | Provide an explicit statement of the objective(s) or question(s) the review addresses. | Background |
| **METHODS** | | |  |
| Eligibility criteria | 5 | Specify the inclusion and exclusion criteria for the review and how studies were grouped for the syntheses. | Methods, eligibility criteria |
| Information sources | 6 | Specify all databases, registers, websites, organisations, reference lists and other sources searched or consulted to identify studies. Specify the date when each source was last searched or consulted. | Methods, search strategy |
| Search strategy | 7 | Present the full search strategies for all databases, registers and websites, including any filters and limits used. | Appendix 2 |
| Selection process | 8 | Specify the methods used to decide whether a study met the inclusion criteria of the review, including how many reviewers screened each record and each report retrieved, whether they worked independently, and if applicable, details of automation tools used in the process. | Methods, study selection and screening |
| Data collection process | 9 | Specify the methods used to collect data from reports, including how many reviewers collected data from each report, whether they worked independently, any processes for obtaining or confirming data from study investigators, and if applicable, details of automation tools used in the process. | Methods, data extraction |
| Data items | 10a | List and define all outcomes for which data were sought. Specify whether all results that were compatible with each outcome domain in each study were sought (e.g. for all measures, time points, analyses), and if not, the methods used to decide which results to collect. | Methods, outcomes |
|  | 10b | List and define all other variables for which data were sought (e.g. participant and intervention characteristics, funding sources). Describe any assumptions made about any missing or unclear information. | Methods, data extraction |
| Study risk of bias assessment | 11 | Specify the methods used to assess risk of bias in the included studies, including details of the tool(s) used, how many reviewers assessed each study and whether they worked independently, and if applicable, details of automation tools used in the process. | Methods, assessment of risk of bias |
| Effect measures | 12 | Specify for each outcome the effect measure(s) (e.g. risk ratio, mean difference) used in the synthesis or presentation of results. | Methods, data synthesis |
| Synthesis methods | 13a | Describe the processes used to decide which studies were eligible for each synthesis (e.g. tabulating the study intervention characteristics and comparing against the planned groups for each synthesis (item #5)). | Methods, data synthesis |
|  | 13b | Describe any methods required to prepare the data for presentation or synthesis, such as handling of missing summary statistics, or data conversions. | Methods, data synthesis |
|  | 13c | Describe any methods used to tabulate or visually display results of individual studies and syntheses. | Methods, data synthesis |
|  | 13d | Describe any methods used to synthesize results and provide a rationale for the choice(s). If meta-analysis was performed, describe the model(s), method(s) to identify the presence and extent of statistical heterogeneity, and software package(s) used. | Methods, data synthesis |
|  | 13e | Describe any methods used to explore possible causes of heterogeneity among study results (e.g. subgroup analysis, meta-regression). | Methods, data synthesis |
|  | 13f | Describe any sensitivity analyses conducted to assess robustness of the synthesized results. | Methods, data synthesis |
| Reporting bias assessment | 14 | Describe any methods used to assess risk of bias due to missing results in a synthesis (arising from reporting biases). | Methods, data synthesis |
| Certainty assessment | 15 | Describe any methods used to assess certainty (or confidence) in the body of evidence for an outcome. | Methods, assessment of certainty of evidence |
| **RESULTS** | | |  |
| Study selection | 16a | Describe the results of the search and selection process, from the number of records identified in the search to the number of studies included in the review, ideally using a flow diagram. | Results, results of search |
|  | 16b | Cite studies that might appear to meet the inclusion criteria, but which were excluded, and explain why they were excluded. | Results, results of search |
| Study characteristics | 17 | Cite each included study and present its characteristics. | Results, characteristics of the included studies |
| Risk of bias in studies | 18 | Present assessments of risk of bias for each included study. | Results, assessment of risk of bias |
| Results of individual studies | 19 | For all outcomes, present, for each study: (a) summary statistics for each group (where appropriate) and (b) an effect estimate and its precision (e.g. confidence/credible interval), ideally using structured tables or plots. | Results, meta-analysis |
| Results of syntheses | 20a | For each synthesis, briefly summarise the characteristics and risk of bias among contributing studies. | Results, meta-analysis |
|  | 20b | Present results of all statistical syntheses conducted. If meta-analysis was done, present for each the summary estimate and its precision (e.g. confidence/credible interval) and measures of statistical heterogeneity. If comparing groups, describe the direction of the effect. | Results, meta-analysis |
|  | 20c | Present results of all investigations of possible causes of heterogeneity among study results. | Results, meta-analysis |
|  | 20d | Present results of all sensitivity analyses conducted to assess the robustness of the synthesized results. | Appendix 6 |
| Reporting biases | 21 | Present assessments of risk of bias due to missing results (arising from reporting biases) for each synthesis assessed. | Not conducted |
| Certainty of evidence | 22 | Present assessments of certainty (or confidence) in the body of evidence for each outcome assessed. | Summary of findings |
| **DISCUSSION** | | |  |
| Discussion | 23a | Provide a general interpretation of the results in the context of other evidence. | Discussion |
|  | 23b | Discuss any limitations of the evidence included in the review. | Discussion, strengths and limitations |
|  | 23c | Discuss any limitations of the review processes used. | Discussion, strengths and limitations |
|  | 23d | Discuss implications of the results for practice, policy, and future research. | Discussion, strengths and limitations |
| **OTHER INFORMATION** | | |  |
| Registration and protocol | 24a | Provide registration information for the review, including register name and registration number, or state that the review was not registered. | Methods (introductory text) |
|  | 24b | Indicate where the review protocol can be accessed, or state that a protocol was not prepared. | Methods (introductory text) |
|  | 24c | Describe and explain any amendments to information provided at registration or in the protocol. | Methods, deviations from protocol |
| Support | 25 | Describe sources of financial or non-financial support for the review, and the role of the funders or sponsors in the review. | Title page |
| Competing interests | 26 | Declare any competing interests of review authors. | Title page |
| Availability of data, code and other materials | 27 | Report which of the following are publicly available and where they can be found template data collection forms; data extracted from included studies; data used for all analyses; analytic code; any other materials used in the review. | Title page |

**Supplementary material 2 - Search strategy**

**Medline (Ovid) (26/02/2025)**

| 1 - MND | Exp motor neuron disease OR Spastic Paraplegia, hereditary.sh OR (Gehrig* disease OR motor neuron* disease* OR progressive bulbar pals* OR progressive muscular atroph* OR spinal muscular atroph* OR hereditary spastic parapares* OR "spinal and bulbar muscular atroph*" OR Kennedy* disease* OR Kennedy* syndrome* OR bulbo-spinal atroph* or flail arm ALS OR flail leg ALS or flail arm syndrome OR anterior horn cell disease* OR charcot disease* OR guam disease* OR bulbar pals* OR fazio-londe* disease* OR fazio-londe* syndrome* OR spinal amyotroph* OR spinal muscular atroph* OR bulbospinal neuronopath* OR hereditary motor neuronopath* OR infantile muscular atroph* OR juvenile muscular atroph* OR infantile acute form SMA OR werdnig hoffman* disease* OR motoneuron disease* OR motor system disease* OR ALS Dementia OR chinese paralytic syndrome* OR Aran Duchenne OR Duchennen Aran OR hereditary spastic paraplegia* OR hereditary autosomal dominant spastic paraplegia* OR hereditary autosomal recessive spastic paraplegia* OR "hereditary motor and sensory neuropath*" OR hereditary x linked recessive spastic paraplegia* OR hypertrophic motor sensory neuropathy spastic paraplegia* OR spastic paraplegia type 2 OR Charcot Marie OR Charcot Hoffman OR hereditary motor sensory neuropath* OR Dejerine sottas OR familial spastic para* OR "hereditary motor and sensory neuropath*" OR hereditary sensorimotor neuropath* OR "hereditary sensory and motor neuropath*" OR neurogenic amyotroph* OR neurogenic atroph* OR neurogenic muscle atroph* OR neurogenic muscle syndrome* OR peroneal atroph* OR perone* muscle atroph* OR perone* muscular atroph* OR spastic hereditary paraglegia OR Struemell* disease* OR Struempell Lorrain OR Strumpel* disease* OR spinal amyotroph* OR bulbospinal neuronopath* OR distal spinal muscular atroph* OR hereditary motor neuronaopath* OR myelopathic muscular atroph* OR Spinobulbar muscular atroph* OR bulbo-spinal atroph* OR MND).ti,ab,kw OR (lateral scleros*).ti,ab,kf | 63441 |
| --- | --- | --- |
| 2 - Values & Preferences | Patient preference.sh OR exp attitude to health OR attitude to death.sh OR exp attitude of health personnel OR ((exp patients OR caregivers.sh OR exp family) AND exp decision making) OR ((Patient* OR famil* OR carer* OR caregiver* OR care-giver* OR caretaker* OR care-taker* OR clinician* OR spouse* OR parent* OR carrier* OR doctor* OR specialist* OR health-care worker* OR healthcare worker* OR health-care professional* OR healthcare professional* OR provider* OR plwMND OR pwMND OR "people living with") adj6 (values OR valued OR prefer* OR desir* OR priorit* OR expectation* OR perspective* OR perceive* OR percept* OR view* OR attitude* OR accept* OR knowledge OR decision* or decid* OR choice* OR satisf* OR sentiment* OR motiv* OR approach*)).ti,ab,kw OR (patient-reported experience measure* OR patient-reported outcome measure* OR ALSAQ-40 OR EQ-5D OR Short Form Health Survey OR SF-36 OR Health Utilities Index OR HUI OR "Quality of Wellbeing" OR QWB).ti,ab,kw | 1433848 |
|  | 1 AND 2 | 2671 |

**Embase (Ovid) (26/02/2025)**

| 1 - MND | Exp motor neuron disease OR exp hereditary motor sensory neuropathy OR (Gehrig* disease OR motor neuron* disease* OR progressive bulbar pals* OR progressive muscular atroph* OR spinal muscular atroph* OR hereditary spastic parapares* OR "spinal and bulbar muscular atroph*" OR Kennedy* disease* OR Kennedy* syndrome* OR bulbo-spinal atroph* or flail arm ALS OR flail leg ALS or flail arm syndrome OR anterior horn cell disease* OR charcot disease* OR guam disease* OR bulbar pals* OR fazio-londe* disease* OR fazio-londe* syndrome* OR spinal amyotroph* OR spinal muscular atroph* OR bulbospinal neuronopath* OR hereditary motor neuronopath* OR infantile muscular atroph* OR juvenile muscular atroph* OR infantile acute form SMA OR werdnig hoffman* disease* OR motoneuron disease* OR motor system disease* OR ALS Dementia OR chinese paralytic syndrome* OR Aran Duchenne OR Duchennen Aran OR hereditary spastic paraplegia* OR hereditary autosomal dominant spastic paraplegia* OR hereditary autosomal recessive spastic paraplegia* OR "hereditary motor and sensory neuropath*" OR hereditary x linked recessive spastic paraplegia* OR hypertrophic motor sensory neuropathy spastic paraplegia* OR spastic paraplegia type 2 OR Charcot Marie OR Charcot Hoffman OR hereditary motor sensory neuropath* OR Dejerine sottas OR familial spastic para* OR "hereditary motor and sensory neuropath*" OR hereditary sensorimotor neuropath* OR "hereditary sensory and motor neuropath*" OR neurogenic amyotroph* OR neurogenic atroph* OR neurogenic muscle atroph* OR neurogenic muscle syndrome* OR peroneal atroph* OR perone* muscle atroph* OR perone* muscular atroph* OR spastic hereditary paraglegia OR Struemell* disease* OR Struempell Lorrain OR Strumpel* disease* OR spinal amyotroph* OR bulbospinal neuronopath* OR distal spinal muscular atroph* OR hereditary motor neuronaopath* OR myelopathic muscular atroph* OR Spinobulbar muscular atroph* OR bulbo-spinal atroph* OR MND).ti,ab,kw OR (lateral scleros*).ti,ab,kf | 100420 |
| --- | --- | --- |
| 2 - Values & Preferences | Exp patient attitude OR attitude to death.sh OR (decision making.sh AND (exp patient OR exp carer OR exp family)) OR ((Patient* OR famil* OR carer* OR caregiver* OR care-giver* OR caretaker* OR care-taker* OR clinician* OR spouse* OR parent* OR carrier* OR doctor* OR specialist* OR health-care worker* OR healthcare worker* OR health-care professional* OR healthcare professional* OR provider* OR plwMND OR pwMND OR "people living with") adj4 (values OR valued OR prefer* OR desir* OR priorit* OR expectation* OR perspective* OR perceive* OR percept* OR view* OR attitude* OR accept* OR knowledge OR decision* or decid* OR choice* OR satisf* OR sentiment* OR motiv* OR approach*)).ti,ab,kw OR (patient-reported experience measure* OR PREM OR patient-reported outcome measure* OR ALSAQ-40 OR EQ-5D OR Short Form Health Survey OR SF-36 OR Health Utilities Index OR HUI OR "Quality of Wellbeing" OR QWB).ti,ab,kw | 1484217 |
|  | 1 AND 2 | 4110 |

**Cochrane Library (CENTRAL) (26/02/2025)**

| 1 - MND | [mh "motor neuron disease"] OR [mh "Spastic Paraplegia, hereditary"] OR ((Gehrig* NEXT disease) OR "motor neuron disease" OR "motor neurone disease" OR (lateral NEXT scleros*) OR ("progressive bulbar" NEXT pals*) OR ("progressive muscular" NEXT atroph*) OR ("spinal muscular" NEXT atroph*) OR ("hereditary spastic" NEXT parapares*) OR ("spinal and bulbar muscular" NEXT atroph*) OR (Kennedy* NEXT disease*) OR (Kennedy* NEXT syndrome*) OR "bulbo-spinal atrophy" OR"flail arm ALS" OR "flail leg ALS" or "flail arm syndrome" OR ("anterior horn cell" NEXT disease*) OR (charcot NEXT disease*) OR (guam NEXT disease*) OR (bulbar NEXT pals*) OR ("fazio-londe" NEXT disease*) OR ("fazio-londe" NEXT syndrome*) OR (spinal NEXT amyotroph*) OR ("spinal muscular" NEXT atroph*) OR (bulbospinal NEXT neuronopath*) OR ("hereditary motor" NEXT neuronopath*) OR ("infantile muscular" NEXT atroph*) OR ("juvenile muscular" NEXT atroph*) OR "infantile acute form SMA" OR (werdnig NEXT hoffman* NEXT disease*) OR (motoneuron NEXT disease*) OR ("motor system" NEXT disease*) OR "ALS Dementia" OR ("chinese paralytic" NEXT syndrome*) OR "Aran Duchenne" OR "Duchennen Aran" OR ("hereditary spastic" NEXT paraplegia*) OR ("hereditary autosomal dominant spastic" NEXT paraplegia*) OR ("hereditary autosomal recessive spastic" NEXT paraplegia*) OR ("hereditary motor and sensory" NEXT neuropath*) OR ("hereditary x linked recessive spastic" NEXT paraplegia*) OR ("hypertrophic motor sensory neuropathy spastic" NEXT paraplegia*) OR "spastic paraplegia type 2" OR "Charcot Marie" OR "Charcot Hoffman" OR ("hereditary motor sensory" NEXT neuropath*) OR "Dejerine sottas" OR ("familial spastic" NEXT para*) OR ("hereditary motor and sensory" NEXT neuropath*) OR ("hereditary sensorimotor" NEXT neuropath*) OR ("hereditary sensory and motor" NEXT neuropath*) OR (neurogenic NEXT amyotroph*) OR (neurogenic NEXT atroph*) OR ("neurogenic muscle" NEXT atroph*) OR ("neurogenic muscle" NEXT syndrome*) OR (peroneal NEXT atroph*) OR (perone* NEXT muscle NEXT atroph*) OR (perone* NEXT muscular NEXT atroph*) OR "spastic hereditary paraglegia" OR (Struemell* NEXT disease*) OR "Struempell Lorrain" OR (Strumpel* NEXT disease*) OR (spinal NEXT amyotroph*) OR (bulbospinal NEXT neuronopath*) OR ("distal spinal muscular" NEXT atroph*) OR ("hereditary motor" NEXT neuronaopath*) OR ("myelopathic muscular" NEXT atroph*) OR ("Spinobulbar muscular" NEXT atroph*) OR ("bulbo-spinal" NEXT atroph*) OR MND):ti,ab,kw | 1175 |
| --- | --- | --- |
| 2 – Values, preferences & QoL indicators | [mh "Patient preference"] OR [mh "attitude to health"] OR [mh "attitude to death"] OR [mh "attitude of health personnel"] OR (([mh patients] OR [mh caregivers] OR [mh family]) AND [mh "decision making"]) OR ((Patient* OR famil* OR carer* OR caregiver* OR care-giver* OR caretaker* OR care-taker* OR clinician* OR spouse* OR parent* OR carrier* OR doctor* OR specialist* OR ("health-care" NEXT worker*) OR (healthcare NEXT worker*) OR ("health-care" NEXT professional*) OR (healthcare NEXT professional*) OR provider* OR plwMND OR pwMND OR "people living with") NEAR/6 (values OR valued OR prefer* OR desir* OR priorit* OR expectation* OR perspective* OR perceive* OR percept* OR view* OR attitude* OR accept* OR knowledge OR decision* or decid* OR choice* OR satisf* OR sentiment* OR motiv* OR approach*)):ti,ab,kw OR (("patient-reported experience" NEXT measure*) OR PREM OR ("patient-reported outcome" NEXT measure*) OR ALSAQ-40 OR EQ-5D OR "Short Form Health Survey" OR SF-36 OR "Health Utilities Index" OR HUI OR "Quality of Wellbeing" OR QWB):ti,ab,kw | 192355 |
|  | 1 AND 2 | 112 |

**Clinical Trials (**[**www.clinicaltrials.gov**](http://www.clinicaltrials.gov)**) (26/02/2025)**

| 1 - MND  2 – Values, preferences, & utility measures | ("Gehrig* disease" OR "motor neuron* disease*" OR "progressive bulbar pals*" OR "progressive muscular atroph*" OR "spinal muscular atroph*" OR "hereditary spastic parapares*" OR "spinal and bulbar muscular atroph*" OR "Kennedy* disease*" OR "Kennedy* syndrome*" OR "bulbo-spinal atroph*" or "flail arm ALS" OR "flail leg ALS" or "flail arm syndrome" OR "anterior horn cell disease*" OR "charcot disease*" OR "guam disease*" OR "bulbar pals*" OR "fazio-londe* disease*" OR "fazio-londe* syndrome*" OR "spinal amyotroph*" OR "spinal muscular atroph*" OR "bulbospinal neuronopath*" OR "hereditary motor neuronopath*" OR "infantile muscular atroph*" OR "juvenile muscular atroph*" OR "infantile acute form SMA" OR "werdnig hoffman* disease*" OR "motoneuron disease*" OR "motor system disease*" OR "ALS Dementia" OR "chinese paralytic syndrome*" OR "Aran Duchenne" OR "Duchennen Aran" OR "hereditary spastic paraplegia*" OR "hereditary autosomal dominant spastic paraplegia*" OR "hereditary autosomal recessive spastic paraplegia*" OR "hereditary motor and sensory neuropath*" OR "hereditary x linked recessive spastic paraplegia*" OR "hypertrophic motor sensory neuropathy spastic paraplegia*" OR "spastic paraplegia type 2" OR "Charcot Marie" OR "Charcot Hoffman" OR "hereditary motor sensory neuropath*" OR "Dejerine sottas" OR "familial spastic para*" OR "hereditary motor and sensory neuropath*" OR "hereditary sensorimotor neuropath*" OR "hereditary sensory and motor neuropath*" OR "neurogenic amyotroph*" OR "neurogenic atroph*" OR "neurogenic muscle atroph*" OR "neurogenic muscle syndrome*" OR "peroneal atroph*" OR "perone* muscle atroph*" OR "perone* muscular atroph*" OR "spastic hereditary paraglegia" OR "Struemell* disease*" OR "Struempell Lorrain" OR "Strumpel* disease*" OR "spinal amyotroph*" OR "bulbospinal neuronopath*" OR "distal spinal muscular atroph*" OR "hereditary motor neuronaopath*" OR "myelopathic muscular atroph*" OR "Spinobulbar muscular atroph*" OR "bulbo-spinal atroph*" OR MND OR "lateral scleros*")  AND  (((patient* OR famil* OR carer* OR caregiver* OR "care-giver*" OR caretaker* OR care-taker* OR clinician* OR spouse* OR parent* OR carrier* OR doctor* OR specialist* OR "health-care worker*" OR "healthcare worker*" OR "health-care professional*" OR "healthcare professional*" OR provider*) AND (value* prefer* OR desir* OR priorit* OR expectation* OR perspective* OR perceive* OR percept* OR view* OR attitude* OR accept* OR knowledge OR decision* or decid* OR choice* OR satisf* OR sentiment* OR motiv* OR approach*)) OR ("patient-reported experience measure*" OR PREM OR "patient-reported outcome measure*" OR "ALSAQ-40" OR "EQ-5D" OR "Short Form Health Survey" OR "SF-36" OR "Health Utilities Index" OR HUI OR "Quality of Wellbeing" OR QWB))  = 500 results |
| --- | --- |

**WHO ICTRP (**[**www.who.int.ictrp**](http://www.who.int.ictrp)**) (26/02/2025)**

| 1 - MND  2 – Values, preferences, & utility measures | ("Gehrig* disease" OR "motor neuron* disease*" OR "progressive bulbar pals*" OR "progressive muscular atroph*" OR "spinal muscular atroph*" OR "hereditary spastic parapares*" OR "spinal and bulbar muscular atroph*" OR "Kennedy* disease*" OR "Kennedy* syndrome*" OR "bulbo-spinal atroph*" or "flail arm ALS" OR "flail leg ALS" or "flail arm syndrome" OR "anterior horn cell disease*" OR "charcot disease*" OR "guam disease*" OR "bulbar pals*" OR "fazio-londe* disease*" OR "fazio-londe* syndrome*" OR "spinal amyotroph*" OR "spinal muscular atroph*" OR "bulbospinal neuronopath*" OR "hereditary motor neuronopath*" OR "infantile muscular atroph*" OR "juvenile muscular atroph*" OR "infantile acute form SMA" OR "werdnig hoffman* disease*" OR "motoneuron disease*" OR "motor system disease*" OR "ALS Dementia" OR "chinese paralytic syndrome*" OR "Aran Duchenne" OR "Duchennen Aran" OR "hereditary spastic paraplegia*" OR "hereditary autosomal dominant spastic paraplegia*" OR "hereditary autosomal recessive spastic paraplegia*" OR "hereditary motor and sensory neuropath*" OR "hereditary x linked recessive spastic paraplegia*" OR "hypertrophic motor sensory neuropathy spastic paraplegia*" OR "spastic paraplegia type 2" OR "Charcot Marie" OR "Charcot Hoffman" OR "hereditary motor sensory neuropath*" OR "Dejerine sottas" OR "familial spastic para*" OR "hereditary motor and sensory neuropath*" OR "hereditary sensorimotor neuropath*" OR "hereditary sensory and motor neuropath*" OR "neurogenic amyotroph*" OR "neurogenic atroph*" OR "neurogenic muscle atroph*" OR "neurogenic muscle syndrome*" OR "peroneal atroph*" OR "perone* muscle atroph*" OR "perone* muscular atroph*" OR "spastic hereditary paraglegia" OR "Struemell* disease*" OR "Struempell Lorrain" OR "Strumpel* disease*" OR "spinal amyotroph*" OR "bulbospinal neuronopath*" OR "distal spinal muscular atroph*" OR "hereditary motor neuronaopath*" OR "myelopathic muscular atroph*" OR "Spinobulbar muscular atroph*" OR "bulbo-spinal atroph*" OR MND OR "lateral scleros*")  AND  (((patient* OR famil* OR carer* OR caregiver* OR "care-giver*" OR caretaker* OR care-taker* OR clinician* OR spouse* OR parent* OR carrier* OR doctor* OR specialist* OR "health-care worker*" OR "healthcare worker*" OR "health-care professional*" OR "healthcare professional*" OR provider*) AND (value* prefer* OR desir* OR priorit* OR expectation* OR perspective* OR perceive* OR percept* OR view* OR attitude* OR accept* OR knowledge OR decision* or decid* OR choice* OR satisf* OR sentiment* OR motiv* OR approach*)) OR ("patient-reported experience measure*" OR PREM OR "patient-reported outcome measure*" OR "ALSAQ-40" OR "EQ-5D" OR "Short Form Health Survey" OR "SF-36" OR "Health Utilities Index" OR HUI OR "Quality of Wellbeing" OR QWB))  = 43 results |
| --- | --- |

**Supplementary material 3 - Reports not retrieved and studies excluded following screening at the full-text stage**

| Study | Reason for Exclusion |
| --- | --- |
| LIVING WELL – Dunn, Y. (2019) | Full-text of peer-reviewed source unavailable |
| A joint respiratory and palliative care clinic – Nathoo, N. (2019) | Full-text of peer-reviewed source unavailable |
| ALS clinical trial engagement – Swenson, A. (2021) | Full-text of peer-reviewed source unavailable |
| Family understanding of patient preferences for family involvement in decision-making at the end of life – Kumar, R. (2010) | Full-text of peer-reviewed source unavailable |
| A systematic review of decision making among patients and their family in ALS care – Foley, G. (2017) | Full-text of peer-reviewed source unavailable |
| A survey of healthcare professionals on the measurement of physical functioning in amyotrophic lateral sclerosis (ALS)/motor neurone disease (MND) and attitudes to development of technology based measurement and monitoring solutions – Murray, D. (2019) | Full-text of peer-reviewed source unavailable |
| Amyotrophic lateral sclerosis (ALS) related impact on social communication patient and caregivers perceptions – Neto, L. L. (2018) | Full-text of peer-reviewed source unavailable |
| How much do patients know about basic research? A survey of knowledge and preferences among people living with ALS and other neurological conditions – Boyce, D. (2022) | Full-text of peer-reviewed source unavailable |
| A multidisciplinary approach improves quality of life perception in patients with Amyotrophic Lateral Sclerosis – Lunetta, C. (2009) | Full-text of peer-reviewed source unavailable |
| The 100 collars project – Glennon, L. (2017) | Full-text of peer-reviewed source unavailable |
| End-of-life decisions and advance care directives in motor neurone disease – Corr, B. (2009) | Full-text of peer-reviewed source unavailable |
| A Patient-centered Approach to Palliative Care for an Amyotrophic Lateral Sclerosis (ALS) Patient at End-of-life – Pennington, M. (2018) | Full-text of peer-reviewed source unavailable |
| Impact of Nusinersen treatment on quality of life in adult patients with Spinal Muscular Atrophy – Castanon, F. G. (2023) | Full-text of peer-reviewed source unavailable |
| COMMENCING NOVEL THERAPIES FOR ADULT SMA PATIENTS – Cader, Z. (2022) | Full-text of peer-reviewed source unavailable |
| Cross-sectional validation study of the spastic paraplegia rating scale (SPRS) and SF-36 health questionnaire in hereditary spastic paraplegia – Trummer, B. (2018) | Full-text of peer-reviewed source unavailable |
| The immediate low profile button gastrostomy – Onders, R. (2014) | Full-text of peer-reviewed source unavailable |
| Analysis of the experiences of patients with MND and their carers following the recommendation to use NIV – Baxter, S. (2012) | Full-text of peer-reviewed source unavailable |
| How patients with ALS, making advanced decisions, can influence outcomes in where their end of life care is received – Callagher, P. (2009) | Full-text of peer-reviewed source unavailable |
| ALS/MND patients prefer low profile gastrostomy tubes – Onders, R. (2016) | Full-text of peer-reviewed source unavailable |
| Carers' perspectives of multidisciplinary home-based e-health for amyotrophic lateral sclerosis – James, N. (2018) | Full-text of peer-reviewed source unavailable |
| A randomised controlled trial on the effect of case management on quality of care, quality of life and caregiver burden in ALS – Creemers, H. (2011) | Full-text of peer-reviewed source unavailable |
| Care preferences in amyotrophic lateral sclerosis (ALS) patients and carers using a discrete choice experiment – Maguire, S. (2016) | Full-text of peer-reviewed source unavailable |
| A pilot study of voice banking in amyotrophic lateral sclerosis patients – Donvito, G. (2018) | Full-text of peer-reviewed source unavailable |
| Home initiation of non-invasive ventilation for motor neurone disease – Johnson, O. (2009) | Full-text of peer-reviewed source unavailable |
| Advance care planning in a dutch tertiary ALS centre, an evaluation of a dutch care approach – Seeber, A. (2014) | Full-text of peer-reviewed source unavailable |
| Amyotrophic lateral sclerosis patients' self-reported satisfaction with assistive technology – Gruis, K. (2010) | Full-text of peer-reviewed source unavailable |
| PRO88 PAYER AND PHYSICIAN PERSPECTIVES OF CURRENT AND EMERGING SPINAL MUSCULAR ATROPHY TREATMENTS IN THE EU5 – Privolnev, Y. (2019) | Full-text of peer-reviewed source unavailable |
| An assessment of the ALSFRS-R by the ALS community – Boyce, D. (2021) | Full-text of peer-reviewed source unavailable |
| When the timing is right – Yogendran, L. (2020) | Full-text of peer-reviewed source unavailable |
| End-of-life decision making in 42 patients with amyotrophic lateral sclerosis – Munroe, C. A. (2007) | Full-text of peer-reviewed source unavailable |
| Longitudinal analysis of a discrete choice experiment of preferences for care of amyotrophic lateral sclerosis patients and their carers – Tobin, K. (2016) | Full-text of peer-reviewed source unavailable |
| Breaking the news in amyotrophic lateral sclerosis. ALS patients' reflections on the two-tiered approach of the ALS centre Amsterdam – Seeber, A. (2013) | Full-text of peer-reviewed source unavailable |
| Computer accessibility – Feldman, S. (2011) | Full-text of peer-reviewed source unavailable |
| Identifying Important Palliative Topics and Communication Preferences Among ALS Patients (QI618) – Yogendran, L. (2020) | Full-text of peer-reviewed source unavailable |
| Genetic testing for familial motor neurone disease (MND) – Crook, A. (2019) | Full-text of peer-reviewed source unavailable |
| Quality of Life (QOL) of Egyptian Patients with Amyotrophic Lateral Sclerosis (ALS) during the Pandemic of COVID-19 – Allam, K. A. E. (2024) | Full-text of peer-reviewed source unavailable |
| [Amyotrophic lateral sclerosis, a total ethical challenge] – Hirsch, Emmanuel (2008) | Full-text of peer-reviewed source unavailable |
| Patient activity of daily living for amyotrophic lateral sclerosis – Statland, J. (2016) | Full-text of peer-reviewed source unavailable |
| ALS patient perspectives on physician assisted death after recent passage of end of life options act – Heinl, N. (2018) | Full-text of peer-reviewed source unavailable |
| Discrete choice experiment for preferences of care in motor neurone patients and their carers – Maguire, S. (2014) | Full-text of peer-reviewed source unavailable |
| A descriptive study of the level of satisfaction with assistive technology devices for patients with amyotrophic lateral sclerosis – Allegretti, A. (2016) | Full-text of peer-reviewed source unavailable |
| Evaluation of a health state staging system defined by loss of independence in amyotrophic lateral sclerosis – Hammond, E. (2013) | Full-text of peer-reviewed source unavailable |
| SUPPORTING PATIENT PREFERENCE FOR LOCATION OF ELECTIVE WITHDRAWAL FROM NON-INVASIVE VENTILATION IN MOTOR NEURONE DISEASE – Cox, G. (2023) | Full-text of peer-reviewed source unavailable |
| HSD5 Patient and Caregiver Spinal Muscular Atrophy (SMA) Treatment Attribute Preferences in Latin America – Saenz, V. (2023) | Full-text of peer-reviewed source unavailable |
| Patient reported experience with multimodal peripheral electrophysiological studies in children and adults with Spinal Muscular Atrophy (SMA) – Ros, L. A. A. (2022) | Full-text of peer-reviewed source unavailable |
| A survey of health care professionals ' views on exercise as a treatment for persons diagnosed with amyotrophic lateral sclerosis – Lewis, M. (2012) | Full-text of peer-reviewed source unavailable |
| People living with ALS and their caregiver's input into drug development – Charpentier, B. (2017) | Full-text of peer-reviewed source unavailable |
| Motor neurone disease – Ng, L. (2011) | Full-text of peer-reviewed source unavailable |
| Use of advance directives in patients with amyotrophic lateral sclerosis – Takacs, S. (2020) | Full-text of peer-reviewed source unavailable |
| An exploratory mixed methods study of patients with motor neurone disease who are on non-invasive ventilation – Ando, H. (2011) | Full-text of peer-reviewed source unavailable |
| Supporting MND patients using NIV – Cousins, R. (2017) | Full-text of peer-reviewed source unavailable |
| Quality of life in adults with spinal muscular atrophy – Bermudez, C. A. (2020) | Full-text of peer-reviewed source unavailable |
| What are MND patients really distressed about? Using the concerns checklist in an MND clinic to enhance the recognition and management of distress – Williams, L. (2018) | Full-text of peer-reviewed source unavailable |
| Exploring the psychological support preferences of informal carers of people with motor neuron disease – Bretten, J. (2021) | Full-text of peer-reviewed source unavailable |
| Measuring value – Tsou, A. (2012) | Full-text of peer-reviewed source unavailable |
| Barriers and facilitators to continuing non-invasive ventilation use in individuals with amyotrophiclateralsclerosis – Marsella, J. (2022) | Full-text of peer-reviewed source unavailable |
| Amyotrophic lateral sclerosis patients' quality of life and their caregiver burden during COVID-19 pandemic – Bombaci, A. (2021) | Full-text of peer-reviewed source unavailable |
| Desire to Die in a Multi-site Cohort of Patients with Amyotrophic Lateral Sclerosis – Thurn, T. (2023) | Full-text of peer-reviewed source unavailable |
| Comparision of health utility measures for amyotrophic lateral sclerosis in the general Korean population – Kim, J. (2017) | Full-text of peer-reviewed source unavailable |
| How do Motor Neurone Disease (MND) patients perceive a Home Visit(HV) service at the Royal Berkshire Hospital (RBH)? – Knight, F. (2022) | Full-text of peer-reviewed source unavailable |
| Stakeholder Perspectives on the Biopsychosocial and Spiritual Realities of Living With ALS – Kukulka, K. (2019) | Cross-sectional study with no explicit outcome valuation |
| Advance Care Planning Among Patients With Amyotrophic Lateral Sclerosis – Phillips, J. (2024) | Cross-sectional study with no explicit outcome valuation |
| Quality of Life Perspectives of People With Amyotrophic Lateral Sclerosis and Their Caregivers – Johnson, S. (2017) | Cross-sectional study with no explicit outcome valuation |
| Understanding the needs of people with ALS – Brizzi, K. T. (2020) | Cross-sectional study with no explicit outcome valuation |
| Decision-making for tracheostomy in amyotrophic lateral sclerosis (ALS) – Ceriana, P. (2017) | Cross-sectional study with no explicit outcome valuation |
| An update on opinions and behaviors of Japanese and American ALS caregivers regarding tracheostomy with invasive ventilation (TIV) – Christodoulou, G. (2014) | Cross-sectional study with no explicit outcome valuation |
| Patients' perspectives of multidisciplinary home-based telehealth for amyotrophic lateral sclerosis – James, N. (2017) | Cross-sectional study with no explicit outcome valuation |
| Usability of eyetracking computer systems and impact on psychological wellbeing in patients with advanced amyotrophic lateral sclerosis – Linse, K. (2018) | Cross-sectional study with no explicit outcome valuation |
| Japanese and American ALS patient preferences regarding TIV (tracheostomy with invasive ventilation) – Rabkin, J. (2014) | Cross-sectional study with no explicit outcome valuation |
| Well-being on supportive techniques in amyotrophic lateral sclerosis – Barc, K. (2022) | Cross-sectional study with no explicit outcome valuation |
| Gastrostomy uptake in motor neurone disease – Labra, J. (2020) | Cross-sectional study with no explicit outcome valuation |
| Understanding neck collar preferences and user experiences in motor neuron disease – Spears, S. D. J. (2024) | Cross-sectional study with no explicit outcome valuation |
| Treatment of respiratory impairment in patients with motor neuron disease in the Netherlands – Raaphorst, J. (2013) | Cross-sectional study with no explicit outcome valuation |
| The diagnostic, therapeutic and assistance pathway for amyotrophic lateral sclerosis in a north-eastern Italian region – Palese, F. (2022) | Cross-sectional study with no explicit outcome valuation |
| Service use and needs of people with motor neurone disease and their carers in Scotland – van Teijlingen, E. R. (2001) | Cross-sectional study with no explicit outcome valuation |
| Patients' self-perceived burden, caregivers' burden and quality of life for amyotrophic lateral sclerosis patients – Geng, D. (2017) | Cross-sectional study with no explicit outcome valuation |
| Experience matters – Aho-Ozhan, H. E. A. (2017) | Cross-sectional study with no explicit outcome valuation |
| Quality of life and mental health in the locked-in-state-differences between patients with amyotrophic lateral sclerosis and their next of kin – Aust, E. (2022) | Cross-sectional study with no explicit outcome valuation |
| Patient reported outcome measures (PROMs) in amyotrophic lateral sclerosis – De Marchi, F. (2020) | Cross-sectional study with no explicit outcome valuation |
| Embedded Psychiatric Services in a Multidisciplinary Amyotrophic Lateral Sclerosis Clinic – Hardy, M. (2022) | Cross-sectional study with no explicit outcome valuation |
| Acceptability and preferences of six different routes of drug application for acute breathlessness – Simon, S. T. (2012) | Cross-sectional study with no explicit outcome valuation |
| Quality of life for ventilator-dependent ALS patients and their caregivers – Gelinas, D. F. (1998) | Cross-sectional study with no explicit outcome valuation |
| Individual and health-related quality of life assessment in amyotrophic lateral sclerosis patients and their caregivers – Lo Coco, G. (2005) | Cross-sectional study with no explicit outcome valuation |
| A study comparing patients with amyotrophic lateral sclerosis and their caregivers on measures of quality of life, depression, and their attitudes toward treatment options – Trail, M. (2003) | Cross-sectional study with no explicit outcome valuation |
| Amyotrophic lateral sclerosis patients' self-reported satisfaction with assistive technology – Gruis, K. L. (2011) | Cross-sectional study with no explicit outcome valuation |
| ALS patient and caregiver attitudes toward physician-hastened death in California – Hauswirth, A. G. (2021) | Cross-sectional study with no explicit outcome valuation |
| Taste changes in amyotrophic lateral sclerosis and effects on quality of life – Tarlarini, C. (2019) | Cross-sectional study with no explicit outcome valuation |
| An observational study on quality of life and preferences to sustain life in locked-in state – Kuzma-Kozakiewicz, M. (2019) | Cross-sectional study with no explicit outcome valuation |
| Assessing individual quality of life in amyotrophic lateral sclerosis – Clarke, S. (2001) | Cross-sectional study with no explicit outcome valuation |
| Depression and QOL in patients with ALS – Grehl, T. (2011) | Cross-sectional study with no explicit outcome valuation |
| The preferences of people with amyotrophic lateral sclerosis on riluzole treatment in Europe – Ludolph, A. C. (2023) | Cross-sectional study with no explicit outcome valuation |
| Quality of life and content with treatment among patients with amyotrophic lateral sclerosis – Leirvik, A. (2006) | Cross-sectional study with no explicit outcome valuation |
| Patient-Reported Outcome of Physical Therapy in Amyotrophic Lateral Sclerosis – Meyer, Robert (2018) | Cross-sectional study with no explicit outcome valuation |
| Development and validation of a short measure of health status for individuals with amyotrophic lateral sclerosis/motor neurone disease – Jenkinson, C. (1999) | Cross-sectional study with no explicit outcome valuation |
| The impact on the family carer of motor neurone disease and intervention with noninvasive ventilation – Baxter, Susan K. (2013) | Cross-sectional study with no explicit outcome valuation |
| Quality of life in patients with amyotrophic lateral sclerosis – Nelson, N. D. (2003) | Cross-sectional study with no explicit outcome valuation |
| Illness perceptions, mood and health-related quality of life in patients with amyotrophic lateral sclerosis – Miglioretti, Massimo (2008) | Cross-sectional study with no explicit outcome valuation |
| [Quality of life of patients with amyotrophic lateral sclerosis] – Leirvik, Atle (2006) | Cross-sectional study with no explicit outcome valuation |
| Quality of life of patients with amyotrophic lateral sclerosis – Tramonti, Francesco (2012) | Cross-sectional study with no explicit outcome valuation |
| Speech therapy and communication device – Korner, S. (2013) | Cross-sectional study with no explicit outcome valuation |
| Evaluation of quality of life and mood disorders in caregivers of patients with amyotrophic lateral sclerosis – Rafiemehr, M. (2020) | Cross-sectional study with no explicit outcome valuation |
| Health-related quality of life and supportive care in patients with rare long-term neurological conditions – Calvert, M. (2013) | Cross-sectional study with no explicit outcome valuation |
| Prospective study to evaluate quality of life in amyotrophic lateral sclerosis – Caballero-Eraso, C. (2023) | Cross-sectional study with no explicit outcome valuation |
| Withdrawal of invasive home mechanical ventilation in patients with advanced amyotrophic lateral sclerosis – Dreyer, Pia Sander (2012) | Cross-sectional study with no explicit outcome valuation |
| Telephone followâ€up for patients with amyotrophic lateral sclerosis – Mannino, M (2007) | Cross-sectional study with no explicit outcome valuation |
| Psychological as well as illness factors influence acceptance of non-invasive ventilation (NIV) and gastrostomy in amyotrophic lateral sclerosis (ALS) – Martin, Naomi H (2014) | Cross-sectional study with no explicit outcome valuation |
| Modelling disease course in amyotrophic lateral Sclerosis – Prell, T. (2020) | Cross-sectional study with no explicit outcome valuation |
| Neurologic disease at the end of life – Goy, E. R. (2008) | Cross-sectional study with no explicit outcome valuation |
| A prospective study of preferences and actual treatment choices in ALS – Albert, S. M. (1999) | Cross-sectional study with no explicit outcome valuation |
| Measuring Health-Related Quality of Life in Amyotrophic Lateral Sclerosis – mcdool, Emily (2024) | Qualitative study - to be considered in subsequent review |
| Decision-making about reproductive choices among individuals at risk for familial amyotrophic lateral sclerosis in families with a known genetic mutation – Holley, D. (2012) | Qualitative study - to be considered in subsequent review |
| What do people living with MND think about dysphagia? – Lisiecka, D. (2016) | Qualitative study - to be considered in subsequent review |
| Home ventilation for amyotrophic lateral sclerosis patients – Moss, A. H. (1993) | Qualitative study - to be considered in subsequent review |
| Still beneficial as motor neurone disease (MND) progresses? Prolonged use of non-invasive ventilation (NIV) from month 12 until death – Ando, H. (2012) | Qualitative study - to be considered in subsequent review |
| Development of a model to guide decision making in amyotrophic lateral sclerosis multidisciplinary care – Hogden, A. (2015) | Qualitative study - to be considered in subsequent review |
| Overcoming therapeutic nihilism. Breaking bad news of amyotrophic lateral sclerosis-a patient-centred perspective in rare diseases – Maksymowicz, S. (2022) | Qualitative study - to be considered in subsequent review |
| Perceptions of quality of life in motor neurone disease – Ando, H. (2013) | Qualitative study - to be considered in subsequent review |
| The lived experience of fatigue in MND – Gibbons, C. (2010) | Qualitative study - to be considered in subsequent review |
| A qualitative study to construct a predictive scale on internal conflict for people with amyotrophic lateral sclerosis – Nakai, M. (2014) | Qualitative study - to be considered in subsequent review |
| The experiences of, and need for, palliative care for people with motor neurone disease and their informal caregivers – Flemming, K. (2020) | Qualitative study - to be considered in subsequent review |
| Understanding quality of life in motor neurone disease – Ando, H. (2013) | Qualitative study - to be considered in subsequent review |
| Understanding psycho-social processes underpinning engagement with services in motor neurone disease – Foley, G. (2014) | Qualitative study - to be considered in subsequent review |
| Patient Preferences for Treatments of Neuromuscular Diseases – Landfeldt, E. (2017) | Qualitative study - to be considered in subsequent review |
| Acceptance and decision making in amyotrophic lateral sclerosis from a life-course perspective – Foley, G. (2014) | Qualitative study - to be considered in subsequent review |
| The impact of home mechanical ventilation on the time and manner of death for those with Motor neurone disease (MND) – Caswell, Glenys (2024) | Qualitative study - to be considered in subsequent review |
| Recognition by family members that relatives with neurodegenerative disease are likely to die within a year – Hubbard, G. (2012) | Qualitative study - to be considered in subsequent review |
| Proof of love and other themes – Herz, H. (2006) | Qualitative study - to be considered in subsequent review |
| Control in the absence of choice – van Eenennaam, R. M. (2023) | Qualitative study - to be considered in subsequent review |
| Experiences of burden, needs, rewards and resilience in family caregivers of people living with Motor Neurone Disease/Amyotrophic Lateral Sclerosis – Weisser, F. B. (2015) | Qualitative study - to be considered in subsequent review |
| How and why people with amyotrophic lateral sclerosis engage with health care services – Foley, G. (2014) | Qualitative study - to be considered in subsequent review |
| Perceptions of quality of life in people with ALS – Foley, Geraldine (2007) | Qualitative study - to be considered in subsequent review |
| A Qualitative Study of Multidisciplinary ALS Clinic Use in the United States – Stephens, Helen E. (2015) | Qualitative study - to be considered in subsequent review |
| Decision-making and referral processes for patients with motor neurone disease – Baxter, Susan (2017) | Qualitative study - to be considered in subsequent review |
| What is unique about quality of life in motor neurone disease? – Young, C. (2014) | Qualitative study - to be considered in subsequent review |
| Choices and control-investigating the needs, thoughts and wishes of people with motor neurone disease in the area of death, dying and end-of-life decision making – Cooney, G. (2012) | Qualitative study - to be considered in subsequent review |
| Need and value of case management in multidisciplinary ALS care – Bakker, M. (2015) | Qualitative study - to be considered in subsequent review |
| A Discourse Analysis – Kitt-Lewis, E. (2018) | Qualitative study - to be considered in subsequent review |
| Amyotrophic lateral sclerosis and assisted ventilation – Lemoignan, J. (2010) | Qualitative study - to be considered in subsequent review |
| The final month of life in patients with ALS – Ganzini, L. (2002) | Qualitative study - to be considered in subsequent review |
| Patient-identified impact of symptoms in spinal and bulbar muscular atrophy – Guber, R. D. (2018) | Qualitative study - to be considered in subsequent review |
| 'The shock of diagnosis' – Aoun, S. M. (2018) | Qualitative study - to be considered in subsequent review |
| Accessing and Receiving Speech-Language Pathology Services at the Multidisciplinary Amyotrophic Lateral Sclerosis Clinic – Huynh, A. (2023) | Qualitative study - to be considered in subsequent review |
| Accepting or declining non-invasive ventilation or gastrostomy in amyotrophic lateral sclerosis – Greenaway, L. P. (2015) | Qualitative study - to be considered in subsequent review |
| Finding meaning despite anxiety over life and death in amyotrophic lateral sclerosis patients – Ozanne, A. O. (2013) | Qualitative study - to be considered in subsequent review |
| PND74 MAPPING A QUALITATIVE EXPLORATION OF MEANINGFUL CHANGE IN LATER-ONSET (TYPE II OR III) SPINAL MUSCULAR ATROPHY TO THE HAMMERSMITH FUNCTIONAL MOTOR SCALE EXPANDED (HFMSE) – Burbridge, C. (2019) | Qualitative study - to be considered in subsequent review |
| Exploring and Addressing 'Concerns' for Significant Others to Extend the Understanding of Quality of Life With Amyotrophic Lateral Sclerosis – Ando, H. (2019) | Qualitative study - to be considered in subsequent review |
| Palliative and End-of-Life Care for People Living with Motor Neurone Disease – Velaga, V. C. (2023) | Qualitative study - to be considered in subsequent review |
| Why don't they accept non-invasive ventilation? – Ando, H. (2015) | Qualitative study - to be considered in subsequent review |
| Amyotrophic lateral sclerosis patients' perspectives on use of mechanical ventilation – Young, J. M. (1994) | Qualitative study - to be considered in subsequent review |
| "Getting ready for the adult world" – Wan, Hamish W. Y. (2019) | Qualitative study - to be considered in subsequent review |
| Interviews with patients, family, and caregivers in amyotrophic lateral sclerosis – Bolmsjo, I. (2001) | Qualitative study - to be considered in subsequent review |
| How people living with motor neurone disease and their carers experience healthcare decision making – Paynter, C. (2022) | Qualitative study - to be considered in subsequent review |
| Understanding family experiences of inherited MND – Howard, J. (2022) | Qualitative study - to be considered in subsequent review |
| Control in the absence of choice – Van Eenennaam, R. (2022) | Qualitative study - to be considered in subsequent review |
| Developing a model of patientcentred decision-making for amyotrophic lateral sclerosis multidisciplinary care – Hogden, A. (2013) | Qualitative study - to be considered in subsequent review |
| Palliative care for patients with motor neurone disease and their bereaved carers – mcveigh, C. (2019) | Qualitative study - to be considered in subsequent review |
| Needs and preferences for psychological interventions of people with motor neuron disease – Weeks, K. R. (2019) | Qualitative study - to be considered in subsequent review |
| A qualitative meta-synthesis of evidence (meta-ethnography) exploring the personal experiences of gastrostomy tube in neurodegenerative diseases – Lisiecka, D. (2022) | Qualitative study - to be considered in subsequent review |
| Understanding living with tracheostomy ventilation for motor neuron disease and the implications for quality of life – Wilson, E. (2023) | Qualitative study - to be considered in subsequent review |
| Decision Making About Gastrostomy and Noninvasive Ventilation in Amyotrophic Lateral Sclerosis – Martin, N. H. (2016) | Qualitative study - to be considered in subsequent review |
| "I hate being a burden" – Foley, G. (2016) | Qualitative study - to be considered in subsequent review |
| A qualitative evaluation of the revised amyotrophic lateral sclerosis functional rating scale (ALSFRS-R) by the patient community – Boyce, D. (2023) | Qualitative study - to be considered in subsequent review |
| End of life decision making when home mechanical ventilation is used to sustain breathing in Motor Neurone Disease – Wilson, E. (2024) | Qualitative study - to be considered in subsequent review |
| EQ-5D and SF-6D health utility scores in patients with spinal and bulbar muscular atrophy – Xu, Richard Huan (2023) | Ineligible participant population |
| How would terminally ill patients have others make decisions for them in the event of decisional incapacity? A longitudinal study – Sulmasy, D. P. (2007) | Ineligible participant population |
| Assessing Symptoms, Concerns, and Quality of Life in Noncancer Patients at End of Life – Hack, Thomas F. (2018) | Ineligible participant population |
| [Survey report on end-of-life care for ALS patients of ALS physicians in Japan] – Ogino, Mieko (2010) | Ineligible participant population |
| Home trials of a speech synthesizer in severe dysarthria – Laffont, I. (2007) | Ineligible participant population |
| Evaluation of health-related quality of life using the MOS 36-Item Short-Form Health Status Survey in patients receiving noninvasive positive pressure ventilation – Windisch, W. (2003) | Ineligible participant population |
| Evaluation of quality of life in complete locked-in syndrome patients – Rousseau, M. C. (2013) | Ineligible participant population |
| The survey report about the end-of-life care with ALS patients of the ALS physicians in Japan – Ogino, M. (2010) | Ineligible participant population |
| Attitude of outpatients with neuromuscular diseases in Japan to pain and use of analgesics – Abe, Y. (2008) | Ineligible participant population |
| Treatment expectations and patient-reported outcomes of nusinersen therapy in adult spinal muscular atrophy – Osmanovic, A. (2020) | Ineligible participant population |
| The attitude of neurologists to end-of-life care in amyotrophic lateral sclerosis – Barc, K. (2018) | Ineligible participant population |
| Population screening for spinal muscular atrophy – Boardman, F. K. (2017) | Ineligible participant population |
| Spinal Muscular Atrophy Update in Best Practices – Schroth, Mary K. (2025) | Ineligible participant population |
| Integrating preferences into health status assessment for amyotrophic lateral sclerosis – Beusterien, Kathleen (2005) | Ineligible participant population |
| Health-related quality of life in patients with myotonic dystrophy type 1 and amyotrophic lateral sclerosis – Peric, S. (2010) | Ineligible participant population |
| Short form 36 assessed health-related quality of life after focal spasticity therapy – Bergfeldt, U. (2009) | Ineligible participant population |
| Illness Perceptions And Quality Of Life In Adolescents With Neuromuscular Disorders And Caregivers – Prikken, S. (2022) | Ineligible participant population |
| Deriving summary indices of health status from the Amyotrophic Lateral Sclerosis Assessment Questionnaires (ALSAQ-40 and ALSAQ-5) – Jenkinson, C. (2003) | Ineligible participant population |
| Trial Participation in Neurodegenerative Diseases – Weemering, D. N. (2024) | Ineligible participant population |
| Supporting people with Motor Neuron Disease (MND) to make decisions about gastrostomy feeding tube placement – White, S. (2024) | Ineligible participant population |
| Advance care planning for patients with advanced neurology diseases – Cheung, K. C. (2018) | Ineligible participant population |
| Comparison of direct and indirect methods of estimating health state utilities for resource allocation – Arnold, D. (2009) | Ineligible participant population |
| Assessing Patient Perspectives on Receiving Bad News – Mirza, R. D. (2019) | Ineligible participant population |
| An exploratory qualitative assessment of patient and clinician perspectives on patient-reported outcome measures and disease-modifying therapies in adults with spinal muscular atrophy – Slayter, Jeremy (2025) | Ineligible participant population |
| Uncertainty and opposition of medical students toward assisted death practices – Warner, T. D. (2001) | Ineligible participant population |
| Korean Medical Professionals' Attitudes and Experiences on Advance Care Planning for Noncancerous Disease – Lee, Ji Eun (2019) | Ineligible participant population |
| EP.42Health Utility index scores in patients with spinal muscular atrophy – Belter, L. (2019) | Ineligible participant population |
| Physicians' own preferences to the limitation and withdrawal of life-sustaining therapy – Marik, P. E. (1999) | Ineligible participant population |
| Systematic Literature Review to Identify Utility Values in Patients with Spinal Muscular Atrophy (SMA) and Their Caregivers – Sutherland, C. S. (2022) | Ineligible participant population |
| Patient and Caregiver Treatment Preferences in Type 2 and Non-ambulatory Type 3 Spinal Muscular Atrophy – Lo, S. H. (2022) | Ineligible participant population |
| Preferences and Utilities for Treatment Attributes in Type 2 and Non-ambulatory Type 3 Spinal Muscular Atrophy in the United Kingdom – Lo, S. H. (2022) | Ineligible participant population |
| Reinvent – Pierucci, P. (2021) | Ineligible participant population |
| Institutions, culture, and chronically ill patients' willingness to pay for medical treatment – Chaikumbung, M. (2022) | Ineligible participant population |
| "The Whole Game is Changing and You've Got Hope" – Farrar, M. A. (2020) | Ineligible participant population |
| Physicians' attitudes toward end-of-life decisions in amyotrophic lateral sclerosis – Thurn, Tamara (2019) | Ineligible participant population |
| Wish to die in end-stage ALS [9] – Kennedy, P. R. (2006) | No valuation of satisfaction or health and quality of life related outcome |
| Airway Clearance in Neuromuscular Disease – Sheers, N. L. (2024) | No valuation of satisfaction or health and quality of life related outcome |
| Should hospices offer respite admissions to patients with motor neurone disease? – Hicks, F. (1993) | No valuation of satisfaction or health and quality of life related outcome |
| Designing and validating a Hereditary Spastic Paraplegia-specific Quality of Life Rating Scale (hspqol) – Siow, S. F. (2024) | No valuation of satisfaction or health and quality of life related outcome |
| Breaking the news of a diagnosis of motor neurone disease – Aoun, Samar M. (2016) | No valuation of satisfaction or health and quality of life related outcome |
| Assessing palliative care outcomes for people with motor neurone disease living at home – Hughes, Rhidian A. (2004) | No valuation of satisfaction or health and quality of life related outcome |
| A Journey with Amyotrophic Lateral Sclerosis – Anonymous, (2018) | No valuation of satisfaction or health and quality of life related outcome |
| Psychological function in individuals with amyotrophic lateral sclerosis (ALS) – Brown, W. A. (1970) | No valuation of satisfaction or health and quality of life related outcome |
| A 10-year literature review of family caregiving for motor neurone disease – Aoun, S. M. (2013) | No valuation of satisfaction or health and quality of life related outcome |
| Quality of life for ventilator-dependent ALS patients and their caregivers – Gelinas, D. F. (1998) | No valuation of satisfaction or health and quality of life related outcome |
| How the prospect of a clinical trial impacts decision-making for predictive genetic testing in ALS – Fontaine, Myriam (2024) | No valuation of satisfaction or health and quality of life related outcome |
| Frequency of euthanasia, factors associated with end-of-life practices, and quality of end-of-life care in patients with amyotrophic lateral sclerosis in the Netherlands – van Eenennaam, R. M. (2023) | No valuation of satisfaction or health and quality of life related outcome |
| Respiratory impairment in patients with motor neuron disease – Raaphorst, J. (2013) | No valuation of satisfaction or health and quality of life related outcome |
| Patients' choices regarding ventilatory support affect opioid use in amyotrophic lateral sclerosis – Matsuda, C. (2025) | No valuation of satisfaction or health and quality of life related outcome |
| Family understanding of seriously-ill patient preferences for family involvement in healthcare decision making – Sharma, R. K. (2011) | No valuation of satisfaction or health and quality of life related outcome |
| Decisions about life support in amyotrophic lateral sclerosis – Goldblatt, D. (1984) | No valuation of satisfaction or health and quality of life related outcome |
| Experience and Perspectives in the US on the Evolving Treatment Landscape in Spinal Muscular Atrophy – Ramos-Platt, L. (2022) | No valuation of satisfaction or health and quality of life related outcome |
| A national study of amyotrophic lateral sclerosis multidisciplinary clinic utilization – Young, J. (2010) | No valuation of satisfaction or health and quality of life related outcome |
| Handicap and quality of life assessment in amyotrophic lateral sclerosis – Danel-Brunaud, V. (2006) | No valuation of satisfaction or health and quality of life related outcome |
| The study of chronic partial denervation and quality of life in patients with motor neuron disease treated with semax – Serdiuk, A. V. (2007) | No valuation of satisfaction or health and quality of life related outcome |
| Patients with amyotrophic lateral sclerosis receiving long-term mechanical ventilation – Moss, A. H. (1996) | No valuation of satisfaction or health and quality of life related outcome |
| Dignity therapy – Bentley, B. (2013) | No valuation of satisfaction or health and quality of life related outcome |
| Information needs and preferences in ALS patients in the Netherlands – Sloots, M. (2021) | No valuation of satisfaction or health and quality of life related outcome |
| Updates on amyotrophic lateral sclerosis – Bradley, W. G. (2009) | No valuation of satisfaction or health and quality of life related outcome |
| The Right Therapy for Neurological Disorders – Bruijn, L. I. (2016) | No valuation of satisfaction or health and quality of life related outcome |
| Development and validation of a short measure of health status for individuals with amyotrophic lateral sclerosis/motor neurone disease – Jenkinson, C. (1999) | No valuation of satisfaction or health and quality of life related outcome |
| Patient reported outcome measures in Amyotrophic Lateral Sclerosis – De Marchi, F. (2019) | No valuation of satisfaction or health and quality of life related outcome |
| Tracheostomy and invasive ventilation in Japanese ALS patients – Tagami, M. (2014) | No valuation of satisfaction or health and quality of life related outcome |
| Amyotrophic lateral sclerosis care in Saudi Arabia – Abuzinadah, A. R. (2020) | No valuation of satisfaction or health and quality of life related outcome |
| Is dignity therapy feasible to enhance the end of life experience for people with motor neurone disease and their family carers? – Bentley, B. (2012) | No valuation of satisfaction or health and quality of life related outcome |
| Current practices and barriers in gastrostomy indication in amyotrophic lateral sclerosis – Van Eenennaam, R. M. (2022) | No valuation of satisfaction or health and quality of life related outcome |
| Online assessment of ALS functional rating scale compares well to in-clinic evaluation – Maier, A. (2012) | No valuation of satisfaction or health and quality of life related outcome |
| Randomised controlled trial in non-invasive ventilation – Pinto, A. (2004) | No valuation of satisfaction or health and quality of life related outcome |
| Estimating the minimum important difference in the ALSFRS-R-instrument in people living with MND – Boddy, S. L. (2025) | Pre-test, post-test study with no explicit outcome valuation |
| Amyotrophic lateral sclerosis caregiver burden and patients' quality of life during COVID-19 pandemic – Giusiano, S. (2022) | Pre-test, post-test study with no explicit outcome valuation |
| SOD1 gene screening in ALS-frequency of mutations, patients' attitudes to genetic information and transition to tofersen treatment in a multi-center program – Meyer, T. (2025) | Pre-test, post-test study with no explicit outcome valuation |
| Improved ALS clinical trials through frequent at-home self-assessment – Rutkove, S. B. (2020) | Pre-test, post-test study with no explicit outcome valuation |
| Depression and quality of life in patients with amyotrophic lateral sclerosis – Lule, D. (2008) | Pre-test, post-test study with no explicit outcome valuation |
| Edaravone for the treatment of amyotrophic lateral sclerosis – Takei, K. (2018) | Pre-test, post-test study with no explicit outcome valuation |
| Influence of a clinical trial in the decision-making processes of patients with amyotrophic lateral sclerosis – Kato, C. (2022) | Pre-test, post-test study with no explicit outcome valuation |
| Healthcare Utilisation and Satisfaction with Care in Patients with Amyotrophic Lateral Sclerosis - An Observational Study – Kierkegaard, M. (2021) | Pre-test, post-test study with no explicit outcome valuation |
| Minimally Detectable Change and Minimal Clinically Important Difference of a Decline in Sentence Intelligibility and Speaking Rate for Individuals With Amyotrophic Lateral Sclerosis – Stipancic, K. L. (2018) | Pre-test, post-test study with no explicit outcome valuation |
| Differences in quality of life modalities give rise to needs of individual support in patients with ALS and their next of kin – Olsson, A. G. (2010) | Pre-test, post-test study with no explicit outcome valuation |
| Disease progression and perceptions of health in patients with motor neurone disease – Young, C. A. (1995) | Pre-test, post-test study with no explicit outcome valuation |
| A longitudinal study on quality of life and depression in ALS patient-caregiver couples – Gauthier, A. (2007) | Pre-test, post-test study with no explicit outcome valuation |
| Cross-sectional and longitudinal correlations between disease progression and different health-related quality of life domains in persons with amyotrophic lateral sclerosis – De Droot, I. J. M. (2007) | Pre-test, post-test study with no explicit outcome valuation |
| Health-related quality of life in amyotrophic lateral sclerosis – Norquist, J. M. (2004) | Pre-test, post-test study with no explicit outcome valuation |
| Measurement and Impact of Physical Impairment in Amyotrophic Lateral Sclerosis – Beaumont, Hospital (2025) | Registration record of ongoing research study |
| Understanding Palliative Care and End of Life Needs in the ALS Population – A. L. S. Society of Canada (2020) | Registration record of ongoing research study |
| Effects of Long Term Ventilation Support on the Quality of Life of ALS Patients and Their Families – Oslo University, Hospital (2023) | Registration record of ongoing research study |
| Online survey study to explore patient and caregiver preferences for different spinal muscular atrophy treatments – ISRCTN11372270 (2021) | Registration record of ongoing research study |
| ALS caregivers – NL-OMON28612 (2016) | Registration record of ongoing research study |
| Effects of aerobic exercise therapy and cognitive behavioural therapy on functioning and quality of life in amyotrophic lateral sclerosis – van Groenestijn, A. C. (2011) | Registration record of ongoing research study |
| Dysphagia, qol and Attitudes Towards PEG in ALS Patients – NCT05336760 (2022) | Registration record of ongoing research study |
| Development of a novel patient reported outcome measure for health-related quality of life in amyotrophic lateral sclerosis (proquals) – Carlton, J. (2024) | Registration record of ongoing research study |
| PATIENT REPORTED OUTCOME MEASURES OF MOUTHPIECE VENTILATION IN NEUROMUSCULAR CONDITIONS – Chowdhury, A. M. (2024) | Registration record of ongoing research study |
| Major Determinants of Quality of Life and Caregiver Burden in Primary Lateral Sclerosis – Beutter, E. (2024) | Registration record of ongoing research study |
| Brain-computer interfaces patient preferences – Brannigan, J. F. M. (2024) | Eligible systematic review - included studies screened individually |
| Elucidating the End-of-Life Experience of Persons With Amyotrophic Lateral Sclerosis – Long, R. (2019) | Eligible systematic review - included studies screened individually |
| Preference-based measures of health-related quality of life in congenital mobility impairment – Bray, N. (2020) | Eligible systematic review - included studies screened individually |
| Decision-making among patients and their family in ALS care – Foley, G. (2018) | Eligible systematic review - included studies screened individually |
| Patients' and caregivers' attitudes towards patient assisted suicide or euthanasia in amyotrophic lateral sclerosis-a meta-analysis – Mavroudis, I. (2024) | Eligible systematic review - included studies screened individually |
| Advance care planning in motor neuron disease – Murray, L. (2016) | Eligible systematic review - included studies screened individually |
| Assisted nutrition – Voltz, R. (2015) | Editorial/commentary/brief report |
| Terminal sedation – Etkind, S. N. (2012) | Editorial/commentary/brief report |
| Battling with motor neurone disease – Wilson, B. (1982) | Editorial/commentary/brief report |
| Choosing the place of death – Chhetri, S. K. (2015) | Editorial/commentary/brief report |
| "No feeding tubes for me! – Nicholson, R. H. (1987) | Editorial/commentary/brief report |
| End of life in patients with amyotrophic lateral sclerosis (ALS) – Connolly, S. (2014) | Non-systematic review |
| Living and dying with respiratory failure – Simonds, A. K. (2004) | Non-systematic review |
| Gastrostomy use in motor neurone disease (MND) – Stavroulakis, T. (2013) | Non-systematic review |
| Maid in Canada – Johnston, W. S. (2018) | Non-systematic review |
| Palliative care at the end of life for intractable neurological diseases – Namba, R. (2006) | Report not retrieved as inability to translate to English |
| Mechanical ventilation for amyotropic lateral sclerosis making a comparison between hospital and home care – Saito, T. (1999) | Report not retrieved as inability to translate to English |

**Supplementary material 4 - Characteristics of included studies**

**Supplementary table 2 -** Summary of the key characteristics of the included studies

| Study ID | Country | Time period | Study design | Disease investigated | Population (participant category and sample size) | Sampling strategy | Outcomes with valuation |
| --- | --- | --- | --- | --- | --- | --- | --- |
| Ackerman 1999^1^ | USA and Canada | Retrospective analysis using participant data from an RCT that was conducted from January 1993 to April 1995 | Standard gamble | ALS | Clinicians providing proxy measures for adults diagnosed with ALS (n=10) | Not described in detail - likely either purposive or consecutive sampling | - Living with ALS (Appel Score = 40 to 59) - Living with ALS (Appel Score = 60 to 86) - Living with ALS (Appel Score = 87 to 109) - Living with ALS (Appel Score = 110 to 128) - Living with ALS (Appel Score = 129 to 164) |
| Adelman 2004^2^ | USA | Not specified – authors reported first visit was in July 2003 | Pre-post study- with structured interview | ALS | Adults with ALS (n=69) and their caregivers (n=69) | Purposive sampling | - How interested is the patient in hastening death - How much does the patient want to live? |
| Akerblom 2021^3^ | Sweden | September 2015 to September 2016 | Cross-sectional | Motor Neurone Disease (including ALS, MND with lower motor neuron signs, and PLS) | Adults with MND (n=55) | Purposive sampling | - Being independent - Being able to work - Being physically active - Being in good health - Being able to travel - Hope for the future |
| Conroy 2023^4^ | Nine European countries:   - Italy - France - Netherlands - Spain - Ireland - UK - Belgium - Germany - Sweden | October to December 2020 | Cross-sectional | ALS | Adults with ALS (n=857) | Convenience sampling | - Stop the progression of ALS/MND - Breathing/respiratory function - Mobility - Speaking/Communication - Daily self-care - Overall physical function - Muscle weakness - Ease the overall burden of ALS/MND - Fatigue - Swallowing - Cognitive ability-focus and thinking - Overall mental health - Ability to express myself in my relationships - Loss of interest (apathy) - Muscle spasticity - Pain - Laughing/crying uncontrollably - Sexual function - Most important outcomes of a new drug: - The drug prolongs survival but has moderate to severe side effects that some people cannot tolerate - The drug slows (does not halt) the spread of ALS/MND and has minimal-moderate side effects - The drug significantly reduces some symptoms from ALS/MND with side-effects that I can manage but does not affect*… (this is cutoff on the paper, and the full option is not reported. Contacted authors).* - The drug prevents further muscle weakening but does not improve strength - The drug increases muscle strength, even if it has moderate to severe side effects that are difficult to manage. |
| Gebrehiwet 2023^5^ | Six countries:   - USA - Australia - Canada - Ireland - Netherlands - Spain | Not reported | Direct utility and health state value study of data from double-blind, randomized, dose-ranging, placebo-controlled trial | ALS | Adults with ALS (total= 456, Reldesemtiv n = 342, Placebo n =114) | Purposive sampling with 1:1:1:1 randomisation | - Health-related quality of life (EQ-5D-5L) *Estimated from the ALSFRS-R that was administered during the trial. - QALYs |
| Green 2003^6,7^ | UK | April – June 2000 | Cross-sectional | ALS but MND more broadly | Adults diagnosed with MND (n=77) | Purposive sampling | - Standard Gamble (Stay in current health state for two years) - Health-related quality of life (EQ-5D VAS) - Health-related quality of life (EQ-5D DSI) |
| Hecht 2002^8^ | Germany | Not explicitly stated but participants contacted either in 1998 or who were registered in 1998 (not clear which). | Pre-post study with interview | ALS | Adults diagnosed with MND (n=41) | Purposive sampling | - Reduction of speech - Reduction of mobility - Knowledge of the poor prognosis - Becoming dependant on other persons - Impairment of activities of daily living - Loss of writing ability - Swallowing problems - Unimpaired consciousness - Helplessness of medicine - Social isolation |
| Helleman 2022^9^ | Three countries:   - The Netherlands - UK - Australia | November 2020 – November 2021 | Cross-sectional | ALS, PLS and PMA | Adults with MND (n = 332)   - Netherlands: 200 - UK: 91 - Australia: 41 | Purposive sampling   - Netherlands – the national ALS registry was accessed and invitations sent - UK – those who attended the Sheffield MND clinic were invited - Australia – participants were from Victoria, Queensland or Western Australia and were registered on the national MND registry and previous consented to be contacted | - Most valuable outcome measure for home-monitoring: - Respiratory function - Muscle strength - Quality of life - Fine motor/speech - Daily activity - Sleep - Body weight |
| Jones 2014^10^ | UK | May 2009 to November 2011 | Direct utility and health state value study | ALS | Adults with ALS (n=214) | Purposive sampling - participants were recruited from the LiCALS trial: a multicentre, double-blind, randomized parallel group study. | - Health-related quality of life (EQ-5D) reported across clinical stages of the disease |
| Maksymowicz-Sliwinska 2023^11^ | Two countries:   - Poland - Germany | Not explicitly stated-looks January 2014 to December 2016 - from a PDF linked website | Cross-sectional | ALS | Caregivers of adults with MND (informal caregivers), total n=164   - Poland n = 80 - Germany n = 84 | Not described in detail - likely either purposive or consecutive sampling | - Would you like your relative with ALS to use life-prolonging measures? - If it were permitted by law, would you like your relative with ALS to use life-shortening measures? |
| Moore 2019^12^ | UK | Not stated | Direct utility and health state value study - data were obtained from the TONiC study, which is a longitudinal cohort study | MND | Adults with MND (n=595) | Not explicitly reported - patients were recruited from MND clinics | - Health-related quality of life (EQ-5D-5L) - Health-related quality of life (EQ-5D VAS) - Health-related quality of life (ALSUI) |
| Raheja 2016^13^ | Two countries:   - USA - Canada | Retrospective analysis of data from an RCT that was conducted from 4^th^ September 2006 and 30^th^ July 2012 | Cross-sectional survey with direct valuation | ALS | Adults diagnosed with MND (n=82) | Retrospective sample of previously randomised participants | - Non-Bulbar: - Pain score (ALSSQoL) - Fatigue Score (ALSSQoL) - Breathing score (ALSSQoL) - Strength/ability to move score (ALSSQoL) - Sleep score (ALSSQoL) - Bowel and bladder score (ALSSQoL) - Bulbar - Eating score (ALSSQoL) - Excessive Saliva (ALSSQoL) - Mucus score (ALSSQoL) - Speaking score (ALSSQoL |
| Rudnicki 1997^14^ | USA | February 1996 – September 1996 | Cross-sectional | ALS | Adults with MND (n=46) | Consecutive sampling | - Reasons for not taking Riluzole |
| Sandstedt 2018^15^ | Sweden | October 2012 – January 2013 | Cross-sectional | ALS | Informal caregivers of adults with MND (n=49, however only 47 EQVAS results are present) | Consecutive sampling | - Health-related quality of life (EQ-5D-VAS) |
| Seo 2024^16^ | Six countries:   - USA - France - Germany - Italy - Spain - UK | November 2020 – April 2022 | Discrete choice experiment and a threshold technique exercise with online survey | ALS | Adults with MND (n=295) | Convenience sampling | - Patient preferences for intrathecal drug administration - Maximum acceptable risk |
| Stenson 2024^17^ | Six countries:   - USA - France - Germany - Italy - Spain - UK | July 2020 – March 2021 | Cross sectional | ALS | Adults diagnosed with MND (n=880)   - Clinicians providing proxy measures of adults diagnosed with MND (142)   and their carers, 172 pALS had self-reported data | Purposive sampling - clinicians were identified by local fieldwork agents using physician panels and publicly available lists and invited to participate if they had a primary specialty of neurology | - Health-related quality of life (EQ-5D-5L) - Health-related quality of life (EQ-5D VAS) - Health-related quality of life (ALSAQ-5) [Adapted from ALSAQ-40]. |
| Tarlarini 2017^18^ | Italy | July – December 2017 | Pre-post study | ALS | Adults with MND (n=31) | Consecutive sampling | - Pain - Swallowing - Speech - Taste - Chewing - Appearance - Activity - Recreation - Saliva - Mood - Anxiety |
| Thakore 2019^19^ | USA | 2010 – 2015 | Health economic modelling | ALS | Adults with MND (n=678) | Convenience sampling | - Health-related quality of life (EQ-5D-3L and VAS) |
| Tobin 2021^20^ | Ireland | April 2014 – May 2015 | Discrete choice experiment | ALS | Adults with MND (n=93) and Informal caregivers of adults with MND (n=56) | Consecutive sampling | - Disease information - Price of additional services - Arranging visits - Distant to clinic - Waiting times - Place of care - Decisions on care - Personal care provision - Timing of hospice care (adults diagnosed with MND only) - Communication devices (adults diagnosed with MND only) - Availability of phone advice - Provision of emotional support - Dependable Healthcare processionals - Who helps in the home |
| Trail 2004^21^ | USA | January – September 2021 | Cross-sectional | ALS | Adults diagnosed with MND (n = 66)  Caregivers of those with MND (n= 61) | Consecutive sampling | - Lack of family support - Worries about the future - Worries about being dependent on others - Loss of faith in religion - Lack of friendship or changes in friendships - Loneliness - Financial stresses - Emotional swings (laughing, crying, irritability) - Depression - Problems breathing - Problems speaking - Difficulty with self-care (i.e. dressing, bathing) - Difficulty with participating in hobbies/leisure activities - Difficulty at work or inability to work - Problems swallowing/eating - Worries about family/loves ones’ emotional and physical well-being - Muscle weakness - Progression of illness - Problems with mobility |
| vanEijk 2024^22^ | The Netherlands | June 2022 – November 2023 | Cross-sectional | ALS, PMA or PLS | Adults diagnosed with MND (n=382) | Purposive sampling | - Which domain is most important to you? |
| vanEijk 2022^23^ | The Netherlands | May 2021 | Cross-sectional | ALS, PMA or PLS | Adults diagnosed with MND (n = 433)   - ALS n = 271 - PMA n = 74 - PLS n = 88 | Purposive sampling | - Which domain is most important to you? |
| Wagner 2018^24^ | USA | Not specified | Cross-sectional | ALS | Adults diagnosed with ALS (n=449) | Convenience sampling | - If I could “do it all over again” I would choose to have genetic testing - I would recommend that other persons with ALS consider genetic testing |
| Wei 2021^25^ | China | May 2018 – May 2020 | Cross-sectional | ALS | Adults with MND (n=547) | Consecutive sampling | - Health-related quality of life (EQ-5D-5L) - Mobility (EQ-5D-5L) - Self-Care - Usual Activities - Pain/Discomfort - Anxiety/Depression |

**Supplementary table 3 –**Summarised risk of bias assessment for all included studies, assessed using ROBVALU

| Study ID | Selection of participants | Completeness of data | Measurement instrument | Data analysis | Overall risk of bias |
| --- | --- | --- | --- | --- | --- |
| Ackerman 1999 | Serious | Not serious | Not serious | Not serious | Serious |
| Adelman 2004 | Not serious | Not serious | Very serious | Extremely serious | Extremely serious |
| Akerblom 2021 | Serious | Not serious | Extremely serious | Extremely serious | Extremely serious |
| Conroy 2023 | Not serious | Extremely serious | Extremely serious | Extremely serious | Extremely serious |
| Gebrehiwet 2023 | Not serious | Extremely serious | Not serious | Extremely serious | Extremely serious |
| Green 2003 | Not serious | Not serious | Not serious | Not serious | Not serious |
| Hecht 2002 | Not serious | Not serious | Serious | Extremely serious | Extremely serious |
| Helleman 2022 | Not serious | Not serious | Not serious | Not serious | Not serious |
| Jones 2014 | Not serious | Not serious | Not serious | Not serious | Not serious |
| Maksymowicz-Sliwinska 2023 | Serious | Not serious | Not serious | Extremely serious | Extremely serious |
| Moore 2019 | Not serious | Not serious | Not serious | Not serious | Not serious |
| Raheja 2016 | Not serious | Not serious | Not serious | Not serious | Not serious |
| Rudnicki 1997 | Not serious | Not serious | Very serious | Extremely serious | Extremely serious |
| Sandstedt 2018 | Not serious | Not serious | Not serious | Not serious | Not serious |
| Seo 2024 | Not serious | Not serious | Not serious | Not serious | Not serious |
| Stenson 2024 | Not serious | Not serious | Not serious | Not serious | Not serious |
| Tarlarini 2017 | Not serious | Serious | Very serious | Extremely serious | Extremely serious |
| Thakore 2019 | Not serious | Not serious | Not serious | Not serious | Not serious |
| Tobin 2021 | Not serious | Not serious | Not serious | Not serious | Not serious |
| Trail 2004 | Not serious | Not serious | Extremely serious | Extremely serious | Extremely serious |
| VanEijk 2024 | Not serious | Not serious | Not serious | Not serious | Not serious |
| vanEijk 2022 | Not serious | Not serious | Not serious | Not serious | Not serious |
| Wagner 2018 | Not serious | Not serious | Extremely serious | Extremely serious | Extremely serious |
| Wei 2021 | Not serious | Serious | Serious | Not serious | Extremely serious |

**Ackerman 1999**

**Data extraction**

| Study ID | Ackerman 1999 |
| --- | --- |
| ***DESIGN*** | |
| ***Other reports of this study identified?*** | N/A |
| ***Time period study was conducted*** | January 1993 and April 1995 |
| ***Design*** | This study is a post hoc analysis of data collected during the North American rhIGF-I clinical trial. The North American rhIGF-I clinical trial was a multicentre, prospective, randomised, placebo-  controlled study. (<https://pubmed.ncbi.nlm.nih.gov/9409357/>) |
| ***Outcomes with a valuation provided relevant to the review*** | Various health states that were defined and presented to the participants were valued in this study. They were as follows (more details in outcome below)  Perfect Health (normal speech/diet/breathing/dress and feed independently, no mobility aids needed, able to work full time, preventative medical care only / 1) Death (e.g. would die a quick and painless death / 0) Health state 1 (Appel Health state 2 Health state 3 Health state 4  Health state 5 |
| ***SETTING*** | |
| ***Country*** | USA and Canada |
| ***Sites/s*** | Madison, Wisconsin Chicago, Illinois Minneapolis, Minnesota Miami, Florida San Francisco, California Dallas, Texas Durham, North Carolina London, Ontario |
| ***Study locale*** | University affiliated medical centres |
| ***Disease investigated*** | ALS |
| ***PARTICPANTS*** | |
| ***Category of participants under investigation*** | Clinicians as proxy to adults diagnosed with MND |
| ***Eligibility criteria of participants*** | Utilities, or preferences, for the health states were elicited from a multispecialty panel of health care professionals.  To be eligible, candidate panellists were required to fulfill the following criteria: have direct contact with ALS patients representative of all ALS health states, including stages close to death, and have no association with the North American rhIGF-I clinical trial. |
| ***Sampling strategy*** | No description provided as to how the 10-member panel were ultimately selected. |
| ***Number of participants included*** | N=10 |
| ***Characteristics/demographics of participants included*** | The authors have provided the demographics of the patients of the original RCT. However, the participants in this trial that provided the health state valuations were the 10-member panel, and the demographics of this panel were not reported, outside of their profession.  The 10-member panel consisted of 2 neurologists, a nurse clinician, 2 social workers, a nutritionist, a patient advocate, a speech-language pathologist, a physical therapist and a mental health nurse. |
| ***Response rate*** | All panel members participated |
| ***OUTCOME 1: HEALTH STATE 1*** | |
| ***Instrument or method*** | Health state descriptions were derived for the 5 clinical stages of ALS using a disease-specific health state classification system (Appel ALS rating scale).  The preference measurement techniques were rating scale and standard gamble. During the utility assessment exercise, the health state descriptions were labelled using geometric shapes (e.g. health state.) rather than numerical identifiers to avoid imposing any hierarchical order among the 6 health states (i.e. 5 clinical stages and death). The respondent made the choice of the least-preferred health state. Further methods of the standard gamble were not provided. |
| ***Preference set used in translation of raw results*** | N/A |
| ***Reported format*** | Utility weight (mean ± SEM) Health state 1 had the following health state description:   - normal to slightly slurred speech - general diet but need to eliminate hard foods that are difficult to swallow - normal breathing - dress and feed independently and can live independently - may require mobility aids such as a cane - able to work full time - medical procedures are strictly diagnostic |
| ***Result*** | 0.89 ± 0.04 |
| ***OUTCOME 2: HEALTH STATE 2*** | |
| ***Instrument or method*** | As above |
| ***Preference set used in translation of raw results*** | N/A |
| ***Reported format*** | Utility weight (mean ± SEM) Health state 2 had the following health state description:   - speech is intelligible at least three-quarters of the time - diet limited to finely chopped or ground foods and thick liquids - breathing is moderately impaired, experience shortness of breath on exertion, but can cough effectively - require minimal care for dressing and feeding - require mobility aids such as crutches or occasionally a wheelchair - work limited to part time - medical procedures include chest physiotherapy and diagnostic spirometry |
| ***Result*** | 0.82 ± 0.04 |
| ***OUTCOME 3: HEALTH STATE 3*** | |
| ***Instrument or method*** | As above |
| ***Preference set used in translation of raw results*** | N/A |
| ***Reported format*** | Utility weight (mean ± SEM) Health state 3 had the following health state description:   - speech is intelligible less than half of the time - diet limited to food of pudding consistency - breathing impaired to point at increased risk for infections - require caretaker to assist with transfers and to assist with dressing and feeding - require use of a wheelchair much of the time - most likely have stopped working - medical care includes PEG consultations, medications for pain, and many unexpected hospitalisations |
| ***Result*** | 0.41 ± 0.17 |
| ***OUTCOME 4: HEALTH STATE 4*** | |
| ***Instrument or method*** | As above |
| ***Preference set used in translation of raw results*** | N/A |
| ***Reported format*** | Utility weight (mean ± SEM) Health state 4 had the following health state description:   - speech is intelligible less than one-quarter of the time; require a device to communicate - diet is limited to pureed, blended, thick liquids and likely to need tube feedings - respiratory function is impaired to the extent that pressure support (CPAP, BIPAP) and home oxygen may be needed - fully dependent on a caregiver for dressing and feeding - wheelchair-bound - not working - medical care includes consultations regarding need for tracheostomy/gastrostomy, as well as medications for pain |
| ***Result*** | 0.01 ± 0.20 |
| ***OUTCOME 5: HEALTH STATE 5*** | |
| ***Instrument or method*** | As above |
| ***Preference set used in translation of raw results*** | N/A |
| ***Reported format*** | Utility weight (mean ± SEM) Health state 5 had the following health state description:   - unable to speak - have had a gastrostomy - respiratory function severely impaired; have had a tracheostomy - fully dependent on a caregiver for dressing and feeding - quadriplegic - not working; most likely in a long-term care facility - medical care includes medications for pain |
| ***Result*** | -0.53 ± 0.14 |

**Risk of bias**

| Subdomain and signalling question | Judgement | Notes |
| --- | --- | --- |
| **Selection of participants** | **Serious** |  |
| Was an appropriate study sample selected from the study’s sampling frame? | No | While the eligibility of the participants was suitable and appropriate, the selection |
| **Completeness of data** | **Not serious** |  |
| Was the attrition rate sufficiently low to minimise the risk of bias? | Yes | No attrition |
| **Measurement instrument** | **Not serious** |  |
| Was the instrument used to measure patient values and preferences in a valid and reliable manner? | Yes | Standard gamble of appropriately defined health states |
| Was the instrument used in the intended way? | Yes | Standard gamble of appropriately defined health states |
| Was a valid representation of the outcome (health state) used? | Yes | Each health state is appropriately and explicitly defined, and derived from the 5 clinical stages of ALS using a disease-specific health state classification system. |
| Did the researchers check for understanding of the instrument?* | Yes | The rating scale was presented as an introductory exercise to familiarise the respondents  with the objective of the interview and the notion  of preferences. |
| **Data Analysis** | **Not serious** |  |
| Were the results analysed appropriately to avoid influence of bias and confounding? | Yes | Health state utilities have been provided as simple means and SEM. |
| **Overall risk of bias** | **Serious** |  |

**Adelman 2004**

**Data extraction**

|  | Study ID | Adelman 2004 |
| --- | --- | --- |
|  | **DESIGN** | |
|  | **Other reports of this study identified?** | NR |
|  | **Time period study was conducted** | No explicit time period provided. Authors state that the final visit was July 2003. |
|  | **Design** | Pre-post with structured interviews and follow-up in patient homes. Trained clinical interviewers visited caregivers and patients once a month, starting at the time of enrolment. These visits continued until the patient died or underwent a tracheostomy. In the latter case, follow-up continued but switched from once a month to every 3 months. For patients who died, caregivers were interviewed once after the death to obtain information about the end of life.  Patients were asked to assess mood and attitudes; a series of Visual Analogue Scale (VAS) ratings were elicited from caregivers and patients. Patients were asked to rate current pain, energy, suffering, depression, control over ALS management, optimism, interest in hastening death, weariness, desire to live, and how burdened they thought their caregivers were.  Caregivers provided a strictly parallel rating of patients’ current pain, energy, suffering, depression, control over ALS management, optimism, interest in hastening death, weariness, and desire to live. In addition, caregivers rated how burdened they were on the same VAS. |
|  | **Outcomes with a valuation provided relevant to the review** | The only outcomes in which a valuation was provided that is relevant to this review include:  1) How interested is patient in hastening death  2) How much does patient want to live? |
|  | **SETTING** | |
|  | **Country** | United States |
|  | **Sites/s** | New York City |
|  | **Study locale** | Nothing is made explicit in text, but the authors state “All potential participants were first approached by clinicians at our ALS Center…” The authors are affiliated with the Eleanor and Lou Gehrig ALS Center and Columbia University Medical Center. |
|  | **Disease investigated** | Amyotrophic lateral sclerosis |
|  | **PARTICPANTS** | |
|  | **Category of participants under investigation** | Adults with ALS and their caregivers |
|  | **Eligibility criteria of participants** | Patient– caregiver pairs were eligible to participate in the study if the patient met El Escorial criteria for definite or probable ALS and had recently developed a forced vital capacity (FVC) of 50% of predicted value, based on gender, stature, and age norms. Patients were also required to be tracheostomy-free at the time of study enrolment and to have unpaid family or friend caregivers willing to participate.  Four percent of potential participants were excluded because of dementia, based on judgment of the referring clinician prior to  enrolment. Non-English-speaking patients were also excluded,  along with patients who lived 3 hours’ drive from New York City. |
|  | **Sampling strategy** | Purposive sampling  All potential participants were first approached by clinicians at the site or by clinicians from other centers who explained the study and invited eligible families to participate. If families expressed interest, they were called by the principal investigator of the study. During this call, the consent form was reviewed, and patients and caregivers were free to ask questions. If families showed interest, their name was passed on to an interviewer, who scheduled an appointment. |
|  | **Number of participants included** | ALS patients n = 69  Caregivers n = 69 |
|  | **Characteristics/demographics of participants included** | Demographics \| Caregivers (n = 69) \| Patients (n = 69); Age, y \| 58.1 ± 14.3 (26–90) \| 61.9 ± 14.9 (27–85); Male, n (%) \| 17 (25) \| 39 (57); White, n (%) \| 62 (90) \| 60 (87); **Education, n (%)** \| \|; Graduate degree \| 21 (30) \| 14 (20); College degree \| 14 (20) \| 16 (23); Some college \| 9 (13) \| 6 (9); High school degree \| 18 (26) \| 27 (40); Did not complete high school \| 7 (10) \| 6 (9); **Income, n (%)** \| \|; Less than $20,000 \| 11 (16) \|; $20,001–$60,000 \| 22 (32) \|; $60,001–$100,000 \| 14 (20) \|; Greater than $100,001 \| 11 (16) \|; Not answered \| 11 (16) \|; **Relationship status** \| \|; Spousal relationship, n (%) \| 42 (62) \|; Years together \| 32 ± 17.9 (3–62) \|; **Employment status, n (%)** \| \|; Working at first visit \| 34 (49) \| 6 (9); Not working \| 35 (51) \| 63 (91) |
|  | **Response rate** | Purposive sampling for maximum completion.  However, by end of follow-up, 43 patients (62%) that had started interviews had died. However, 54 of 69 pairs had two or more study assessments. |
|  | **OUTCOME 1: HOW INTERESTED IS THE PATIENT IN HASTENING DEATH?** | |
|  | **Instrument or method** | Structured interview with VAS. Scales ranged from 1 to 10. A value of “1” represented least intensity and “10” greatest intensity. |
|  | **Preference set used in translation of raw results** | NA |
|  | **Reported format** | Mean and standard deviation |
|  | **Result** | First assessment (all pairs)  Patient: 3.2 ± 3.3  Caregiver: 2.8 ± 2.7  First assessment (only patient-spouse pairs)  Patient: 3.5 ± 3.4  Caregiver: 2.7 ± 2.7  Last assessment (all pairs)  Patient: 3.1 ± 3.3  Caregiver: 2.7 ± 2.8  Last assessment (only patient-spouse pairs)  Patient: 3.3 ± 3.4  Caregiver: 2.8 ± 3.0 |
| **OUTCOME 2: HOW MUCH DOES PATIENT WANT TO LIVE?** | | |
| **Instrument or method** | | Structured interview with VAS. Scales ranged from 1 to 10. A value of “1” represented least intensity and “10” greatest intensity. |
| **Preference set used in translation of raw results** | | NA |
| **Reported format** | | Mean and standard deviation |
| **Result** | | First assessment (all pairs)  Patient: 7.8 ± 3.1  Caregiver: 8.0 ± 3.0  First assessment (only patient-spouse pairs)  Patient: 7.8 ± 3.1  Caregiver: 7.8 3.2  Last assessment (all pairs)  Patient: 7.5 ± 3.4  Caregiver: 6.9 ± 3.3  Last assessment (only patient-spouse pairs)  Patient: 7.4 ± 3.5  Caregiver: 7.2 ± 3.2 |

**Risk of bias**

| Subdomain and signalling question | Judgement | Notes |
| --- | --- | --- |
| **Selection of participants** | **Not serious** |  |
| Was an appropriate study sample selected from the study’s sampling frame? | Probably yes | The authors have utilised a purposive sample strategy, whereby patients were approached by clinicians at an ALS centre. It was not clear if patients were sampled consecutive or following a specific selection rule. However, the purpose of this study was to evaluate agreement between ALS patients and their caregivers, and not for this sample to serve as representative of a broader population of people with ALS or their caregivers. Therefore, a judgement of probably yes is appropriate. |
| **Completeness of data** | **Not serious** |  |
| Was the attrition rate sufficiently low to minimise the risk of bias? | Yes | 54 of 69 pairs were seen twice. The mean number of days between the first and last assessment was 104 days (ranged from 35 to 846). |
| **Measurement instrument** | **Very serious** |  |
| Was the instrument used to measure patient values and preferences in a valid and reliable manner? | Yes | A structured interview with a VAS was utilised in this study. Scales ranged from 1 to 10. A value of “1” represented least intensity and “10” greatest intensity. |
| Was the instrument used in the intended way? | Probably yes | Ideally, a statement would have been provided by the authors as to who (of the research team) administered the interviews or who organised the augmentative communication devices. However, none of this was provided. A judgement of probably yes is appropriate given it was a structured interview, and all the questions asked were identical for each participant. |
| Was a valid representation of the outcome (health state) used? | No | This study has provided an indirect measure of a health state. That being a response to two specific questions in a structured interview. As such, there have not been specific, high-quality descriptions provided to describe the health state. As per the guidance, wrong or insufficient information as to the health state may mislead participant and bias the measurement. As no description was provided as to what the health states were, a judgement of no is appropriate. |
| Did the researchers check for understanding of the instrument? | Investigators did not formally test the understanding, but evidence suggested adequate understanding | There is no statement from the authors that the participants understood (or were asked if they understood) the questions that were asked of them in the structured interview. |
| **Data Analysis** | **Extremely serious** |  |
| Were the results analysed appropriately to avoid influence of bias and confounding? | No | Only simple means and standard deviations have been presented, no adjustments for potential or actual confounders have been made. |
| **Overall risk of bias** | **Extremely Serious** |  |

**Akerblom 2021**

**Data extraction**

| Study ID | Akerblom 2014 |
| --- | --- |
| **DESIGN** | |
| **Other reports of this study identified?** | None |
| **Time period study was conducted** | September 2015 to September 2016 |
| **Design** | Cross-sectional study - Individual quality of life was measured with a study-specific version of the Schedule for the Evaluation of  Individual Quality of Life - Direct Weighting. The design was correlational using interview and survey data from the first point of measurement in a prospective study with all in all five measurement occasions. |
| **Outcomes with a valuation provided relevant to the review** | The only outcomes with a valuation that are relevant to this review come from the IQOL frequency of nominations. The following ‘areas’ are relevant: Being independent  Being able to work  Being physically active  Being in good health  Being able to travel  Hope for the future |
| **SETTING** | |
| **Country** | Sweden |
| **Sites/s** | Uppsala, four multidisciplinary MND teams (sites not specified). |
| **Study locale** | Outpatient care (clinic visits) |
| **Disease investigated** | Motor Neuron Disease (including ALS, MND with lower motor neuron signs, and PLS) |
| **PARTICPANTS** | |
| **Category of participants under investigation** | Adults with MND |
| **Eligibility criteria of participants** | Patients, who had a scheduled visit to any of the MND teams during the recruitment period. Diagnosis of MND, aged over 18. Excluded if Kennedy’s disease, cognitive impairment, language barriers, or other neurological conditions affecting symptoms. |
| **Sampling strategy** | Purposive sampling Participants were approached during scheduled clinical visits by team coordinators or data collectors; recruitment based on eligibility during routine appointments. |
| **Number of participants included** | 61 participants (55 included in main analysis) |
| **Characteristics/demographics of participants included** | Patients characteristics, n \| All patients, 61; Gender, male/female n (%) \| 39/22 (64/36); Age, all patients, m (SD) \| 61.9 (12.3); Family situation, n (%) \|; Married/cohabitant \| 35 (57); Partner and children \| 11 (18); Single parent \| 3 (5); Single \| 12 (20); Education, n (%) \|; Elementary school \| 15 (25); High school \| 24 (39); University \| 22 (36); Occupational status, n (%) \|; Working full-time \| 6 (10); Working part-time \| 5 (8); Sickness benefits fulltime \| 17 (28); Retired \| 31 (51); Unemployed \| 0 (0); Other \| 2 (3); Diagnosis, n (%) \|; ALS \| 31 (51); MND \| 23 (38); PLS \| 7 (12); Time since first symptom of disease, m year (sd) \| 5.8 (7.2); Pain, BPI-SF, n = 55 \|; Pain, n = yes (%) \| 41 (74); Pain during past 24 h, n = yes (%) \| 39 (71); Worst level of pain, md (IQR) \| 5.0 (4.5); PSI, n = 41, m (sd) \| 3.8 (2.4); Neuropathic pain, n (%), DN4 \| 12 (20); Chronic pain before onset of MND, n (%) \| 22 (36); Disease severity, ALSFRS-R, md (Q1–Q3) \|; The bulbar function \| 10 (7.5–12); The fine motor function \| 8 (4–10); The gross motor function \| 7 (5–9); The respiratory function \| 12 (10–12); Drugs, n (%) \|; Analgesics \| 6 (10); Antidepressants \| 17 (28); Antiepileptics \| 2 (3); Anxiolytics \| 9 (15); Hypnotics \| 16 (26); NSAID \| 8 (13); Opioids \| 6 (10); Spasmolytics \| 6 (10); Triptans \| 2 (3); Riluzole \| 55 (90); Nothing \| 2 (3) |
| **Response rate** | 95 eligible; 61 participated (~64%) |
| **OUTCOME 1: BEING INDEPENDENT** | |
| **Instrument or method** | Individual quality of life (IQOL) The instrument is based on a semi-structured interview and started with an open question; “If you think about your whole life situation as it is right now, what are the most important areas, both good and bad, that are vital for your quality of life?”. Thereafter, participants were asked to: 1. identify the most important areas of quality of life, and describe the meaning of each area, 2. select five of the areas, which currently are the most important, 3. rate how satisfied you are with respect to each of the five areas frequency of nominations among those with pain/without pain. What is presented below is the response to point 2, being the frequency that these items were picked (indicating most important, and therefore, outcome valuation). |
| **Preference set used in translation of raw results** | Not Applicable |
| **Reported format** | N (%) |
| **Result** | With pain: 9 (22)  Without pain: 4 (28) |
| **OUTCOME 2: BEING ABLE TO WORK** | |
| **Instrument or method** | See above |
| **Preference set used in translation of raw results** | Not Applicable |
| **Reported format** | N (%) |
| **Result** | With pain: 9 (22)  Without pain: 2 (14) |
| **OUTCOME 3: BEING PHYSICALLY ACTIVE** | |
| **Instrument or method** | See above |
| **Preference set used in translation of raw results** | Not Applicable |
| **Reported format** | N (%) |
| **Result** | With pain: 9 (22)  Without pain: 2 (14) |
| **OUTCOME 4: BEING IN GOOD HEALTH** | |
| **Instrument or method** | See above |
| **Preference set used in translation of raw results** | Not Applicable |
| **Reported format** | N (%) |
| **Result** | With pain: 3 (7)  Without pain: 4 (28) |
| **OUTCOME 5: BEING ABLE TO TRAVEL** | |
| **Instrument or method** | See above |
| **Preference set used in translation of raw results** | Not Applicable |
| **Reported format** | N (%) |
| **Result** | With pain: 4 (10)  Without pain: 1 (7) |
| **OUTCOME 6: HOPE FOR THE FUTURE** | |
| **Instrument or method** | See above |
| **Preference set used in translation of raw results** | Not Applicable |
| **Reported format** | N (%) |
| **Result** | With pain: 2 (5)  Without pain: 0 (0) |

**Risk of bias**

| Subdomain and signalling question | Judgement | Notes |
| --- | --- | --- |
| **Selection of participants** | serious |  |
| Was an appropriate study sample selected from the study’s sampling frame? | Probably no | The authors have employed a purposive sampling strategy. Patients were recruited from four multidisciplinary MND teams in Sweden from September 2015 to September 2016. Patients, who had a scheduled visit to any of the MND teams during the recruitment period, had an MND diagnosis and were over 18 years old were asked to participate in the study. Only 61 of 95 eligible patients participated in the study. There are still latent concerns of selection bias, hence probably no and serious risk of bias designation. |
| **Completeness of data** | Not serious |  |
| Was the attrition rate sufficiently low to minimise the risk of bias? | Yes | Of the 61 participants that agreed to take the survey, only 6 did not complete. |
| **Measurement instrument** | Extremely serious |  |
| Was the instrument used to measure patient values and preferences in a valid and reliable manner? | Probably no | The IOQL used in this study is a study specific version of the SEIQoL-DW. Which is a validated and reliable measure for determining health states. The authors of this study explicitly state that the weighing procedure (of the standard SEIQoL) was omitted in this version. |
| Was the instrument used in the intended way? | No | The authors have used an adapted version of the SEIQoL-DW for this study. |
| Was a valid representation of the outcome (health state) used? | Probably no | The authors do state that patients were asked to list the areas that were of most important, they then state that these items were subjected to qualitative content analysis, which suggests that participant responses were categorised based on researcher interpretation to the ‘outcomes’ reported, and a consistent, specific representation of the outcome is now lost due to this interpretation. |
| Did the researchers check for understanding of the instrument? | Investigators did not formally test the understanding, but evidence suggested adequate understanding |  |
| **Data Analysis** | Extremely serious |  |
| Were the results analysed appropriately to avoid influence of bias and confounding? | No | Simples counts and percentages have been reported. |
| **Overall risk of bias** | **Extremely serious** |  |

**Conroy 2023**

**Data extraction**

| Study ID | Conroy 2023 |
| --- | --- |
| **DESIGN** | |
| **Other reports of this study identified?** | None |
| **Time period study was conducted** | October to December 2020 |
| **Design** | Cross-sectional survey (with direct outcome valuation). The IMPACT-ALS survey (36 questions with follow-up questions). The questions of the survey relevant to this review are related to “Approaches to treating ALS/MND” which enquired about medications and other treatment strategies (e.g. gastrostomy, non-invasive ventilation, exercise, and mobility aids), functional independence, lifestyle changes, motives for involvement in research, and wishes for future treatments. |
| **Outcomes with a valuation provided relevant to the review** | Symptoms or functional impairment of ALS on which they most wanted current treatments to have a beneficial impact   - Stop the progression of ALS/MND - Breathing/respiratory function - Mobility - Speaking/Communication - Daily self-care - Overall physical function - Muscle weakness - Ease the overall burden of ALS/MND - Fatigue - Swallowing - Cognitive ability-focus and thinking - Overall mental health - Ability to express myself in my relationships - Loss of interest (apathy) - Muscle spasticity - Pain - Laughing/crying uncontrollably - Sexual function   Most important outcomes of a new drug   - The drug prolongs survival but has moderate to severe side effects that some people cannot tolerate - The drug slows (does not halt) the spread of ALS/MND and has minimal-moderate side effects - The drug significantly reduces some symptoms from ALS/MND with side-effects that I can manage but does not affect*… (this is cutoff on the paper, and the full option is not reported. Contacted authors).* - The drug prevents further muscle weakening but does not improve strength - The drug increases muscle strength, even if it has moderate to severe side effects that are difficult to manage. |
| **SETTING** | |
| **Country** | Occurred over nine European countries, Italy, France, Netherlands, Spain, Ireland, UK, Belgium, Germany, Sweden |
| **Sites/s** | Not reported |
| **Study locale** | This was an online survey (conducted during COVID-19 pandemic) |
| **Disease investigated** | ALS |
| **PARTICPANTS** | |
| **Category of participants under investigation** | Adults diagnosed with MND |
| **Eligibility criteria of participants** | Self-identified people living with ALS, this was an anonymous survey with online participation. All people living with ALS were eligible to participate without any specific exclusion criteria. |
| **Sampling strategy** | Convenience sampling, the authors mention publicising the survey link within ENCALS and local advocacy groups and ALS clinics. |
| **Number of participants included** | 857 responses from people living with ALS. |
| **Characteristics/demographics of participants included** | (\| Total responding \| n (%); Country of residence \| 506 \|; Belgium \| \| 16 (3.2); France \| \| 75 (14.8); Germany \| \| 11 (2.2); Ireland \| \| 24 (4.7); Italy \| \| 254 (50.2); Netherlands Spam W 6.9) \| \| 63 (12.5); Sweden \| \| 11 (2.2); UK \| \| 22 (4.3); Sex \| 506 \|; Female \| \| 217 (42.9); Male \| \| 289 (57.1); Age (years) \| 506 \|; 2-4 \| \| 5 (1.0); 35-44 \| \| 41 (8.1); 45-54 \| \| 88 (17.4); 55-64 \| \| 183 (36.2); 65-74 \| \| 145 (28.7); 75+ \| \| 44 (8.7); Highest level of education completed \| 506 \|; Primary school \| \| 20 (4.0); Lower secondary school \| \| 71 (14); Upper secondary school \| \| 110 (21.7); ‘Technical or vocational \| \| 88 (17.4); Advanced or higher certificate/completed apprenticeship \| \| 50 (9.9); Ordinary bachelor’s degree or national diploma \| \| 59 (11.7); Honours bachelor’s degree/professional qualification \| \| 40 (7.9); Postgraduate diploma/ Master's degree \| \| 56 (11.1); Doctorate (PhD) \| \| 12 (2.4); Marital status \| 506 \|; Single \| \| 39 (7.6); Separated \| \| 15 (2.9); Married \| \| 388 (75.2); Divorced \| \| 21 (4.1); Living with partner \| \| 32 (6.2); Widowed \| \| 21 (4.1); Living arrangement \| 530 \|; Alone \| \| 40 (7.5); Live with siblings \| \| 6 (1.1); Live with spouse/partner \| \| 249 (47); Live with parents \| \| 17 (3.2); Live with spouse/partner and children \| \| 164 (30.9); Live with children \| \| 20 (3.8); Live with other \| \| 34 (6.4); Employment \| 506 \|; Full-time \| \| 69 (13.6); Part-time \| \| 31 (6.1); Retired \| \| 222 (43.9); On disability \| \| 115 (22.7); Unemployed \| \| 11 (2.2); Other, please specify \| \| 58 (11.5); Private health insurance \| 506 \|; Yes \| \| 169 (33.4); No \| \| 337 (66.6); Private health insurance by country \| \|; Belgium \| \| 14 (87.5); France \| \| 41 (54.7); Germany \| \| 1 (9.1); Ireland \| \| 18 (75); Italy \| \| 24 (9.4); Netherlands \| \| 52 (82.5); Spain \| \| 14 (46.7); Sweden \| \| 1 (9.1); UK \| \| 4 (18.2)) |
| **Response rate** | Not calculable, number of people invited to the survey is unknown (open call). The authors have reported that 506 of 857 respondents ranked the outcomes of a new drug from important to least important. |
| **OUTCOME 1: STOP THE PROGRESSION OF MND** | |
| **Instrument or method** | People were asked to identify what they wanted their current ALS treatments to impact. |
| **Preference set used in translation of raw results** | Not Applicable |
| **Reported format** | Number of respondents |
| **Result** | 161 (total not specified) |
| **OUTCOME 2: BREATHING/RSPIRATORY FUNCTION** | |
| **Instrument or method** | See outcome 1 |
| **Preference set used in translation of raw results** | Not Applicable |
| **Reported format** | Number of respondents |
| **Result** | 90 (total not specified) |
| **OUTCOME 3: MOBILITY** | |
| **Instrument or method** | See outcome 1 |
| **Preference set used in translation of raw results** | Not Applicable |
| **Reported format** | Number of respondents |
| **Result** | 49 (total not specified) |
| **OUTCOME 4: SPEAKING/COMMUNICATION** | |
| **Instrument or method** | See outcome 1 |
| **Preference set used in translation of raw results** | Not Applicable |
| **Reported format** | Number of respondents |
| **Result** | 33 (total not specified) |
| **OUTCOME 5: DAILY SELF-CARE** | |
| **Instrument or method** | See outcome 1 |
| **Preference set used in translation of raw results** | Not Applicable |
| **Reported format** | Number of respondents |
| **Result** | 28 (total not specified) |
| **OUTCOME 6: OVERALL PHYSICAL FUNCTION** | |
| **Instrument or method** | See outcome 1 |
| **Preference set used in translation of raw results** | Not Applicable |
| **Reported format** | Number of respondents |
| **Result** | 25 (total not specified) |
| **OUTCOME 7: MUSCLE WEAKNESS** | |
| **Instrument or method** | See outcome 1 |
| **Preference set used in translation of raw results** | Not Applicable |
| **Reported format** | Number of respondents |
| **Result** | 23 (total not specified) |
| **OUTCOME 8: EASE THE OVERALL BURDEN OF ALS/MND** | |
| **Instrument or method** | See outcome 1 |
| **Preference set used in translation of raw results** | Not Applicable |
| **Reported format** | Number of respondents |
| **Result** | 20 (total not specified) |
| **OUTCOME 9: FATIGUE** | |
| **Instrument or method** | See outcome 1 |
| **Preference set used in translation of raw results** | Not Applicable |
| **Reported format** | Number of respondents |
| **Result** | 15 (total not specified) |
| **OUTCOME 10: SWALLOWING** | |
| **Instrument or method** | See outcome 1 |
| **Preference set used in translation of raw results** | Not Applicable |
| **Reported format** | Number of respondents |
| **Result** | 7 (total not specified) |
| **OUTCOME 11: COGNITIVE ABILITY-FOCUS AND THINKING** | |
| **Instrument or method** | See outcome 1 |
| **Preference set used in translation of raw results** | Not Applicable |
| **Reported format** | Number of respondents |
| **Result** | 6 (total not specified) |
| **OUTCOME 12: OVERALL MENTAL HEALTH** | |
| **Instrument or method** | See outcome 1 |
| **Preference set used in translation of raw results** | Not Applicable |
| **Reported format** | Number of respondents |
| **Result** | 4 (total not specified) |
| **OUTCOME 13: ABILITY TO EXPRESS MYSELF IN RELATIONSHIP** | |
| **Instrument or method** | See outcome 1 |
| **Preference set used in translation of raw results** | Not Applicable |
| **Reported format** | Number of respondents |
| **Result** | 3 (total not specified) |
| **OUTCOME 14: LOSS OF INTEREST (APATHY)** | |
| **Instrument or method** | See outcome 1 |
| **Preference set used in translation of raw results** | Not Applicable |
| **Reported format** | Number of respondents |
| **Result** | 3 (total not specified) |
| **OUTCOME 15: MUSCLE SPASTICITY** | |
| **Instrument or method** | See outcome 1 |
| **Preference set used in translation of raw results** | Not Applicable |
| **Reported format** | Number of respondents |
| **Result** | 2 (total not specified) |
| **OUTCOME 16: PAIN** | |
| **Instrument or method** | See outcome 1 |
| **Preference set used in translation of raw results** | Not Applicable |
| **Reported format** | Number of respondents |
| **Result** | 1 (total not specified) |
| **OUTCOME 17: LAUGHING/CRYING UNCONTROLLABLY** | |
| **Instrument or method** | See outcome 1 |
| **Preference set used in translation of raw results** | Not Applicable |
| **Reported format** | Number of respondents |
| **Result** | 1 (total not specified) |
| **OUTCOME 18: SEXUAL FUNCTION** | |
| **Instrument or method** | See outcome 1 |
| **Preference set used in translation of raw results** | Not Applicable |
| **Reported format** | Number of respondents |
| **Result** | 1 (total not specified) |
| **OUTCOME 19: THE DRUG PROLONGS SURVIVAL BUT HAS MODERATE TO SEVERE SIDE EFFECTS THAT SOME PEOPLE CANNOT TOLERATE** | |
| **Instrument or method** | In the survey, participants ranked outcomes of a new drug (hypothetical) from most important to least important. |
| **Preference set used in translation of raw results** | Not Applicable |
| **Reported format** | The percentage of respondents that indicated that this outcome was their most important |
| **Result** | 8.5% |
| **OUTCOME 20: THE DRUG SLOWS (DOES NOT HALT) THE SPREAD OF ALS/MND AND HAS MINIMAL-MODERATE SIDE EFFECTS** | |
| **Instrument or method** | See Outcome 19 |
| **Preference set used in translation of raw results** | Not Applicable |
| **Reported format** | The percentage of respondents that indicated that this outcome was their most important |
| **Result** | 28.9% |
| **OUTCOME 21: THE DRUG SIGNIFICANTLY REDUCE SOME SYMPTOMS FROM ALS/MND WITH SIDE-EFFECTS THAT I CAN MANAGE BUT DOES NOT AFFECT…(THIS IS CUTOFF ON THE PAPER, AND THE FULL OPTION IS NOT REPORTED. CONTACTED AUTHORS).** | |
| **Instrument or method** | See Outcome 19 |
| **Preference set used in translation of raw results** | Not Applicable |
| **Reported format** | The percentage of respondents that indicated that this outcome was their most important |
| **Result** | 14.2% |
| **OUTCOME 22: THE DRUG PREVENTS FURTHER MUSCLE WEAKENING BUT DOES NOT IMPROVE STRENGTH** | |
| **Instrument or method** | See Outcome 19 |
| **Preference set used in translation of raw results** | Not Applicable |
| **Reported format** | The percentage of respondents that indicated that this outcome was their most important |
| **Result** | 24.5% |
| **OUTCOME 23: THE DRUG INCREASES MUSCLE STRENGTH, EVEN IF IT HAS MODERATE TO SEVERE SIDE EFFECTS THAT ARE DIFFICULT TO MANAGE.** | |
| **Instrument or method** | See Outcome 19 |
| **Preference set used in translation of raw results** | Not Applicable |
| **Reported format** | The percentage of respondents that indicated that this outcome was their most important |
| **Result** | 23.9% |

**Risk of bias**

| Subdomain and signalling question | Judgement | Notes |
| --- | --- | --- |
| **Selection of participants** | Not serious |  |
| Was an appropriate study sample selected from the study’s sampling frame? | Probably yes | Online survey reports data from 857 plwALS. Recruitment of plwALS and survey distribution occurred through established networks within ENCALS and local advocacy groups. However, due to the survey being conducted online and anonymously, there is potential (albeit unlikely) that the intended recipient did not complete the survey as there was no verification of ALS diagnosis built into the survey. |
| **Completeness of data** | Extremely serious |  |
| Was the attrition rate sufficiently low to minimise the risk of bias? | No | As this was an online survey with an open call this not determinable. However, of the 857 respondents, only 506 of them provided responses regarding outcome valuation. (59%). |
| **Measurement instrument** | Extremely serious |  |
| Was the instrument used to measure patient values and preferences in a valid and reliable manner? | No | This was a simple survey designed specifically for this study. As such, it was not designed to directly measure patient values and preferences of their outcomes. |
| Was the instrument used in the intended way? | Yes | This was a simple online, anonymous survey. |
| Was a valid representation of the outcome (health state) used? | Probably yes | The authors do state that the questions were piloted-tested before initiation of the survey and the definitions used regarding outcomes of a new drug are descriptive, and highly specific. |
| Did the researchers check for understanding of the instrument? | Investigators did not formally test the understanding, but evidence suggested adequate understanding | However, as pilot-testing it is assumed that understanding was tested, despite it not being stated specifically. |
| **Data Analysis** | Extremely serious |  |
| Were the results analysed appropriately to avoid influence of bias and confounding? | No | Simples counts and percentages have been reported. No adjustments have been made |
| **Overall risk of bias** | **Extremely serious** |  |

**Gebrehiwet 2023**

**Data extraction**

| Study ID | Gebrehiwet 2023 |
| --- | --- |
| **DESIGN** | |
| **Other reports of this study identified?** | None |
| **Time period study was conducted** | Not reported |
| **Design** | Direct utility and health state value study, derived from a patient population that participated in the FORTITUDE-ALS clinical trial. Patients completed the ALSFRS-R at baseline and then during set intervals over the course of the trial. The results of the ALSFRS-R were then mapped to the EQ-5D using a published mapping algorithm. |
| **Outcomes with a valuation provided relevant to the review** | Health-related quality of life (EQ-5D-5L)*  *Estimated from the ALSFRS-R that was administered during the trial.  QALYs |
| **SETTING** | |
| **Country** | United States, Australia, Canada, Ireland, Netherlands, Spain |
| **Sites/s** | The FORTITUDE-ALS clinical trial was a multi-centre trial (from clinical trials registration) that occurred across multiple sites from the countries above. |
| **Study locale** | Outpatient clinical trial setting |
| **Disease investigated** | ALS |
| **PARTICPANTS** | |
| **Category of participants under investigation** | Adults diagnosed with MND |
| **Eligibility criteria of participants** | Adults diagnosed with ALS who met the FORTITUDE-ALS trial criteria. Must have received at least one dose of study drug and have ALSFRS-R assessment at baseline and one post-baseline visit. |
| **Sampling strategy** | The sampling strategy for the original clinical trial was through randomisation with 1:1:1:1 allocation (3 different reldesemtiv doses and placebo). |
| **Number of participants included** | 456 total  Reldesemtiv n = 342  Placebo n =114 |
| **Characteristics/demographics of participants included** | Characteristic \| Reldesemtiv (n = 342) \| Placebo (n = 114); Age, years, mean (SD) \| 58.3 (10.8) \| 59.7 (10.6); Male, n (%) \| 209 (61.1) \| 67 (58.8); White, n (%) \| 316 (92.4) \| 106 (93.0); BMI, kg/m², mean (SD) \| 26.7 (4.7) \| 26.1 (4.4); ALSFRS-R total score, mean (SD) \| 37.5 (5.5) \| 37.0 (5.6); SVC, % predicted, mean (SD) \| 84.6 (15.5) \| 84.9 (14.8); Time since diagnosis, months, mean (SD) \| 8.5 (6.0) \| 8.8 (6.4); Time since 1st symptom, months, mean (SD) \| 23.0 (20.9) \| 22.2 (12.4); ALS site of onset: bulbar, n (%) \| 65 (19.0) \| 22 (19.3); On riluzole alone, n (%) \| 194 (56.7) \| 63 (55.3); On edaravone alone, n (%) \| 14 (4.1) \| 5 (4.4); On riluzole plus edaravone, n (%) \| 70 (20.5) \| 24 (21.1) |
| **Response rate** | Reldesemtiv Placebo  Baseline 342 114  Week 2 339 113  Week 4 331 107  Week 8 314 104  Week 12 305 100 |
| **OUTCOME 1: HEALTH-RELATED QUALITY OF LIFE (EQ-5D-5L)*** | |
| **Instrument or method** | Health utilities from EQ-5D-5L were calculated for baseline and weeks 2, 4, 8, and 12. As EQ-5D-5L was not administered in FORTITUDE-ALS, EQ-5D-5L utility was estimated from the ALSFRS-R using a published mapping algorithm. |
| **Preference set used in translation of raw results** | UK general population (according to reference #9 in the paper) |
| **Reported format** | Mean (SD) |
| **Result** | Baseline: 0.67 (0.177)  Week 2: 0.66 (0.182)  Week 4: 0.66 (0.186)  Week 8: 0.64 (0.191)  Week 12: 0.63 (0.190) |
| **OUTCOME 2: QALYS** | |
| **Instrument or method** | QALYs were calculated using the area under the curve, using the baseline, week 2, 4, 8, and 12 EQ-5D-5L utilities |
| **Preference set used in translation of raw results** | Not Applicable |
| **Reported format** | Mean (SD) |
| **Result** | 0.1639 (0.045) |

**Risk of bias**

| Subdomain and signalling question | Judgement | Notes |
| --- | --- | --- |
| **Selection of participants** | Not serious |  |
| Was an appropriate study sample selected from the study’s sampling frame? | Yes | The participants in this study were derived from participants that were randomised to groups in a randomised controlled trial. |
| **Completeness of data** | Extremely serious risk of bias |  |
| Was the attrition rate sufficiently low to minimise the risk of bias? | No | By week 12, only 37 of 342 (10%) participants in the intervention arm did not complete the ALSFRS-R (and therefore the EQ-5D). And only 14 of 114 (12%) of participants in the control arm did not complete the ALSFRS-R. |
| **Measurement instrument** | Not serious |  |
| Was the instrument used to measure patient values and preferences in a valid and reliable manner? | Yes | The participants in the RCT did not complete the EQ-5D themselves. They completed the ALSFRS-R and had their responses mapped to the EQ-5D, following a published algorithm. Given this algorithm has been peer-reviewed and seems appropriate judgement of yes appears relevant |
| Was the instrument used in the intended way? | Yes | As above, the respondents completed the ALSFRS-R, however these responses were mapped according to the published algorithm to the EQ-5D. |
| Was a valid representation of the outcome (health state) used? | Yes | Technically, no representation of the outcome was presented, as the participants completed the ALSFRS, which was then mapped to the EQ-%D. But given this algorithm is peer-reviewed and seems appropriate, a score of yes seems appropriate. |
| Did the researchers check for understanding of the instrument? | Investigators did not formally test the understanding, but evidence suggested adequate understanding |  |
| **Data Analysis** | Extremely serious |  |
| Were the results analysed appropriately to avoid influence of bias and confounding? | No | Simples means and standard deviations have been presented. |
| **Overall risk of bias** | **Extremely** |  |

**Green 2003**

**Data extraction**

| Study ID | Green 2003 |
| --- | --- |
| **DESIGN** | |
| **Other reports of this study identified?** | Kiebert 2001 (merged) |
| **Time period study was conducted** | April – June 2000 |
| **Design** | Cross-sectional study Patients conducted a structured interview that consisted of the completion of three different tasks in a predefined sequence. Patients were asked to complete (in this order) the ALS Assessment Questionnaire (ALSAQ- 40), the EuroQol EQ-5D questionnaire and a standard gamble (SG) health state valuation exercise |
| **Outcomes with a valuation provided relevant to the review** | Standard Gamble (Stay in current health state for two years)  Health-related quality of life (EQ-5D VAS)  Health-related quality of life (EQ-5D DSI) |
| **SETTING** | |
| **Country** | United Kingdom |
| **Sites/s** | King’s MND Care and Research Centre, London  Royal Preston Hospital, Preston |
| **Study locale** | Outpatient clinics and patient homes |
| **Disease investigated** | ALS but MND more broadly |
| **PARTICPANTS** | |
| **Category of participants under investigation** | Adults diagnosed with MND |
| **Eligibility criteria of participants** | Patients with serious comorbidities (e.g. cancer, multiple sclerosis) were excluded from participation. The only other eligibility criteria were the ability to read and understand English. |
| **Sampling strategy** | Purposive sampling. Patients were invited to participate in the study by letter or directly by a member of the staff in the centre. Patients were categorised and selected by the clinical tam from the hospital database, based on their level of disease severity. |
| **Number of participants included** | N = 77 |
| **Characteristics/demographics of participants included** | Age, years mean (SD) \| 58.1 (12.1); Range \| 27–79; Gender, n (%) \|; Male \| 49 (64%); Female \| 28 (36%); Time since diagnosis, months \|; Mean (SD) \| 25.3 (22.6); Range \| 1–112; Time from onset of disease, months \|; Mean (SD) \| 43.6 (26.9); Range \| 7–128; Disease type, n (%) \|; Familial \| 6 (8%); Sporadic \| 71 (92%); Disease onset \|; Bulbar \| 14 (18%); Limb \| 63 (82%); ALS severity level (n, %) \|; Level 1 (Mild) \| 15 (19%); Level 2 (Moderate) \| 21 (27%); Level 3 (Severe) \| 22 (29%); Level 4 (Terminal) \| 19 (25%) |
| **Response rate** | Only one patient refused the Standard Gamble and patient refused the VAS. |
| **OUTCOME 1: STANDARD GAMBLE (STAY IN CURRENT HEALTH STATE FOR TWO YEARS)** | |
| **Instrument or method** | The SG technique was used to elicit health state values for the patient’s own health state. The authors asked patients to consider the certain outcome of living for 2 years in their own present health state vs. the uncertain outcome, involving a probability (p), of  returning to full health or a probability of death. The latter alternative was presented based on a hypothetical intervention.  To assist in the presentation of the SG the authors used props, in the  form of coloured pie charts, depicting a probability wheel approach |
| **Preference set used in translation of raw results** | None (direct patient valuation) |
| **Reported format** | Mean (SD), 95% CI, Median, Range (have reported according to disease severity). |
| **Result** | \| Level 1 (Mild) N = 15 \| Level 2 (Moderate) N = 21 \| Level 3 (Severe) N = 22 \| Level 4 (Terminal) N = 19 \| Total N = 77; Mean (SD) \| 0.79 (0.15) \| 0.67 (0.24) \| 0.71 (0.28) \| 0.45 (0.35) \| 0.65 (0.29); 95% CI \| 0.71–0.88 \| 0.56–0.77 \| 0.58–0.83 \| 0.28–0.61 \| 0.58–0.71; Median \| 0.8 \| 0.75 \| 0.75 \| 0.5 \| 0.75; Range \| 0.55–1.00 \| 0.01–1.00 \| 0.05–1.00 \| 0.01–0.99 \| 0.01–1.00 |
| **OUTCOME 2: EQ5D VAS (AGGREGATE HEALTH STATE VALUATION)** | |
| **Instrument or method** | The EQ5D was used as a measure of health outcome. The EQ-5D asks respondents to complete a rating of their current HRQL on a calibrated visual analogue scale (VAS) (thermometer type), with endpoints of 100 (best imaginable health state) at the top and zero (worst imaginable health state) at the bottom. |
| **Preference set used in translation of raw results** | None (direct patient valuation) |
| **Reported format** | Mean (SD), 95% CI, Median, Range (have reported according to disease severity). |
| **Result** | \| Level 1 (Mild) N = 15 \| Level 2 (Moderate) N = 21 \| Level 3 (Severe) N = 22 \| Level 4 (Terminal) N = 19 \| Total N = 77; Mean (SD) \| 0.74 (0.19) \| 0.63 (0.17) \| 0.51 (0.18) \| 0.37 (0.18) \| 0.55 (0.22); 95% CI \| 0.64–0.85 \| 0.55–0.71 \| 0.43–0.59 \| 0.29–0.46 \| 0.50–0.60; Median \| 0.8 \| 0.6 \| 0.5 \| 0.4 \| 0.5; Range \| 0.29–0.99 \| 0.40–0.95 \| 0.30–1.00 \| 0.00–0.75 \| 0–1.00 |
| **OUTCOME 3: EQ5D DSI (AGGREGATE HEALTH STATE VALUATION)** | |
| **Instrument or method** | The EQ5D was used as a measure of health outcome. The EQ-5D provides both a compact descriptive profile and a derived single index (DSI) value that can be used in the clinical and economic evaluation of health care. The DSI values, associated with health states defined using the EQ-5D descriptive system, are based on societal valuations, and are scored from -0.57 to 1.0, where 1.0 represents the best imaginable health. |
| **Preference set used in translation of raw results** | DSI is derived from the United Kingdom general population |
| **Reported format** | Mean (SD), 95% CI, Median, Range (have reported according to disease severity). |
| **Result** | \| Level 1 (Mild) N = 15 \| Level 2 (Moderate) N = 21 \| Level 3 (Severe) N = 22 \| Level 4 (Terminal) N = 19 \| Total N = 77; Mean (SD) \| 0.63 (0.25) \| 0.56 (0.28) \| 0.27 (0.05) \| –0.01 (0.22) \| 0.35 (0.35); 95% CI \| 0.50–0.77 \| 0.43–0.68 \| 0.16–0.38 \| –0.11–0.10 \| 0.27–0.43; Median \| 0.69 \| 0.64 \| 0.2 \| 0.08 \| 0.31; Range \| –0.18–0.85 \| –0.003–1.00 \| –0.25–0.69 \| –0.43–0.27 \| –0.43–1.00 |

**Risk of bias**

| Subdomain and signalling question | Judgement | Notes |
| --- | --- | --- |
| **Selection of participants** | Not serious |  |
| Was an appropriate study sample selected from the study’s sampling frame? | Probably yes | Patients were selected from two UK MND specialist centres using clinical databases, stratified by disease severity. Sampling was not random but aimed for balanced representation across severity levels. |
| **Completeness of data** | Not serious |  |
| Was the attrition rate sufficiently low to minimise the risk of bias? | Yes | 77 patients were included. Only two patients did not complete all assessments (1 did not complete the SD and 1 did not complete the VAS). |
| **Measurement instrument** | Not serious |  |
| Was the instrument used to measure patient values and preferences in a valid and reliable manner? | Yes | The Standard Gamble and the EQ-5D are used and validated instruments to measure patient values and preferences. |
| Was the instrument used in the intended way? | Yes | The SG and the EQ-5D were administered with face-to-face interviews and supported by visual aids where appropriate. |
| Was a valid representation of the outcome (health state) used? | Yes | The health outcome under evaluation was living in their current health state for the next two years. Given this, the patients did not need specific of highly detailed descriptions of the outcomes being evaluated as this was a SG regarding their current health state, or a potential return to full health (gambled against death) |
| Did the researchers check for understanding of the instrument? | Investigators did not formally test the understanding, but evidence suggested adequate understanding | The authors engaged in a variety of illustrative lotteries to explain the SG approach, but no formal testing of understanding was reported. |
| **Data Analysis** | Not serious |  |
| Were the results analysed appropriately to avoid influence of bias and confounding? | Probably yes | The authors have reported measures of average and variance simply (i.e. no adjustments). However, they have presented this transparently across (and between disease severity groups). |
| **Overall risk of bias** | **Not serious** |  |

**Hecht 2002**

**Data extraction**

| Study ID | Hecht 2002 |
| --- | --- |
| **DESIGN** | |
| **Other reports of this study identified?** | None identified |
| **Time period study was conducted** | Not explicitly stated but participants contacted either in 1998 or who were registered in 1998 (not clear which). |
| **Design** | Standardised interview where the patients were asked in free conversation “In your personal experience, what are the worst aspects of the disease”. Afterwards, the patients weighted their answers as “most important” and “less important”. |
| **Outcomes with a valuation provided relevant to the review** | Reduction of speech  Reduction of mobility  Knowledge of the poor prognosis  Becoming dependant on other persons  Impairment of activities of daily living  Loss of writing ability  Swallowing problems  Unimpaired consciousness  Helplessness of medicine  Social isolation |
| **SETTING** | |
| **Country** | Germany |
| **Sites/s** | Not specific details, but discussed that participants were recruited from one or more ALS centres |
| **Study locale** | Likely outpatient, as no hospital or details of the home setting were provided |
| **Disease investigated** | ALS |
| **PARTICPANTS** | |
| **Category of participants under investigation** | Adults diagnosed with MND |
| **Eligibility criteria of participants** | Patients contacted (details below) needed to be able to communicate without communication aids. No other explicit eligibility criteria were mentioned. |
| **Sampling strategy** | All patients in 1998 who were registered at the local centre for neuromuscular diseases were contacted by telephone. |
| **Number of participants included** | 41 patients of 51 registered patients agreed to take part in the study. |
| **Characteristics/demographics of participants included** | No baseline characteristics table was provided but the authors have narratively reported that of the 41 patients, 27 were men and 14 were women with a mean age of 59.9 years (SD: 11.4). The ALSFRS ranged from 3 to 40 with a median of 25. |
| **Response rate** | The interview was completed by 41 patients in the first examination and by 31 patients in the second examination at 6-months follow-up. |
| **OUTCOME 1: REDUCTION OF SPEECH** | |
| **Instrument or method** | Standardised interview where the patients were asked in free conversation “In your personal experience, what are the worst aspects of the disease in your personal experience”.  What is listed here are the number of times an item was selected as the most important. |
| **Preference set used in translation of raw results** | Not applicable |
| **Reported format** | Frequency of responses and % |
| **Result** | Baseline: 12 (29.3%)  6-month follow up: 6 (19.4%) |
| **OUTCOME 2: REDUCTION OF MOBILITY** | |
| **Instrument or method** | See outcome #1 |
| **Preference set used in translation of raw results** | Not applicable |
| **Reported format** | Frequency of responses and % |
| **Result** | Baseline: 8 (19.5%)  6-month follow up: 9 (29%) |
| **OUTCOME 3: KNOWLEDGE OF THE POOR PROGNOSIS** | |
| **Instrument or method** | See outcome #1 |
| **Preference set used in translation of raw results** | Not applicable |
| **Reported format** | Frequency of responses and % |
| **Result** | Baseline: 8 (19.5%)  6-month follow up: 7 (22.6%) |
| **OUTCOME 4: BECOMING DEPENDANT ON OTHER PERSONS** | |
| **Instrument or method** | See outcome #1 |
| **Preference set used in translation of raw results** | Not applicable |
| **Reported format** | Frequency of responses and % |
| **Result** | Baseline: 5 (12.2%)  6-month follow up: 3 (9.7%) |
| **OUTCOME 5: IMPAIRMENT OF ACTIVITIES OF DAILY LIVING** | |
| **Instrument or method** | See outcome #1 |
| **Preference set used in translation of raw results** | Not applicable |
| **Reported format** | Frequency of responses and % |
| **Result** | Baseline: 4 (9.8%)  6-month follow up: 1 (3.2%) |
| **OUTCOME 6: LOSS OF WRITING ABILITY** | |
| **Instrument or method** | See outcome #1 |
| **Preference set used in translation of raw results** | Not applicable |
| **Reported format** | Frequency of responses and % |
| **Result** | Baseline: 1 (2.4%)  6-month follow up: 1 (3.2%) |
| **OUTCOME 7: SWALLOWING PROBLEMS** | |
| **Instrument or method** | See outcome #1 |
| **Preference set used in translation of raw results** | Not applicable |
| **Reported format** | Frequency of responses and % |
| **Result** | Baseline: 1 (2.4%)  6-month follow up: 1 (3.2%) |
| **OUTCOME 8: UNIMPAIRED CONSCIOUSNESS** | |
| **Instrument or method** | See outcome #1 |
| **Preference set used in translation of raw results** | Not applicable |
| **Reported format** | Frequency of responses and % |
| **Result** | Baseline: 1 (2.4%)  6-month follow up: 1 (3.2%) |
| **OUTCOME 9: HELPLESSNESS OF MEDICINE** | |
| **Instrument or method** | See outcome #1 |
| **Preference set used in translation of raw results** | Not applicable |
| **Reported format** | Frequency of responses and % |
| **Result** | Baseline: 1 (2.4%)  6-month follow up: 1 (3.2%) |
| **OUTCOME 10: SOCIAL ISOLATION** | |
| **Instrument or method** | See outcome #1 |
| **Preference set used in translation of raw results** | Not applicable |
| **Reported format** | Frequency of responses and % |
| **Result** | Baseline: 0 (0%)  6-month follow up: 2 (6.5%) |

**Risk of bias**

| Subdomain and signalling question | Judgement | Notes |
| --- | --- | --- |
| **Selection of participants** | Not serious |  |
| Was an appropriate study sample selected from the study’s sampling frame? | Probably yes | While not a random selection, the authors asked all registered patients at the local ALS clinic to participate. |
| **Completeness of data** | Not serious |  |
| Was the attrition rate sufficiently low to minimise the risk of bias? | Probably yes | All 41 patients provided baseline data, however only 31 of the original 41 provided data at the 6-month follow-up, almost a 25% drop-out rate. Given the small sample and nature of disease this seems acceptable. |
| **Measurement instrument** | Serious |  |
| Was the instrument used to measure patient values and preferences in a valid and reliable manner? | Probably no | The interview questions used to elicit outcome valuations were neither standardised nor validated. They seem appropriate for the research question but are not ideal for measuring of patient values and preferences. |
| Was the instrument used in the intended way? | Yes | Interviews were conducted by the research team. As this was a study specific questionnaire intention of use was likely achieved. |
| Was a valid representation of the outcome (health state) used? | Yes | The interviewers asked the participants for their “worst aspects of the disease”. This was based on participant interpretation of their own health states. |
| Did the researchers check for understanding of the instrument? | Investigators tested the understanding, and understanding was adequate | No mention of checks for understanding or piloting of the interview guide. |
| **Data Analysis** | Extremely serious |  |
| Were the results analysed appropriately to avoid influence of bias and confounding? | No | The results presented were descriptive and clearly reported, however no multivariable analysis or control for confounders was done. |
| **Overall risk of bias** | **Extremely serious** |  |

**Helleman 2022**

**Data extraction**

| Study ID | Helleman 2022 |
| --- | --- |
| **DESIGN** | |
| **Other reports of this study identified?** | None |
| **Time period study was conducted** | November 2020 – November 2021 |
| **Design** | Cross-sectional survey.  Developed in English and Dutch. One question provided a list of seven outcome measures for home-monitoring, of which patients had to rank a top three of the most valuable outcome measures. |
| **Outcomes with a valuation provided relevant to the review** | Most valuable outcome measure for home-monitoring:  Respiratory function  Muscle strength  Quality of life  Fine motor/speech  Daily activity  Sleep  Body weight |
| **SETTING** | |
| **Country** | The Netherlands, United Kingdom, Australia |
| **Sites/s** | Not explicitly mentioned in text. The authors state that the present study was conducted by multidisciplinary MND clinics, and the authors affiliations are as:  University Medical Centre Utrecht  Sheffield Institute for Translational Neuroscience  Australian Institute for Bioengineering and Nanotechnology  However, the sampling was through national MND registries, MND organisations and invitations sent via email. |
| **Study locale** | Community-based |
| **Disease investigated** | ALS, PLS and PMA |
| **PARTICPANTS** | |
| **Category of participants under investigation** | Adults with MND |
| **Eligibility criteria of participants** | Not explicitly mentioned, is participants were on the registries listed then they were eligible. |
| **Sampling strategy** | Netherlands – the national ALS registry was accessed and invitations sent  UK – those who attended the Sheffield MND clinic were invited  Australia – participants were from Victoria, Queensland or Western Australia and were registered on the national MND registry and previous consented to be contacted for research purposes.  Invitations were also disseminated through social media, calls for research participation online and emails. |
| **Number of participants included** | N = 332  Netherlands: 200  UK: 91  Australia: 41 |
| **Characteristics/demographics of participants included** | Characteristic \| The Netherlands (N = 200) \| United Kingdom (N = 91) \| Australia (N = 41) \| p value; Sex, male (%) \| 135 (68.2) \| 55 (61.1) \| 29 (70.7) \| 0.42; Age at enrolment, years \| 63.4 (10.2)* \| 66.8 (10.4)* \| 64.8 (7.8) \| 0.029; Symptom onset, bulbar (%) \| 34 (17.2) \| 18 (21.3) \| 4 (12.1) \| 0.44; Symptom duration, months (median [IQR]) \| 42.1 (21.7–68.5)* \| 64.1 (27.1–148.3)† \| 44.0 (24.6–77.7) \| <0.001; Diagnostic delay, months (median [IQR]) \| 13.7 (6.9–29) \| 17.5 (8.4–29.5) \| 16.0 (5.3–25.5) \| 0.51; Method of completing questionnaire digitally \| 171 (85.5)* \| 0 (0)* \| 100 (100) \| <0.001; Current digital technology use (>1× per week) \| \| \| \|; – Smartphone \| 169 (85.3)* \| 67 (79.3)† \| 38 (92.7)† \| 0.017; – Computer/laptop \| 123 (62.4)* \| 42 (50.6)‡ \| 32 (78.0)† \| 0.003; – Tablet \| 102 (51.8)* \| 53 (63.2) \| 31 (75.6)* \| 0.016; – At least one of the above \| 188 (95.4) \| 83 (93.3) \| 39 (95.1) \| 0.46; Participated in research (clinic-based), n (%) \| 90 (45.7)* \| 47 (52.2)† \| 29 (70.7) \| 0.024 |
| **Response rate** | Of eligible participants, the % who completed the survey were:  Netherlands: 53.4%  UK: 41.6%  Australia: 27.1% |
| **OUTCOME 1: RESPIRATORY FUNCTION** | |
| **Instrument or method** | Patients ranked a top 3 out of 7 proposed outcomes from most valuable to least valuable. 1^st^ place received a score of 3, 3^rd^ place received a score of 1. The ranking score is the sum of scores per outcome measure. The ranking scores have been normalised by divided a ranking score by the highest possible ranking score. |
| **Preference set used in translation of raw results** | Not applicable. |
| **Reported format** | Normalised ranking score |
| **Result** | *Note the authors have not provided raw data for these outcomes. These values have been approximated using the GetData Graph Digitizer.  All countries: 0.63  Netherlands: 0.63  United Kingdom: 0.59  Australia: 0.5 |
| **OUTCOME 2: MUSCLE STRENGTH** | |
| **Instrument or method** | See outcome 1 |
| **Preference set used in translation of raw results** | Not applicable. |
| **Reported format** | Normalised ranking score |
| **Result** | *Note the authors have not provided raw data for these outcomes. These values have been approximated using the GetData Graph Digitizer.  All countries: 0.39  Netherlands: 0.39  United Kingdom: 0.34  Australia: 0.39 |
| **OUTCOME 3: QUALITY OF LIFE** | |
| **Instrument or method** | See outcome 1 |
| **Preference set used in translation of raw results** | Not applicable. |
| **Reported format** | Normalised ranking score |
| **Result** | *Note the authors have not provided raw data for these outcomes. These values have been approximated using the GetData Graph Digitizer.  All countries: 0.24  Netherlands: 0.24  United Kingdom: 0.23  Australia: 0.235 |
| **OUTCOME 4: FINE MOTOR/SPEECH** | |
| **Instrument or method** | See outcome 1 |
| **Preference set used in translation of raw results** | Not applicable. |
| **Reported format** | Normalised ranking score |
| **Result** | *Note the authors have not provided raw data for these outcomes. These values have been approximated using the GetData Graph Digitizer.  All countries: 0.22  Netherlands: 0.22  United Kingdom: 0.25  Australia: 0.19 |
| **OUTCOME 5: DAILY ACTIVITY** | |
| **Instrument or method** | See outcome 1 |
| **Preference set used in translation of raw results** | Not applicable. |
| **Reported format** | Normalised ranking score |
| **Result** | *Note the authors have not provided raw data for these outcomes. These values have been approximated using the GetData Graph Digitizer.  All countries: 0.21  Netherlands: 0.21  United Kingdom: 0.24  Australia: 0.21 |
| **OUTCOME 6: SLEEP** | |
| **Instrument or method** | See outcome 1 |
| **Preference set used in translation of raw results** | Not applicable. |
| **Reported format** | Normalised ranking score |
| **Result** | *Note the authors have not provided raw data for these outcomes. These values have been approximated using the GetData Graph Digitizer.  All countries: 0.17  Netherlands: 0.13  United Kingdom: 0.11  Australia: 0.39 |
| **OUTCOME 7: BODY WEIGHT** | |
| **Instrument or method** | See outcome 1 |
| **Preference set used in translation of raw results** | Not applicable. |
| **Reported format** | Normalised ranking score |
| **Result** | *Note the authors have not provided raw data for these outcomes. These values have been approximated using the GetData Graph Digitizer.  All countries: 0.16  Netherlands: 0.15  United Kingdom: 0.17  Australia: 0.21 |

**Risk of bias**

| Subdomain and signalling question | Judgement | Notes |
| --- | --- | --- |
| **Selection of participants** | Not serious |  |
| Was an appropriate study sample selected from the study’s sampling frame? | Yes | This was a broad and diverse international sample. While not random, the authors considered the appropriate avenues to access participants and verified diagnosis with national registries. |
| **Completeness of data** | Not serious |  |
| Was the attrition rate sufficiently low to minimise the risk of bias? | Yes | The survey was completed by 332 patients. While the response rate was low this has no bearing on attrition. |
| **Measurement instrument** | Not serious |  |
| Was the instrument used to measure patient values and preferences in a valid and reliable manner? | Yes | Although this is not a standardised or validated survey it was co-designed by patients with MND and explicitly asked patients to prioritise their top 3 most important outcomes. |
| Was the instrument used in the intended way? | Probably yes | The survey was administered consistently across an online platform and via post. This was a self-assessed survey, so there is potential for the survey to have been completed by the unintended participant, but this is unlikely. |
| Was a valid representation of the outcome (health state) used? | Probably yes | The authors do not report whether (or not) specific and highly detailed descriptions of the outcomes were provided. However, given this was co-designed by patients the outcomes selected are likely to be understood by those diagnosed with MND. |
| Did the researchers check for understanding of the instrument? | Investigators did not formally test the understanding, but evidence suggested inadequate understanding | While no formal testing of understanding was reported, this survey was co-designed by patients, suggesting that understanding of the instrument was likely appropriate for people diagnosed with MND. |
| **Data Analysis** | Not serious |  |
| Were the results analysed appropriately to avoid influence of bias and confounding? | Yes | The authors have followed a clear statistical analysis plan, using appropriate tests to determine if identified covariates were related to responses. |
| **Overall risk of bias** | **Not serious** |  |

**Jones 2014**

**Data extraction**

| Study ID | Jones 2014 |
| --- | --- |
| **DESIGN** | |
| **Other reports of this study identified?** | NR |
| **Time period study was conducted** | This data came from an 18-month, multicentre, double-blinded, randomised parallel group trial. Following the citation for this, the dates this data came from are May 2009 to November 2011. |
| **Design** | Direct utility and health state value study. Participants completed the EQ-5D (with VAS analogue where appropriate) and the HADS self-assessment form. |
| **Outcomes with a valuation provided relevant to the review** | Health-related quality of life (EQ-5D)  The have reported this across clinical stages of the disease. |
| **SETTING** | |
| **Country** | United Kingdom |
| **Sites/s** | London, Liverpool, Sheffield, Sussex, Oxford, and Newcastle |
| **Study locale** | Outpatient care; clinical trial setting |
| **Disease investigated** | Amyotrophic lateral sclerosis (ALS) |
| **PARTICPANTS** | |
| **Category of participants under investigation** | Adults diagnosed with ALS (Stages 2A to 4B). |
| **Eligibility criteria of participants** | Participants of the LiCALS trial (described above) participated. Eligible participants of the trial were adults aged at least 18 years who met the following criteria: possible, laboratory supported probable, probable, or definite ALS according to the revised version of the El Escorial criteria (the Airlie House Statement); 12 an electromyogram compatible with ALS; disease duration of at least 6 months and no more than 36 months (inclusive); and receipt of riluzole treatment for at least 1 month before enrolment. Patients with significant cognitive impairment were excluded from the LiCALS trial and consequently this paper. |
| **Sampling strategy** | Participants were recruited from the LiCALS trial: a multicentre, double-blind, randomised parallel group study. |
| **Number of participants included** | 214 |
| **Characteristics/demographics of participants included** | Characteristic \| Summary statistics; Total \| n = 214; Gender (Female) \| 66 (31%); Ethnicity (White) \| 210 (98%); Age at Onset (Mean, SD) \| 58.1 (10.8); Site of onset (Bulbar) \| 47 (22%); Type of onset (Sporadic) \| 167 (78%); El Escorial diagnostic category: Clinically definite ALS Clinically probable ALS Clinically probable laboratory supported ALS Clinically possible ALS \| 82 (38%) 80 (37%) 38 (17%) 14 (5%) |
| **Response rate** | 214 of 217 participants completed EQ-5D and HADS scales at least once |
| **OUTCOME 1: HEALTH-RELATED QUALITY OF LIFE (EQ-5D)** | |
| **Instrument or method** | EQ-5D. Anchored at 1 (perfect health) and 0 (death). |
| **Preference set used in translation of raw results** | UK preference set |
| **Reported format** | Mean and 95%CI |
| **Result** | 2A: 0.65 (0.59 – 0.71)  2B: 0.53 (0.49 – 0.58)  3: 0.41 (0.36 – 0.46)  4: 0.27 (0.24 – 0.30) |

**Risk of bias**

| Subdomain and signalling question | Judgement | Notes |
| --- | --- | --- |
| **Selection of participants** | Not serious |  |
| Was an appropriate study sample selected from the study’s sampling frame? | Yes | This data has been derived from participants who were recruited into a randomised controlled trial. |
| **Completeness of data** | Not serious |  |
| Was the attrition rate sufficiently low to minimise the risk of bias? | Yes | Of the 217 participants eligible to complete both the EQ-5D and the HADS assessment 214 did so. |
| **Measurement instrument** | Not serious |  |
| Was the instrument used to measure patient values and preferences in a valid and reliable manner? | Yes | The EQ-5D is a valid and reliable measures for patient values and preferences. |
| Was the instrument used in the intended way? | Yes | The EQ-5D is a self-assessment. Where appropriate, a visual analogue scale was used |
| Was a valid representation of the outcome (health state) used? | Probably yes | As this study utilised the EQ-5D it is assumed that participants were provided with high-quality, specified descriptions of the health states were provided |
| Did the researchers check for understanding of the instrument? | Investigators did not formally test the understanding, but evidence suggested adequate understanding | There is no statement from the authors that the participants understood (or were asked if they understood) the questions that were asked of them. |
| **Data Analysis** | Not serious |  |
| Were the results analysed appropriately to avoid influence of bias and confounding? | Yes. | The authors considered several factors in their analysis including gender, age of onset (AOO), disease onset site, treatment group (in the original trial), and ALS clinical stage. |
| **Overall risk of bias** | **Not serious** |  |

**Maksymowicz-Sliwinska 2023**

**Data extraction**

| Study ID | Maksymowicz-Sliwinska 2023 |
| --- | --- |
| **DESIGN** | |
| **Other reports of this study identified?** | None identified |
| **Time period study was conducted** | Not explicitly stated. However, this study was conducted within the ‘JPND Needs in ALS Project’. Which (according to a PDF on the linked website) started in January 2014 and concluded in December 2016. |
| **Design** | Cross-sectional study. Caregivers were asked a series of questions concerning their own attitudes towards the use of life-prolonging methods (NIV, IV, percutaneous gastrostomy – PEG) for the patient in case it was medically justified, and life-shortening methods. |
| **Outcomes with a valuation provided relevant to the review** | Would you like your relative with ALS to use life-prolonging measures?  If it were permitted by law, would you like your relative with ALS to use life-shortening measures? |
| **SETTING** | |
| **Country** | Poland and Germany |
| **Sites/s** | Department of Neurology, Medical University of Warsaw (Poland)  Department of Neurology, University of Ulm (Germany) |
| **Study locale** | Community/home-based caregivers of ALS patients |
| **Disease investigated** | ALS |
| **PARTICPANTS** | |
| **Category of participants under investigation** | Caregivers of adults with MND (informal caregivers e.g. not professional). |
| **Eligibility criteria of participants** | To be eligible to participate, caregivers had to be taking care of patients with clinically possible, probable or definite ALS according to the El Escorial criteria and disease duration of > 3 months.  The exclusion criteria included clinical diagnosis of dementia, additional neurological/psychiatric disorders or locked-in state. |
| **Sampling strategy** | The sampling strategy is not described in detail. Likely either purposive or consecutive sampling, given the nature of the study. |
| **Number of participants included** | Poland n = 80  Germany n = 84 |
| **Characteristics/demographics of participants included** | CGs \| Entire group N = 164 \| Polish group n = 80 \| German group n = 84 \| p-value; Gender (%) \| \| \| \|; Male \| 32.3 \| 25 \| 39.3 \| 0.05; Female \| 67.7 \| 75 \| 60.7 \|; Age (years) (%) \| \| \| \| 0.38; Mean ±SD \| 52.40 ±14.59 \| 51.36 ±14.70 \| 53.39 ±14.43 \|; Median \| 53 \| 53.5 \| 53 \|; < 30 \| 7.2 \| 9.0 \| 6.0 \|; 30–39 \| 12.0 \| 17.8 \| 7.2 \|; 40–49 \| 22.3 \| 17.8 \| 26.5 \|; 50–59 \| 24.2 \| 26.4 \| 22.9 \|; 60–69 \| 22.7 \| 14.4 \| 28.9 \|; > 70 \| 14.4 \| 10.2 \| 14.4 \|; Education (years) \| \| \| \| 0.85; Mean ±SD \| 14.40 ±2.78 \| 14.36 ±2.58 \| 14.45 ±2.97 \|; Median \| 14 \| 15 \| 14 \|; Religion (%) \| \| \| \| 0.0001; Catholic \| 59.8 \| 91.3 \| 29.8 \|; Protestants \| 20.1 \| 0.0 \| 39.3 \|; Atheists \| 18.9 \| 8.8 \| 28.6 \|; Other \| 1.2 \| 0.0 \| 2.4 \|; IIR (4–17) \| \| \| \| 0.0001; Mean ±SD \| 6.82 ±4.933 \| 11.10 ±3.634 \| 2.75 ±0.890 \|; Median \| 6 \| 12 \| 5 \|; Attending services (2–10) \| \| \| \| 0.0001; Mean ±SD \| 4.07 ±2.530 \| 5.88 ±2.166 \| 2.36 ±1.411 \|; Median \| 3 \| 6 \| 2 \|; Degree of belief (2–7) \| \| \| \| 0.0001; Mean ±SD \| 3.96 ±1.784 \| 5.23 ±1.599 \| 2.75 ±0.890 \|; Median \| 4 \| 6 \| 3 \|; CG’s family status (%) \| \| \| \| 0.54; Spouse/partner \| 65.9 \| 61.3 \| 70.2 \|; Children \| 22.0 \| 25.0 \| 19.0 \|; Parents \| 3.7 \| 5.0 \| 2.4 \|; Siblings \| 3.7 \| 2.5 \| 4.8 \|; Others \| 4.9 \| 6.3 \| 3.6 \|; ECAS-BS (0–10) \| \| \| \| 0.1; Mean ±SD \| 0.55 ±0.99 \| 0.63 ±1.02 \| 0.48 ±0.97 \| |
| **Response rate** | Not stated |
| **OUTCOME 1: WOULD YOU LIKE YOUR RELATIVE WITH ALS TO USE LIFE-PROLONGING MEASURES** | |
| **Instrument or method** | A self-administered survey, where patients were asked a series of questions. This question could be answered with a) definitely not, b) probably not, c) probably yes, d) definitely yes |
| **Preference set used in translation of raw results** | Not applicable |
| **Reported format** | Percentage of responses |
| **Result** | Entire Group Polish Group German Group  No or Probably No (%) 30.5 17.5 42.9  Probably Yes or Yes (%) 69.5 82.5 57.1 |
| **OUTCOME 2: IF PERMITTED BY LAW, WOULD YOU LIKE YOUR RELATIVE WITH ALS TO USE LIFE-SHORTENING MEASURES** | |
| **Instrument or method** | A self-administered survey, where patients were asked a series of questions. This question could be answered with a) definitely not, b) probably not, c) probably yes, d) definitely yes |
| **Preference set used in translation of raw results** | Not applicable |
| **Reported format** | Percentage of responses |
| **Result** | Entire Group Polish Group German Group  No or Probably No (%) 61.3 73.8 48.8  Probably Yes or Yes (%) 38.7 26.2 51.2 |

**Risk of bias**

| Subdomain and signalling question | Judgement | Notes |
| --- | --- | --- |
| **Selection of participants** | Serious risk |  |
| Was an appropriate study sample selected from the study’s sampling frame? | Probably no | The method of sampling was not described in sufficient methodological detail. Despite this being a cross-sectional study, how patients were approached to participate should have been reported. As should the number of participants that chose not to participate. The sample was well defined however, but a response of probably no is appropriate here. |
| **Completeness of data** | Not serious |  |
| Was the attrition rate sufficiently low to minimise the risk of bias? | Yes | All 164 caregivers completed the survey. There was no report of participants not completing the survey or only partially completing the survey |
| **Measurement instrument** | Not serious |  |
| Was the instrument used to measure patient values and preferences in a valid and reliable manner? | Probably yes | The study was conducted prospectively using a questionnaire designed within the JPND Needs in ALS project. |
| Was the instrument used in the intended way? | Yes | Self-administered survey |
| Was a valid representation of the outcome (health state) used? | Yes | Questions were asked about hypothetical scenarios and measured the caregivers perceptions on these. The outcomes were all related to the objective outcome of death |
| Did the researchers check for understanding of the instrument? | Investigators did not formally test the understanding, but evidence suggested adequate understanding | No mention of testing or pre-testing piloting |
| **Data Analysis** | Extremely serious risk |  |
| Were the results analysed appropriately to avoid influence of bias and confounding? | No. | Only raw data expressed as percentages is presented |
| **Overall risk of bias** | **Extremely serious** |  |

**Moore 2019**

**Data extraction**

| Study ID | Moore 2019 |
| --- | --- |
| DESIGN | |
| **Other reports of this study identified?** | None identified |
| **Time period study was conducted** | Not stated |
| **Design** | Direct utility and health state value study.  Data were obtained from the TONiC study, which is a longitudinal cohort study. Patients attending clinics are provided with questionnaires at 0, 4, 9, 14, 18, 27, and 60 months from their inclusion in the TONiC study.  Patients completed the EQ-5D-5L, which comprises of 5 domains: mobility, self-care, usual activities, pain/discomfort, and anxiety/depression. Each of these domains has 5 levels indicating worsening health, from having no problems to having severe problems. Responses to the EQ-5D-5L were used to calculate health utilities.  EQ-5D is the same but with visual analogue adaptability  The ALSUI was calculated from responses to the ALSFRS-R.  This measure is the first such to present a disease-specific, preference-based index in MND, based on scoring determined from a standard gamble experiment conducted among members of the general public in the United States. The ALSUI algorithm attaches a preference weighting to ALSFRS-R items 1, 6, 8, 10, and 12, to obtain a single index value ranging from 0 (worse possible state) to 1 (best possible state). |
| **Outcomes with a valuation provided relevant to the review** | Health-related quality of life (EQ-5D-5L)  Health-related quality of life (EQ-5D VAS)  Health-related quality of life (ALSUI) |
| **SETTING** | |
| **Country** | United Kingdom |
| **Sites/s** | 22 regional MND clinics. These clinics were participating in the broader ongoing TONiC study |
| **Study locale** | Community-based |
| **Disease investigated** | ALS (but use the terms MND and ALS interchangeably). |
| **PARTICPANTS** | |
| **Category of participants under investigation** | Adults diagnosed with MND |
| **Eligibility criteria of participants** | Eligibility criteria were not explicitly reported in the paper. Patients were recruited from MND clinics as per the TONiC study. |
| **Sampling strategy** | Sampling strategy was not explicitly reported in the paper. Patients were recruited from MND clinics as per the TONiC study. However, it is likely that all registered attendees at the clinics received the postal questionnaire |
| **Number of participants included** | Questionnaires were posted to 958 eligible patients. However, data from only 595 patients has been utilised. |
| **Characteristics/demographics of participants included** | Characteristic \| Value; Sample size, n \| 595; Age in years, mean (SD) \| 65.07 (10.89); Female, n (%) \| 232 (39.0%); Months since diagnosis, mean (SD) \| 26.54 (38.8); MND onset type, n (%) \|; Limb \| 404 (69.9%); Bulbar \| 159 (26.7%); Respiratory \| 11 (2.5%); ALSFRS-R index \|; Mean (95% CI) \| 31.95 (31.19–32.55); Median (IQR) \| 33 (27–38); Kings staging, n (%) \|; Stage 1 \| 89 (15.0%); Stage 2 \| 135 (22.7%); Stage 3 \| 206 (34.6%); Stage 4 \| 162 (27.3%); MiToS staging, n (%) \|; Stage 0 \| 301 (50.59%); Stage 1 \| 198 (33.28%); Stage 2 \| 73 (12.26%); Stage 3 \| 18 (3.03%); Stage 4 \| 5 (0.84%) |
| **Response rate** | In total, 958 patients received posted questionnaires. Of which, only 636 were returned and 41 of these were not sufficiently completed. Health utility data was available from 595 patients. |
| **OUTCOME 1: HEALTH-RELATED QUALITY OF LIFE (EQ-5D-5L)** | |
| **Instrument or method** | EQ-5D-5L |
| **Preference set used in translation of raw results** | UK value set |
| **Reported format** | Mean (95% CI) |
| **Result** | Full Sample: 0.57 (0.55 – 0.59)  Kings Stage 1: 0.76 (0.71 – 0.80)  Kings Stage 2: 0.60 (0.56 – 0.64)  Kings Stage 3: 0.53 (0.50 – 0.56)  Kings Stage 4: 0.50 (0.45 – 0.54)  MiToS Stage 0: 0.71 (0.69–0.73)  MiToS Stage 1: 0.48 (0.44–0.51)  MiToS Stage 2: 0.36 (0.31–0.42)  MiToS Stage 3: 0.33 (0.27–0.40)  MiToS Stage 4: 0.25 (0.13–0.34) |
| **OUTCOME 3: HEALTH-RELATED QUALITY OF LIFE (EQ-5D VAS)** | |
| **Instrument or method** | EQ-5D VAS |
| **Preference set used in translation of raw results** | None (direct patient valuation) |
| **Reported format** | Mean (95% CI) |
| **Result** | Full Sample: 60 (58-62)  Kings Stage 1: 72 (68-76)  Kings Stage 2: 63 (59 – 66)  Kings Stage 3: 59 (57-62)  Kings Stage 4: 52 (48-56)  MiToS Stage 0: 68 (66-70)  MiToS Stage 1: 55 (52-58)  MiToS Stage 2: 49 (43-54)  MiToS Stage 3: 47 (37-58)  MiToS Stage 4: 45 (22-70) |
| **OUTCOME 2: HEALTH-RELATED QUALITY OF LIFE (ALSUI)** | |
| **Instrument or method** | ALSUI translates ALSFRS-R scores into utility values using public preferences obtained via standard gamble. |
| **Preference set used in translation of raw results** | Standard gamble experiment from general public in the United States |
| **Reported format** | Mean (95% CI) |
| **Result** | Full Sample: 0.4 (0.38-0.42)  Kings Stage 1: 0.63 (0.6-0.68)  Kings Stage 2: 0.5 (0.46-0.54)  Kings Stage 3: 0.35 (0.33-0.37)  Kings Stage 4: 0.24 (0.21-0.26)  MiToS Stage 0: 0.56 (0.54-0.58)  MiToS Stage 1: 0.30 (0.28-0.32)  MiToS Stage 2: 0.16 (0.13-0.18)  MiToS Stage 3: 0.08 (0.06-0.11)  MiToS Stage 4: 0.07 (0.03-0.09) |

**Risk of bias**

| Subdomain and signalling question | Judgement | Notes |
| --- | --- | --- |
| **Selection of participants** | Not serious |  |
| Was an appropriate study sample selected from the study’s sampling frame? | Probably yes | The recruitment strategy is not described in sufficient detail. However, the authors reported that 958 eligible participants were posted the questionnaires. Which indicates that all registered and eligible (noting eligibility criteria were not documented) were sent the questionnaire. More details would permit a stronger yes |
| **Completeness of data** | Not serious |  |
| Was the attrition rate sufficiently low to minimise the risk of bias? | Probably yes | Only 595 participants (of 958 eligible) completed the survey as instructed. While this is only 62% as this was a posted survey this seems mostly appropriate |
| **Measurement instrument** | Not serious |  |
| Was the instrument used to measure patient values and preferences in a valid and reliable manner? | Yes | Both the EQ-5D and the ALSUI were used appropriately. |
| Was the instrument used in the intended way? | Yes | The EQ-5D and the ALSFRS-R (which informs the ALSUI) were used as per the guidance for these instruments. |
| Was a valid representation of the outcome (health state) used? | Yes | The EQ-5d asks participants to consider their own outcomes, as does the ALSFRS-R. |
| Did the researchers check for understanding of the instrument? | Investigators did not formally test the understanding, but evidence suggested adequate understanding | No mention of testing |
| **Data Analysis** | Not serious |  |
| Were the results analysed appropriately to avoid influence of bias and confounding? | Probably yes | While a GLM was used in this study it was not for the evaluation of health-states. However, given these health state data are converted to a single utility value using a pre-defined scoring algorithm, the presentation of simple means and confidence intervals is appropriate for this data. |
| **Overall risk of bias** | **Not serious** |  |

**Raheja 2016**

**Data extraction**

| Study ID | Raheja 2016 |
| --- | --- |
| **DESIGN** | |
| **Other reports of this study identified?** | None identified |
| **Time period study was conducted** | 4^th^ September 2006 and 30^th^ July 2012 (original RCT) |
| **Design** | Cross-sectional survey with direct valuation This was a retrospective study using data from a previous RCT.  Participants who partook in the trial for 96 weeks completed the ALSSQoL.  The authors have only reported the data for the 6 items from the non-bulbar symptoms of the ALSSQoL ad 4 items from the bulbar symptoms of the ALSSQoL. |
| **Outcomes with a valuation provided relevant to the review** | Non-Bulbar:  Pain score (ALSSQoL)  Fatigue Score (ALSSQoL)  Breathing score (ALSSQoL)  Strength/ability to move score (ALSSQoL)  Sleep score (ALSSQoL)  Bowel and bladder score (ALSSQoL)  Bulbar:  Eating score (ALSSQoL)  Excessive Saliva (ALSSQoL)  Mucus score (ALSSQoL)  Speaking score (ALSSQoL) |
| **SETTING** | |
| **Country** | United States and Canada |
| **Sites/s** | The RCT in which this data is derived was performed at 59 clinical sites in the USA and Canada |
| **Study locale** | Outpatient ALS clinics |
| **Disease investigated** | ALS |
| **PARTICPANTS** | |
| **Category of participants under investigation** | Adults diagnosed with MND |
| **Eligibility criteria of participants** | The main inclusion and exclusion criteria for the trial were an El Escorial diagnosis of possible ALS or higher, vital capacity of more than 60% of predicted, and symptom duration of less than three years. To be included in this retrospective data analysis, participants must have completed a minimum of 96 weeks in the trial.  Participants must have had no dementia or other disorder and no locked-in-syndrome. |
| **Sampling strategy** | Retrospective sample of previously randomised participants who had maximum follow-up at 96 weeks. |
| **Number of participants included** | 82 participants of the original trial were eligible for this secondary analysis. |
| **Characteristics/demographics of participants included** | No baseline demographics table was provided. The authors have narratively reported that There were 82 subjects for whom there were complete ALSSQoL data for week 0 and week 96. Fifty-four (56%) of the subjects were males. The mean age was 53 ± 10.3 years. |
| **Response rate** | Not applicable. This was a retrospective analysis of RCT data. |
| **OUTCOME 1: PAIN (ALSSQOL)** | |
| **Instrument or method** | The ALSSQoL is an ALS-specific measure of overall QoL, assessing health-related and non-health-related factors. It assesses overall QoL via six specific domains and has been validated on a national U.S. sample of adult males and females who were receiving care for their disease in ALS multidisciplinary clinics.  Each of its 59 items uses a 0–10-point Likert scale, with 0 being the least desirable situation, and 10 the most desirable. |
| **Preference set used in translation of raw results** | None; outcomes based on patient perception of burden, not societal utility valuation |
| **Reported format** | Mean ± SD |
| **Result** | Week 0: 1.6 ± 2.27  Week 96: 2.5 ± 2.37 |
| **OUTCOME 2: FATIGUE (ALSSQOL)** | |
| **Instrument or method** | The ALSSQoL is an ALS-specific measure of overall QoL, assessing health-related and non-health-related factors. It assesses overall QoL via six specific domains and has been validated on a national U.S. sample of adult males and females who were receiving care for their disease in ALS multidisciplinary clinics.  Each of its 59 items uses a 0–10-point Likert scale, with 0 being the least desirable situation, and 10 the most desirable. |
| **Preference set used in translation of raw results** | None; outcomes based on patient perception of burden, not societal utility valuation |
| **Reported format** | Mean ± SD |
| **Result** | Week 0: 3.24 ± 2.24  Week 96: 4.38 ± 2.22 |
| **OUTCOME 3: BREATHING (ALSSQOL)** | |
| **Instrument or method** | The ALSSQoL is an ALS-specific measure of overall QoL, assessing health-related and non-health-related factors. It assesses overall QoL via six specific domains and has been validated on a national U.S. sample of adult males and females who were receiving care for their disease in ALS multidisciplinary clinics.  Each of its 59 items uses a 0–10-point Likert scale, with 0 being the least desirable situation, and 10 the most desirable. |
| **Preference set used in translation of raw results** | None; outcomes based on patient perception of burden, not societal utility valuation |
| **Reported format** | Mean ± SD |
| **Result** | Week 0: 0.67 ± 1.33  Week 96: 2.83 ± 2.77 |
| **OUTCOME 4: STRENGTH/ ABILITY TO MOVE (ALSSQOL)** | |
| **Instrument or method** | The ALSSQoL is an ALS-specific measure of overall QoL, assessing health-related and non-health-related factors. It assesses overall QoL via six specific domains and has been validated on a national U.S. sample of adult males and females who were receiving care for their disease in ALS multidisciplinary clinics.  Each of its 59 items uses a 0–10-point Likert scale, with 0 being the least desirable situation, and 10 the most desirable. |
| **Preference set used in translation of raw results** | None; outcomes based on patient perception of burden, not societal utility valuation |
| **Reported format** | Mean ± SD |
| **Result** | Week 0: 3.96 ± 2.57  Week 96: 6.46 ± 2.36 |
| **OUTCOME 5: SLEEP (ALSSQOL)** | |
| **Instrument or method** | The ALSSQoL is an ALS-specific measure of overall QoL, assessing health-related and non-health-related factors. It assesses overall QoL via six specific domains and has been validated on a national U.S. sample of adult males and females who were receiving care for their disease in ALS multidisciplinary clinics.  Each of its 59 items uses a 0–10-point Likert scale, with 0 being the least desirable situation, and 10 the most desirable. |
| **Preference set used in translation of raw results** | None; outcomes based on patient perception of burden, not societal utility valuation |
| **Reported format** | Mean ± SD |
| **Result** | Week 0: 1.44 ± 1.9  Week 96: 2.85 ± 2.54 |
| **OUTCOME 6: BOWEL AND BLADDER (ALSSQOL)** | |
| **Instrument or method** | The ALSSQoL is an ALS-specific measure of overall QoL, assessing health-related and non-health-related factors. It assesses overall QoL via six specific domains and has been validated on a national U.S. sample of adult males and females who were receiving care for their disease in ALS multidisciplinary clinics.  Each of its 59 items uses a 0–10-point Likert scale, with 0 being the least desirable situation, and 10 the most desirable. |
| **Preference set used in translation of raw results** | None; outcomes based on patient perception of burden, not societal utility valuation |
| **Reported format** | Mean ± SD |
| **Result** | Week 0: 0.94 ± 1.79  Week 96: 2.18 ± 2.54 |
| **OUTCOME 7: EATING (ALSSQOL)** | |
| **Instrument or method** | The ALSSQoL is an ALS-specific measure of overall QoL, assessing health-related and non-health-related factors. It assesses overall QoL via six specific domains and has been validated on a national U.S. sample of adult males and females who were receiving care for their disease in ALS multidisciplinary clinics.  Each of its 59 items uses a 0–10-point Likert scale, with 0 being the least desirable situation, and 10 the most desirable. |
| **Preference set used in translation of raw results** | None; outcomes based on patient perception of burden, not societal utility valuation |
| **Reported format** | Mean ± SD |
| **Result** | Week 0: 0.91 ± 1.54  Week 96: 2.54 ± 3.09 |
| **OUTCOME 8: EXCESSIVE SALIVA (ALSSQOL)** | |
| **Instrument or method** | The ALSSQoL is an ALS-specific measure of overall QoL, assessing health-related and non-health-related factors. It assesses overall QoL via six specific domains and has been validated on a national U.S. sample of adult males and females who were receiving care for their disease in ALS multidisciplinary clinics.  Each of its 59 items uses a 0–10-point Likert scale, with 0 being the least desirable situation, and 10 the most desirable. |
| **Preference set used in translation of raw results** | None; outcomes based on patient perception of burden, not societal utility valuation |
| **Reported format** | Mean ± SD |
| **Result** | Week 0: 1.12 ± 1.92  Week 96: 2.54 ± 2.93 |
| **OUTCOME 9: MUCUS (ALSSQOL)** | |
| **Instrument or method** | The ALSSQoL is an ALS-specific measure of overall QoL, assessing health-related and non-health-related factors. It assesses overall QoL via six specific domains and has been validated on a national U.S. sample of adult males and females who were receiving care for their disease in ALS multidisciplinary clinics.  Each of its 59 items uses a 0–10-point Likert scale, with 0 being the least desirable situation, and 10 the most desirable. |
| **Preference set used in translation of raw results** | None; outcomes based on patient perception of burden, not societal utility valuation |
| **Reported format** | Mean ± SD |
| **Result** | Week 0: 1.24 ± 1.73  Week 96: 2.74 ± 2.84 |
| **OUTCOME 10: SPEAKING (ALSSQOL)** | |
| **Instrument or method** | The ALSSQoL is an ALS-specific measure of overall QoL, assessing health-related and non-health-related factors. It assesses overall QoL via six specific domains and has been validated on a national U.S. sample of adult males and females who were receiving care for their disease in ALS multidisciplinary clinics.  Each of its 59 items uses a 0–10-point Likert scale, with 0 being the least desirable situation, and 10 the most desirable. |
| **Preference set used in translation of raw results** | None; outcomes based on patient perception of burden, not societal utility valuation |
| **Reported format** | Mean ± SD |
| **Result** | Week 0: 1.85 ± 2.59  Week 96: 4.30 ± 3.77 |

**Risk of bias**

| Subdomain and signalling question | Judgement | Notes |
| --- | --- | --- |
| **Selection of participants** | Not serious |  |
| Was an appropriate study sample selected from the study’s sampling frame? | Probably yes | Participants were selected based on their participation in a previous RCT. They had already been randomly selected and allocated to groups; however, all participant data was used here (regardless of group assignment). However, only participants that completed follow-up at 96 weeks were included however, which means there may be a bias in the presentation of the data. |
| **Completeness of data** | Not serious |  |
| Was the attrition rate sufficiently low to minimise the risk of bias? | Yes | Retrospective sampling, no attrition |
| **Measurement instrument** | Not serious |  |
| Was the instrument used to measure patient values and preferences in a valid and reliable manner? | Yes | The ALSSQoL is a validated, ALS-specific instrument designed to assess symptoms burden, and asks participants to determine their outcomes from the least desirable situation to the most desirable situation |
| Was the instrument used in the intended way? | Yes | The ALSSQoL was administered as per the trial protocol at standardised intervals |
| Was a valid representation of the outcome (health state) used? | Yes | The ALSSQoL asks participants to assess their subjective burden of their existing symptoms |
| Did the researchers check for understanding of the instrument? | Investigators did not formally test the understanding, but evidence suggested adequate understanding | No explicit mention of pre-testing comprehension or pilot testing for the ALSSQoL in this trial population. |
| **Data Analysis** | Not serious |  |
| Were the results analysed appropriately to avoid influence of bias and confounding? | Yes | The data has come from participants randomised as per the RCT protocol. The presentation of data is appropriate. |
| **Overall risk of bias** | **Not serious** |  |

**Rudnicki 1997**

**Data extraction**

| Study ID | Rudnicki 1997 |
| --- | --- |
| **DESIGN** | |
| **Other reports of this study identified?** | None identified |
| **Time period study was conducted** | February 1996 – September 1996 |
| **Design** | Observational descriptive study  Patients were walked through an option of using Riluzole. |
| **Outcomes with a valuation provided relevant to the review** | Reasons for not taking Riluzole |
| **SETTING** | |
| **Country** | United States |
| **Sites/s** | Little Rock, Arkansas  University of Arkansas for Medical Sciences Motor Neuron Disease Clinic |
| **Study locale** | Outpatient MND clinic |
| **Disease investigated** | ALS |
| **PARTICPANTS** | |
| **Category of participants under investigation** | Adults diagnosed with MND |
| **Eligibility criteria of participants** | Probable of definite ALS by El Escorial criteria. Patients seen at the clinic during the study period |
| **Sampling strategy** | Consecutive sampling Patients who attended the clinic between February and September 1996 |
| **Number of participants included** | N=46 |
| **Characteristics/demographics of participants included** | No participant demographics were provided  Of all included patients 17 (37%) chose to take Riluzole and 29 (63%) declined taking Riluzole |
| **Response rate** | NA |
| **OUTCOME 1: REASONS FOR NOT TAKING RILUZOLE** | |
| **Instrument or method** | Assumed semi-structured interview. The only methodological detail provided reads “If a patient chose not to take the Riluzole, they were asked to give the reason(s). |
| **Preference set used in translation of raw results** | NA |
| **Reported format** | Raw counts for provided reasons |
| **Result** | Insufficient benefit \| 14; High cost \| 9; Do not want to prolong life \| 8; Doing worse \| 2*; Potential side effects \| 2; Lack of improved QoL \| 1; Participation in another study \| 1; *These patients had participated in the early access program for riluzole \| |

**Risk of bias**

| Subdomain and signalling question | Judgement | Notes |
| --- | --- | --- |
| **Selection of participants** | Not serious |  |
| Was an appropriate study sample selected from the study’s sampling frame? | Probably yes | Consecutive sampling of a relevant population |
| **Completeness of data** | Not serious |  |
| Was the attrition rate sufficiently low to minimise the risk of bias? | Yes | No attrition |
| **Measurement instrument** | Very serious |  |
| Was the instrument used to measure patient values and preferences in a valid and reliable manner? | No | Assumed semi-structured interview but too few methodological details are provided. No validated survey instrument was employed to capture preferences it seems. |
| Was the instrument used in the intended way? | Probably yes | Limited methodological details are provided; it is likely that all patients were asked the same questions but uniformity of this may be questioned without proper methodological detail. |
| Was a valid representation of the outcome (health state) used? | Yes | Reasons for Riluzole refusal were directly related to the outcomes of the patients and the importance they placed on them |
| Did the researchers check for understanding of the instrument? | Investigators did not formally test the understanding, but evidence suggested adequate understanding | No formal cognitive testing of patient understanding reported. |
| **Data Analysis** | Extremely serious |  |
| Were the results analysed appropriately to avoid influence of bias and confounding? | No | Basic descriptive statistics only |
| **Overall risk of bias** | **Extremely serious** |  |

**Sandstedt 2018**

**Data extraction**

| Study ID | Sandstedt 2018 |
| --- | --- |
| **DESIGN** | |
| **Other reports of this study identified?** | None identified |
| **Time period study was conducted** | October 2012 – January 2013 |
| **Design** | Cross-sectional study  Standardised and study-specific questionnaires were used (Caregiver Reaction Assessment CRA)) was used to assess informal caregivers experience of caregiving. The EQ 5D-VAS and SF-36 was used to assess HRQoL of the caregivers |
| **Outcomes with a valuation provided relevant to the review** | Health-related quality of life (EQ-5D-VAS) |
| **SETTING** | |
| **Country** | Sweden |
| **Sites/s** | Karolinska University Hospital, Stockholm |
| **Study locale** | In home visits of ALS patients (data was collected from caregivers during these visits) |
| **Disease investigated** | ALS |
| **PARTICPANTS** | |
| **Category of participants under investigation** | Informal caregivers of adults with MND |
| **Eligibility criteria of participants** | Informal caregivers of ALS patients who were recruited from Karolinska University Hospital who were also taking part in another study. ALS diagnosis according to the revised El Escorial criteria and 18 years or older |
| **Sampling strategy** | Consecutive sampling All caregivers identified from ALS patients participating in a previous study were invited. ALS patients were registered on a hospital’s registry. Caregivers of these patients were approached. |
| **Number of participants included** | N=49 (However only 47 EQVAS results are present) |
| **Characteristics/demographics of participants included** | Characteristic \| Value; Informal caregivers \|; Age, years, mean (SD) \| 57 (14); Women, n (%) \| 34 (69); University education, n (%) \| 24 (49); Work status, full/part time, n (%) \| 14/16 (29/33); Changed working time (after ALS diagnosis), n (%) \| 15 (31); Relation to patient with ALS, n (%) \|; Partner/ex-partner \| 31/2 (63/4); Child \| 8 (26); Parent/sibling/friend \| 1/6/1 (2/12/2); Cohabiting with patient with ALS, n (%) \| 33 (67); Informal caregiving >7 hrs/week, n (%) \|; In personal activities of daily living (P-ADL) \| 18 (37); In instrumental-ADL (I-ADL) \| 20 (41); In other activities \| 18 (37); In P-ADL, I-ADL and/or other activities \| 35 (71); Patients with ALS \|; Age, years, mean (SD) \| 63 (11); Men, n (%) \| 28 (57); University education, n (%) \| 18 (37); Severe disease, n (%) \| 17 (35); Low coping capacity, n (%) \| 6 (13); Fatigue, n (%) \| 27 (61); Anxiety and/or depression, n (%) \| 17 (35); Low frequency of social and lifestyle activities, n (%) \| 38 (78); Home care or salaried personal assistance, n (%) \| 24 (49); Home adaptations/technical aids, n (%) \| 30/46 (61/94) |
| **Response rate** | 58 caregivers were approached but only 49 agreed to participate. |
| **OUTCOME 1: HEALTH-RELATED QUALITY OF LIFE (EQ-5D-VAS)** | |
| **Instrument or method** | The EQ VAS is a vertical 20 cm scale ranging from zero (worst health) to 100 (best health), on which respondents mark their health state “today.” The EQ VAS is a participant‐based measure and a valid and reliable reflection of the participant's own judgement. |
| **Preference set used in translation of raw results** | The mean norm value in the Swedish population is reportedly 83 |
| **Reported format** | Mean, range |
| **Result** | Mean EQVAS = 66 (ranging from 15-95). |

**Risk of bias**

| Subdomain and signalling question | Judgement | Notes |
| --- | --- | --- |
| **Selection of participants** | Not serious |  |
| Was an appropriate study sample selected from the study’s sampling frame? | Yes | All caregivers identified from ALS patients participating in a previous study were invited. ALS patients were registered on a hospital’s registry. Caregivers of these patients were approached |
| **Completeness of data** | Not serious |  |
| Was the attrition rate sufficiently low to minimise the risk of bias? | Probably yes | Very high response rate (49 of 58). However worth noting that only 47 caregivers provided valuation through EQVAS. The difference from 49 is not discussed by authors but this is low attrition |
| **Measurement instrument** | Not serious |  |
| Was the instrument used to measure patient values and preferences in a valid and reliable manner? | Yes. | The EQ-5D VAS is a validated instrument with well documented ability to assess health utility states. |
| Was the instrument used in the intended way? | Yes | EQ-5D VAS was utilised as intended administered at home visits by centre staff to ALS patients |
| Was a valid representation of the outcome (health state) used? | Yes | As this study utilised the EQ-5D it is assumed that participants were provided with high-quality, specific descriptions of the health states were provided |
| Did the researchers check for understanding of the instrument? | Investigators did not formally test the understanding, but evidence suggested adequate understanding | There is no statement from the authors that the participants understood (or were asked if they understood) the questions that were asked of them. |
| **Data Analysis** | Not serious |  |
| Were the results analysed appropriately to avoid influence of bias and confounding? | Yes | Multiple variables were tested via multivariable regression analyses |
| **Overall risk of bias** | Not serious |  |

**Seo 2024**

**Data extraction**

| Study ID | Seo 2024 |
| --- | --- |
| **DESIGN** | |
| **Other reports of this study identified?** | None identified |
| **Time period study was conducted** | November 2020 – April 2022 |
| **Design** | Online survey that included a discrete choice experiment and a threshold technique exercise.  Participants followed a web link to the online survey, where they answered screening questions and then read and completed an online consent form. After providing consent, participants were shown text asking them to imagine a situation in which their physician advised that they would need regular injections of medicine into the intrathecal space.  Participants were presented with descriptions of IDDD and lumbar puncture options for intrathecal drug delivery, an illustration of each option, and descriptions of the attributes used in the survey.  They then advanced to the DCE and threshold technique exercises. |
| **Outcomes with a valuation provided relevant to the review** | Patient preferences for intrathecal drug administration  Maximum acceptable risk |
| **SETTING** | |
| **Country** | United states, France, Germany, Italy, Spain, UK |
| **Sites/s** | Online survey |
| **Study locale** | Community-based patients with ALS |
| **Disease investigated** | ALS |
| **PARTICPANTS** | |
| **Category of participants under investigation** | Adults with MND |
| **Eligibility criteria of participants** | Patients were eligible for if they were diagnosed with ALS, aged =>18 years, no current used of IDDD, were not in hospice care and did not have a tracheostomy |
| **Sampling strategy** | Convenience sampling  Online survey, US participants were recruited via physician referral. Participants across Europe were recruited via physician referral as well as social media and patient advocacy groups. |
| **Number of participants included** | n=295 |
| **Characteristics/demographics of participants included** | Characteristic \| Overall (N=295) \| US (n=200) \| Europe (n=95); **Age** \| \| \|; Mean (SD) [min–max] \| 57.7 (11.2) [20–84] \| 59.6 (10.7) [32–84] \| 53.9 (11.2) [20–83]; **Sex** \| \| \|; Female \| 149 (50.5) \| 101 (50.5) \| 48 (50.5); **Racial/ethnic background** \| \| \|; White \| – \| 138 (69.0) \| –; Black/African/Caribbean \| – \| 23 (11.5) \| –; Asian \| – \| 10 (5.0) \| –; Other \| – \| 24 (12.0) \| –; Prefer not to say \| – \| 5 (2.5) \| –; **Highest level of education** \| \| \|; Less than high school \| 14 (4.7) \| 3 (1.5) \| 11 (11.6); High school \| 81 (27.5) \| 36 (18.0) \| 45 (47.4); Some college \| 73 (24.7) \| 60 (30.0) \| 13 (13.7); College/university degree \| 85 (28.8) \| 71 (35.5) \| 14 (14.7); Graduate school degree \| 39 (13.2) \| 27 (13.5) \| 12 (12.6); Other \| 3 (1.0) \| 3 (1.5) \| 0 (0.0); **Time since ALS diagnosis** \| \| \|; <6 months \| 45 (15.3) \| 31 (15.5) \| 14 (14.7); 6 months–1 year \| 67 (22.7) \| 53 (26.5) \| 14 (14.7); 1 year–3 years \| 107 (36.3) \| 73 (36.5) \| 34 (35.8); 3 years–5 years \| 42 (14.2) \| 25 (12.5) \| 17 (17.9); 5 years–10 years \| 24 (8.1) \| 14 (7.0) \| 10 (10.5); ≥10 years \| 9 (3.1) \| 3 (1.5) \| 6 (6.3); Do not know/do not remember \| 1 (0.3) \| 1 (0.5) \| 0 (0.0); **ALSFRS-R score** \| \| \|; Mean (SD) [min–max] \| 31.8 (10.1) [6–48] \| 33.5 (9.8) [6–48] \| 28.3 (9.7) [7–44]; ALSFRS-R severity grouping \| \| \|; >40 (minimal to mild disease) \| 84 (28.5) \| 68 (34.0) \| 16 (16.8); 30–39 (mild to moderate disease) \| 84 (28.5) \| 57 (28.5) \| 27 (28.4); 20–29 (moderate to severe disease) \| 85 (28.8) \| 56 (28.0) \| 29 (30.5); <20 (advanced disease) \| 42 (14.2) \| 19 (9.5) \| 23 (24.2) |
| **Response rate** | The online resources states that overall, 328 people were invited and 295 provided data. Low attrition rate. |
| **OUTCOME 1: PATIENT PREFERENCES FOR IDDD VERSUS LP (DCE)** | |
| **Instrument or method** | The DCE elicited acceptable trade-offs between overall duration, location, frequency, risk associated with each injection, and device failure risk. Within the DCE, participants were asked to repeatedly make choices between a lumbar puncture profile and two IDDD profiles. Each profile was described by the five DCE attributes, and levels varied across profiles and choice tasks to encourage participants to make trade-offs when denoting their administration preference. The design consisted of 26 choice tasks, which were split into two blocks of equal size to minimize the burden imposed on participants. The order of the choice tasks was randomized between participants to avoid ordering effects, and each participant was randomly allocated to one of the two blocks. The scenarios were presented as such: Attribute \| Scenario 1 (LP / IDDD) \| Scenario 2 (LP / IDDD) \| Scenario 3 (LP / IDDD) \| Scenario 4 (LP / IDDD); **Overall duration** \| 2 hr / 1 hr \| 1 hr / 1 hr \| 2 hr / 90 min \| 1 hr / 90 min; **Location** \| Hospital / Local clinic \| ALS Hospital / Local clinic \| Hospital / Hospital \| ALS Hospital / Hospital; **Frequency** \| Every month / Every month \| Every 4 months / Every 4 months \| Every month / Every month \| Every month / Every month; **Administration risk** \| 1% / 2% \| 1% / 3% \| 1% / 1% \| 1% / 1%; **Risk of device failure** \| n/a / 13% \| n/a / 15% \| n/a / 13% \| n/a / 13%; **Risk of implantation surgery** \| n/a / 5% \| n/a / 2% \| n/a / 2% \| n/a / 5% |
| **Preference set used in translation of raw results** | Not applicable |
| **Reported format** | Predicted Choice Probability (PCP) (SE) [95%CI]  This data comes the supplementary material (document 7) |
| **Result** | Scenario 1  LP: 18.2% (4.2%) [10.0%; 26.4%]  IDDD: 81.8% (4.2%) [73.6%; 90.0%]  Scenario 2:  LP: 31.1% (6.2%) [19.0%; 43.2%]  IDDD: 68.9% (6.2%) [56.8%; 81.0%]  Scenario 3:  LP: 27.0% (4.6%) [18.1%; 36.0%]  IDDD: 73.0% (4.6%) [64.0%; 81.9%]  Scenario 4:  LP: 48.0% (6.9%) [34.5%; 61.5%]  IDDD: 52.0% (6.9%) [38.5%; 65.5%]  The authors also report in the text of their results that “An IDDD option was preferred over lumbar puncture in 78.7% of DCE choice tasks” |
| **OUTCOME 2: MAXIMUM ACCEPTABLE RISK FOR IDDD (TT)** | |
| **Instrument or method** | In the threshold technique exercise, participants were asked to choose between the first IDDD that they had selected in the original DCE choice task and the lumbar puncture option from that same DCE choice task.  The implantation risk appeared on the threshold technique exercise as an additional attribute and was varied iteratively to determine the risk level at which a participant would switch their preference to choose lumbar puncture over the IDDD.  The implantation risk was increased incrementally from 1% up to 30% (1%, 10%, 20%, 30%), until the participant chose lumbar puncture over the IDDD. |
| **Preference set used in translation of raw results** | Not applicable |
| **Reported format** | Average maximum acceptable risk (MAR) (SE) [95%CI] |
| **Result** | 0.290 (0.014) [0.264 – 0.317] (Derived from supplementary material 7  Also reported in text of results that “On average, participants were willing to accept up to a 29.0% risk of complications from the implantation surgery. |

**Risk of bias**

| Subdomain and signalling question | Judgement | Notes |
| --- | --- | --- |
| **Selection of participants** | Not serious |  |
| Was an appropriate study sample selected from the study’s sampling frame? | Yes | Participants were conveniently sampled from physician referral, patient advocacy groups and through advertising via social media |
| **Completeness of data** | Not serious |  |
| Was the attrition rate sufficiently low to minimise the risk of bias? | Yes | 328 people were invited and 295 people provided outcome data. Attrition rate is sufficient low |
| **Measurement instrument** | Not serious |  |
| Was the instrument used to measure patient values and preferences in a valid and reliable manner? | Yes | A discrete choice and threshold technique were utilised which are both appropriate designs to measures patient values and preferences regarding an intervention |
| Was the instrument used in the intended way? | Yes | Both techniques were rigorously pilot tested over 4 unique phases. |
| Was a valid representation of the outcome (health state) used? | Yes | The outcome (from both lumbar puncture and IDDD) is assumed to be “better health” as it was never made explicit.  According to the authors “After providing consent, participants were shown text asking them to imagine a situation in which their physician advised that they would need regular injections of medicine into the intrathecal space.  Participants were presented with descriptions of IDDD and lumbar puncture options for intrathecal drug delivery, an illustration of each option, and descriptions of the attributes used in the survey.” |
| Did the researchers check for understanding of the instrument? | Investigators tested the understanding, and understanding was adequate | Both the DCE and TT were piloted tested on patients across multiple countries. There were multiple rounds in which feedback was collected and integrated into the exercises. |
| **Data Analysis** | Not serious |  |
| Were the results analysed appropriately to avoid influence of bias and confounding? | Yes | Mixed logit models have been utilised appropriately |
| **Overall risk of bias** | Not serious |  |

**Stenson 2024**

**Data extraction**

| Study ID | Stenson 2024 |
| --- | --- |
| **DESIGN** | |
| **Other reports of this study identified?** | None identified |
| **Time period study was conducted** | July 2020 – March 2021 |
| **Design** | Cross sectional survey. Data were sourced from the Adelphi ALS Disease Specific Programme (DS) a large, prospective, cross-sectional survey of neurologists, pALS and cALS presenting in a real-world clinical setting.  Using the EQ-5D-5L and ALSAQ-40 to assess overall health state at different disease stages (according to both King’s and MiToS). These were compared with the judgment of physicians (e.g. proxy measure of patient health states) who assessed the patients as being early, middle or late stage of the disease based off their clinical judgement. |
| **Outcomes with a valuation provided relevant to the review** | Health-related quality of life (EQ-5D-5L)  Health-related quality of life (EQ-5D VAS)  Health-related quality of life (ALSAQ-5*) [Adapted from ALSAQ-40].  *Note – in the supplementary material, the authors have provided the breakdown per domain. However, these are simple n(%) data, used for assignment of the 5-digit health code. These have not been translated and are not appropriate to be extracted/utilised. |
| **SETTING** | |
| **Country** | United states, France Germany, Italy, Spain, United Kingdom |
| **Sites/s** | Outpatient neurology practices |
| **Study locale** | Community-based outpatient settings |
| **Disease investigated** | ALS |
| **PARTICPANTS** | |
| **Category of participants under investigation** | Adults diagnosed with MND  Clinicians providing proxy measures of adults diagnosed with MND  And their carers |
| **Eligibility criteria of participants** | Adults with ALS => 18 years with a physician-confirmed diagnosis of ALS  Clinicians were identified by local fieldwork agents using physician panels and publicly available lists and invited to participate if they had a primary specialty of neurology (general neurologist, neuromuscular specialist, or ALS specialist), were actively involved in the management of ALS, were seeing two or more pALS per month, and agreed to adhere to all survey rules and regulations. |
| **Sampling strategy** | Clinicians as above  pALS were consecutively invited to complete the surveys |
| **Number of participants included** | 142 clinicians completed patient record forms for 880 pALS.  Of these, 172 pALS had self-reported data. This is the data used in this study. |
| **Characteristics/demographics of participants included** | The demographics of the clinicians was not provided. Below is the demographics of pALS Characteristic \| Value; **Country** \|; France \| 33 (19.2%); Germany \| 19 (11.1%); Italy \| 27 (15.7%); Spain \| 42 (24.4%); UK \| 8 (4.7%); US \| 43 (25.0%); **Age** \|; Mean (SD) \| 60.8 (11.5); Range \| 22.0–90.0; Gender (male) \| 104 (60.5%); **BMI** \|; Mean (SD) \| 23.7 (3.0); Range \| 11.3–29.4; Underweight \| 9 (5.2%); Healthy weight \| 106 (61.6%); Overweight \| 57 (33.1%); Obese \| 0 (0.0%); **Ethnicity** \|; White/Caucasian \| 161 (93.6%); Hispanic/Latino/Latina \| 8 (4.7%); Other \| 2 (1.2%); **Living circumstances** \|; Lives alone in own home \| 15 (8.8%); Lives with partner/spouse/immediate family \| 141 (82.5%); Lives with other family/friends \| 8 (4.7%); Lives in hospice \| 1 (0.6%); Lives in nursing home \| 5 (2.9%); Lives in assisted living residence/residential home \| 1 (0.6%); **Employment status** \|; Working full time \| 13 (7.7%); Working part time \| 21 (12.4%); On long term sick leave \| 32 (18.8%); Homemaker \| 13 (7.7%); Student \| 1 (0.6%); Retired \| 81 (47.7%); Unemployed \| 9 (5.3%); **Number of comorbidities** \|; Mean (SD) \| 1.5 (1.4); Range \| 0.0–7.0; **Time since diagnosis (months)** \|; Mean (SD) \| 22.0 (29.2); Range \| 0.0–221.4; **ALSFRS-R score** \|; Mean (SD) \| 32.7 (12.4); Range \| 0.0–48.0; **ALS stage – physician judgement** \|; Early stage \| 64 (37.2%); Middle stage \| 76 (44.2%); Late stage \| 32 (18.6%); **ALS stage – MiToS** \|; Stage 0 \| 122 (70.9%); Stage 1 \| 18 (10.5%); Stage 2 \| 10 (5.8%); Stage 3 \| 8 (4.7%); Stage 4 \| 14 (8.1%); **ALS stage – King’s** \|; Stage 1 \| 29 (17.2%); Stage 2 \| 27 (16.0%); Stage 3 \| 61 (36.1%); Stage 4a/b \| 52 (30.8%) |
| **Response rate** | Not reported |
| **OUTCOME 1: HEALTH-RELATED QUALITY OF LIFE (EQ-5D-5L)** | |
| **Instrument or method** | EQ-5D-5L  *Note – in the supplementary material, the authors have provided the breakdown per domain. However, these are simple n(%) data, used for assignment of the 5-digit health code. These have not been translated and are not appropriate to be extracted/utilised. |
| **Preference set used in translation of raw results** | Hernandez-Alava UK value set |
| **Reported format** | This data comes directly from figure 1. The score and n have been provided, and while confidence intervals are indicated on the figure the values of these have not been reported. |
| **Result** | Physician judgment:  Early: 0.61 (n-61)  Middle: 0.42 (n=72)  Late: -0.05 (n=32)  King’s Staging  1: 0.65 (n =29)  2: 0.51 (n = 27)  3: 0.45 (n = 56)  4: 0.11 (n = 50)  MiToS Staging:  0: 0.53 (n = 116)  1: 0.34 (n = 17)  2: 0.00 (n = 10)  3: 0.01 (n = 8)  4: -0.10 (n = 14) |
| **OUTCOME 2: HEALTH-RELATED QUALITY OF LIFE (EQ-5D VAS)** | |
| **Instrument or method** | EQ-5D VAS |
| **Preference set used in translation of raw results** | Hernandez-Alava UK value set |
| **Reported format** | This data comes directly from figure. The score and n have been provided, and while confidence intervals are indicated on the figure the values of these have not been reported. |
| **Result** | Physician judgment:  Early: 56.9 (n = 64)  Middle: 45 (n=76)  Late: 26.8 (n=32)  King’s Staging  1: 59.9 (n=29)  2: 52.0 (n=27)  3: 46.9 (n=61)  4: 31.6 (n=52)  MiToS Staging:  0: 51.6 (n=122)  1: 39.9 (n=18)  2: 29.4 (n=10)  3: 31.2 (n=8)  4: 25.7 (n=14) |
| **OUTCOME 3: HEALTH-RELATED QUALITY OF LIFE (ALSAQ-5*) [ADAPTED FROM ALSAQ-40]** | |
| **Instrument or method** | ALSAQ-5 The 40-item Amyotrophic Lateral Sclerosis Assessment Questionnaire (ALSAQ-40) is a 40-item questionnaire used to specifically measure the HRQoL of pALS and motor neuron disease. The 5-item ALSAQ (ALSAQ-5) used in the present study is an abbreviated version of the ALSAQ-40, with five items (physical mobility, activities of daily living, eating/drinking, communication, and emotional functioning), each representing one domain of the longer form. The ALSAQ-5 is useful in surveys and trials, as it produces very similar results to the ALSAQ-40. The overall ALSAQ-5 score ranges from 0 (best imaginable health state) to 100 (worst imaginable health state). |
| **Preference set used in translation of raw results** | Hernandez-Alava UK value set |
| **Reported format** | This data comes directly from figure 3. The score and n have been provided, and while confidence intervals are indicated on the figure the values of these have not been reported. |
| **Result** | Physician judgment:  Early: 43.7 (n=60)  Middle: 52.6 (n=63)  Late: 52.6 (n=8)  King’s Staging  1: 39.7 (n=25)  2: 47.9 (n=26)  3: 50.9 (n=49)  4: 54 (n=29)  MiToS Staging:  0: 47.2 (n=108)  1: 55.0 (n=13)  2: 54.9 (n=4)  3: 53.5 (n=5)  4: N/A (n=0) |

**Risk of bias**

| Subdomain and signalling question | Judgement | Notes |
| --- | --- | --- |
| **Selection of participants** | Not serious |  |
| Was an appropriate study sample selected from the study’s sampling frame? | Probably yes | The data utilised in this study were sourced from a previous prospective cross-sectional survey of neurologists, pALS and cALS. pALS and cALS were consecutively invited to the surveys from outpatient neurology clinics.  The clinicians were invited by local fieldwork agents using physician panels and publicly available lists. |
| **Completeness of data** | Not serious |  |
| Was the attrition rate sufficiently low to minimise the risk of bias? | Probably yes | There is some missingness in the data, which is unexplained, but the missingness is very small  122 recruited versus 116 to provide data (MiToS stage 0)  18 recruited versus 17 to provide data (MiToS stage 1)  61 recruited versus 56 to provide data (King’s stage 3)  52 recruited versus 50 to provide data (King’s stage 4) |
| **Measurement instrument** | Not serious |  |
| Was the instrument used to measure patient values and preferences in a valid and reliable manner? | Yes | The EQ-5D-5L, EQ-5D VAS and ALSAQ-5 are validated instruments and have been utilised appropriately to assess health states |
| Was the instrument used in the intended way? | Yes | Surveys completed according to standardised instructions. These surveys were self-completed however caregiver assistance was noted. |
| Was a valid representation of the outcome (health state) used? | Yes | Health states presented in the EQ-5D-5L and ALSAQ ask the user to reflect on their current health and understanding therefore this is valid. |
| Did the researchers check for understanding of the instrument? | Investigators did not formally test the understanding, but evidence suggested adequate understanding | No specific mention of cognitive testing or piloting, however these are well used and validated tools which addresses some concerns. |
| **Data Analysis** | Not serious |  |
| Were the results analysed appropriately to avoid influence of bias and confounding? | Yes | Linear regression adjusting for age, sex, BMI, comorbidities was performed |
| **Overall risk of bias** | Not serious |  |

**Tarlarini 2017**

**Data extraction**

| Study ID | Tarlarini 2017 |
| --- | --- |
| **DESIGN** | |
| **Other reports of this study identified?** | None identified |
| **Time period study was conducted** | July – December 2017 |
| **Design** | This was an observational, pre-post cohort study evaluating the effect of a flavour device on taste perception and QoL in pALS with dysphagia.  However, the outcomes in which a valuation have been provided have been collected in section 1 (detailed below) and section 3 (below).  A specific device that provides selected flavours of natural meals obtained by molecular cuisine was created and used for this study. This device, previously tested in healthy volunteers, allows to perceive in a safety manner flavours without the need to swallow.  The set of questionnaires included four sections, described as follows. Section 1 included a set of visual-analogue scales (VAS) to evaluate the degree of interest of pALS in the perception of flavours and the degree of interest of ET patients in food and flavours. Section 2 focused on the detection of taste and smell changes in pALS, using a questionnaire on chemosenses that measured the eventual loss or alteration of either taste, smell or both.  Section 3 evaluated the impact of dysphagia on the QoL of pALS, through the use of a questionnaire on QoL obtained from the already existing ‘University of Washington Quality of Life Questionnaire.  section 4, a VAS was used in order to evaluate the ability to identify the chosen flavours presented through the device and the degree of pleasantness. |
| **Outcomes with a valuation provided relevant to the review** | How much is flavour perception important for you?  Pain  Swallowing  Speech  Taste  Chewing  Appearance  Activity  Recreation  Saliva  Mood  Anxiety |
| **SETTING** | |
| **Country** | Italy |
| **Sites/s** | NEuroMuscular Omnicenter (NEMO), Milan |
| **Study locale** | Inpatient and outpatient ALS clinics |
| **Disease investigated** | ALS |
| **PARTICPANTS** | |
| **Category of participants under investigation** | Adults with MND |
| **Eligibility criteria of participants** | Clinical diagnosis of ALS, presence of dysphagia with an ALSFRS-R swallowing score of <4 (excluded if no swallowing impairment). |
| **Sampling strategy** | Consecutive sampling of 32 patients with ALS attending inpatients and outpatient facility at NEMO. |
| **Number of participants included** | N=31 (The authors report 32 in the text, but the table (below) indicates 31? This is not made clear in the text of the report).  Enteral Tube-fed = 10  Oral way-fed = 21 |
| **Characteristics/demographics of participants included** | \| Total sample (n = 31) \| Patients with ET (n = 10) \| Patients without ET (n = 21) \| p value; Age: years, mean ± std \| 62.77 ± 11.39 \| 60.23 ± 10.60 \| 63.97 ± 11.80 \| 0.3207; Gender; male/female, n (%) \| 19/13 \| 6/4 \| 13/8 \| 0.9188; Site of onset; bulbar, n (%) \| 7 (22.58) \| 2 (20.00) \| 5 (23.81) \| 0.5778; Disease Duration: months, mean ± std \| 58.97 ± 41.88 \| 76.26 ± 44.89 \| 50.73 ± 38.78 \| 0.0725; ALSFRS-R total, mean ± std \| 16.00 ± 12.17 \| 4.00 ± 2.36 \| 22.32 ± 10.28 \| < 0.0001*; ALSFRS-R bulbar, mean ± std \| 5.42 ± 3.36 \| 2.90 ± 2.02 \| 6.62 ± 3.23 \| 0.0033*; NIV; yes (%), n (%) \| 21 (67.74) \| 9 (90.00) \| 11 (52.38) \| 0.0134*; Tracheo; yes (%), n (%) \| 9 (29.03) \| 6 (60.00) \| 2 (9.52) \| 0.0059* |
| **Response rate** |  |
| **OUTCOME 1: HOW MUCH IS FLAVOUR PERCEPTION IMPORTANT FOR YOU?** | |
| **Instrument or method** | A set of visual-analogue scales (VAS) to evaluate the degree of interest of pALS in the perception of flavours and the degree of interest of ET patients in food and flavours. Score 0  corresponded to ‘little’ and 100 corresponded to ‘a lot’ |
| **Preference set used in translation of raw results** | Not applicable |
| **Reported format** | Narrative |
| **Result** | Directly from the authors: “The analyses of section 1 showed that food perception is an important issue in our patients, as demonstrated by the answers at the question ‘How much is flavour perception important for you?’: 24 out of 32 patients (75%) reported a score equal or higher than 75.” |
| **OUTCOME 2: PAIN** | |
| **Instrument or method** | The importance of 11 different aspects on QoL with the score ranging from 0 (worst possible score) to 100 (best possible score). |
| **Preference set used in translation of raw results** | NA |
| **Reported format** | Average value as percentage |
| **Result** | Patients with ET = 9.09  Patients without ET = 9.52 |
| **OUTCOME 3: SWALLOWING** | |
| **Instrument or method** | As above |
| **Preference set used in translation of raw results** | As above |
| **Reported format** | As above |
| **Result** | Patients with ET = 100  Patients without ET = 28.57 |
| **OUTCOME 4: SPEECH** | |
| **Instrument or method** | As above |
| **Preference set used in translation of raw results** | As above |
| **Reported format** | As above |
| **Result** | Patients with ET = 90.91  Patients without ET = 42.86 |
| **OUTCOME 4: TASTE** | |
| **Instrument or method** | As above |
| **Preference set used in translation of raw results** | As above |
| **Reported format** | As above |
| **Result** | Patients with ET = 54.55  Patients without ET = 76.19 |
| **OUTCOME 5: CHEWING** | |
| **Instrument or method** | As above |
| **Preference set used in translation of raw results** | As above |
| **Reported format** | As above |
| **Result** | Patients with ET = 90.91  Patients without ET = 9.52 |
| **OUTCOME 6: APPEARANCE** | |
| **Instrument or method** | As above |
| **Preference set used in translation of raw results** | As above |
| **Reported format** | As above |
| **Result** | Patients with ET = 54.55  Patients without ET = 4.76 |
| **OUTCOME 7: ACTIVITY** | |
| **Instrument or method** | As above |
| **Preference set used in translation of raw results** | As above |
| **Reported format** | As above |
| **Result** | Patients with ET = 36.36  Patients without ET = 47.62 |
| **OUTCOME 8: RECREATION** | |
| **Instrument or method** | As above |
| **Preference set used in translation of raw results** | As above |
| **Reported format** | As above |
| **Result** | Patients with ET = 45.45  Patients without ET = 52.38 |
| **OUTCOME 9: SALIVA** | |
| **Instrument or method** | As above |
| **Preference set used in translation of raw results** | As above |
| **Reported format** | As above |
| **Result** | Patients with ET = 18.18  Patients without ET = 4.76 |
| **OUTCOME 10: MOOD** | |
| **Instrument or method** | As above |
| **Preference set used in translation of raw results** | As above |
| **Reported format** | As above |
| **Result** | Patients with ET = 36.36  Patients without ET = 38.10 |
| **OUTCOME 11: ANXIETY** | |
| **Instrument or method** | As above |
| **Preference set used in translation of raw results** | As above |
| **Reported format** | As above |
| **Result** | Patients with ET = 45.45  Patients without ET = 28.57 |

**Risk of bias**

| Subdomain and signalling question | Judgement | Notes |
| --- | --- | --- |
| **Selection of participants** | Not serious |  |
| Was an appropriate study sample selected from the study’s sampling frame? | Yes | Consecutive sampling of patients attending inpatient and outpatient facility at NEMO |
| **Completeness of data** | Serious |  |
| Was the attrition rate sufficiently low to minimise the risk of bias? | Probably no | The reporting of participant numbers is inconsistent, and no explanation is provided by the authors as this discrepancy. Methods reports 32 patients, results are presented for 32 patients, but characteristics of only 31 patients are provided. |
| **Measurement instrument** | Very serious |  |
| Was the instrument used to measure patient values and preferences in a valid and reliable manner? | Probably no | The outcome valuation in this study was a single question delivered via a survey. The authors mention that ‘interviews’ were conducted (where it is assumed these surveys were administered), but too few methodological details have been provided (e.g. the survey questions were not provided, just a summary of the ‘type’ of questions included). |
| Was the instrument used in the intended way? | Probably yes | It looks like the survey was administered in a standard way for all participants |
| Was a valid representation of the outcome (health state) used? | Yes | The question posed “how much is flavour perception important for you” is directly related to participant perception of this outcome. |
| Did the researchers check for understanding of the instrument? | Investigators did not formally test the understanding, but evidence suggested adequate understanding | No mention of piloting or cognitive testing of survey questions |
| **Data Analysis** | Extremely |  |
| Were the results analysed appropriately to avoid influence of bias and confounding? | No | Only raw descriptive data presented. |
| **Overall risk of bias** |  |  |

**Thakore 2019**

**Data extraction**

| Study ID | Thakore 2019 |
| --- | --- |
| DESIGN | |
| **Other reports of this study identified?** | None identified |
| **Time period study was conducted** | Data was collected from routine clinical care over 2010 – 2015 (from Appendix 1) Modelling was performed based on this data |
| **Design** | Health economic modelling study using a Markov model of riluzole treatment for ALS |
| **Outcomes with a valuation provided relevant to the review** | Health-related quality of life (EQ-5D-3L and VAS but reported together)  This has been sub-grouped based on bulbar-onset patients and limb-onset patients and has been separated based on FT9 staging. |
| **SETTING** | |
| **Country** | United States |
| **Sites/s** | Not made explicit, so assumed to be the ‘Cleveland Clinic’ as per the affiliations of the authors and the following text from the methods “Self-reported measures including ALSFRS-R and EQ-5D (3-level) were obtained at our institution from patients with ALS”. |
| **Study locale** | Outpatient ALS clinic |
| **Disease investigated** | ALS |
| **PARTICPANTS** | |
| **Category of participants under investigation** | Adults with MND |
| **Eligibility criteria of participants** | Not stated, these are patients from the Cleveland Clinic over 10 years of observations. The patients must have completed both the EQ-5D-3L and the ALSFRS-R to have their data included. |
| **Sampling strategy** | Convenience sampling of patients at the above clinic |
| **Number of participants included** | 678 patients were included, of which 1868 observations were taken (same patient may have been observed multiple times). |
| **Characteristics/demographics of participants included** | No data regarding the characteristics of the demographics of the included participants was provided. |
| **Response rate** | Not provided and incalculable as only patients that provided eligible data were included. |
| **OUTCOME 1: HEALTH-RELATED QUALITY OF LIFE** | |
| **Instrument or method** | EQ-5D-3L (VAS also used reported below)  The EQ-5D provides a utility-based summary measure of health-related quality of life, derived by aggregating responses across five dimensions of health using a population-based value set. The 3-level set was used in these results |
| **Preference set used in translation of raw results** | US Valuation (D1 valuation model) |
| **Reported format** | Mean (se) (values directly from Appendix 1) |
| **Result** | FT9 stage \| n \| Mean EQ-5D \| SE (EQ-5D) \| Mean EQ-5D (bulbar-onset) \| Mean EQ-5D (limb-onset) \| Mean VAS \| SE (VAS) \| Mean VAS (bulbar onset) \| Mean VAS (limb-onset); **0** \| 160 \| 0.789 \| 0.013 \| 0.852 \| 0.775 \| 72.9 \| 1.7 \| 79.9 \| 70.5; **1** \| 494 \| 0.743 \| 0.009 \| 0.826 \| 0.716 \| 66.1 \| 1.1 \| 72.7 \| 63.5; **2** \| 542 \| 0.629 \| 0.010 \| 0.741 \| 0.592 \| 56.9 \| 1.0 \| 61.9 \| 55.4; **3** \| 381 \| 0.541 \| 0.012 \| 0.657 \| 0.500 \| 51.2 \| 1.2 \| 56.9 \| 49.3; **4** \| 291 \| 0.464 \| 0.015 \| 0.568 \| 0.421 \| 45.2 \| 1.3 \| 50.0 \| 43.3 |

**Risk of bias**

| Subdomain and signalling question | Judgement | Notes |
| --- | --- | --- |
| **Selection of participants** | Not serious |  |
| Was an appropriate study sample selected from the study’s sampling frame? | Probably yes | Consecutive sampling. Health state utilities came from patients who attended the Cleveland clinic for routine clinical care while they sat in clinic waiting rooms. |
| **Completeness of data** | Not serious |  |
| Was the attrition rate sufficiently low to minimise the risk of bias? | Yes | No missingness as the authors used retrospective data from patients that had provided full responses to both ALSFRS-R and EQ-5D-3L |
| **Measurement instrument** | Not serious |  |
| Was the instrument used to measure patient values and preferences in a valid and reliable manner? | Yes | EQ-5D-3L was used with the US valuation preference set |
| Was the instrument used in the intended way? | Yes | Collected as part of routine clinical care, through a tablet while patients attended a clinic. |
| Was a valid representation of the outcome (health state) used? | Yes | Health states as presented in the EQ-5D-3L are directly valued outcomes based on patient perception |
| Did the researchers check for understanding of the instrument? | Investigators did not formally test the understanding, but evidence suggested adequate understanding | No cognitive interviewing or pilot testing reported |
| **Data Analysis** | Not serious |  |
| Were the results analysed appropriately to avoid influence of bias and confounding? | Yes | Mean EQ-5D-3L and VAS were obtained using generalised estimating equations to adjust for within-patient correlation. |
| **Overall risk of bias** | Not serious |  |

**Tobin 2021**

**Data extraction**

| Study ID | Tobin 2021 |
| --- | --- |
| **DESIGN** | |
| **Other reports of this study identified?** | None identified |
| **Time period study was conducted** | April 2014 – May 2015 |
| **Design** | Discrete choice experiment with nice choice sets, developed to measure the preferences for health services of ALS patients and their caregivers and the relative importance of various aspects of cares. The attribute levels were randomised into choice sets using the rotation design method |
| **Outcomes with a valuation provided relevant to the review** | Disease information  Price of additional services  Arranging visits  Distant to clinic  Waiting times  Place of care  Decisions on care  Personal care provision  Timing of hospice care (adults diagnosed with MND only)  Communication devices (adults diagnosed with MND only)  Availability of phone advice  Provision of emotional support  Dependable Healthcare processionals  Who helps in the home |
| **SETTING** | |
| **Country** | Ireland |
| **Sites/s** | The site is not named specifically; author affiliations suggest Dublin. |
| **Study locale** | National multidisciplinary clinic |
| **Disease investigated** | ALS |
| **PARTICPANTS** | |
| **Category of participants under investigation** | Adults with MND and Informal caregivers of adults with MND |
| **Eligibility criteria of participants** | Adults over the age of 18, patients with ALS that fulfilled the EL Escorial Diagnostic Criteria, were cognitively normal using the Edinburg Cognitive and Behavioural ALS Screen.  Caregivers had to be over the age of 18 and whose patient was participating in the study. |
| **Sampling strategy** | Consecutive sampling. Participants were recruited during routine clinic appointments |
| **Number of participants included** | Patients n = 93  Caregivers n = 56 |
| **Characteristics/demographics of participants included** | Characteristics \| Patients (N = 93) n(%) \| Caregivers (N = 56) n(%); Sex: Male \| 55 (59.1) \| 21 (37.5); Mean age (range) \| 61 (25–86) \| 51 (28–78); Caregiver relationship to patient: \| \|; Spouse/partner \| – \| 36 (64.3); Child of patient \| – \| 17 (30.4); Other \| – \| 3 (5.4); Site of onset: \| \|; Bulbar \| 23 (24.7) \| –; Spinal \| 69 (74.1) \| –; Cognitive \| 1 (1.1) \| –; Median time in months since diagnosis (Q1, Q3) \| 12.2 (5.5, 30.6) \| –; Diagnosis ≤ 6 months \| 23 (24.7) \| –; Diagnosis > 6 months \| 70 (75.3) \| –; King’s Clinical Staging: \| \|; Stage 1 or 2 \| 26 (28.0) \| –; Stage 3 or 4 \| 62 (66.7) \| – |
| **Response rate** | All participants that were recruited provided full data |
| **OUTCOME 1: DISEASE INFORMATION** | |
| **Instrument or method** | Consenting participants who met the eligibility criteria were presented with a paper copy of the experiment and asked to give their preferred option from each of the scenarios or choice set presented.  Each choice set consisted of (at least) two hypothetical packages of care, and participants were asked to choose the package that they preferred. This process was repeated nine times with each participant. |
| **Preference set used in translation of raw results** | Not applicable |
| **Reported format** | Random effects probit model was applied to estimate the impact of each attribute on a participant’s choice. The model estimates the coefficient for each attribute level, and the magnitude of the coefficient is related to the tendency for participants to choose that attribute level. A large positive coefficient indicates that study participants preferred that level of the attribute, while a negative coefficient indicates a negative preference for an attribute level. The magnitude of attribute coefficients and their associated 95% confidence interval were used as an indicator of the strength of participant preferences. |
| **Result** | A1. I would get all the information about motor neuron disease at the time of diagnosis (reference)  A2. I would get all information about motor neuron disease when I think I will need it  A3. I don’t want to know anything about motor neuron disease at any time  Patients strongly preferred receipt of all information about ALS at the time of diagnosis, rather than when they consider the information to be personally relevant (coefficient: -0.22, 95% CI: − 0.44, − 0.01).  Subgroups: Directly from supplementary material:  Grouping by stage of disease, patients who were in earlier stages of disease (King’s stage 1 or 2) showed a strong preference for receipt of extensive information about ALS at the time of diagnosis, rather than at a later stage (when clinically relevant)(coefficient: -0.51, 95% CI: − 0.99, − 0.05), or not receiving relevant information at any time during their illness (coefficient: -0.57, 95% CI: − 1.05, − 0.10).  Males:  A2: -0.2 (-0.48, 0.08)  A3: -0.24 (-0.54, 0.05)  Females  A2: -0.21 (-0.56, 0.14)  A3: -0.06 (-0.42, 0.29)  Bulbar Onset  A2: -0.34 (-0.78, 0.10)  A3: -0.26 (-0.71, 0.20)  Spinal Onset  A2: -0.19 (-0.44, 0.06)  A3: -0.15 (-0.41, 0.11)  Diagnosis ≤ 6 months  A2: -0.21 (-0.67, 0.24)  A3: -0.19 (-0.65, 0.27)  **Diagnosis >6 months**  **A2: -0.25 (-0.50, -0.01)**  A3: -0.24 (-0.50, 0.01)  **King’s Stage 1 or 2**  **A2: -0.51 (-0.99, -0.05)**  **A3: -0.57 (-1.04, -0.10)**  King’s Sage 3 or 4  A2: -0.12 (-0.37, 0.13)  A3: -0.05 (-0.31, 0.21) |
| **OUTCOME 2: PRICE OF ADDITIONAL SERVICES** | |
| **Instrument or method** | As above |
| **Preference set used in translation of raw results** | As above |
| **Reported format** | As above |
| **Result** | All other attributes had small coefficients with wide confidence intervals, indicating that patients showed no strong preference for their inclusion in their healthcare package. |
| **OUTCOME 3: ARRANGING VISITS** | |
| **Instrument or method** | As above |
| **Preference set used in translation of raw results** | As above |
| **Reported format** | As above |
| **Result** | C1. I would get regular visits from community multidisciplinary team (for e.g. physiotherapy, occupational therapist) and the public health nurse in my home (reference)  C2. I would arrange multidisciplinary team (for e.g. physiotherapy, occupational therapist) and public health nurse visits as I need in my home  All other attributes had small coefficients with wide confidence intervals, indicating that patients showed no strong preference for their inclusion in their healthcare package.  Directly from supplementary material:  Males:  C2: 0.03 (-0.17, 0.22)  Females  C2: -0.09 (-0.33, 0.14)  Bulbar Onset  C2: 0.19 (-0.12, 0.51)  Spinal Onset  C2: 0.01 (-0.16, 0.19)  Diagnosis ≤ 6 months  C2: 0.05 (-0.26, 0.35)  Diagnosis >6 months  C2: 0.02 (-0.16, 0.19)  King’s Stage 1 or 2  C2: 0.25 (-0.05, 0.56)  King’s Sage 3 or 4  C2: 0.03 (-0.15, 0.20) |
| **OUTCOME 4: DISTANT TO CLINIC** | |
| **Instrument or method** | As above |
| **Preference set used in translation of raw results** | As above |
| **Reported format** | As above |
| **Result** | This choice was presented in the study, but no results were reported regarding it. |
| **OUTCOME 5: WAITING TIMES** | |
| **Instrument or method** | As above |
| **Preference set used in translation of raw results** | As above |
| **Reported format** | As above |
| **Result** | E1. I would be prepared to go to a multidisciplinary clinic with long waiting times in the clinic to see more than one professional (reference)  E2. I would not be prepared to go to a multidisciplinary clinic with long waiting times in the clinic to see more than one professional  All other attributes had small coefficients with wide confidence intervals, indicating that patients showed no strong preference for their inclusion in their healthcare package.  Subgroups: Directly from supplementary material:  Males:  E2: 0.09 (-0.13, 0.32)  Females  E2: -0.03 (-0.30, 0.25)  Bulbar Onset  E2: 0.05 (-0.29, 0.39)  Spinal Onset  E2: 0.04 (-0.16, 0.24)  Diagnosis ≤ 6 months  E2: 0.27 (-0.09, 0.64)  Diagnosis >6 months  E2: 0.00 (-0.20, 0.20)  King’s Stage 1 or 2  E2: -0.05 (-0.39, 0.29)  King’s Sage 3 or 4  E2: 0.06 (-0.14, 0.27) |
| **OUTCOME 6: PLACE OF CARE** | |
| **Instrument or method** | As above |
| **Preference set used in translation of raw results** | As above |
| **Reported format** | As above |
| **Result** | F1. No matter what I would like to receive all my care at home (reference)  F2. I would receive care in a hospital or a hospice as an inpatient  All other attributes had small coefficients with wide confidence intervals, indicating that patients showed no strong preference for their inclusion in their healthcare package.  Subgroups: Directly from supplementary material:  Males:  F2: -0.09 (-0.29, 0.11)  Females  F2: -0.02 (-0.27, 0.22)  Bulbar Onset  F2: 0.02 (-0.29, 0.33)  Spinal Onset  F2: -0.07 (-0.25, 0.11)  Diagnosis ≤ 6 months  F2: -0.08 (-0.24, 0.04)  Diagnosis >6 months  F2: -0.06 (-0.24, 0.12)  King’s Stage 1 or 2  F2: 0.08 (-0.22, 0.38)  King’s Sage 3 or 4  F2: -0.09 (-0.28, 0.09) |
| **OUTCOME 7: DECISIONS ON CARE** | |
| **Instrument or method** | As above |
| **Preference set used in translation of raw results** | As above |
| **Reported format** | As above |
| **Result** | G1. The doctors advise and help me when I need treatments including ventilation and stomach tube (reference)  G2. The doctors advise me, and I would decide what treatments I get including ventilation and stomach tube  All other attributes had small coefficients with wide confidence intervals, indicating that patients showed no strong preference for their inclusion in their healthcare package.  Subgroups: Directly from supplementary material:  Males:  G2: -0.15 (-0.34, 0.04)  Females  G2: -0.05 (-0.27, 0.17)  Bulbar Onset  G2: -0.19 (-0.47, 0.11)  Spinal Onset  G2: -0.08 (-0.25, 0.08)  Diagnosis ≤ 6 months  G2: -0.35 (-0.67, -0.05)  Diagnosis >6 months  G2: -0.07 (-0.23, 0.10)  King’s Stage 1 or 2  G2: -0.19 (-0.47, 0.10)  King’s Sage 3 or 4  G2: -0.07 (-0.25, 0.10) |
| **OUTCOME 8: PERSONAL CARE PROVISION** | |
| **Instrument or method** | As above |
| **Preference set used in translation of raw results** | As above |
| **Reported format** | As above |
| **Result** | H1. Personal care in the home is provided to me by someone who is not a relative or friend (reference)  H2. Personal care in the home is provided by a relative or friend  All other attributes had small coefficients with wide confidence intervals, indicating that patients showed no strong preference for their inclusion in their healthcare package.  Subgroups: Directly from supplementary material:  Males:  H2: -0.01 (-0.25, 0.26)  Females  H2: -0.16 (-0.47, 0.16)  Bulbar Onset  H2: -0.19 (-0.58, 0.21)  Spinal Onset  H2: -0.01 (-0.24, 0.22)  Diagnosis ≤ 6 months  H2: -0.28 (-0.73, 0.17)  Diagnosis >6 months  H2: 0.00 (-0.22, 0.22)  King’s Stage 1 or 2  H2: -0.26 (-0.64, 0.13)  King’s Sage 3 or 4  H2: 0.04 (-0.19, 0.28) |
| **OUTCOME 9: TIMING OF HOSPICE CARE (ADULTS DIAGNOSED WITH MND ONLY)** | |
| **Instrument or method** | As above |
| **Preference set used in translation of raw results** | As above |
| **Reported format** | As above |
| **Result** | I1. I would start to see the Hospice Care team early on in my illness (reference)  I2. I would start to see the Hospice Care team when something serious happens later in my illness  A strong preference was placed on seeing the hospice care  team (attribute I) later rather than early on in the illness  (coefficient: 0.16, 95% CI: 0.02, 0.30).  Subgroups: Directly from supplementary material:  The subgroup of patients with spinal onset ALS showed a preference for delaying engagement with palliative/hospice services (coefficient: 0.17, 95% CI: 0.01, 0.33).  Males:  I2: 0.19 (-0.00, 0.37)  Females  I2: 0.15 (-0.08, 0.37)  Bulbar Onset  I2: 0.12 (-0.16, 0.40)  **Spinal Onset**  **I2: 0.17 (0.01, 0.33)**  Diagnosis ≤ 6 months  I2: 0.21 (-0.10, 0.51)  Diagnosis >6 months  I2: 0.13 (-0.03, 0.29)  King’s Stage 1 or 2  I2: 0.24 (-0.05, 0.53)  King’s Sage 3 or 4  I2: 0.14 (-0.03, 0.30) |
| **OUTCOME 10: COMMUNICATION DEVICES (ADULTS DIAGNOSED WITH MND ONLY)** | |
| **Instrument or method** | As above |
| **Preference set used in translation of raw results** | As above |
| **Reported format** | As above |
| **Result** | J1. I would use new communication technology including voice banking (reference)  J2. I would not use new communication technology including voice banking  Patients also indicated their willingness to consider the use of communication devices (attribute J) (coefficient: -0.14, 95% CI: − 0.29, − 0.00).  Subgroups: Directly from supplementary material:  When grouped by sex, female patients demonstrate a strong preference for using communication devices (coefficient: -0.25, 95% CI: − 0.47, − 0.21). Males did not show a strong preference for any attribute.  Males:  J2: -0.14 (-0.33, 0.05)  **Females**  **J2: -0.25 (-0.47, -0.02)**  Bulbar Onset  J2: -0.16 (-0.45, 0.12)  Spinal Onset  J2: -0.14 (-0.31, 0.03)  Diagnosis ≤ 6 months  J2: -0.01 (-0.33, 0.30)  Diagnosis >6 months  J2: -0.17 (-0.33, -0.00)  King’s Stage 1 or 2  J2: -0.04 (-0.32, 0.23)  King’s Sage 3 or 4  J2: -0.17 (-0.34, 0.01) |
| **OUTCOME 11: AVAILABILITY OF PHONE ADVICE** | |
| **Instrument or method** | As above |
| **Preference set used in translation of raw results** | As above |
| **Reported format** | As above |
| **Result** | K1. Phone advice is not available (reference)  K2. Phone advice is available 24hours a day  K3. Phone advice is available during the hours of 9am – 5pm Monday to Friday  All other attributes had small coefficients with wide confidence intervals, indicating that patients showed no strong preference for their inclusion in their healthcare package.  Subgroups: Directly from supplementary material:  Males:  K2: -0.06 (-0.31, 0.19)  K3: -0.06 (-0.25, 0.13)  Females  K2: 0.21 (-0.10, 0.53)  K3: -0.09 (-0.32, 0.13)  Bulbar Onset  K2: 0.01 (-0.37, 0.40)  K3: 0.04 (-0.25, 0.33)  Spinal Onset  K2: 0.05 (-0.18, 0.28)  K3: -0.13 (-0.30, 0.03)  Diagnosis ≤ 6 months  K2: -0.06 (-0.47, 0.34)  K3: -0.05 (-0.35, 0.24)  Diagnosis >6 months  K2: 0.00 (-0.22, 0.22)  K3: -0.11 (-0.28, 0.05)  King’s Stage 1 or 2  K2: 0.03 (-0.35, 0.41)  K3: 0.14 (-0.15, 0.43)  King’s Sage 3 or 4  K2: 0.05 (-0.18, 0.28)  K3: -0.17 (-0.33, 0.00) |
| **OUTCOME 12: PROVISION OF EMOTIONAL SUPPORT** | |
| **Instrument or method** | As above |
| **Preference set used in translation of raw results** | As above |
| **Reported format** | As above |
| **Result** | L1. Emotional support is not provided (reference)  L2. Emotional support is provided by group meetings with other MND patients  L3. Emotional support is provided from a counsellor  All other attributes had small coefficients with wide confidence intervals, indicating that patients showed no strong preference for their inclusion in their healthcare package.  Subgroups: Directly from supplementary material:  Males:  L2: 0.13 (-0.13, 0.38)  L3: 0.01 (-0.19, 0.21)  Females  L2: -0.01 (-0.31, 0.30)  L3: -0.07 (-0.31, 0.16)  Bulbar Onset  L2: 0.03 (-0.36, 0.42)  L3: -0.03 (-0.34, 0.28)  Spinal Onset  L2: 0.04 (-0.19, 0.26)  L3: 0.02 (-0.15, 0.20)  Diagnosis ≤ 6 months  L2: 0.16 (-0.23, 0.56)  L3: 0.03 (-0.30, 0.36)  Diagnosis >6 months  L2: 0.01 (-0.21, 0.24)  L3: 0.03 (-0.15, 0.20)  King’s Stage 1 or 2  L2: -0.02 (-0.41, 0.37)  L3: 0.31 (0.00, 0.62))  King’s Sage 3 or 4  L2: 0.05 (-0.17, 0.28)  L3: -0.06 (-0.24, 0.11) |
| **OUTCOME 12: DEPENDABLE HEALTHCARE PROCESSIONALS** | |
| **Instrument or method** | As above |
| **Preference set used in translation of raw results** | As above |
| **Reported format** | As above |
| **Result** | M1. I would have healthcare professionals who I can depend on (reference)  M2. I would not necessarily need healthcare professionals to depend on  All other attributes had small coefficients with wide confidence intervals, indicating that patients showed no strong preference for their inclusion in their healthcare package.  Subgroups: Directly from supplementary material:  Males:  M2: 0.12 (-0.12, 0.37)  Females  M2: 0.14 (-0.16, 0.44)  Bulbar Onset  M2: 0.15 (-0.22, 0.52)  Spinal Onset  M2: 0.08 (-0.14, 0.29)  Diagnosis ≤ 6 months  M2: 0.42 (0.02, 0.83)  Diagnosis >6 months  M2: 0.09 (-0.12, 0.30)  King’s Stage 1 or 2  M2: 0.36 (-0.00, 0.72)  King’s Sage 3 or 4  M2: 0.02 (-0.20, 0.25) |
| **OUTCOME: WHO HELPS IN THE HOME** | |
| **Instrument or method** | As above |
| **Preference set used in translation of raw results** | As above |
| **Reported format** | As above |
| **Result** | N1. I would have non-related individuals in my house to provide home help for my loved one (reference)  N2. I would not have non-related individuals in my house to provide home help for my loved one. All other attributes had small coefficients with wide confidence intervals, indicating that patients showed no strong preference for their inclusion in their healthcare package.  Subgroups: Directly from supplementary material:  Males:  N2: -0.19 (-0.47, 0.09)  Females  N2: 0.00 (-0.22, 0.22)  Bulbar Onset  N2: -0.03(-0.24, 0.18)  Spinal Onset  N2: -0.22 (-0.56, 0.11)  *Data not provided for diagnosis time or staging. |

**Risk of bias**

| Subdomain and signalling question | Judgement | Notes |
| --- | --- | --- |
| **Selection of participants** | Not serious |  |
| Was an appropriate study sample selected from the study’s sampling frame? | Yes | Consecutive sampling of eligible ALS patients and clinics during routine clinic appointments |
| **Completeness of data** | Not serious |  |
| Was the attrition rate sufficiently low to minimise the risk of bias? | Yes | No attrition, all consenting participants provided full data. |
| **Measurement instrument** | Not serious |  |
| Was the instrument used to measure patient values and preferences in a valid and reliable manner? | Yes | Discrete choice experiment that followed a rigorous design, that randomised choice sets using a ‘rotation design method’. |
| Was the instrument used in the intended way? | Yes | The Discrete choice experiment was administered consistently |
| Was a valid representation of the outcome (health state) used? | Yes | The scenarios presented in the DCE are directly relevant to their own MND experience |
| Did the researchers check for understanding of the instrument? | Investigators tested the understanding, and understanding was adequate | The DCE was piloted tested with patients and caregivers and iterative feedback was provided before the final DCE was administered. |
| **Data Analysis** | Not serious |  |
| Were the results analysed appropriately to avoid influence of bias and confounding? | Yes | Random effects probit model with rigours subgroup analysis was performed. Authors have fully and transparently reported all their data in the supplementary materials |
| **Overall risk of bias** | Not serious |  |

**Trail 2004**

**Data extraction**

| Study ID | Trail 2004 |
| --- | --- |
| **DESIGN** | |
| **Other reports of this study identified?** | None identified |
| **Time period study was conducted** | January – September 2021 |
| **Design** | Cross-sectional survey.  Patients and caregivers completed a 19-item survey where they were asked to rank their top 3 most important concerns |
| **Outcomes with a valuation provided relevant to the review** | Lack of family support  Worries about the future  Worries about being dependent on others  Loss of faith in religion  Lack of friendship or changes in friendships  Loneliness  Financial stresses  Emotional swings (laughing, crying, irritability)  Depression  Problems breathing  Problems speaking  Difficulty with self-care (i.e. dressing, bathing)  Difficulty with participating in hobbies/leisure activities  Difficulty at work or inability to work  Problems swallowing/eating  Worries about family/loves ones’ emotional and physical well-being  Muscle weakness  Progression of illness  Problems with mobility |
| **SETTING** | |
| **Country** | United States |
| **Sites/s** | Baylor College of Medicine, Department of Neurology, Vicki Appel MDA, ALS Clinic |
| **Study locale** | Outpatient, multidisciplinary ALS specialty clinic |
| **Disease investigated** | ALS |
| **PARTICPANTS** | |
| **Category of participants under investigation** | Adults with MND and caregivers of adults with MND [all the caregivers were spouses or close family members].  Caregivers completion of the survey appears to be done as proxy to adult diagnosed with MND, but this was not clear in the text. |
| **Eligibility criteria of participants** | Not stated specifically, must have been attendees of the ALS clinic as patient or caregiver. |
| **Sampling strategy** | Consecutive sampling of ALS patients (and their caregivers when present) who attended the ALS clinic. |
| **Number of participants included** | Adults diagnosed with MND: n = 66  Caregivers of those with MND: n= 61 |
| **Characteristics/demographics of participants included** | Characteristic \| Patients (n = 66) \| Caregivers (n = 61); Gender: Men \| 45 (68%) \| 19 (31%); Gender: Women \| 21 (32%) \| 42 (69%); Patient age – Mean ± SD \| 57.9 ± 14.8 \| —; Patient age – Range \| 30–82 years \| —; Disease duration – Mean ± SD \| 2.75 ± 2.61 \| —; Disease duration – Range \| 0.42–8.17 years \| —; AALS total score – Mean ± SD \| 77.0 ± 24.7 \| —; AALS total score – Range \| 34–132 \| —; Bulbar score – Mean ± SD \| 13.1 ± 6.7 \| —; Bulbar score – Range \| 6–30 \| —; Functional vital capacity – Mean ± SD \| 77.5 ± 22.2 \| —; Functional vital capacity – Range \| 25–126 \| — |
| **Response rate** | All approached patients agreed to participate. One caregiver declined. All those that agreed provided full data. |
| **OUTCOME 1: LACK OF FAMILY SUPPORT** | |
| **Instrument or method** | Cross-sectional survey. Patients and caregivers completed a 19-item survey where they were asked to rank their top 3 most important concerns |
| **Preference set used in translation of raw results** | N/A |
| **Reported format** | Responses presented as proportions for each domain (existential, physical, psychosocial concerns) and item. [Have extracted item only – more descriptive].  Data here is the proportion of respondents that included this item as their top 3 priorities. |
| **Result** | Patients (3%)  Caregivers (6.8%) |
| **OUTCOME 2: WORRIES ABOUT THE FUTURE** | |
| **Instrument or method** | As above |
| **Preference set used in translation of raw results** | As above |
| **Reported format** | As above |
| **Result** | Patients (16.7%)  Caregivers (25.4%) |
| **OUTCOME 3: WORRIES ABOUT BEING DEPENDENT ON OTHERS** | |
| **Instrument or method** | As above |
| **Preference set used in translation of raw results** | As above |
| **Reported format** | As above |
| **Result** | Patients (37.9%)  Caregivers (6.8%) |
| **OUTCOME 4: LOSS OF FAITH IN RELIGION** | |
| **Instrument or method** | As above |
| **Preference set used in translation of raw results** | As above |
| **Reported format** | As above |
| **Result** | Patients (1.5%)  Caregivers (0%) |
| **OUTCOME 5: LACK OF FRIENDSHIP OR CHANGES IN FRIENDSHIPS** | |
| **Instrument or method** | As above |
| **Preference set used in translation of raw results** | As above |
| **Reported format** | As above |
| **Result** | The authors did not report on this result at all. |
| **OUTCOME 6: LONELINESS** | |
| **Instrument or method** | As above |
| **Preference set used in translation of raw results** | As above |
| **Reported format** | As above |
| **Result** | Patients (3%)  Caregivers (3%) |
| **OUTCOME 7: FINANCIAL STRESSES** | |
| **Instrument or method** | As above |
| **Preference set used in translation of raw results** | As above |
| **Reported format** | As above |
| **Result** | Patients (16.7%)  Caregivers (13.7%) |
| **OUTCOME 8: EMOTIONAL SWINGS (LAUGHING, CRYING, IRRITABILITY)** | |
| **Instrument or method** | As above |
| **Preference set used in translation of raw results** | As above |
| **Reported format** | As above |
| **Result** | Patients (9%)  Caregivers (17%) |
| **OUTCOME 9: DEPRESSION** | |
| **Instrument or method** | As above |
| **Preference set used in translation of raw results** | As above |
| **Reported format** | As above |
| **Result** | Patients (3%)  Caregivers (5.1%) |
| **OUTCOME 10: PROBLEMS BREATHING** | |
| **Instrument or method** | As above |
| **Preference set used in translation of raw results** | As above |
| **Reported format** | As above |
| **Result** | Patients (13.6%)  Caregivers (25.4%) |
| **OUTCOME 11: PROBLEMS SPEAKING** | |
| **Instrument or method** | As above |
| **Preference set used in translation of raw results** | As above |
| **Reported format** | As above |
| **Result** | Patients (27.3%)  Caregivers (17%) |
| **OUTCOME 12: DIFFICULTY WITH SELF-CARE (I.E. DRESSING, BATHING)** | |
| **Instrument or method** | As above |
| **Preference set used in translation of raw results** | As above |
| **Reported format** | As above |
| **Result** | Patients (18.2%)  Caregivers (22%) |
| **OUTCOME 13: DIFFICULTY WITH PARTICIPATING IN HOBBIES/LEISURE ACTIVITIES** | |
| **Instrument or method** | As above |
| **Preference set used in translation of raw results** | As above |
| **Reported format** | As above |
| **Result** | Patients (10.6%)  Caregivers (5.1%) |
| **OUTCOME 14: DIFFICULTY AT WORK OR INABILITY TO WORK** | |
| **Instrument or method** | As above |
| **Preference set used in translation of raw results** | As above |
| **Reported format** | As above |
| **Result** | Patients (10.6%)  Caregivers (1.7%) |
| **OUTCOME 15: PROBLEMS SWALLOWING/EATING** | |
| **Instrument or method** | As above |
| **Preference set used in translation of raw results** | As above |
| **Reported format** | As above |
| **Result** | Patients (19.7%)  Caregivers (30.5%) |
| **OUTCOME 16: WORRIES ABOUT FAMILY/LOVES ONES’ EMOTIONAL AND PHYSICAL WELL-BEING** | |
| **Instrument or method** | As above |
| **Preference set used in translation of raw results** | As above |
| **Reported format** | As above |
| **Result** | Patients (28.8%)  Caregivers (27.1%) |
| **OUTCOME 17: MUSCLE WEAKNESS** | |
| **Instrument or method** | As above |
| **Preference set used in translation of raw results** | As above |
| **Reported format** | As above |
| **Result** | Patients (25.8%)  Caregivers (22%) |
| **OUTCOME 18: PROGRESSION OF ILLNESS** | |
| **Instrument or method** | As above |
| **Preference set used in translation of raw results** | As above |
| **Reported format** | As above |
| **Result** | Patients (37.9%)  Caregivers (42.4%) |
| **OUTCOME 19: PROBLEMS WITH MOBILITY** | |
| **Instrument or method** | As above |
| **Preference set used in translation of raw results** | As above |
| **Reported format** | As above |
| **Result** | Patients (16.7%)  Caregivers (13.6%) |

**Risk of bias**

| Subdomain and signalling question | Judgement | Notes |
| --- | --- | --- |
| **Selection of participants** | Not serious |  |
| Was an appropriate study sample selected from the study’s sampling frame? | Probably yes | Consecutive sampling of clinic attendees and their caregivers. While eligibility criteria were not specified these were patients of a multi-disciplinary clinic specialising in ALS which is appropriate |
| **Completeness of data** | Not serious |  |
| Was the attrition rate sufficiently low to minimise the risk of bias? | Yes | All but one caregiver approached agreed to participate. There was 100% return of surveys administered |
| **Measurement instrument** | Extremely serious |  |
| Was the instrument used to measure patient values and preferences in a valid and reliable manner? | No | This was an internally developed checklist, that was based on ‘previous reviews’, ‘previous studies’ (an open-ended questionnaire) and the authors clinical experience. |
| Was the instrument used in the intended way? | Probably yes | Likely. The survey was completed by participants (with caregivers assisting those with ALS if required) but the authors acknowledge that some patients took these surveys home and mailed them in. |
| Was a valid representation of the outcome (health state) used? | Probably yes | The outcome descriptors were generic (e.g. problems breathing/ problems speaking used) and non-specific. However, given that the patients and caregivers were asked to consider these items from their unique perspective this is probably acceptable. |
| Did the researchers check for understanding of the instrument? | Investigators did not formally test the understanding, but evidence suggested adequate understanding | No mention of pilot testing or testing for understanding of the questions or instructions of the survey |
| **Data Analysis** | Extremely serious |  |
| Were the results analysed appropriately to avoid influence of bias and confounding? | No | The authors have provided simple descriptive statistics as to the number of respondents that listed an item in their top 3. |
| **Overall risk of bias** | **Extremely serious** |  |

**vanEijk 2022**

**Data extraction**

| Study ID | Van Eijk 2022 |
| --- | --- |
| **DESIGN** | |
| **Other reports of this study identified?** | None identified |
| **Time period study was conducted** | May 2021 |
| **Design** | Cross-sectional survey.  Patients with ALS were asked to score functional domains with an online, self-reported questionnaire (ALSFRS-R) and rank the importance of each domain. The questionnaire was supplemented with two questions; 1) which domain bothers you the most?  2) imagine you will receive a treatment that delays disease progression; delay of which domain is the most important to you? (participants ranked most important to least important) |
| **Outcomes with a valuation provided relevant to the review** | Which domain is most important to you? |
| **SETTING** | |
| **Country** | The Netherlands |
| **Sites/s** | National ALS registry |
| **Study locale** | Community-based, online survey |
| **Disease investigated** | ALS, PMA or PLS |
| **PARTICPANTS** | |
| **Category of participants under investigation** | Adults diagnosed with MND |
| **Eligibility criteria of participants** | Registration at the Netherlands ALS centre on a registry. This requires diagnosis of possible, probable or definite ALS according to the revised El Escorial criteria, or diagnosis with PMA or PLS. |
| **Sampling strategy** | Staff approached all 789 living patients on the registry, who had provided prior consent to be contacted for medical research purposes.  A valid email was only available for 668 of 789 |
| **Number of participants included** | N = 433  ALS n = 271  PMA n = 74  PLS n = 88 |
| **Characteristics/demographics of participants included** | Characteristic \| All patients (N=433) \| ALS (N=271) \| PMA (N=74) \| PLS (N=88); Age at study enrolment, years \| 65 (10) \| 64 (10) \| 67 (9) \| 66 (10); Sex, male \| 295 (68%) \| 186 (69%) \| 57 (77%) \| 52 (60%); Symptom duration, months \| 51 (78) \| 36 (40) \| 79 (78) \| 143 (125); Diagnostic delay, months \| 17 (25) \| 12 (16) \| 26 (47) \| 34 (49); Site of symptom onset, bulbar \| 82 (19%) \| 65 (24%) \| 2 (3%) \| 15 (17%); ALSFRS-R total score \| 32 (10) \| 31 (10) \| 33 (10) \| 35 (8); ΔFRS, points per month \| −0.37 (.38) \| −0.48 (0.41) \| −0.26 (0.28) \| −0.13 (0.19); Riluzole use, yes \| 271 (63%) \| 214 (79%) \| 46 (62%) \| 11 (13%); Gastrostomy, yes \| 67 (16%) \| 58 (21%) \| 5 (7%) \| 4 (5%); Respiratory support, yes \| 87 (20%) \| 62 (23%) \| 18 (24%) \| 7 (8%); Presence of frontotemporal dementia \| 9 (2%) \| 7 (3%) \| 0 (0%) \| 2 (2%) |
| **Response rate** | 668 eligible patients were contacted. 443 patients completed the online survey. |
| **OUTCOME 1: WHICH DOMAIN IS MOST IMPORTANT TO YOU?** | |
| **Instrument or method** | Online questionnaire that asked “imagine you will receive a treatment that delays disease progression; delay of which domain is the most important to you? (participants ranked most important to least important)” |
| **Preference set used in translation of raw results** | Not applicable |
| **Reported format** | % of responses |
| **Result** | The most important domain reported by the patients was respiratory (37.5%), followed by bulbar (30.9%), gross motor (20.4%) and fine motor (11.2%). Interesting to note that there is a weak to moderate association between the domain that is most important to them and the domain that is most affected (Cohen’s Kappa 0.323, p<0.001), or was first affected (Cohen’s Kappa 0.121, p<0.001). Overall, a wide variation was found in the order of importance of domains reported by patients. The below table has been digitised using ChatGPT from a bar chart presented in the original stud (R= Respiratory, B=Bulbar, G=Gross Motor, F=Fine motor). Order of Importance \| Proportion of Patients; All equal \| 0.375; RBFG \| 0.095; RBGF \| 0.060; BRFG \| 0.052; BRGF \| 0.045; BFGR \| 0.037; BFRG \| 0.034; GBFR \| 0.028; RFGB \| 0.026; GFBR \| 0.024; GBRF \| 0.024; GRFB \| 0.021; GFRB \| 0.021; BGFR \| 0.018; GBFR \| 0.018; RGBF \| 0.015; RGFB \| 0.014; FGBR \| 0.014; FGRB \| 0.013; RFBG \| 0.012; FRBG \| 0.012; FBRG \| 0.011; BGRF \| 0.010; FBRG \| 0.009; FBGR \| 0.008 |

**Risk of bias**

| Subdomain and signalling question | Judgement | Notes |
| --- | --- | --- |
| **Selection of participants** | Not serious |  |
| Was an appropriate study sample selected from the study’s sampling frame? | Probably yes | This was an online survey, and participants were contacted from a national registry. There may be some concerns that the respondent completing the survey was not the respondent it was addressed to, but this seems unlikely. |
| **Completeness of data** | Not serious |  |
| Was the attrition rate sufficiently low to minimise the risk of bias? | Yes | Only 20 patients completed but did not finish the survey. Only data from completed surveys (n=433) was used. |
| **Measurement instrument** | Not serious |  |
| Was the instrument used to measure patient values and preferences in a valid and reliable manner? | Probably no | The outcome valuation that took place in this study is derived from the single question “Imagine you will receive a treatment that delays disease progression; delay of which domain is the most important to you?” of which the patients ranked the four domains from most to least important. However, these questions have not been previously validated to measure patient values and preferences, it does however, directly ask the patients for their perspective of domains that were described through the ALSFRS-R |
| Was the instrument used in the intended way? | Yes | Survey that was administered routinely via an online platform |
| Was a valid representation of the outcome (health state) used? | Yes | The domains were based on the four domains of the validated ALSFRS-R. Patients were asked of these domains, which would be most important, from their perspective, for an improvement to be seen. |
| Did the researchers check for understanding of the instrument? | Investigators did not formally test the understanding, but evidence suggested adequate understanding | No explicit mention of cognitive testing or piloting to confirm participant understanding. |
| **Data Analysis** | Not serious |  |
| Were the results analysed appropriately to avoid influence of bias and confounding? | Yes | The authors have assessed the data to explore the impacts of multiple potential covariates and have analysed their data according to what domain was affect, or first onset. |
| **Overall risk of bias** | **Not serious** |  |

**VanEijk 2024**

**Data extraction**

| Study ID | Van Eijk 2024 |
| --- | --- |
| **DESIGN** | |
| **Other reports of this study identified?** | None identified |
| **Time period study was conducted** | June 2022 – November 2023 |
| **Design** | Cross-sectional survey.  Patients with ALS were asked to score functional domains with an online, self-reported questionnaire (ALSFRS-R) and rank the importance of each domain. The questionnaire was supplemented with two questions; 1) which domain bothers you the most?  2) imagine you will receive a treatment that delays disease progression; delay of which domain is the most important to you? (participants ranked most important to least important)  *Note, identical methods to 2022 paper |
| **Outcomes with a valuation provided relevant to the review** | Which domain is most important to you? |
| **SETTING** | |
| **Country** | The Netherlands |
| **Sites/s** | National ALS registry |
| **Study locale** | Community-based, online survey |
| **Disease investigated** | ALS, PMA or PLS |
| **PARTICPANTS** | |
| **Category of participants under investigation** | Adults diagnosed with MND |
| **Eligibility criteria of participants** | Registration at the Netherlands ALS centre on a registry. This requires diagnosis of possible, probable or definite ALS according to the revised El Escorial criteria, or diagnosis with PMA or PLS. |
| **Sampling strategy** | Staff approached all patients that were alive on June 8^th^ 2022. 855 living patients on the registry, who had provided prior consent to be contacted for medical research purposes. |
| **Number of participants included** | Completed survey: n = 433  Completed re-test survey: n = 382 (the data used in this paper) |
| **Characteristics/demographics of participants included** | Patient characteristic \| Reliability cohort (N=382); Age at baseline, y \| 65 (10); Sex, male, n (%) \| 254 (67); Site of symptom onset, bulbar, n (%) \| 57 (15); Type of MND, n (%) - ALS \| 233 (61); Type of MND, n (%) - PMA \| 62 (16); Type of MND, n (%) - PLS \| 87 (23); Symptom duration, mo \| 44 (80); ALSFRS-R total score \| 35 (9); ΔFRS, points per month \| −0.35 (0.35); Riluzole use, yes, n (%) \| 250 (65); Gastrostomy, yes, n (%) \| 38 (10); Respiratory support, yes, n (%) \| 62 (16); Most bothersome domain (current) - Bulbar \| 77 (20); Most bothersome domain (current) - Fine motor \| 117 (31); Most bothersome domain (current) - Gross motor \| 158 (41); Most bothersome domain (current) - Respiratory \| 30 (8) |
| **Response rate** | Of the 663 patients who were approached in June 2022, 423 patients completed the survey (63.8%). After 2 weeks, the retest survey was completed by 382 of the 423 patients (90.3%); |
| **OUTCOME 1: WHICH DOMAIN IS MOST IMPORTANT TO YOU?** | |
| **Instrument or method** | Online questionnaire that asked “imagine you will receive a treatment that delays disease progression; delay of which domain is the most important to you? (participants ranked most important to least important)” |
| **Preference set used in translation of raw results** | Not applicable |
| **Reported format** | n (%) |
| **Result** | Most important domain - No preference (all equal): n = 141 (37%)  Most important domain – Bulbar: n = 85 (22%)  Most important domain - Fine motor: n = 32 (8%)  Most important domain - Gross motor: n = 38 (10%)  Most important domain- Respiratory: n = 86 (23%) |

**Risk of bias**

| Subdomain and signalling question | Judgement | Notes |
| --- | --- | --- |
| **Selection of participants** | Not serious |  |
| Was an appropriate study sample selected from the study’s sampling frame? | Probably yes | This was an online survey, and participants were contacted from a national registry. There may be some concerns that the respondent completing the survey was not the respondent it was addressed to, but this seems unlikely. |
| **Completeness of data** | Not serious |  |
| Was the attrition rate sufficiently low to minimise the risk of bias? | Probably yes | The flow of data is confusing in this study. 433 participants completed the survey, and then a second retest was administered 2-weeks later, of which only 382 participants completed. The data from the 382 participants was utilised in this review for direct outcome valuation. This attrition seems high, but is likely OK, given only the second data was utilised. |
| **Measurement instrument** | Not serious |  |
| Was the instrument used to measure patient values and preferences in a valid and reliable manner? | Probably yes | The outcome valuation that took place in this study is derived from the single question “Imagine you will receive a treatment that delays disease progression; delay of which domain is the most important to you?” of which the patients ranked the four domains from most to least important. While this question has not been previously validated to measure patient values and preferences, it does directly ask the patients for their perspective of domains that were described through the ALSFRS-R. |
| Was the instrument used in the intended way? | Yes | Survey that was administered routinely via an online platform. |
| Was a valid representation of the outcome (health state) used? | Yes | The domains were based on the four domains of the validated ALSFRS-R. Patients were asked of these domains, which would be most important, from their perspective, for an improvement to be seen. A brief introduction was provided to outline which symptoms were associated with each domain. |
| Did the researchers check for understanding of the instrument? | Investigators tested the understanding, and understanding was adequate | Only the data from the re-rest was used. Meaning participants had already been exposed to these questions as a ‘pilot’ of sorts. |
| **Data Analysis** | Not serious |  |
| Were the results analysed appropriately to avoid influence of bias and confounding? | Yes | The authors have assessed the data to explore the impacts of multiple potential covariates and have analysed their data according to what domain was affect, or first onset. |
| **Overall risk of bias** | **Not serious** |  |

**Wagner 2018**

**Data extraction**

| Study ID | Wagner 2018 |
| --- | --- |
| **DESIGN** | |
| **Other reports of this study identified?** | None identified |
| **Time period study was conducted** | Not specified |
| **Design** | Cross-sectional survey. 43 questions  Anonymous online survey, the following questions in this survey provide direct valuation: |
| **Outcomes with a valuation provided relevant to the review** | If I could “do it all over again” I would choose to have genetic testing  I would recommend that other persons with ALS consider genetic testing |
| **SETTING** | |
| **Country** | United States |
| **Sites/s** | Adults diagnosed with ALS that were enrolled in the CDC ALS registry. |
| **Study locale** | Online survey |
| **Disease investigated** | ALS |
| **PARTICPANTS** | |
| **Category of participants under investigation** | Adults diagnosed with ALS |
| **Eligibility criteria of participants** | Confirmed ALS diagnosis had enrolled on the CDC ALS registry and had provided consent to be contact for medical research purposes.  In some cases, caregivers responded on behalf of patients. But these results have not been separated in the data. |
| **Sampling strategy** | Convenience sampling. Eligible patients were contacted via email with a link to complete the survey |
| **Number of participants included** | N = 449 |
| **Characteristics/demographics of participants included** | Baseline characteristics of survey respondents has not been reported. The following data comes from Wagner 2016 which used the same sample.  White/Caucasian ethnicity (95.3 %; *n* = 428/449)  age over 50 years (88.4 %; *n* = 397).  Onset of ALS symptoms was indicated as limb in 77.7 % (*n* = 349) and bulbar in 22.2 % (*n* = 100).  The majority of respondents (82.4 %; *n* = 370) indicated age at diagnosis as over age 50 years. |
| **Response rate** | N/A only those that completed the survey have been in included in the data analysis |
| **OUTCOME 1: IF I COULD “DO IT ALL OVER AGAIN” I WOULD CHOOSE TO HAVE GENETIC TESTING** | |
| **Instrument or method** | Online survey, responses to the question were “agree”, “neutral” or “disagree” |
| **Preference set used in translation of raw results** | Not Applicable |
| **Reported format** | % |
| **Result** | Agree = 80.1%  Neutral = 5.3%  Disagree = 4.2% |
| **OUTCOME 2: I WOULD RECOMMEND THAT OTHER PERSONS WITH ALS CONSIDER GENETIC TESTING** | |
| **Instrument or method** | Online survey, responses to the question were “agree”, “neutral” or “disagree” |
| **Preference set used in translation of raw results** | Not Applicable |
| **Reported format** | % |
| **Result** | Agree = 83.3%  Neutral = 12.5%  Disagree = 1.1% |

**Risk of bias**

| Subdomain and signalling question | Judgement | Notes |
| --- | --- | --- |
| **Selection of participants** | Not serious |  |
| Was an appropriate study sample selected from the study’s sampling frame? | Probably yes | Online survey was sent to adults diagnosed with ALS that were registered on the CDC ALS register and had consented to being contacted for medical research purposes. The authors do acknowledge that in some instances carers completed the assessment on behalf of the person with ALS; however, this distinction is not reflected in how the data has been presented or analysed (discussed below). |
| **Completeness of data** | Not serious |  |
| Was the attrition rate sufficiently low to minimise the risk of bias? | Yes | An online survey was sent to all eligible participants. Only completed survey responses were utilised in this study. |
| **Measurement instrument** | Extremely serious |  |
| Was the instrument used to measure patient values and preferences in a valid and reliable manner? | No | The questions as posed in this survey are not an appropriate measure to establish patient values and preferences. It should be noted that they were not designed to be, but they still provide indirect valuation. |
| Was the instrument used in the intended way? | Yes | An online survey, administered through survey monkey. All respondents were provided with the same survey experience. |
| Was a valid representation of the outcome (health state) used? | Yes | The questions explicitly ask the patient to refer to their own subjective experience, and whether or not they would make the same decision should they have their time over again. |
| Did the researchers check for understanding of the instrument? | Investigators did not formally test the understanding, but evidence suggested adequate understanding | No cognitive screening or instrument comprehension reported. Reliant on patient recall. |
| **Data Analysis** | Extremely serious |  |
| Were the results analysed appropriately to avoid influence of bias and confounding? | No | Data has only been presented descriptively. The authors also acknowledge that in some instances carers completed the assessment on behalf of the person with ALS; however, this distinction is not reflected in how the data has been presented or analysed. |
| **Overall risk of bias** | **Extremely serious** |  |

**Wei 2021**

**Data extraction**

| Study ID | Wei 2021 |
| --- | --- |
| **DESIGN** | |
| **Other reports of this study identified?** | None identified |
| **Time period study was conducted** | May 2018 – May 2020 |
| **Design** | Cross-sectional survey where patients self-assessed HRQoL using the EQ-5D-5L and EQ-VAS through face-to-face interviews with assistance of a trained neurologist, or self-administered by literature patients accompanied by a supervisor |
| **Outcomes with a valuation provided relevant to the review** | Health-related quality of life (EQ-5D-5L)  Mobility (EQ-5D-5L)  Self-Care  Usual Activities  Pain/Discomfort  Anxiety/Depression |
| **SETTING** | |
| **Country** | China |
| **Sites/s** | West China Hospital, Sichuan University |
| **Study locale** | Outpatient clinic with community follow-up |
| **Disease investigated** | ALS |
| **PARTICPANTS** | |
| **Category of participants under investigation** | Adults diagnosed with MND |
| **Eligibility criteria of participants** | Patients who were diagnosed with definite, probable or possible ALS according to the El Escorial revised criteria.  Patients with a diagnosis of progressive muscular atrophy, progressive bulbar paralysis, primary lateral sclerosis, and juvenile ALS were excluded. |
| **Sampling strategy** | Consecutive sampling - From May 2018 to May 2020, patients who were diagnosed with definite, probable, or possible ALS (according to the El Escorial revised criteria) were registered in the study. |
| **Number of participants included** | 547 |
| **Characteristics/demographics of participants included** | Characteristic \| Total (n=547) \| Stage 1 (n=224) \| Stage 2 (n=193) \| Stage 3 (n=86) \| Stage 4 (n=44); Age, years (M (SD)) \| 54.7 (11.4) \| 54.0 (11.0) \| 55.1 (11.8) \| 55.1 (12.7) \| 55.5 (9.5); Male n (%) \| 347 (63.4) \| 153 (68.3) \| 114 (59.1) \| 52 (60.5) \| 28 (63.6); Education, years (M (SD)) \| 9.4 (3.6) \| 9.7 (3.8) \| 9.1 (3.4) \| 8.8 (3.4) \| 9.5 (3.7); Age at onset, years (M (SD)) \| 53.4 (11.4) \| 52.8 (11.1) \| 53.8 (11.7) \| 53.9 (12.6) \| 53.8 (9.5); Disease duration, months (M (SD)) \| 15.5 (14.4) \| 15.1 (14.3) \| 15.2 (14.3) \| 14.5 (13.2) \| 20.7 (16.5); Diagnostic delay, months (M (SD)) \| 14.6 (14.0) \| 14.3 (13.6) \| 14.3 (14.1) \| 13.6 (13.1) \| 20.1 (16.6); Onset region (B, UL, LL) \| 68, 273, 206 \| 30, 109, 85 \| 22, 96, 75 \| 10, 47, 29 \| 6, 21, 17; ALSFRS-R score, (M (SD)) \| 41.4 (4.4) \| 44.4 (2.4) \| 40.8 (3.4) \| 37.6 (4.2) \| 36.4 (5.1); BMI, M (SD) \| 22.6 (3.1) \| 22.8 (3.1) \| 22.5 (3.2) \| 22.5 (3.1) \| 22.5 (3.2) |
| **Response rate** | Not provided |
| **OUTCOME 1: HEALTH-RELATED QUALITY OF LIFE** | |
| **Instrument or method** | EQ-5D-5L is a quantitative tool to evaluate health status by measuring perception of questions related to five dimensions of health: Mobility (MO), Self-care (SC), Usual activities (UA), Pain/discomfort (PD), and Anxiety/ depression (AD). Each dimension has five levels: no problems, slight problems, moderate problems, severe problems, and extreme problems. The five EQ- 5D-5L descriptive scores may be converted into a single aggregated “health utility” score anchored at 1 (perfect health) and 0 (death). |
| **Preference set used in translation of raw results** | Chinese value set |
| **Reported format** | The authors have stated: was convenient to dichotomize the EQ-5D-5L levels into ’no problems’ (i.e. level 1) and ’problems’ (i.e. levels 2 to 5), which changed the profiles into frequencies of reported problems. |
| **Result** | The median health utility score was 0.78 and the median EQ-VAS score was 70 for all 503 ALS patients. **Group** \| **Subgroup** \| **n** \| **Health Utility Score Median (IQR)**; Age of onset \| > 45 \| 390 \| 0.74 (0.57, 0.88); \| < 45 \| 113 \| 0.80 (0.63, 0.91); Sex \| Male \| 319 \| 0.78 (0.58, 0.91); \| Female \| 184 \| 0.73 (0.57, 0.87); Onset region \| Bulbar onset \| 62 \| 0.90 (0.80, 1.00); \| Spinal onset \| 441 \| 0.73 (0.56, 0.86); ALSFRS-R score \| ≥ 40 \| 378 \| 0.80 (0.67, 0.91); \| < 40 \| 125 \| 0.53 (0.31, 0.68); Stages \| 1 \| 224 \| 0.85 (0.69, 0.93); \| 2 \| 193 \| 0.69 (0.51, 0.84); \| 3 \| 86 \| 0.66 (0.44, 0.79); FAB \| ≥ 16 \| 335 \| 0.78 (0.59, 0.91); \| < 16 \| 168 \| 0.73 (0.56, 0.86); ACE-R \| ≥ 75 \| 332 \| 0.78 (0.59, 0.89); \| < 75 \| 171 \| 0.73 (0.54, 0.88); Depression \| > 7 \| 238 \| 0.65 (0.46, 0.78); \| ≤ 7 \| 265 \| 0.85 (0.73, 0.93); Anxiety \| > 7 \| 150 \| 0.63 (0.44, 0.74); \| ≤ 7 \| 353 \| 0.80 (0.65, 0.91); EDS \| ≥ 10 \| 78 \| 0.78 (0.58, 0.86); \| < 10 \| 425 \| 0.77 (0.57, 0.89); RBD \| ≥ 5 \| 31 \| 0.69 (0.54, 0.84); \| < 5 \| 472 \| 0.78 (0.58, 0.89); PSQI \| ≥ 5 \| 183 \| 0.71 (0.55, 0.85); \| ≤ 5 \| 320 \| 0.78 (0.60, 0.91) |
| **OUTCOME 2: MOBILITY** | |
| **Instrument or method** | As above |
| **Preference set used in translation of raw results** | As above |
| **Reported format** | The authors have stated: was convenient to dichotomize the EQ-5D-5L levels into ’no problems’ (i.e. level 1) and ’problems’ (i.e. levels 2 to 5), which changed the profiles into frequencies of reported problems. |
| **Result** | Level 1 = 4.9  Level 2 = 10.5  Level 3 = 16.3  Level 4 = 28.3  Level 5 = 39.9 |
| **OUTCOME 3: SELF-CARE** | |
| **Instrument or method** | As above |
| **Preference set used in translation of raw results** | As above |
| **Reported format** | The authors have been convenient to dichotomize the EQ-5D-5L levels into ’no problems’ (i.e. level 1) and ’problems’ (i.e. levels 2 to 5), which changed the profiles into frequencies of reported problems. |
| **Result** | Level 1 = 9.9  Level 2 = 12.3  Level 3 = 15.2  Level 4 = 39.9  Level 5 = 22.7 |
| **OUTCOME 4: USUAL ACTIVITY** | |
| **Instrument or method** | As above |
| **Preference set used in translation of raw results** | As above |
| **Reported format** | The authors have stated: was convenient to dichotomize the EQ-5D-5L levels into ’no problems’ (i.e. level 1) and ’problems’ (i.e. levels 2 to 5), which changed the profiles into frequencies of reported problems. |
| **Result** | Level 1 = 5.2  Level 2 = 15.4  Level 3 = 21.5  Level 4 = 40.2  Level 5 = 17.6 |
| **OUTCOME 5: PAIN/DISCOMFORT** | |
| **Instrument or method** | As above |
| **Preference set used in translation of raw results** | As above |
| **Reported format** | The authors have stated: was convenient to dichotomize the EQ-5D-5L levels into ’no problems’ (i.e. level 1) and ’problems’ (i.e. levels 2 to 5), which changed the profiles into frequencies of reported problems. |
| **Result** | Level 1 = 0  Level 2 = 3.1  Level 3 = 6.8  Level 4 = 31.6  Level 5 = 58.5 |
| **OUTCOME 6: ANXIETY/DEPRESSION** | |
| **Instrument or method** | As above |
| **Preference set used in translation of raw results** | As above |
| **Reported format** | The authors have stated: was convenient to dichotomize the EQ-5D-5L levels into ’no problems’ (i.e. level 1) and ’problems’ (i.e. levels 2 to 5), which changed the profiles into frequencies of reported problems. |
| **Result** | Level 1 = 0  Level 2 = 2.7  Level 3 = 14.6  Level 4 = 34.7  Level 5 = 47.7 |

**Risk of bias**

| Subdomain and signalling question | Judgement | Notes |
| --- | --- | --- |
| **Selection of participants** | Not serious |  |
| Was an appropriate study sample selected from the study’s sampling frame? | Yes | Participants were patients at an outpatient clinic who had diagnosed ALS. |
| **Completeness of data** | Serious |  |
| Was the attrition rate sufficiently low to minimise the risk of bias? | Probably no | The authors have not provided any detail regarding the number of participants eligible or the number of participants that did not complete the survey. As such, a score of probably no has been provided as it is conservative without penalising the study too harshly. |
| **Measurement instrument** | Serious |  |
| Was the instrument used to measure patient values and preferences in a valid and reliable manner? | Yes | The EQ-5D-5L is a validated and standardised tool to assess patient values and preferences. |
| Was the instrument used in the intended way? | Probably no | This has been scored as a probably no, as the authors acknowledge that some participants provided data via self-report accompanied by a supervisor, while others participated in an interview with a trained neurologist. This point of difference has not been discussed further, and has not been considered in the presentation of the results. |
| Was a valid representation of the outcome (health state) used? | Yes | The health-states as utilised in the Eq-5D-5L are appropriate and objectively described. |
| Did the researchers check for understanding of the instrument? | Investigators did not formally test the understanding, but evidence suggested adequate understanding | No pilot testing or cognitive testing reported. |
| **Data Analysis** | Not serious |  |
| Were the results analysed appropriately to avoid influence of bias and confounding? | Yes | Multiple potential covariates were considered in the analysis and stratification of the results which have been reported transparently and in full. |
| **Overall risk of bias** | **Serious** |  |

**Supplementary material 5 - Additional methods**

Study selection and screening

Prior to importing records into Covidence, all identified records were initially compiled and managed within EndNote™. Where relevant systematic reviews were identified in the search, we reviewed their reference lists of included and excluded studies for consideration; however, the systematic reviews themselves were not included. Where members of the author team were named as authors on primary studies identified through the search, they were to be excluded from any decision-making regarding the inclusion of the study or the assessment of the study's risk of bias and methodological limitations. Ultimately, this scenario did not occur.

Assessment of risk of bias

The application of the ROBVALU tool to assess risk of bias in studies evaluating the value people place on outcomes follows seven steps:

1. Specify the review question
2. Specify the outcome being assessed
3. Identify the sampling frame, the measurement instrument used, and the data analysis plan
4. Answer the signalling questions of the four subdomains
5. Make a judgment if the four subdomains have important risk of bias concerns
6. Formulate a judgement regarding bias for the four subdomains
7. Formulate an overall risk of bias judgment for the study outcome being assessed

Data synthesis

As per the methods of Saeed 2020,^28^ relative heterogeneity across studies was assessed using the I^2^ statistic. Heterogeneity was interpreted using I2 thresholds (0-40%: low; 30-60%: moderate; 50-90%: substantial; 75-100%: considerable) in accordance with the Cochrane Handbook.^30^ In addition to I^2^, Cochran’s Q test was used to statistically evaluate whether the observed variability in effect estimates was greater than expected by chance. Significant Q value at the 5% level was interpreted as evidence of heterogeneity. For random-effects meta-analysis, we also reported tau-squared to indicate between study variance. Similarly absolute heterogeneity was investigated by visual inspection of forest plot and estimating prediction interval for effect estimates. A global Wald-type test was used to assess the statistical significance of the pooled interactions, providing a single overall test for subgroup effect.

Where health states were reported across multiple studies, however different instruments have been used to preclude meta-analysis, then the ranges of each utility score from these studies was reported.

Where a single study has provided a utility score for a health state, an appropriate reported measure of central tendency and associated measure of variability were extracted. A narrative description for each health outcome was reported as per the methods of Etxeandia-Ikobaltzeta 2020.

Non-utility quantitative outcome data (e.g. data from direct choice studies or other quantitative studies on outcome importance) were narratively reported according to pre-determined population subgroups (Patients, Caregivers, Clinicians or Carriers).

We contacted study authors to obtain missing data and received data from one study (detailed below). We did not impute any missing data from available data.

**Supplementary material 6 - Additional result data**

Characteristics of the included studies

#### Study designs and time periods

Thirteen included studies employed a cross-sectional design with direct outcome valuations reported.^3,4,6,9,11,13,14,17,21-25^ Four studies conducted direct utility and health state value studies through analysis of retrospective RCT or cohort study data.^1,5,10,12^ Three studies conducted a pre-test post-test study design.^2,8,18^ Two studies each employed discrete choice experiment methodology,^16,20^ and standard gamble methodology.^6,12^ Thakore 2019^19^ conducted health economic modelling to examine the importance of outcomes within a dataset to inform cost-effectiveness of ALS care. For the studies that collected data prospectively, most employed purposive sampling to recruit participants, while consecutive or convenience sampling methods were the next most common.

The included studies were conducted between 1996 (Rudnicki 1997^14^) – 2023 (VanEjik 2024^22^). Ten studies^3,4,9,16-18,21-23,25^ were conducted over the last ten years (2015 or later) and seven studies^4,16,17,21-23^ were conducted over the last five years (2020 or later). In five studies,^2,5,8,11,12^ the study period was not clearly specified. Regarding study duration, the longest was Raheja 2016^13^ (2006-2012), followed by Thakore 2019^19^ (2010-2015), Wei 2021^25^ (2018-2020), Jones 2014^10^ (2009-2011) and Seo 2024^16^ (2020-2022). The remaining studies were conducted over a two-year period or less. The characteristics of the included studies have been presented in full, and summarised as Supplementary Material 4.

#### Participants and settings

In total, 10,397 individuals participated in the studies included in this review, comprising 6,246 adults with MND (inclusive of MND subtypes), 399 caregivers, and 152 clinicians and/or healthcare providers. The sample sizes of the included studies ranged from a minimum of 10 to a maximum of 880 participants. There were 17 studies that recruited adults with MND (inclusive of MND subtypes).^3-6,8-10,12-14,16,18,19,22-25^ Three studies included both adults with MND patients and their caregivers,^2,20,21^ two studies involved only caregivers,^11,15^ one study involved both adults with MND and their clinicians who provided proxy measures of outcome valuation on behalf of the patients,^17^ and one study that only included clinicians who provided proxy measures.^1^ The mean age of the adults with MND ranged from 52.40^11^ to 65.07 years,^12^ while the age of caregivers ranging from 51^20^ to 58^2^ years.

Eight studies recruited participants from multiple countries^1,4,5,9,11,13,16,17^ including European countries, the USA and Canada. Among these, two studies included participants from Australia.^5,9^ Conroy 2023^4^ recruited from nine European countries (Italy, France, the Netherlands, Spain, Ireland, UK, Belgium, Germany and Sweden). Five studies were conducted in the USA,^2,14,19,21,24^ three in the UK,^6,10,12^ two in the Netherlands^22,23^ and two in Sweden.^3,15^ The remaining studies were from Germany,^8^ Italy,^18^ Ireland^20^ and China.^25^

Ten of the included studies specified eligibility criteria requiring a definitive, probable or possible diagnosis of ALS according to *the El Escorial revised criteria*.^2,10,11,13-15,20,22,23,25^ Maksymowicz-Sliwinska 2023^11^ specified that caregivers had to be taking care of patients diagnosed with clinically possible, probable or definite ALS according to *the El Escorial criteria*, with a disease duration of more than 3 months. Adelman 2004^2^ specified that, in addition to the above criteria, participants were required to have a forced vital capacity (FVC) of 50% predicted value, and be tracheostomy-free at the time of recruitment. Raheja 2016^13^ recruited ALS patients with vital capacity of more than 60% of predicted, and symptom duration of less than three years. Seo 2024^16^ also specified that eligible patients were those diagnosed with ALS, aged 18 years or older, not in hospice care and did not have a tracheostomy. Ackerman 1999^1^ provided health state descriptions of living with ALS, derived from the 5 clinical stages. These were then mapped to the Appel ALS rating scale to organise ALS into 5 distinct ‘levels’.

Conroy 2023^4^ recruited patients with ALS through an anonymous survey without any exclusion criteria. Gebrehiwet 2023^5^ selected adults diagnosed with ALS who met the FORTITUDE-ALS trial criteria and received at least one dose of the study drug (from previous RCT) and had ALSFRS-R assessment at baseline and one post-baseline visit. Tarlarini 2017^18^ recruited patients with a clinical diagnosis of ALS and dysphagia with an ALSFRS-R swallowing score of <4. Van Eijk 2022^23^ included patients with a diagnosis of PMA and PLS, whereas Wei 2021^25^ excluded patients with PMA, PLS, and juvenile ALS.

The majority of the studies recruited participants from outpatient settings,^2,3,5,6,8,10,13,14,19-21,25^ such as MND clinics, ALS centre,^2^ neurology departments and multidisciplinary MND clinic^20^ or MND speciality clinic,^21^ while others were community based studies.^9,11,12,16,17,22,23^ Tarlarini 2017 included patients from both inpatient and outpatient units.^18^ Few studies recruited participants through online surveys^4,24^ or a home visit.^15^

Few studies specified exclusion of patients, those that did provided reasons such as the inability to communicate,^8^ cognitive impairment,^3,10^ dementia^11,13^ and serious comorbidities.^6^

**Assessment of risk of bias**

#### Overall risk of bias

Twelve of the included studies^6,9,10,12,13,15-17,19,20,22,23^ had no serious overall risk of bias. In contrast, eleven studies^2-5,8,11,14,18,21,24,25^were found to have extremely serious overall risk of bias. A detailed breakdown of the risk of bias across each domain in each study is presented below.

#### Selection of participants

The vast majority^2,4-6,8^ ^9,10,12-25^ of the included studies had no serious risk of bias arising from selection of participants. Ackerman 1999,^1^ Akerblom 2021^3^ and Maksymowicz-Sliwinska 2023^11^ had serious risk of bias in this domain. Ackerman 1999^1^ provided suitable eligibility criteria for their participants, but did not provide any methods for how the participants were selected into the study. Akerblom 2021^3^ used purposive sampling to recruit participants without specifying a sampling frame, which may have introduced selection bias. Patients with a confirmed MND diagnosis and had scheduled for a visit to any of the MND teams during recruitment period were invited to participate. Maksymowicz-Sliwinska 2023,^11^ did not provide sufficient details about their sampling methods and an appropriate sample was not selected from a defined sampling frame.

#### Completeness of data

Twenty studies^1-3,6,8-17,19-24^ had no serious risk of bias arising from completeness of data. Conroy 2023^4^ and Gebrehiwet 2023^5^ had extremely serious risk of bias arising from issues with data completeness. In Conroy 2023^4^, 506 participants out of 857 participants that were approached, completed the survey, which may have introduced bias from data completeness. Particularly as the authors have not reported totals next to the reporting of some outcome data. Similarly, Gebrehiwet 2023^5^ had missing data for key outcomes of the study (ALSFRS-R score and EQ-5D). Tarlarini 2017^18^ had serious risk of bias in this domain, with inconsistencies in the number of participants who provided data. Similarly, Wei 2021^25^ had serious risk of bias arising from data completeness; the authors did not report the number eligible participants or those who did not complete the study survey.

#### Measurement instrument

Fifteen studies^1,5,6,9-13,15-17,19,20,22,23^ had no serious risk of bias arising from measurement instrument.

Four studies (Akerblom 2021,^3^ Conroy 2023,^4^ Trail 2004^21^ and Wagner 2018^24^) had extremely serious risk of bias arising from measurement instrument. Akerblom 2021^3^ used a validated tool (Schedule for the Evaluation of Individual Quality of Life-Direct Weighting) however the weighting procedure of the standard instrument was omitted. Participants were asked to list what is most important to them from a list of outcomes, however there was no evidence that these outcomes were appropriately defined to the participants. Additionally, the authors did not test participant’s understanding of the instruments used. Conroy 2023^4^ conducted a simple online survey which was not specifically designed to directly measure patient values and preferences of outcomes, and there was also no evidence of testing for understanding of the instrument. Trail 2004^21^ used an instrument developed by the authors and there was no evidence of checking for understanding of the instrument among participants. Wagner 2018^24^ conducted a survey which was not specifically designed to measure patient values and preference of outcomes, additionally the authors also did not check for understanding of the instrument.

Three studies (Adelman 2004,^2^ Rudnicki 1997^14^ and Tarlarini 2017^18^) had a very serious risk of bias. Adelman 2004^2^ used an indirect measure of health states and did not providing specific descriptions to describe the health state for their participants. In Rudnicki 1997^14^ the authors used an instrument which was not validated, and they did not check participants’ understanding of this instrument. Tarlarini 2017^18^ did not check participant understanding of the instrument they employed to assess patient values and preferences.

Two studies (Hecht 2002,^8^ and Wei 2021^25^) were found to have serious risk of bias in this domain. Hecht 2002^8^ used an interview-based questionnaire to elicit outcome valuations; however, the tool was neither standardised nor validated. In Wei 2021^25^ the instrument was not used as intended. Additionally, the authors did not formally check participants’ understanding of the instrument.

#### Data analysis

Fourteen studies^1,6,9,10,12,13,15-17,19,20,22,23,25^ had no serious risk of bias arising from data analysis. The remaining ten studies (Adelman 2004,^2^ Akerblom 2021,^3^ Conroy 2023,^4^ Gebrehiwet 2023,^5^ Hecht 2002,^8^ Maksymowicz-Sliwinska 2023,^11^ Rudnicki 1997,^14^ Tarlarini 2017,^18^ Trail 2004^21^ and Wagner 2018^24^) had extremely serious risk of bias in this domain. Specifically, Adelman 2004^2^ and Gebrehiwet 2023,^5^ reported means and standard deviations without making any adjustments for potential or actual confounders. Maksymowicz-Sliwinska 2023,^11^ Conroy 2023^4^ and Akerblom 2021^3^ reported only counts and percentages without accounting for potential bias or confounding effect. Similarly, Hecht 2002,^8^ Rudnicki 1997,^14^ Tarlarini 2017,^18^ Trail 2004^21^ and Wagner 2018^24^ conducted descriptive analysis without adjusting for confounding variables.

Forest-plots

#### Overall health-related quality for adults diagnosed with ALS

**Supplementary figure 1 -** Forest-plot detailing the meta-analysis (inverse variance, fixed-effect model) of mean EQ5D health state valuation for adults diagnosed with ALS at any stage of disease progression

#### Health-related quality for adults diagnosed with ALS at different stage of disease progression (ALS Health State Scale)

**Supplementary figure 2 -** Forest-plot detailing the mean EQ5D health state valuation for adults diagnosed with ALS sub-grouped according to disease progression using the ALS Health State Scale.

#### Health-related quality for adults diagnosed with ALS at different stage of disease progression (Functional Test 9)

**Supplementary figure 3** - Forest-plot detailing the mean EQ5D health state valuation for adults diagnosed with ALS sub-grouped according to disease progression using the Functional Test 9 staging system.

**Supplementary material 7 - Detailed narrative syntheses of each outcome**

**Living with MND**

**Living with ALS (Appel Score = 40 to 59)**

One study conducted a standard gamble exercise to elicit preferences to specific health states. Clinicians provided these valuations as proxy to adults with ALS. Health state descriptions were derived from the 5 clinical stages that were mapped to the Appel ALS rating scale, a disease-specific classification system. This study presented these health states with geometric states (e.g. health state ■) to avoid imposing any hierarchical order among the available health states. Each health state corresponded with an Appel ALS score. This health state (Appel score from 40 to 59) had the following health state description: normal to slightly slurred speech; general diet but need to eliminate hard foods that are difficult to swallow; normal breathing; dress and feed independently and can live independently; may require mobility aids such as a cane; able to work full time; medical procedures are strictly diagnostic. The mean valuation of this health state was 0.89 ± 0.04.

**Living with ALS (Appel Score = 60 to 86)**

One study conducted a standard gamble exercise to elicit preferences to specific health states. Clinicians provided these valuations as proxy to adults with ALS. Health state descriptions were derived from the 5 clinical stages that were mapped to the Appel ALS rating scale, a disease-specific classification system. This study presented these health states with geometric states (e.g. health state ■) to avoid imposing any hierarchical order among the available health states. Each health state corresponded with an Appel ALS score. This health state (Appel score from 60 to 86) had the following health state description: speech is intelligible at least three-quarters of the time; diet limited to finely chopped or ground foods and thick liquids; breathing is moderately impaired, experience shortness of breath on exertion, but can cough effectively; require minimal care for dressing and feeding; require mobility aids such as crutches or occasionally a wheelchair; work limited to part time; medical procedures include chest physiotherapy and diagnostic spirometry. The mean valuation of this health state was 0.82 ± 0.04.

**Living with ALS (Appel Score = 87 to 109)**

One study conducted a standard gamble exercise to elicit preferences to specific health states. Clinicians provided these valuations as proxy to adults with ALS. Health state descriptions were derived from the 5 clinical stages that were mapped to the Appel ALS rating scale, a disease-specific classification system. This study presented these health states with geometric states (e.g. health state ■) to avoid imposing any hierarchical order among the available health states. Each health state corresponded with an Appel ALS score. This health state (Appel score from 87 to 109) had the following health state description: speech is intelligible less than half of the time; diet limited to food of pudding consistency; breathing impaired to point at increased risk for infections; require caretaker to assist with transfers and to assist with dressing and feeding; require use of a wheelchair much of the time; most likely have stopped working; medical care includes PEG consultations, medications for pain, and many unexpected hospitalisations. The mean valuation of this health state was 0.41 ± 0.17.

**Living with ALS (Appel Score = 110 to 128)**

One study conducted a standard gamble exercise to elicit preferences to specific health states. Clinicians provided these valuations as proxy to adults with ALS. Health state descriptions were derived from the 5 clinical stages that were mapped to the Appel ALS rating scale, a disease-specific classification system. This study presented these health states with geometric states (e.g. health state ■) to avoid imposing any hierarchical order among the available health states. Each health state corresponded with an Appel ALS score. This health state (Appel score from 110 to 128) had the following health state description: speech is intelligible less than one-quarter of the time or requires a device to communicate; diet is limited to pureed, blended, thick liquids and likely to need tube feedings; respiratory function is impaired to the extent that pressure support (CPAP, BIPAP) and home oxygen may be needed; fully dependent on a caregiver for dressing and feeding; wheelchair-bound; not working; medical care includes consultations regarding need for tracheostomy/gastrostomy, as well as medications for pain. The mean valuation of this health state was 0.01 ± 0.20.

**Living with ALS (Appel Score = 129 to 164)**

One study conducted a standard gamble exercise to elicit preferences to specific health states. Clinicians provided these valuations as proxy to adults with ALS. Health state descriptions were derived from the 5 clinical stages that were mapped to the Appel ALS rating scale, a disease-specific classification system. This study presented these health states with geometric states (e.g. health state ■) to avoid imposing any hierarchical order among the available health states. Each health state corresponded with an Appel ALS score. This health state (Appel score from 129 to 164) had the following health state description: unable to speak; have had a gastrostomy; respiratory function severely impaired; have had a tracheostomy; fully dependent on a caregiver for dressing and feeding; quadriplegic; not working; most likely in a long-term care facility; medical care includes medications for pain. The mean valuation of this health state was -0.53 ± 0.14.

**Day to day living**

There were two studies that provided outcome data related to day to day living. Helleman 2022^9^ have reported the outcome of ‘daily activity’ had a normalised ranking score of 0.21 (all countries), 0.21 (Netherlands), 0.24 (United Kingdom) and 0.21 (Australia). In the structured interview of Hecht 2002^8^ (methods above) ‘impairment of activities of daily living’ was identified as the worst aspect of the disease by 9.8% of adults with ALS at baseline, and by 3.2% of adults with ALS at a 6-month follow-up interview.

**Physical functioning and mobility**

There were three studies that reported outcome data that we have organised under the broad category of physical functioning and mobility. In the study of Hecht 2002^8^ adults diagnosed with ALS were asked as part of a standardised interview “What are the worst aspects of the disease in your personal experience”. Reduction of mobility was identified and listed as the worst aspect by 19.5% of participants at baseline, and by 29% of participants at a 6-month follow-up interview. In a cross-sectional survey Conroy 2023^4^ asked adults diagnosed with ALS to identify what they wanted their current ALS treatments to impact. There were 49 participants in this study (no total was provided) who stated mobility in response to this question. The final study of Trail 2004^21^ presented a cross-sectional survey, where both adults diagnosed with ALS and caregivers of adults diagnosed with ALS were asked to rank their top three most important concerns. Problems with mobility were listed in the top 3 most important concerns by 16.7% of people diagnosed with ALS and 13.6% of caregivers of people with ALS.^24^

**Genetic Testing**

Only a single study reported outcome valuation data related to genetic testing.^24^ Wagner 2018^24^ conducted an anonymous online survey of adults diagnosed with ALS who had been enrolled on the United States CDC (Centers for Disease Control) ALS registry who had previously reported as having undertaken genetic testing regarding their condition. Adults with ALS were asked “If I “could do it all over again” I would choose to have genetic testing”, to which 80.1% agreed, 5.3% responded neutrally and 4.2% disagreed. When asked “I would recommend that other persons with ALS consider genetic testing” 83.3% agreed, 12.5% responded neutrally and 1.1% disagreed.

**Cognition**

There were 2 studies that reported data related to cognition. In the cross-sectional survey of Conroy 2023^4^ only 6 adults diagnosed with ALS (total not specified) reported that they wanted their current ALS treatments to impact their cognitive ability (improvement in focus and thinking). In the structured interview of Hecht 2002^8^ (methods above) unimpaired consciousness was only identified as the worst aspect of the disease by 2.4% of adults with ALS at baseline, and by 3.2% of adults with ALS at a 6-month follow-up interview.

**Psychological and emotional wellbeing**

**Mental health and mood**

There were two studies that provided outcome data related to mental health and mood. Conroy 2023^4^ reported that 4 adults diagnosed with ALS (total not specified) reported that they wanted their current ALS treatments to impact their overall mental health. Tarlarini 2017^18^ asked adults diagnosed with MND, some that were enteral-tube fed and others that were oral-fed (of a larger cohort study investigating taste perception – details below) the importance of 11 different aspects on their overall quality of life, with the score ranging from 0 (least important) to 100 (most important). Regarding ‘mood’ the mean importance of this aspect towards overall quality of life was 36.4% for adults with ALS that were enteral-fed and 38.1% for those that were orally-fed.

**Provision of emotional support**

Only the single study provided data regarding emotional support. Tobin 2021^20^ conducted a discrete choice experiment where both adults diagnosed with ALS and caregivers of people with ALS were provided with a series of ‘choice sets’. Each choice-set consisted of at least two hypothetical packages of care, and participants were asked to choose the package that they preferred. The data combines the responses of adults diagnosed with ALS, with the responses provided by carers and has been presented as the magnitude of attribute coefficients and their associated 95% confidence interval, against the reference hypothetical package of care. The authors of this study have reported their data according to sex, site of onset (bulbar or spinal), diagnosis (≤ 6 months or > 6 months) and King’s staging (stage 1 and 2 or stage 3 and 4). The data reported here is that of the King’s staging. Compared to the reference package of care (Emotional support is not provided), the hypothetical choice of “Emotional support is provided by group meetings with other MND patients” was seen as less preferable (for both adults with ALS and caregivers of adults with ALS) for those at stage 1 or 2 (-0.02, 95%CI: -0.41 to 0.28), but was seen as more preferable for those at stage 3 or 4 (0.05, 95%CI: -0.17 to 0.28), however both these values were not statistically significant. Compared to the reference package of care, the hypothetical choice of “Emotional support is provided from a counsellor” was seen as more preferable for those at stage 1 or 2 (0.31, 95%CI: 0.00 to 0.62), but was seen as less preferable for those at stage 3 or 4 (-0.06, 95%CI: -0.24 to 0.11), however both these values were not statistically significant.

**Emotional swings**

There were two studies that provided outcome data related to emotional swings. Trail 2004^21^ (methods above) have stated that 16.7% of adults with ALS, and 13.7% of caregivers of adults with ALS reported emotional swings as one of their top three concerns. In the cross-sectional survey of Conroy 2023^4^ only 1 adult diagnosed with ALS (total not specified) reported that they wanted their current ALS treatments to impact their uncontrollable laughing/crying.

**Anxiety**

Only the single study provided data regarding anxiety. In Tarlarini 2017^18^ (methods above), the mean importance of anxiety towards overall quality of life was 45.4% for adults with ALS that were enteral-fed and 28.6% for those that were orally-fed.

**Depression**

Only the single study provided data regarding depression. Trail 2004^21^ (methods above) have stated that 3% of adults with ALS, and 5.1% of caregivers of adults with ALS reported depression as one of their top three concerns.

**Loss faith**

Only the single study provided data regarding loss of faith. Trail 2004^21^ (methods above) have stated that 1.5% of adults diagnosed with ALS reported ‘loss of faith’ as one of their top three concerns. No caregivers of adults diagnosed with ALS reported this as one of their top three concerns.

**Apathy**

Only the single study provided data regarding apathy. In the cross-sectional survey of Conroy 2023^4^ only 3 adults diagnosed with ALS (total not specified) reported that they wanted their current ALS treatments to impact their apathy (loss of interest).

**Financial burden**

There were two studies that provided outcome data related to financial burden. Trail 2004^21^ (methods above) have stated that 16.7% of adults with ALS, and 13.7% of caregivers of adults with ALS reported financial stresses as one of their top three concerns. In the discrete choice experiment of Tobin 2021^20^ (methods above) the authors have only narratively reported that participants (both adults with ALS and caregivers of adult with ALS) showed no strong preference for the price of additional services in the inclusion in their healthcare package.

**Concern for future**

There were two studies that provided outcome data related to ‘concern for future’. Akerblom 2014^3^ conducted a semi-structured interview with adults diagnosed with ALS where participants were asked to identify the top five most important areas impacting their quality of life. The responses to this question were reported according to those with and without current pain. Only 2 (5%) adults with ALS that were in pain reported ‘hope for the future’ as one of their top five most important concerns impacting their quality of live. No adults with ALS that were without pain reported ‘hope for the future’ as one of their top five most important concerns impacting their quality of life. Trail 2004^21^ (methods above) have stated that 16.7% of adults with ALS, and 25.4% of caregivers of adults with ALS reported being worried about the future as one of their top three concerns.

**Swallowing and nutrition**

**Eating score**

Only the single study provided data regarding eating score. Raheja 2016^13^ have analysed a retrospective sample of participants of a previous multi-centre randomised controlled trial across North America investigating the effectiveness of ceftriaxone. Participants were assessed on the ALSSQoL which assess six domains of health-related quality of life using a 0-10 Likert scale and asks participants to consider the current determinants of their own quality of life (0 worst quality of life, 10 being best quality of life). This secondary analysis included only participants that provided baseline data and follow-up data at 96 weeks post randomisation. All participants, regardless of intervention assignment in the original RCT have been included in the reported data (the study did not show efficacy). The mean eating score of adults with ALS was 0.91 (± 1.54) at baseline and was 2.54 (± 3.09) at week 96.

**Flavour perception**

Only the single study provided data regarding flavour perception. Tarlarini 2017^18^ have reported data to this outcome using different methods to those reported above (and below). In this component of their study, participants were asked directly, “how much is flavour perception important for you”, which was answered using a visual analogue scale where a score of 0 corresponded to “a little” and 100 corresponded to ‘a lot’. Unlike the other outcomes reported from this study, this outcome has not been reported separately for enteral-fed participants from orally-fed participants. Tarlarini 2017^18^ have reported narratively 24 of 32 (75%) adults with ALS reported a score equal or higher than 75.

**Taste**

Only the single study provided data regarding taste. Tarlarini 2017^18^ (methods above) reported the mean importance of taste towards overall quality of life was 54.5% for adults with ALS that were enteral-fed and 76.19% for those that were orally-fed.

**Swallowing and chewing**

There were four separate studies that provided outcome data related to swallowing and chewing, the methods of which have all been reported earlier. Conroy 2023^4^ have reported that 7 adults with ALS (total not specified) reported that they wanted their current ALS treatments to impact their swallowing (positively). Trail 2004^21^ (methods above) have stated that 19.7% of adults with ALS reported “problems with swallowing/eating” as one of their top three concerns, and 30.5% of caregivers of adults with ALS reported this as one of their top three concerns. In the structured interview of Hecht 2002^8^ (methods above) swallowing problems was identified as the worst aspect of the disease by only 2.4% of adults with ALS at baseline, and by 3.2% of adults with ALS at a 6-month follow-up interview. Finally, Tarlarini 2017^18^ (methods above), have reported two outcomes that have been grouped under this category. The mean importance of swallowing and chewing towards overall quality of life was 100% and 90.91% for adults with ALS that were enteral-fed respectively. For adults that were orally-fed the mean importacet towards quality of life for swallowing was 28.57% and was 9.52% for chewing.

**Body weight**

Only the single study reported outcome data related to body weight. Helleman 2022^9^ conducted a cross-sectional survey which presented adults with ALS, PLS or PMA with a list of seven outcome measures, of which patients had to rank a top three for their most valuable outcome measures. The outcome data has been reported using a normalised ranking score (with 0 being the least valuable outcome and 1 being the most valuable outcome) according to the participating countries and across all countries. The outcome of ‘body weight’ had a normalised ranking score of 0.16 (all countries), 0.15 (Netherlands), 0.17 (United Kingdom) and 0.21 (Australia).

**Personal care and daily activities**

**Independence**

This outcome was reported by three individual studies. In the structured interview of Hecht 2002^8^ (methods above) ‘becoming dependant on other persons’ was identified as the worst aspect of the disease by 12.2% of adults with ALS at baseline, and by 9.7% of adults with ALS at a 6-month follow-up interview. In the semi-structured interview of Akerblom 2014^3^ 9 (22%) of adults with ALS that were in pain and 4 (28%) adults with ALS that were not in pain reported ‘being independent’ as one of their top five most important concerns impacting their quality of life. Finally, Trail 2004^21^ (methods above) have stated that 37.9% of adults with ALS, and 6.8% of caregivers of adults with ALS reported independence as one of their top three concerns.

**Recreation**

Only the single study reported outcome data related to recreation. Tarlarini 2017^18^ (methods above), have reported that the mean importance of ‘recreation’ towards overall quality of life was 45.4% for adults with ALS that were enteral-fed and 52.4% for those that were orally-fed.

**Ability to work**

There were two studies that provided outcome data related to ability to work. In the semi-structured interview of Akerblom 2014^3^, 9 (22%) adults with ALS that were in pain and 2 (14%) adults with ALS that were not in pain reported ‘being able to work’ as one of their top five most important concerns impacting their quality of life. Trail 2004^21^ (methods above) have stated that 10.6 % of adults with ALS reported ‘inability to work’ as one of their top three concerns but only 1.7% of caregivers of adults with ALS reported this as one of their top three concerns.

**Daily self-care**

There were two studies that provided outcome data related to self-care. Conroy 2023^4^ have reported that 28 adults with ALS (total not specified) reported that they wanted their current ALS treatments to impact their daily self-care (positively). Trail 2004^21^ (methods above) reported that 18.2% of adults with ALS, and 22% of caregivers of adults with ALS reported ‘difficulty with self-care (i.e. dressing, bathing)’ as one of their top three concerns.

**Ability to write**

Only the single study reported outcome data related to ability to write. Hecht 2002^8^ (methods above) reported ‘loss of writing ability’ was identified as the worst aspect of the disease by 2.4% of adults with ALS at baseline, and by 3.2% of adults with ALS at a 6-month follow-up interview.

**Appearance**

Only the single study reported outcome data related to appearance. Tarlarini 2017^18^ (methods above), have reported that the mean importance of ‘appearance’ towards overall quality of life was 54.5% for adults with ALS that were enteral-fed and 4.76% for those that were orally-fed.

**Being able to travel**

Only the single study reported outcome data related to being able to travel. In the semi-structured interview of Akerblom 2014^3^ 4 (10%) adults with ALS that were in pain and 1 (7%) adult with ALS that was not in pain reported ‘being able to travel’ as one of their top five most important concerns impacting their quality of life.

**Personal care provision**

Only the single study reported outcome data related to personal care provision. In the discrete choice experiment of Tobin 2021^20^ (methods above) compared to the reference package of care (personal care in the home is provided to me by someone who is not a relative or friend), the hypothetical choice of “personal care in the home is provided by a relative or friend” was seen as less preferable (for both adults with ALS and caregivers of adults with ALS) for those at stage 1 or 2 (-0.26, 95%CI: -0.64 to 0.13), but was seen as more preferable for those at stage 3 or 4 (0.04, 95%CI: -0.19 to 0.28), however both these values were not statistically significant.

**Symptom management**

**Pain**

There were three studies that reported data towards the outcome of pain. Conroy 2023^4^ have reported that only 1 adult with ALS (total not specified) reported that they wanted their current ALS treatments to impact their pain (positively). Tarlarini 2017^18^ (methods above), have reported that the mean importance of ‘pain’ towards overall quality of life was 9.1% for adults with ALS that were enteral-fed and 9.5% for those that were orally-fed. Finally, the retrospective analysis of Raheja 2016^13^ have reported the mean pain score of adults with ALS was 1.6 (± 2.27) at baseline and 2.5 (± 2.37) at week 96.

**Breathing**

Outcome data regarding breathing was reported in four of the included studies. Conroy 2023^4^ have reported that 90 adults with ALS (total not specified) reported that they wanted their current ALS treatments to impact their breathing and respiratory function (positively). Trail 2004^21^ (methods above) reported that 13.6% of adults with ALS, and 25.4% of caregivers of adults with ALS reported ‘problems breathing’ as one of their top three concerns. Helleman 2022^9^ (methods above) have reported the outcome of ‘respiratory function’ had a normalised ranking score of 0.63 (all countries), 0.63 (Netherlands), 0.59 (United Kingdom) and 0.5 (Australia). Finally, the retrospective analysis of Raheja 2016^13^ have reported the mean breathing score of adults with ALS was 0.67 (± 1.33) at baseline and 2.83 (± 2.77) at week 96.

**Muscle spasticity**

Only the single study reported outcome data related to muscle spasticity. Conroy 2023^4^ state that only 2 adults with ALS (total not specified) reported that they wanted their current ALS treatments to impact their muscle spasticity (positively).

**Muscle strength**

Muscle strength has been reported by four individual studies. Helleman 2022^9^ (methods above) have reported the outcome of ‘muscle strength’ had a normalised ranking score of 0.39 (all countries), 0.39 (Netherlands), 0.34 (United Kingdom) and 0.39 (Australia). Trail 2004^21^ (methods above) reported that 25.8% of adults with ALS, and 22% of caregivers of adults with ALS reported ‘muscle weakness’ as one of their top three concerns. In the retrospective analysis of Raheja 2016^13^ the mean strength/ability to move score of adults with ALS was 3.96 (± 2.57) at baseline and 6.46 (± 2.36) at week 96. Finally, Conroy 2023^4^ (methods above) have stated that 23 adults with ALS (total not specified) reported that they wanted their current ALS treatments to impact their muscle weakness (positively). Additionally, Conroy 2023^4^ presented their participants the outcomes of a hypothetical new drug (noting this is different to the previous question – which asked them to consider their current treatment). Participants ranked a series of outcomes related to this hypothetical new drug most to least important, 23.9% of adults with ALS indicated that “the drug increases muscles strength, even if it has moderate to severe side effects that are difficult to manage” as the most important outcome. In addition, 24.5% of adults with ALS indicated that “The drug prevents further muscle weakening but does not improve strength” as the most important outcome.

**Improved symptoms**

Only two studies reported outcome data regarding how adults with ALS valued the possible improvement of their symptoms. When participants of the study conducted by Conroy 2023^4^ were presented with the outcomes of a hypothetical new drug, 28.9% indicated the most important outcome would be “the drug slows (but does not halt) the spread of ALS/MND and has minimal-moderate side effects” in the same study 14.2% of participants reported that “reduction of symptoms” was considered to be the most important outcome. In the component of the study where adults with ALS were asked to consider the most important outcomes in which they want their current ALS treatment to target,^4^ 20 people stated “ease the overall burden of ALS/MND” and 161 people stated “stop the progression of ALS/MND” (total unspecified for both).

Green 2003^6^ conducted a standard gamble-based utility elicitation study. Adults with ALS were asked to consider the certain outcome of living for two years in their present health state versus the uncertain outcome, involving varying probabilities of returning to full health or risking certain death. The results have been presented on a scale of 0-1 (with 1 indicating a greater probability of choosing to gamble), and organised according to disease severity, staged using the ALS Health State Scale. The mean probability of choosing to gamble was 0.79 (±0.15), 0.67 (±0.24), 0.71 (±0.28) and 0.45 (±0.24) for adults with MND at levels 1, 2, 3 and 4 respectively.

**Fatigue**

Only the single study reported outcome data related to fatigue. Conroy 2023^4^ (methods above) have stated that 15 adults with ALS (total not specified) reported that they wanted their current ALS treatments to impact their fatigue (positively). In the retrospective analysis of Raheja 2016^13^ the mean fatigue score of adults with ALS was 3.24 (± 2.24) at baseline and 4.38 (± 2.22) at week 96.

**Saliva**

There were two studies that provided outcome data related to saliva. In the study of Tarlarini 2017^18^ (methods above), the mean importance of saliva towards overall quality of life was 18.18% for adults with ALS that were enteral-fed and 4.76% for those that were orally-fed. In the retrospective analysis of Raheja 2016^13^ the mean excessive score of adults with ALS was 1.12 (± 1.92) at baseline and 2.54 (± 2.93) at week 96.

**Bowel and bladder**

Only the single study reported outcome data related to bowel and bladder. Raheja 2016,^13^ state that the mean excessive bowel and bladder score of adults with ALS was 1.12 (± 1.92) at baseline and 2.54 (± 2.93) at week 96.

**Mucus**

Only the single study reported outcome data related to mucus. Raheja 2016,^13^ state that the mean mucus score of adults with ALS was 1.24 (± 1.73) at baseline and 2.74 (± 2.84) at week 96.

**Sleep**

There were two studies that provided outcome data related to sleep. Helleman 2022^9^ have reported the outcome of ‘sleep’ had a normalised ranking score of 0.17 (all countries), 0.13 (Netherlands), 0.11 (United Kingdom) and 0.39 (Australia). In the retrospective analysis of Raheja 2016,^13^ who have stated that the mean sleep score of adults with ALS was 1.44 (±1.9) at baseline and 2.85 (±2.54) at week 96.

**Disease modifying therapies**

**Riluzole**

The following outcome valuations are related to the use (or non-use) of specific disease modifying therapies. Rudnicki 1997^14^ have conducted what was assumed to be a semi-structured interview (methodological details were not clear) of adults with ALS that had previous participated in an early access program for riluzole. If a patient chose to not take riluzole at any point, they were asked to provide their reasoning. The following reasons were provided: ‘insufficient benefit’ (n = 14), ‘high cost’ (n = 9), ‘do not want to prolong life’ (n = 8), ‘doing worse’ (n = 2), ‘potential side effects’ (n = 2), ‘lack of improved quality of life’ (n = 1) and ‘participation in another study’ (n = 1).

**Intrathecal drug delivery**

Seo 2024^16^ conducted a discrete choice experiment and threshold technique exercise, where adults with ALS were presented with descriptions of an intrathecal drug delivery device (IDDD) and lumbar puncture options for intrathecal drug delivery. Within the discrete choice experiment, participants were asked to repeatedly make choices between a lumbar puncture profile and two IDDD profiles. Each profile was described by the overall duration, location, frequency, risk associated with each injection, and device failure risk. The scenarios presented to participants were as follows:

| Attribute | Scenario 1 (LP / IDDD) | Scenario 2 (LP / IDDD) | Scenario 3 (LP / IDDD) | Scenario 4 (LP / IDDD) |
| --- | --- | --- | --- | --- |
| **Overall duration** | 2 hr / 1 hr | 1 hr / 1 hr | 2 hr / 90 min | 1 hr / 90 min |
| **Location** | Hospital / Local clinic | ALS Hospital / Local clinic | Hospital / Hospital | ALS Hospital / Hospital |
| **Frequency** | Every month / Every month | Every 4 months / Every 4 months | Every month / Every month | Every month / Every month |
| **Administration risk** | 1% / 2% | 1% / 3% | 1% / 1% | 1% / 1% |
| **Risk of device failure** | n/a / 13% | n/a / 15% | n/a / 13% | n/a / 13% |
| **Risk of implantation surgery** | n/a / 5% | n/a / 2% | n/a / 2% | n/a / 5% |

The predicted choice probability for scenario 1 was 18.2% (±4.2%) [lumbar puncture] and 81.8% (±4.2%) [IDDD]. In scenario 2, it was 31.1% (±6.2%) [lumbar puncture] and 68.9% (±6.2%) [IDDD]. In scenario 3, it was 27% (±4.6%) [lumbar puncture] and 73% (±4.6%) [IDDD]. In scenario 4, it was 48% (±6.9%) [lumbar puncture] and 52% (±6.9%) [IDDD]. The authors also report narratively that an IDDD option was preferred over lumbar puncture in 78.7% of the discrete choice experiment tasks.

**Communication and expression**

**Speech**

Several different studies provided valuation towards outcomes related to speech, all six of which have been described previously. Helleman 2022^9^ have reported the outcome of ‘fine motor/speech’ had a normalised ranking score of 0.22 (all countries), 0.22 (Netherlands), 0.25 (United Kingdom) and 0.19 (Australia). In the structured interview of Hecht 2002^8^ ‘reduction of speech’ was identified as the worst aspect of the disease by 29.3% of adults with ALS at baseline, and by 19.4% of adults with ALS at a 6-month follow-up interview. Tarlarini 2017^18^ have reported, the mean importance of ‘speech’ towards overall quality of life was 90.91% for adults with ALS that were enteral-fed and 42.86% for those that were orally-fed. In the study of Trail 2004,^21^ the authors have reported that 27.3% of adults with ALS, and 17% of caregivers of adults with ALS reported ‘problems speaking’ as one of their top three concerns. In the retrospective analysis of Raheja 2016^13^ it was reported the mean speaking score of adults with ALS was 1.85 (± 2.59) at baseline and 4.3 (± 3.77) at week 96. Finally, Conroy 2023^4^ have stated that 33 adults with ALS (total not specified) reported that they wanted their current ALS treatments to impact their speaking/communication (positively).

**Use of communication technologies**

Only the single study reported outcome data related to the use of communication technologies. The discrete choice experiment of Tobin 2021,^20^ compared to the reference package of care (‘I would use new communication technology including voice banking’), the hypothetical choice of “I would not use new communication technology including voice banking” was seen as more preferable (for adults with ALS) for those at stage 1 or 2 (-0.04, 95%CI: -0.32 to 0.23), and at stage 3 or 4 (-0.17, 95%CI: -0.34 to 0.01), however both these values were not statistically significant. However, when grouped by sex, female patients did demonstrate a strong preference for using communication devices (-0.25, 95% CI: -0.47, -0.21). Males did not show a strong preference for any attribute.

**Healthcare experiences**

**Availability of phone advice**

Only the single study reported outcome data related to the use of availability of phone advice. The discrete choice experiment of Tobin 2021,^20^ compared to the reference package of care (‘phone advice is not available’), the hypothetical choice of “phone advice is available 24 hours a day was seen as more preferable (for adults with ALS and their caregivers) for those at stage 1 or 2 (0.03, 95%CI: -0.35 to 0.41), and at stage 3 or 4 (0.05, 95%CI: -0.18 to 0.28), however both these values were not statistically significant. Meanwhile, the hypothetical choice of “phone advice is available during the hours of 9am – 5pm Monday to Friday” was more preferable (for adults with ALS and their caregivers) for those at stage 1 or 2 (0.14, 95%CI:-0.15 to 0.43) but less preferable for those as stage 3 or 4 (-0.17, 95%CI: -0.33 to 0.00).

**Dependence on healthcare providers**

Only the single study reported outcome data related to the use of healthcare providers. The discrete choice experiment of Tobin 2021,^20^ compared to the reference package of care (‘I would have healthcare professionals who I can depend on’), the hypothetical choice of “I would not necessarily need healthcare professionals to depend on” was seen as less preferable (for adults with ALS and their caregivers) for those at stage 1 or 2 (0.36, 95%CI: -0.00 to 0.72), and at stage 3 or 4 (0.02, 95%CI: -0.20 to 0.25), however both these values were not statistically significant.

**Disease information**

Only the single study reported outcome data related to the use of disease information. The discrete choice experiment of Tobin 2021,^20^ compared to the reference package of care (‘I would get all the information about motor neuron disease at the time of diagnosis’), the hypothetical choice of I would get all information about motor neuron disease when I think I will need it” stage 1 or 2 participants showed a strong preference for receiving information about ALS at the time of diagnosis, rather than at a later stage (when clinically relevant)(-0.51, 95%CI: -0.99 to -0.05). However, this was not the case for those at stage 3 or 4 (-0.12, 95%CI: -0.37 to 0.13). Additionally, the reference choice of was also strongly preferred against the hypothetical choice of “I don’t want to know anything about motor neuron disease at any time” for those at stage 1 or 2 (-0.57 95%CI: -1.04 to -0.10), but no statistical significance was observed for those at stage 3 or 4 (-0.05 95%CI: -0.31 to 0.21).

**Timing of hospice care**

Only the single study reported outcome data related to the use of timing of hospice care. The discrete choice experiment of Tobin 2021,^20^ compared to the reference package of care ‘I would start to see the Hospice Care team early on in my illness’, the hypothetical choice of “I would start to see the Hospice Care team when something serious happens later in my illness” was seen as more preferable for those at stage 1 or 2 (0.24, 95%CI: -0.05 to 0.53), and for those at stage 3 or 4 (0.14, 95%CI: -0.03 to 0.30). However, both these values were not statistically significant.

**Waiting times**

Only the single study reported outcome data related to the use of timing of waiting times. The discrete choice experiment of Tobin 2021,^20^ compared to the reference package of care ‘I would be prepared to go to a multidisciplinary clinic with long waiting times in the clinic to see more than one professional’, the hypothetical choice of “I would not be prepared to go to a multidisciplinary clinic with long waiting times in the clinic to see more than one professional” was seen as less preferable for those at stage 1 or 2 (-0.05, 95%CI: -0.39 to 0.29), but was seen as more preferable for those at stage 3 or 4 (0.06, 95%CI: -0.14 to 0.27). However, both these values were not statistically significant.

**Helplessness of medicine**

Only the single study reported outcome data related to the helplessness of medicine. The structured interview of Hecht 2002^8^ the ‘helplessness of medicine’ was only identified as the worst aspect of the disease by 2.4% of adults with ALS at baseline, and by 3.2% of adults with ALS at a 6-month follow-up interview.

**Prognosis**

**Survival (prolonging of life)**

Survival was an outcome reported in three different studies. Adelman 2004^2^ conducted a structured interview of adults with ALS and their caregivers every month until the person with ALS died, or underwent a tracheostomy. Responses to the questions asked as part of this interview were scored using a visual analogue scale from 0 (the least intensity) to 10 (greatest intensity). The first assessment of all pairs, and the last assessment have been reported as means. In response to the question “How much does [the person with ALS] want to live?”, at the first assessment, the mean score for people with ALS was 7.8 (±3.1) and for caregivers was 8.0 (±3.0). At the last assessment, the mean score for people with ALS was 7.5 (±3.4) and for caregivers was 6.9 (±3.3). Maksymowicz-Sliwinska 2023^11^ conducted a cross-sectional survey of caregivers of adults with ALS. Participants were asked a series of questions that could be answered with a) definitely not, b) probably not, c) probably yes, d) definitely yes. In response to the question “would you like your relative with ALS to use life-prolonging measures?” 30.5% responded with definitely not or probably not, and 69.5% responded with probably yes or definitely yes. Finally, when participants of the study conducted by Conroy 2023^4^ were presented with the outcomes of a hypothetical new drug, 8.5% indicated the most important outcome would be “the drug prolongs survival but has moderate to severe side effects that some people cannot tolerate”.

**Hastening death**

There were two studies that provided outcome data related to hastening death. Adelman 2004^2^ have stated that in response to the question “How interested is the patient in hastening their death?”, at the first assessment, the mean score for people with ALS was 3.2 (± 3.3) and for caregivers was 2.7 (± 2.8). At the last assessment, the mean score for people with ALS was 3.1 (±3.3) and for caregivers was 2.7 (± 2.8). Maksymowicz-Sliwinska 2023^11^ reported that in response to the question “if it were permitted by law, would you like your relative with ALS to use life-shortening measures?” 61.3% of caregivers responded with definitely not or probably not and 38.7% of caregivers responded with probably yes or definitely yes.

**Social and Interpersonal relationships**

**Isolation and loneliness**

There were two studies that provided outcome data related to isolation and loneliness. In the structured interview of Hecht 2002^8^ the authors have reported that ‘*social* *isolation’* was not identified as the worst aspect of the disease by any adult with ALS at baseline, but by 6.5% of adults with ALS at a 6-month follow-up interview. Trail 2004^21^ have stated that 3% of adults with ALS and 3% of caregivers of adults with ALS reported ‘*loneliness’* as one of their top three concerns.

**Who helps in the home**

Only the single study reported outcome data related to who helps in the home. The discrete choice experiment of Tobin 2021,^20^ have only narratively reported that participants (both adults with ALS and caregivers of adult with ALS) showed no strong preference regarding if non-related individuals help in the home.

**Sexual function**

Only the single study reported outcome data related to sexual function. Conroy 2023^4^ have reported that only the single adult with ALS (total not specified) reported that they wanted their current ALS treatments to impact their sexual function (positively).

**Domain importance**

Outcomes related to ‘domain importance’ were reported in the studies by Van Eijk 2022^23^ and 2024^22^. Both of these studies involved a cross-sectional survey, where adults with ALS were asked to rank their personal importance for functional domains of health. In response to the question “which domain is most important to you”, the 2022 study identified the most important domain reported by the patients was respiratory (37.5%), followed by bulbar (30.9%), gross motor (20.4%) and fine motor (11.2%). The 2024 study identified that most participants had no preference (i.e. all domains were equal) (37%), followed by respiratory (23%), bulbar (22%), gross motor (10%) and fine motor (8%).

**Supplementary material 8 - Sensitivity analyses**

Health-related quality for adults diagnosed with ALS at different stage of disease progression (King’s) – Fixed Effect Model

| Study ID | N | Weight % | Mean [95%CI:] |
| --- | --- | --- | --- |
| Stenson 2024 | 29 | 13.93 | 0.65 (0.61, 0.69) |
| Moore 2019 | 89 | 10.37 | 0.76 (0.72, 0.80) |
| Floating Kings Stage 1 |  |  | 0.71 (0.68, 0.74) |
| Stenson 2024 | 27 | 14.04 | 0.51 (0.47, 0.55) |
| Moore 2019 | 135 | 12.99 | 0.60 (0.56, 0.64) |
| Floating Kings Stage 2 |  |  | 0.56 (0.54, 0.59) |
| Stenson 2024 | 56 | 13.33 | 0.45 (0.41, 0.49) |
| Moore 2019 | 206 | 22.95 | 0.53 (0.5, 0.56) |
| Floating Kings Stage 3 |  |  | 0.49 (0.47, 0.52) |
| Stenson 2024 | 50 | 2.15 | 0.11 (0.01, 0.21) |
| Moore 2019 | 162 | 10.24 | 0.5 (0.46, 0.54) |
| Floating Kings Stage 4 |  |  | 0.40 (0.36, 0.44) |
| Overall |  |  | 0.55 (0.54, 0.57) |

Health-related quality for adults diagnosed with ALS at different stage of disease progression (MiToS) – Fixed Effect Model

| Study ID | N | Weight % | Mean [95%CI:] |
| --- | --- | --- | --- |
| Stenson 2024 | 116 | 38.48% | 0.53 (0.51, 0.55) |
| Moore 2019 | 301 | 9.23% | 0.71 (0.67, 0.75) |
| Mito's Stage 0 Estimate |  |  | 0.56 (0.55, 0.58) |
| Stenson 2024 | 17 | 0.99% | 0.34 (0.20, 0.48) |
| Moore 2019 | 198 | 11.67% | 0.48 (0.44, 0.52) |
| Mito's Stage 1 Estimate |  |  | 0.47 (0.43, 0.51) |
| Stenson 2024 | 10 | 0.11% | 0.00 (-0.42, 0.42) |
| Moore 2019 | 73 | 20.66% | 0.36 (0.33, 0.39) |
| Mito's Stage 2 Estimate |  |  | 0.36 (0.33, 0.39) |
| Stenson 2024 | 8 | 0.06% | 0.01 (-0.55, 0.57) |
| Moore 2019 | 18 | 9.29% | 0.33 (0.29, 0.37) |
| Mito's Stage 3 Estimate |  |  | 0.33 (0.28, 0.37) |
| Stenson 2024 | 14 | 0.17% | -0.10 (-0.43, 0.23) |
| Moore 2019 | 5 | 9.34% | 0.24 (0.20, 0.28) |
| Mito's Stage 4 Estimate |  |  | 0.23 (0.19, 0.28) |
| Overall |  |  | 0.46 (0.44, 0.47) |

Health-related quality for adults diagnosed with ALS at different stage of disease progression (ALS HSS) – Fixed Effect Model

| Study ID | N | Weight % | Mean [95%CI:] |
| --- | --- | --- | --- |
| Green 2003 | 15 | 17.26% | 0.74 (0.64, 0.84) |
| Level 1 Estimate |  |  | 0.74 (0.64, 0.84) |
| Green 2003 | 24 | 30.18% | 0.63 (0.56, 0.70) |
| Level 2 Estimate |  |  | 0.63 (0.56, 0.70) |
| Green 2003 | 22 | 28.20% | 0.51 (0.43, 0.59) |
| Level 3 Estimate |  |  | 0.51 (0.43, 0.59) |
| Green 2003 | 19 | 24.36% | 0.37 (0.29, 0.45) |
| Level 4 Estimate |  |  | 0.37 (0.29, 0.45) |
| Overall |  |  | 0.55 (0.51, 0.59) |

Health-related quality for adults diagnosed with ALS at different stage of disease progression (FT9) – Fixed Effect Model

| Study ID | N | Weight % | Mean [95%CI:] |
| --- | --- | --- | --- |
| Thakore 2019 | 160 | 14.92% | 0.79 (0.76, 0.81) |
| FT9 0 Estimate |  |  | 0.79 (0.76, 0.81) |
| Thakore 2019 | 494 | 31.14% | 0.74 (0.73, 0.76) |
| FT9 1 Estimate |  |  | 0.74 (0.73, 0.76) |
| Thakore 2019 | 542 | 25.22% | 0.63 (0.61, 0.65) |
| FT9 2 Estimate |  |  | 0.63 (0.61, 0.65) |
| Thakore 2019 | 381 | 17.51% | 0.54 (0.52, 0.56) |
| FT9 3 Estimate |  |  | 0.54 (0.52, 0.56) |
| Thakore 2019 | 291 | 11.21% | 0.46 (0.43, 0.49) |
| FT9 4 Estimate |  |  | 0.46 (0.43, 0.49) |
| Overall |  |  | 0.65 (0.64, 0.66) |

**Supplementary material 9 - Transformations to facilitate synthesis**

**Helleman 2022**

The authors reported outcome ranking scores in a figure (on a scale of 0 to 1) and we were not able to extract the specific ranking scores for each outcome. We reached out to the author and received raw excel data and calculated outcome ranking scores manually.

The outcome ranking assessment item required respondents to rank three of the seven proposed home measures (respiratory function, muscle strength, quality of life, fine motor/speech, daily activity, sleep and body weight) for home monitoring from most valuable to least valuable. Rank 1 received a score of 3, rank 2 a scores of 2 and rank 3 a score of 1; measures not listed in the top 3 received a score of 0. The total rank score of each home measure was subsequently calculated by taking the sum of the rank scores and were divided by the maximal possible score (all patients ranked a measure as 1st), resulting in a score between 0 and 1. For the comparisons between countries, the total rank scores of dividing them by the highest possible score.

|  | 1*st* | 2*nd* | 3*rd* | Total and ranking score |
| --- | --- | --- | --- | --- |
| Respiratory function | 163*3 = 489 | 31*2 =62 | 21*1 | 572/996 =0.574 |
| Muscle strength | 36*3 = 108 | 84*2= 168 | 65*1 | 341/996 =0.342 |
| Quality of life | 31*3 = 93 | 31*2 = 62 | 55*1 | 210/996 =0.211 |
| Fine motor/speech | 17*3 = 51 | 49*2 = 98 | 56*1 | 205/996 = 0.206 |
| Daily activity | 26*3 = 78 | 39*2 =78 | 36*1 | 192/996 = 0.192 |
| Sleep | 16*3 = 48 | 35*2 = 70 | 40*1 | 158/996 =0.158 |
| Weight | 22*3 = 66 | 32*2 =64 | 20*1 | 150/996 =0.151 |

**Moore 2019**

The authors have reported EQ-5D-5L data in the mean (95%CI) format [0.57(0.55-0.59)]. The study had a sample size of 595. To facilitate meta-analysis this needed to be converted to standard deviation according to the following:

$$SD\sim\frac{(0.59-0.55) \times\sqrt{595}}{2 \times1.96}= \frac{0.04\times24.3926}{3.92}= \frac{0.9757}{3.92} \sim0.2488$$

**Stenson 2004**

The authors have only presented their data per group visually in a column graph. While means have been reported on this graph. The 95% confidence intervals have only been drawn on. The approximate value for the upper and lower confidence intervals have been digitised using the GetData Graph Digitiser to facilitate meta-analysis.

These values are as follows for the King’s staging data:

| Group | Mean | CI Lower | CI Upper | n | Estimated SD |
| --- | --- | --- | --- | --- | --- |
| 1 | 0.65 | 0.61 | 0.69 | 29 | 0.1052 |
| 2 | 0.51 | 0.47 | 0.55 | 27 | 0.1011 |
| 3 | 0.45 | 0.41 | 0.49 | 56 | 0.1494 |
| 4 | 0.11 | 0.01 | 0.21 | 50 | 0.3519 |

These values are as follows for the MiToS staging data:

| Group | Mean | CI Lower | CI Upper | n | Estimated SD |
| --- | --- | --- | --- | --- | --- |
| 0 | 0.53 | 0.49 | 0.57 | 116 | 0.121 |
| 1 | 0.34 | 0.27 | 0.41 | 17 | 0.289 |
| 2 | 0.00 | -0.15 | 0.15 | 10 | 0.671 |
| 3 | 0.01 | -0.17 | 0.19 | 8 | 0.802 |
| 4 | -0.10 | -0.27 | 0.07 | 14 | 0.632 |

**Wei 2021**

The authors have not reported data according to the entire sample, only data to various subgroups. To facilitate meta-analysis, we are utilising the data for Spinal Onset ALS, as it was the most populous subgroup (n=441), reported as Median of 0.73 and IQR (0.56 to 0.86).

To facilitate meta-analysis, these values then needed to be converted to an estimated mean and standard deviation.^31^

$$Mean\sim\frac{Q1+Median+Q3}{3}= \frac{0.56+0.73+0.86}{3}= \frac{2.15}{3} \sim0.7167$$

$$SD\sim\frac{Q3-Q1}{1.35}= \frac{0.86-0.56}{1.35}= \frac{0.3}{1.35} \sim0.2222$$

$$SE\sim\frac{SD}{\sqrt{n}}= \frac{0.2222}{\sqrt{441}}= \frac{0.2222}{21} \sim0.01058$$

$${CI}_{lower}= 0.7167-(1.96 \times0.01058)= 0.69595$$

$${CI}_{upper}= 0.7167+(1.96 \times0.01058)= 0.73745$$

**Supplementary material 10 - R Code**

**Supplementary figure 1**

```
library(metafor)
## Warning: package 'metafor' was built under R version 4.2.3
## Loading required package: Matrix
## Loading required package: metadat
## Warning: package 'metadat' was built under R version 4.2.3
## Loading required package: numDeriv
##
## Loading the 'metafor' package (version 4.4-0). For an
## introduction to the package please type: help(metafor)
# Input data
set.seed(123)
dat <- data.frame(
  Study = c("Moore 2019", "Wei 2021"),
  Group = c("UK Set", "China Set"),
  Mean = c(0.57, 0.7167),
  SD = c(0.2488, 0.2222),
  N = c(595, 441)
)

# Calculate effect sizes
dat_es <- escalc(measure = "MN", mi = Mean, sdi = SD, ni = N,
                 slab = dat$Study, data = dat)

# Fit full model (fixed effect, Inverse Variance)
res_all <- rma(yi, vi, data = dat_es, method = "FE")

# Helper formatting functions
fmtx <- function(x, digits = 2) formatC(x, format = "f", digits = digits)
fmtp2 <- function(pval) if (pval < .001) "< .001" else sprintf("p = %.3f", pval)
mlabfun <- function(text, x) {
  list(bquote(paste(.(text),
                    " (Q = ", .(fmtx(x$QE, digits=2)),
                    ", df = ", .(x$k - x$p), ", ",
                    .(fmtp2(x$QEp)), "; ",
                    I^2, " = ", .(fmtx(x$I2, digits=1)), "%", ")")))
}

# Define layout of rows and build forest-plot
rows <- c(6,3)
par(mar = c(4, 4, 3, 2))

forest(dat_es$yi, dat_es$vi,
       xlim = c(0.34, 1),
       refline = res_all$b,
       at = seq(0.5, 0.8, by = 0.05),
       ilab = dat$N,
       ilab.xpos = 0.83,
       cex = 0.9,
       ylim = c(-1, 8),
       rows = rows,
       slab = dat$Study,
       showweights = FALSE,
       psize = 1.5,
       top = TRUE,
       xlab = "Mean EQ5D Health State Utilities",
       mlab = mlabfun("Overall", res_all))
# Add labels for subgroups and manually add weight
text(x = 0.9, y = rows,
     labels = paste0(fmtx(weights(res_all), 2), "%"),
     cex = 0.9)
text(0.344, 6.5, labels = "UK Set",
     font = 2, adj = c(0, 0.5))
text(0.344, 3.5, labels = "China Set",
     font = 2, adj = c(0, 0.5))

# Add headings
text(0.358, 8, pos = 1, font = 4,
     labels = "Study ID")
text(0.83, 8, pos = 1, font = 4,
     labels = "n")
text(0.9, 8, pos = 1, font = 4,
     labels = "weight")
text(0.97, 8, pos = 1, font = 4,
     labels = "mean [95% CI]")

# Do analysis of subgroups
res_uk <- rma(yi, vi, subset = (Group == "UK Set"), data = dat_es, method = "FE")
res_cn <- rma(yi, vi, subset = (Group == "China Set"), data = dat_es, method = "FE")

# Add pooled subgroup results to fp
addpoly(res_uk, row = 5, mlab = mlabfun("UK Set", res_uk))
addpoly(res_cn, row = 2, mlab = mlabfun("China Set", res_cn))
addpoly(res_all, row = 0, mlab = mlabfun("Overall", res_all))
```

**Figure 2 and Sensitivity Analysis (King’s Subgroup)**

```
library(metafor)
## Loading required package: Matrix
## Loading required package: metadat
## Loading required package: numDeriv
##
## Loading the 'metafor' package (version 4.6-0). For an
## introduction to the package please type: help(metafor)
library(dplyr)
##
## Attaching package: 'dplyr'
## The following objects are masked from 'package:stats':
##
##     filter, lag
## The following objects are masked from 'package:base':
##
##     intersect, setdiff, setequal, union
# Input data
set.seed(123)
dat <- data.frame(
  Study = c("Stenson 2024", "Moore 2019",
            "Stenson 2024", "Moore 2019",
            "Stenson 2024", "Moore 2019",
            "Stenson 2024", "Moore 2019"),
  Group = c("Stage 1", "Stage 1",
            "Stage 2", "Stage 2",
            "Stage 3", "Stage 3",
            "Stage 4", "Stage 4"),
  Mean = c(0.65, 0.76,
           0.51, 0.60,
           0.45, 0.53,
           0.11, 0.50),
  SD = c(0.1052, 0.2136,
         0.1011, 0.235,
         0.1494, 0.2184,
         0.3519, 0.29),
  N = c(29, 89,
        27, 135,
        56, 206,
        50, 162)
)

# Calculate effect sizes
dat_es <- escalc(measure = "MN", mi = Mean, sdi = SD, ni = N,
                 slab = dat$Study, data = dat)

# Fit full model (random effects, Dersimonian Laird)
res_all <- rma(yi, vi, data = dat_es, method = "DL")

# Within trial calculation
# Prepare data for the two studies
stenson_data <- dat_es[dat_es$Study == "Stenson 2024", ]
moore_data <- dat_es[dat_es$Study == "Moore 2019", ]

# Calculate interaction vectors for each study relative to Stage 1
gamma_s <- c(stenson_data$yi[2] - stenson_data$yi[1],
             stenson_data$yi[3] - stenson_data$yi[1],
             stenson_data$yi[4] - stenson_data$yi[1])

gamma_m <- c(moore_data$yi[2] - moore_data$yi[1],
             moore_data$yi[3] - moore_data$yi[1],
             moore_data$yi[4] - moore_data$yi[1])

# Calculate Variance-Covariance matrices for each study
v1_s <- stenson_data$vi[1]
V_s <- matrix(c(stenson_data$vi[2] + v1_s, v1_s, v1_s,
                v1_s, stenson_data$vi[3] + v1_s, v1_s,
                v1_s, v1_s, stenson_data$vi[4] + v1_s), nrow = 3)

v1_m <- moore_data$vi[1]
V_m <- matrix(c(moore_data$vi[2] + v1_m, v1_m, v1_m,
                v1_m, moore_data$vi[3] + v1_m, v1_m,
                v1_m, v1_m, moore_data$vi[4] + v1_m), nrow = 3)

# Pool the interactions using matrix algebra
W_s <- solve(V_s)
W_m <- solve(V_m)
V_gamma_hat <- solve(W_s + W_m)
gamma_hat <- V_gamma_hat %*% (W_s %*% gamma_s + W_m %*% gamma_m)

# Calculate the pooled reference effect
dat_es$adj_effect <- NA
for(i in 1:nrow(dat_es)){
  adj_factor <- ifelse(dat_es$Group[i] == "Stage 1", 0,
                       ifelse(dat_es$Group[i] == "Stage 2", gamma_hat[1],
                              ifelse(dat_es$Group[i] == "Stage 3", gamma_hat[2],
                                     gamma_hat[3])))
  dat_es$adj_effect[i] <- dat_es$yi[i] - adj_factor
}

model_theta <- rma.uni(yi = adj_effect, vi = vi, data = dat_es, method = "FE")
theta_hat <- as.numeric(model_theta$b)
var_theta_hat_uncorrected <- as.numeric(model_theta$vb)

# Calculate final floating estimates and their variances
k <- 4
Z <- rbind(0, diag(k - 1))
W_diag <- sapply(split(dat_es, dat_es$Group), function(x) sum(1/x$vi))
W <- diag(W_diag)
ones_k <- matrix(1, nrow = k, ncol = 1)
inv_denom <- 1 / (t(ones_k) %*% W %*% ones_k)
term3 <- t(ones_k) %*% W %*% Z
A_matrix <- Z - (ones_k %*% inv_denom %*% term3)

V_beta_hat <- (matrix(1, k, k) * var_theta_hat_uncorrected) + (A_matrix %*% V_gamma_hat %*% t(A_matrix))

# Create the final results data frame to use in the plot
floating_results <- data.frame(
  Group = c("Stage 1", "Stage 2", "Stage 3", "Stage 4"),
  Mean = c(theta_hat, theta_hat + gamma_hat[1], theta_hat + gamma_hat[2], theta_hat + gamma_hat[3]),
  SE = sqrt(diag(V_beta_hat))
)
print(floating_results)
##     Group      Mean         SE
## 1 Stage 1 0.7128667 0.01494111
## 2 Stage 2 0.5629981 0.01408142
## 3 Stage 3 0.4929132 0.01214514
## 4 Stage 4 0.4024467 0.02109524
# Helper formatting functions
fmtx <- function(x, digits = 2) formatC(x, format = "f", digits = digits)
fmtp2 <- function(pval) if (pval < .001) "< .001" else sprintf("p = %.3f", pval)
mlabfun <- function(text, x) {
  list(bquote(paste(.(text),
                    " (Q = ", .(fmtx(x$QE, digits=2)),
                    ", df = ", .(x$k - x$p), ", ",
                    .(fmtp2(x$QEp)), "; ",
                    I^2, " = ", .(fmtx(x$I2, digits=1)), "%, ",
                    tau^2, " = ", .(fmtx(x$tau2, digits=4)), ")")))
}

# Define layout of rows and build forest-plot
rows <- c(17:16, 13:12, 9:8, 5:4)
par(mar = c(4, 4, 3, 2))

forest(res_all,
       xlim = c(-0.9, 1.4),
       refline = res_all$b,
       at = seq(-0.1, 1.0, by = 0.1),
       ilab = dat$N,
       ilab.xpos = 1.1,
       cex = 0.9,
       ylim = c(-3, 19),
       rows = rows,
       slab = dat$Study,
       shade = "zebra2",
       showweights = FALSE,
       psize = 1.5,
       top = TRUE,
       xlab = "Mean EQ5D Health State Utilities",
       mlab = mlabfun("Overall", res_all))

# Add labels for subgroups and manually add weight
text(x = 1.18, y = rows,
     labels = paste0(fmtx(weights(res_all), 2), "%"),
     cex = 0.9)
text(-0.9, c(18, 14, 10, 6), pos = 2, font = 2,
     labels = c("Stage 1", "Stage 2", "Stage 3", "Stage 4"))

# Add headings
text(-0.85, c(20), pos = 1, font = 4,
     labels = c("Study ID"))
text(1.1, c(20), pos = 1, font = 4,
     labels = c("n"))
text(1.18, c(20), pos = 1, font = 4,
     labels = c("weight"))
text(1.32, c(20), pos = 1, font = 4,
     labels = c("mean [95% CI]"))


# Calculate prediction interval for the overall model
pi_all <- predict(res_all)

# Add pooled subgroup results to fp
mlabfun_float <- function(text) {
  list(bquote(paste(.(text))))
}
addpoly(x = floating_results$Mean[1], sei = floating_results$SE[1], row = 15, mlab = mlabfun_float("Floating: King's Stage 1"))
addpoly(x = floating_results$Mean[2], sei = floating_results$SE[2], row = 11, mlab = mlabfun_float("Floating: King's Stage 2"))
addpoly(x = floating_results$Mean[3], sei = floating_results$SE[3], row = 7,  mlab = mlabfun_float("Floating: King's Stage 3"))
addpoly(x = floating_results$Mean[4], sei = floating_results$SE[4], row = 3,  mlab = mlabfun_float("Floating: King's Stage 4"))


# Manually add the prediction interval as an arrow
arrows(x0 = pi_all$pi.lb, x1 = pi_all$pi.ub, y0 = 1, y1 = 1,
       angle = 90, code = 3, length = 0.05, col = "black", lwd = 2)
text (-0.9, 1, pos = 4, cex =0.8, bquote("Prediction Interval: 0.273, 0.767"))

# Manually calculate the global test statistics for subgroup interaction
q_statistic <- t(gamma_hat) %*% solve(V_gamma_hat) %*% gamma_hat
q_df <- length(gamma_hat)
q_pvalue <- pchisq(q_statistic, df = q_df, lower.tail = FALSE)

# Add to plot
text(x = -0.9, y = 2, pos = 4, cex = 0.8, bquote(paste("Global Test of Interactions: ",
                                                       Q, " = ", .(fmtx(q_statistic, digits=2)),
                                                       ", df = ", .(q_df), ", ",
                                                       .(fmtp2(q_pvalue)))))

library(metafor)
## Loading required package: Matrix
## Loading required package: metadat
## Loading required package: numDeriv
##
## Loading the 'metafor' package (version 4.6-0). For an
## introduction to the package please type: help(metafor)
# Input data
set.seed(123)
dat <- data.frame(
  Study = c("Stenson 2024", "Moore 2019",
            "Stenson 2024", "Moore 2019",
            "Stenson 2024", "Moore 2019",
            "Stenson 2024", "Moore 2019"),
  Group = c("Stage 1", "Stage 1",
            "Stage 2", "Stage 2",
            "Stage 3", "Stage 3",
            "Stage 4", "Stage 4"),
  Mean = c(0.65, 0.76,
           0.51, 0.60,
           0.45, 0.53,
           0.11, 0.50),
  SD = c(0.1052, 0.2136,
         0.1011, 0.235,
         0.1494, 0.2184,
         0.3519, 0.29),
  N = c(29, 89,
        27, 135,
        56, 206,
        50, 162)
)

# Calculate effect sizes
dat_es <- escalc(measure = "MN", mi = Mean, sdi = SD, ni = N,
                 slab = dat$Study, data = dat)

# Fit full model (fixed effect, Inverse Variance)
res_all <- rma(yi, vi, data = dat_es, method = "FE")

# Within trial calculation
# Prepare data for the two studies
stenson_data <- dat_es[dat_es$Study == "Stenson 2024", ]
moore_data <- dat_es[dat_es$Study == "Moore 2019", ]

# Calculate interaction vectors (gamma) for each study relative to Stage 1
gamma_s <- c(stenson_data$yi[2] - stenson_data$yi[1],
             stenson_data$yi[3] - stenson_data$yi[1],
             stenson_data$yi[4] - stenson_data$yi[1])

gamma_m <- c(moore_data$yi[2] - moore_data$yi[1],
             moore_data$yi[3] - moore_data$yi[1],
             moore_data$yi[4] - moore_data$yi[1])

# Calculate Variance-Covariance matrices (V) for each study
v1_s <- stenson_data$vi[1]
V_s <- matrix(c(stenson_data$vi[2] + v1_s, v1_s, v1_s,
                v1_s, stenson_data$vi[3] + v1_s, v1_s,
                v1_s, v1_s, stenson_data$vi[4] + v1_s), nrow = 3)

v1_m <- moore_data$vi[1]
V_m <- matrix(c(moore_data$vi[2] + v1_m, v1_m, v1_m,
                v1_m, moore_data$vi[3] + v1_m, v1_m,
                v1_m, v1_m, moore_data$vi[4] + v1_m), nrow = 3)

# Pool the interactions using matrix algebra
W_s <- solve(V_s)
W_m <- solve(V_m)
V_gamma_hat <- solve(W_s + W_m)
gamma_hat <- V_gamma_hat %*% (W_s %*% gamma_s + W_m %*% gamma_m)

# Calculate the pooled reference effect (theta_hat)
dat_es$adj_effect <- NA
for(i in 1:nrow(dat_es)){
  adj_factor <- ifelse(dat_es$Group[i] == "Stage 1", 0,
                       ifelse(dat_es$Group[i] == "Stage 2", gamma_hat[1],
                              ifelse(dat_es$Group[i] == "Stage 3", gamma_hat[2],
                                     gamma_hat[3])))
  dat_es$adj_effect[i] <- dat_es$yi[i] - adj_factor
}

model_theta <- rma.uni(yi = adj_effect, vi = vi, data = dat_es, method = "FE")
theta_hat <- as.numeric(model_theta$b)
var_theta_hat_uncorrected <- as.numeric(model_theta$vb)

# Calculate final floating estimates and their CORRECTED variances
k <- 4
Z <- rbind(0, diag(k - 1))
W_diag <- sapply(split(dat_es, dat_es$Group), function(x) sum(1/x$vi))
W <- diag(W_diag)
ones_k <- matrix(1, nrow = k, ncol = 1)
inv_denom <- 1 / (t(ones_k) %*% W %*% ones_k)
term3 <- t(ones_k) %*% W %*% Z
A_matrix <- Z - (ones_k %*% inv_denom %*% term3)

V_beta_hat <- (matrix(1, k, k) * var_theta_hat_uncorrected) + (A_matrix %*% V_gamma_hat %*% t(A_matrix))

# Create the final results data frame to use in the plot
floating_results <- data.frame(
  Group = c("Stage 1", "Stage 2", "Stage 3", "Stage 4"),
  Mean = c(theta_hat, theta_hat + gamma_hat[1], theta_hat + gamma_hat[2], theta_hat + gamma_hat[3]),
  SE = sqrt(diag(V_beta_hat))
)
print(floating_results)
##     Group      Mean         SE
## 1 Stage 1 0.7128667 0.01494111
## 2 Stage 2 0.5629981 0.01408142
## 3 Stage 3 0.4929132 0.01214514
## 4 Stage 4 0.4024467 0.02109524
# Helper formatting functions
fmtx <- function(x, digits = 2) formatC(x, format = "f", digits = digits)
fmtp2 <- function(pval) if (pval < .001) "< .001" else sprintf("p = %.3f", pval)
mlabfun <- function(text, x) {
  list(bquote(paste(.(text),
                    " (Q = ", .(fmtx(x$QE, digits=2)),
                    ", df = ", .(x$k - x$p), ", ",
                    .(fmtp2(x$QEp)), "; ",
                    I^2, " = ", .(fmtx(x$I2, digits=1)), "%", ")")))
}

# Define layout of rows and build forest-plot
rows <- c(17:16, 13:12, 9:8, 5:4)
par(mar = c(4, 4, 3, 2))

forest(res_all,
       xlim = c(-0.9, 1.4),
       refline = res_all$b,
       at = seq(-0.1, 1.0, by = 0.1),
       ilab = dat$N,
       ilab.xpos = 1.1,
       cex = 0.9,
       ylim = c(-2, 19),
       rows = rows,
       slab = dat$Study,
       shade = "zebra2",
       showweights = FALSE,
       psize = 1.5,
       top = TRUE,
       xlab = "Mean EQ5D Health State Utilities",
       mlab = mlabfun("Overall", res_all))

# Add labels for subgroups and manually add weight
text(x = 1.18, y = rows,
     labels = paste0(fmtx(weights(res_all), 2), "%"),
     cex = 0.9)
text(-0.9, c(18, 14, 10, 6), pos = 2, font = 2,
     labels = c("Stage 1", "Stage 2", "Stage 3", "Stage 4"))

# Add headings
text(-0.85, c(20), pos = 1, font = 4,
     labels = c("Study ID"))
text(1.1, c(20), pos = 1, font = 4,
     labels = c("n"))
text(1.18, c(20), pos = 1, font = 4,
     labels = c("weight"))
text(1.32, c(20), pos = 1, font = 4,
     labels = c("mean [95% CI]"))

# Add pooled subgroup results to fp
mlabfun_float <- function(text) {
  list(bquote(paste(.(text))))
}
addpoly(x = floating_results$Mean[1], sei = floating_results$SE[1], row = 15, mlab = mlabfun_float("Floating: King's Stage 1"))
addpoly(x = floating_results$Mean[2], sei = floating_results$SE[2], row = 11, mlab = mlabfun_float("Floating: King's Stage 2"))
addpoly(x = floating_results$Mean[3], sei = floating_results$SE[3], row = 7,  mlab = mlabfun_float("Floating: King's Stage 3"))
addpoly(x = floating_results$Mean[4], sei = floating_results$SE[4], row = 3,  mlab = mlabfun_float("Floating: King's Stage 4"))

# Manually calculate the global test statistics from our existing results
q_statistic <- t(gamma_hat) %*% solve(V_gamma_hat) %*% gamma_hat
q_df <- length(gamma_hat)
q_pvalue <- pchisq(q_statistic, df = q_df, lower.tail = FALSE)

# Add to plot
text(x = -0.9, y = 2, pos = 4, cex = 0.8, bquote(paste("Global Test of Interactions: ",
                                                       Q, " = ", .(fmtx(q_statistic, digits=2)),
                                                       ", df = ", .(q_df), ", ",
                                                       .(fmtp2(q_pvalue)))))
```

**Figure 3 and Sensitivity Analysis**

```
library(metafor)
## Warning: package 'metafor' was built under R version 4.2.3
## Loading required package: Matrix
## Loading required package: metadat
## Warning: package 'metadat' was built under R version 4.2.3
## Loading required package: numDeriv
##
## Loading the 'metafor' package (version 4.4-0). For an
## introduction to the package please type: help(metafor)
# Input data
set.seed(123)
dat <- data.frame(
  Study = c("Stenson 2024", "Moore 2019",
            "Stenson 2024", "Moore 2019",
            "Stenson 2024", "Moore 2019",
            "Stenson 2024", "Moore 2019",
            "Stenson 2024", "Moore 2019"),
  Group = c("Stage 0", "Stage 0",
            "Stage 1", "Stage 1",
            "Stage 2", "Stage 2",
            "Stage 3", "Stage 3",
            "Stage 4", "Stage 4"),
  Mean = c(0.53, 0.71,
           0.34, 0.48,
           0.00, 0.36,
           0.01, 0.33,
           -0.10, 0.24),
  SD = c(0.121, 0.398,
         0.289, 0.287,
         0.671, 0.131,
         0.802, 0.097,
         0.632, 0.051),
  N = c(116, 301,
        17, 198,
        10, 73,
        8, 18,
        14, 5)
)

# Calculate effect sizes
dat_es <- escalc(measure = "MN", mi = Mean, sdi = SD, ni = N,
                 slab = dat$Study, data = dat)

# Fit full model (random effects, Dersimonian Laird)
res_all <- rma(yi, vi, data = dat_es, method = "DL")

# Helper formatting functions
fmtx <- function(x, digits = 2) formatC(x, format = "f", digits = digits)
fmtp2 <- function(pval) if (pval < .001) "< .001" else sprintf("p = %.3f", pval)
mlabfun <- function(text, x) {
  list(bquote(paste(.(text),
                    " (Q = ", .(fmtx(x$QE, digits=2)),
                    ", df = ", .(x$k - x$p), ", ",
                    .(fmtp2(x$QEp)), "; ",
                    I^2, " = ", .(fmtx(x$I2, digits=1)), "%, ",
                    tau^2, " = ", .(fmtx(x$tau2, digits=4)), ")")))
}

# Define layout of rows and build forest-plot
rows <- c(24:23, 20:19, 16:15, 12:11, 8:7)
par(mar = c(4, 4, 3, 2))

forest(dat_es$yi, dat_es$vi,
       xlim = c(-1.4 , 1.5),
       refline = res_all$b,
       at = seq(-0.6, 1.0, by = 0.1),
       ilab = dat$N,
       ilab.xpos = 1.1,
       cex = 0.9,
       ylim = c(3, 26),
       rows = rows,
       slab = dat$Study,
       shade = "zebra2",
       showweights = FALSE,
       psize = 1.5,
       top = TRUE,
       xlab = "Mean EQ5D Health State Utilities",
       mlab = mlabfun("Overall", res_all))
# Calculate prediction interval for the overall model
pi_all <- predict(res_all)

# Add labels for subgroups and manually add weight
text(x = 1.21, y = rows,
     labels = paste0(fmtx(weights(res_all), 2), "%"),
     cex = 0.9)
text(-1.28, c(25, 21, 17, 13, 9), pos = 2, font = 2,
     labels = c("Stage 0", "Stage 1", "Stage 2", "Stage 3", "Stage 4"))

# Add headings
text(-1.34, 27, c(20), pos = 1, font = 4,
     labels = c("Study ID"))
text(1.1, 27, c(20), pos = 1, font = 4,
     labels = c("n"))
text(1.21, 27, c(20), pos = 1, font = 4,
     labels = c("weight"))
text(1.4, 27, c(20), pos = 1, font = 4,
     labels = c("mean [95% CI]"))

# Do analysis of subgroups
res0 <- rma(yi, vi, subset = (Group == "Stage 0"), data = dat_es, method = "DL")
res1 <- rma(yi, vi, subset = (Group == "Stage 1"), data = dat_es, method = "DL")
res2 <- rma(yi, vi, subset = (Group == "Stage 2"), data = dat_es, method = "DL")
res3 <- rma(yi, vi, subset = (Group == "Stage 3"), data = dat_es, method = "DL")
res4 <- rma(yi, vi, subset = (Group == "Stage 4"), data = dat_es, method = "DL")

# Add pooled subgroup results to fp
addpoly(res0, row = 22, mlab = mlabfun("Mito's Stage 0", res1))
addpoly(res1, row = 18, mlab = mlabfun("Mito's Stage 1", res1))
addpoly(res2, row = 14, mlab = mlabfun("Mito's Stage 2", res2))
addpoly(res3, row = 10,  mlab = mlabfun("Mito's Stage 3", res3))
addpoly(res4, row = 6,  mlab = mlabfun("Mito's Stage 4", res4))
addpoly(res_all, row = 4, mlab = mlabfun("Overall", res_all))

# Manually add the prediction interval as an arrow
arrows(x0 = pi_all$pi.lb, x1 = pi_all$pi.ub, y0 = 3, y1 = 3,
       angle = 90, code = 3, length = 0.05, col = "black", lwd = 2)
text (-0.9, 3, pos = 4, cex =0.8, bquote("Prediction Interval: 0.0659, 0.675"))

# Test for subgroup differences and add to fp
res_mod <- rma(yi, vi, mods = ~ Group, data = dat_es, method = "REML")
text(-1.3, 2.5, pos = 4, cex = 0.8, bquote(paste("Test for Subgroup Differences: ",
                                               Q[M], " = ", .(fmtx(res_mod$QM, digits=2)),
                                               ", df = ", .(res_mod$p - 1), ", ", .(fmtp2(res_mod$QMp)))))

library(metafor)
## Warning: package 'metafor' was built under R version 4.2.3
## Loading required package: Matrix
## Loading required package: metadat
## Warning: package 'metadat' was built under R version 4.2.3
## Loading required package: numDeriv
##
## Loading the 'metafor' package (version 4.4-0). For an
## introduction to the package please type: help(metafor)
# Input data
set.seed(123)
dat <- data.frame(
  Study = c("Stenson 2024", "Moore 2019",
            "Stenson 2024", "Moore 2019",
            "Stenson 2024", "Moore 2019",
            "Stenson 2024", "Moore 2019",
            "Stenson 2024", "Moore 2019"),
  Group = c("Stage 0", "Stage 0",
            "Stage 1", "Stage 1",
            "Stage 2", "Stage 2",
            "Stage 3", "Stage 3",
            "Stage 4", "Stage 4"),
  Mean = c(0.53, 0.71,
           0.34, 0.48,
           0.00, 0.36,
           0.01, 0.33,
           -0.10, 0.24),
  SD = c(0.121, 0.398,
         0.289, 0.287,
         0.671, 0.131,
         0.802, 0.097,
         0.632, 0.051),
  N = c(116, 301,
        17, 198,
        10, 73,
        8, 18,
        14, 5)
)

# Calculate effect sizes
dat_es <- escalc(measure = "MN", mi = Mean, sdi = SD, ni = N,
                 slab = dat$Study, data = dat)

# Fit full model (fixed effect, Inverse Variance)
res_all <- rma(yi, vi, data = dat_es, method = "FE")

# Helper formatting functions
fmtx <- function(x, digits = 2) formatC(x, format = "f", digits = digits)
fmtp2 <- function(pval) if (pval < .001) "< .001" else sprintf("p = %.3f", pval)
mlabfun <- function(text, x) {
  list(bquote(paste(.(text),
                    " (Q = ", .(fmtx(x$QE, digits=2)),
                    ", df = ", .(x$k - x$p), ", ",
                    .(fmtp2(x$QEp)), "; ",
                    I^2, " = ", .(fmtx(x$I2, digits=1)), "%", ")")))
}

# Define layout of rows and build forest-plot
rows <- c(24:23, 20:19, 16:15, 12:11, 8:7)
par(mar = c(4, 4, 3, 2))

forest(dat_es$yi, dat_es$vi,
       xlim = c(-1.4 , 1.5),
       refline = res_all$b,
       at = seq(-0.6, 1.0, by = 0.1),
       ilab = dat$N,
       ilab.xpos = 1.1,
       cex = 0.9,
       ylim = c(3, 26),
       rows = rows,
       slab = dat$Study,
       shade = "zebra2",
       showweights = FALSE,
       psize = 1.5,
       top = TRUE,
       xlab = "Mean EQ5D Health State Utilities",
       mlab = mlabfun("Overall", res_all))
# Add labels for subgroups and manually add weight
text(x = 1.21, y = rows,
     labels = paste0(fmtx(weights(res_all), 2), "%"),
     cex = 0.9)
text(-1.28, c(25, 21, 17, 13, 9), pos = 2, font = 2,
     labels = c("Stage 0", "Stage 1", "Stage 2", "Stage 3", "Stage 4"))

# Add headings
text(-1.34, 27, c(20), pos = 1, font = 4,
     labels = c("Study ID"))
text(1.1, 27, c(20), pos = 1, font = 4,
     labels = c("n"))
text(1.21, 27, c(20), pos = 1, font = 4,
     labels = c("weight"))
text(1.4, 27, c(20), pos = 1, font = 4,
     labels = c("mean [95% CI]"))

# Do analysis of subgroups
res0 <- rma(yi, vi, subset = (Group == "Stage 0"), data = dat_es, method = "FE")
res1 <- rma(yi, vi, subset = (Group == "Stage 1"), data = dat_es, method = "FE")
res2 <- rma(yi, vi, subset = (Group == "Stage 2"), data = dat_es, method = "FE")
res3 <- rma(yi, vi, subset = (Group == "Stage 3"), data = dat_es, method = "FE")
res4 <- rma(yi, vi, subset = (Group == "Stage 4"), data = dat_es, method = "FE")

# Add pooled subgroup results to fp
addpoly(res0, row = 22, mlab = mlabfun("Mito's Stage 0", res1))
addpoly(res1, row = 18, mlab = mlabfun("Mito's Stage 1", res1))
addpoly(res2, row = 14, mlab = mlabfun("Mito's Stage 2", res2))
addpoly(res3, row = 10,  mlab = mlabfun("Mito's Stage 3", res3))
addpoly(res4, row = 6,  mlab = mlabfun("Mito's Stage 4", res4))
addpoly(res_all, row = 4, mlab = mlabfun("Overall", res_all))

# Test for subgroup differences and add to fp
res_mod <- rma(yi, vi, mods = ~ Group, data = dat_es, method = "REML")
text(-2, 0.8, pos = 4, cex = 0.8, bquote(paste("Test for Subgroup Differences: ",
                                               Q[M], " = ", .(fmtx(res_mod$QM, digits=2)),
                                               ", df = ", .(res_mod$p - 1), ", ", .(fmtp2(res_mod$QMp)))))
```

**Figure 4 and Sensitivity Analysis**

```
library(metafor)
## Warning: package 'metafor' was built under R version 4.2.3
## Loading required package: Matrix
## Loading required package: metadat
## Warning: package 'metadat' was built under R version 4.2.3
## Loading required package: numDeriv
##
## Loading the 'metafor' package (version 4.4-0). For an
## introduction to the package please type: help(metafor)
# Input data
set.seed(123)
dat <- data.frame(
  Study = rep("Green 2003", 4),
  Group = c("Level 1", "Level 2", "Level 3", "Level 4"),
  Mean = c(0.74, 0.63, 0.51, 0.37),
  SD = c(0.19, 0.17, 0.18, 0.18),
  N = c(15, 21, 22, 19)
)

# Calculate effect sizes
dat_es <- escalc(measure = "MN", mi = Mean, sdi = SD, ni = N,
                 slab = dat$Study, data = dat)

# Fit full model (fixed)
es_overall <- rma(yi, vi, data = dat_es, method = "DL")

# Helper formatting functions
fmtx <- function(x, digits = 2) formatC(x, format = "f", digits = digits)
fmtp2 <- function(pval) if (pval < .001) "< .001" else sprintf("p = %.3f", pval)
mlabfun <- function(text, x) {
  list(bquote(paste(.(text),
                    " (Q = ", .(fmtx(x$QE, digits=2)),
                    ", df = ", .(x$k - x$p), ", ",
                    .(fmtp2(x$QEp)), "; ",
                    I^2, " = ", .(fmtx(x$I2, digits=1)), "%, ",
                    tau^2, " = ", .(fmtx(x$tau2, digits=4)), ")")))
}
# Define layout of rows
study_rows <- c(10, 7, 4, 1)
poly_rows <- c(9, 6, 3, 0)
par(mar = c(4, 4, 3, 2))

forest(dat_es$yi, dat_es$vi,
       slab = dat_es$Study,
       xlim = c(-0.4, 1.3),
       at = seq(0.1, 1.0, by = 0.1),
       ylim = c(-4, 14),
       rows = study_rows,
       psize = 1.5,
       refline = es_overall$b,
       cex = 0.9,
       ilab = dat$N,
       ilab.xpos = 1.02,
       xlab = "Mean EQ5D Health State Utilities")

# Calculate prediction interval for the overall model
pi_all <- predict(es_overall)

# Add labels for subgroups and manually add weight
text(x = 1.11, y = study_rows,
     labels = paste0(fmtx(weights(es_overall), 2), "%"),
     cex = 0.9)
text(-0.4, study_rows + 1, font = 2, pos = 4,
     labels = c("Level 1", "Level 2", "Level 3", "Level 4"))

# Add headings
text(-0.355, 13, pos = 1, font = 4, labels = "Study ID")
text(1.02, 13, pos = 1, font = 4, labels = "n")
text(1.115,  13, pos = 1, font = 4, labels = "weight")
text(1.23, 13, pos = 1, font = 4, labels = "mean [95% CI]")

# Do analysis of subgroups
res_1 <- rma(yi, vi, subset = (Group == "Level 1"), data = dat_es, method = "DL")
res_2 <- rma(yi, vi, subset = (Group == "Level 2"), data = dat_es, method = "DL")
res_3 <- rma(yi, vi, subset = (Group == "Level 3"), data = dat_es, method = "DL")
res_4 <- rma(yi, vi, subset = (Group == "Level 4"), data = dat_es, method = "DL")

# Manually add the prediction interval as an arrow
arrows(x0 = pi_all$pi.lb, x1 = pi_all$pi.ub, y0 = -4, y1 = -4,
       angle = 90, code = 3, length = 0.05, col = "black", lwd = 2)
text (-0.1, -4, pos = 4, cex =0.8, bquote("Prediction Interval: 0.244, 0.878"))

# Add pooled subgroup results to fp
addpoly(res_1, row = 9, mlab = mlabfun("Level 1", res_1))
addpoly(res_2, row = 6, mlab = mlabfun("Level 2", res_2))
addpoly(res_3, row = 3, mlab = mlabfun("Level 3", res_3))
addpoly(res_4, row = 0, mlab = mlabfun("Level 4", res_4))
addpoly(es_overall, row = -2, mlab = mlabfun("Overall", es_overall))

library(metafor)
## Warning: package 'metafor' was built under R version 4.2.3
## Loading required package: Matrix
## Loading required package: metadat
## Warning: package 'metadat' was built under R version 4.2.3
## Loading required package: numDeriv
##
## Loading the 'metafor' package (version 4.4-0). For an
## introduction to the package please type: help(metafor)
# Input data
set.seed(123)
dat <- data.frame(
  Study = rep("Green 2003", 4),
  Group = c("Level 1", "Level 2", "Level 3", "Level 4"),
  Mean = c(0.74, 0.63, 0.51, 0.37),
  SD = c(0.19, 0.17, 0.18, 0.18),
  N = c(15, 21, 22, 19)
)

# Calculate effect sizes
dat_es <- escalc(measure = "MN", mi = Mean, sdi = SD, ni = N,
                 slab = dat$Study, data = dat)

# Fit full model (fixed)
es_overall <- rma(yi, vi, data = dat_es, method = "FE")

# Helper formatting functions
fmtx <- function(x, digits = 2) formatC(x, format = "f", digits = digits)
fmtp2 <- function(pval) if (pval < .001) "< .001" else sprintf("p = %.3f", pval)
mlabfun <- function(text, x) {
  list(bquote(paste(.(text),
                    " (Q = ", .(fmtx(x$QE, digits=2)),
                    ", df = ", .(x$k - x$p), ", ",
                    .(fmtp2(x$QEp)), "; ",
                    I^2, " = ", .(fmtx(x$I2, digits=1)), "%)",)))
}
# Define layout of rows
study_rows <- c(10, 7, 4, 1)
poly_rows <- c(9, 6, 3, 0)
par(mar = c(4, 4, 3, 2))

forest(dat_es$yi, dat_es$vi,
       slab = dat_es$Study,
       xlim = c(-0.4, 1.3),
       at = seq(0.1, 1.0, by = 0.1),
       ylim = c(-3, 14),
       rows = study_rows,
       psize = 1.5,
       refline = es_overall$b,
       cex = 0.9,
       ilab = dat$N,
       ilab.xpos = 1.02,
       xlab = "Mean EQ5D Health State Utilities")

# Add labels for subgroups and manually add weight
text(x = 1.11, y = study_rows,
     labels = paste0(fmtx(weights(es_overall), 2), "%"),
     cex = 0.9)
text(-0.4, study_rows + 1, font = 2, pos = 4,
     labels = c("Level 1", "Level 2", "Level 3", "Level 4"))

# Add headings
text(-0.355, 13, pos = 1, font = 4, labels = "Study ID")
text(1.02, 13, pos = 1, font = 4, labels = "n")
text(1.115,  13, pos = 1, font = 4, labels = "weight")
text(1.23, 13, pos = 1, font = 4, labels = "mean [95% CI]")

# Do analysis of subgroups
res_1 <- rma(yi, vi, subset = (Group == "Level 1"), data = dat_es, method = "FE")
res_2 <- rma(yi, vi, subset = (Group == "Level 2"), data = dat_es, method = "FE")
res_3 <- rma(yi, vi, subset = (Group == "Level 3"), data = dat_es, method = "FE")
res_4 <- rma(yi, vi, subset = (Group == "Level 4"), data = dat_es, method = "FE")

# Add pooled subgroup results to fp
addpoly(res_1, row = 9, mlab = mlabfun("Level 1", res_1))
addpoly(res_2, row = 6, mlab = mlabfun("Level 2", res_2))
addpoly(res_3, row = 3, mlab = mlabfun("Level 3", res_3))
addpoly(res_4, row = 0, mlab = mlabfun("Level 4", res_4))
addpoly(es_overall, row = -2, mlab = mlabfun("Overall", es_overall))
```

**Figure 5 and Sensitivity Analysis**

```
library(metafor)
## Warning: package 'metafor' was built under R version 4.2.3
## Loading required package: Matrix
## Loading required package: metadat
## Warning: package 'metadat' was built under R version 4.2.3
## Loading required package: numDeriv
##
## Loading the 'metafor' package (version 4.4-0). For an
## introduction to the package please type: help(metafor)
# Input data
set.seed(123)
dat <- data.frame(
  Study = rep("Thakore 2019", 5),
  Group = c("FT9 0", "FT9 1", "FT9 2", "FT9 3", "FT9 4"),
  Mean = c(0.789, 0.743, 0.629, 0.541, 0.464),
  SE = c(0.013, 0.009, 0.010, 0.012, 0.015),
  N = c(160, 494, 542, 381, 291)
)
dat$Var <- dat$SE^2

# Calculate effect sizes
dat_es <- dat
dat_es$yi <- dat_es$Mean
dat_es$vi <- dat_es$Var

# Fit full model (random)
es_overall <- rma(yi, vi, data = dat_es, method = "DL")

# Helper formatting functions
fmtx <- function(x, digits = 2) formatC(x, format = "f", digits = digits)
fmtp2 <- function(pval) if (pval < .001) "< .001" else sprintf("p = %.3f", pval)
mlabfun <- function(text, x) {
  list(bquote(paste(.(text),
                    " (Q = ", .(fmtx(x$QE, digits=2)),
                    ", df = ", .(x$k - x$p), ", ",
                    .(fmtp2(x$QEp)), "; ",
                    I^2, " = ", .(fmtx(x$I2, digits=1)), "%, ",
                    tau^2, " = ", .(fmtx(x$tau2, digits=4)), ")")))
}
# Define layout of rows
study_rows <- c(13, 10, 7, 4, 1)
poly_rows  <- c(12, 9, 6, 3, 0)
par(mar = c(4, 4, 3, 2))

forest(dat_es$yi, dat_es$vi,
       slab = dat_es$Study,
       xlim = c(-0.4, 1.3),
       at = seq(0.1, 1.0, by = 0.1),
       ylim = c(-4, 17),
       rows = study_rows,
       psize = 1.5,
       refline = es_overall$b,
       cex = 0.9,
       ilab = dat$N,
       ilab.xpos = 1.02,
       xlab = "Mean EQ5D Health State Utilities")

# Calculate prediction interval for the overall model
pi_all <- predict(es_overall)

# Add labels for subgroups and manually add weight
text(x = 1.11, y = study_rows,
     labels = paste0(fmtx(weights(es_overall), 2), "%"),
     cex = 0.9)
text(-0.4, study_rows + 1, font = 2, pos = 4,
     labels = c("Level 1", "Level 2", "Level 3", "Level 4"))

# Add headings
text(-0.361, 16, pos = 1, font = 4, labels = "Study ID")
text(1.02, 16, pos = 1, font = 4, labels = "n")
text(1.115,  16, pos = 1, font = 4, labels = "weight")
text(1.245, 16, pos = 1, font = 4, labels = "mean [95% CI]")

# Do analysis of subgroups
res_0 <- rma(yi, vi, subset = (Group == "FT9 0"), data = dat_es, method = "DL")
res_1 <- rma(yi, vi, subset = (Group == "FT9 1"), data = dat_es, method = "DL")
res_2 <- rma(yi, vi, subset = (Group == "FT9 2"), data = dat_es, method = "DL")
res_3 <- rma(yi, vi, subset = (Group == "FT9 3"), data = dat_es, method = "DL")
res_4 <- rma(yi, vi, subset = (Group == "FT9 4"), data = dat_es, method = "DL")

# Manually add the prediction interval as an arrow
arrows(x0 = pi_all$pi.lb, x1 = pi_all$pi.ub, y0 = -4, y1 = -4,
       angle = 90, code = 3, length = 0.05, col = "black", lwd = 2)
text (-0.1, -4, pos = 4, cex =0.8, bquote("Prediction Interval: 0.371, 0.896"))

# Add pooled subgroup results to fp
addpoly(res_0, row = 12, mlab = mlabfun("FT9 0", res_0))
addpoly(res_1, row = 9,  mlab = mlabfun("FT9 1", res_1))
addpoly(res_2, row = 6,  mlab = mlabfun("FT9 2", res_2))
addpoly(res_3, row = 3,  mlab = mlabfun("FT9 3", res_3))
addpoly(res_4, row = 0,  mlab = mlabfun("FT9 4", res_4))
addpoly(es_overall, row = -2, mlab = mlabfun("Overall", es_overall))

library(metafor)
## Warning: package 'metafor' was built under R version 4.2.3
## Loading required package: Matrix
## Loading required package: metadat
## Warning: package 'metadat' was built under R version 4.2.3
## Loading required package: numDeriv
##
## Loading the 'metafor' package (version 4.4-0). For an
## introduction to the package please type: help(metafor)
# Input data
set.seed(123)
dat <- data.frame(
  Study = rep("Thakore 2019", 5),
  Group = c("FT9 0", "FT9 1", "FT9 2", "FT9 3", "FT9 4"),
  Mean = c(0.789, 0.743, 0.629, 0.541, 0.464),
  SE = c(0.013, 0.009, 0.010, 0.012, 0.015),
  N = c(160, 494, 542, 381, 291)
)
dat$Var <- dat$SE^2

# Calculate effect sizes
dat_es <- dat
dat_es$yi <- dat_es$Mean
dat_es$vi <- dat_es$Var

# Fit full model (fixed)
es_overall <- rma(yi, vi, data = dat_es, method = "FE")

# Helper formatting functions
fmtx <- function(x, digits = 2) formatC(x, format = "f", digits = digits)
fmtp2 <- function(pval) if (pval < .001) "< .001" else sprintf("p = %.3f", pval)
mlabfun <- function(text, x) {
  list(bquote(paste(.(text),
                    " (Q = ", .(fmtx(x$QE, digits=2)),
                    ", df = ", .(x$k - x$p), ", ",
                    .(fmtp2(x$QEp)), "; ",
                    I^2, " = ", .(fmtx(x$I2, digits=1)), "%)",
                    )))
}
# Define layout of rows
study_rows <- c(13, 10, 7, 4, 1)
poly_rows  <- c(12, 9, 6, 3, 0)
par(mar = c(4, 4, 3, 2))

forest(dat_es$yi, dat_es$vi,
       slab = dat_es$Study,
       xlim = c(-0.4, 1.3),
       at = seq(0.1, 1.0, by = 0.1),
       ylim = c(-3, 17),
       rows = study_rows,
       psize = 1.5,
       refline = es_overall$b,
       cex = 0.9,
       ilab = dat$N,
       ilab.xpos = 1.02,
       xlab = "Mean EQ5D Health State Utilities")

# Add labels for subgroups and manually add weight
text(x = 1.11, y = study_rows,
     labels = paste0(fmtx(weights(es_overall), 2), "%"),
     cex = 0.9)
text(-0.4, study_rows + 1, font = 2, pos = 4,
     labels = c("Level 1", "Level 2", "Level 3", "Level 4"))

# Add headings
text(-0.361, 16, pos = 1, font = 4, labels = "Study ID")
text(1.02, 16, pos = 1, font = 4, labels = "n")
text(1.115,  16, pos = 1, font = 4, labels = "weight")
text(1.245, 16, pos = 1, font = 4, labels = "mean [95% CI]")

# Do analysis of subgroups
res_0 <- rma(yi, vi, subset = (Group == "FT9 0"), data = dat_es, method = "FE")
res_1 <- rma(yi, vi, subset = (Group == "FT9 1"), data = dat_es, method = "FE")
res_2 <- rma(yi, vi, subset = (Group == "FT9 2"), data = dat_es, method = "FE")
res_3 <- rma(yi, vi, subset = (Group == "FT9 3"), data = dat_es, method = "FE")
res_4 <- rma(yi, vi, subset = (Group == "FT9 4"), data = dat_es, method = "FE")

# Add pooled subgroup results to fp
addpoly(res_0, row = 12, mlab = mlabfun("FT9 0", res_0))
addpoly(res_1, row = 9,  mlab = mlabfun("FT9 1", res_1))
addpoly(res_2, row = 6,  mlab = mlabfun("FT9 2", res_2))
addpoly(res_3, row = 3,  mlab = mlabfun("FT9 3", res_3))
addpoly(res_4, row = 0,  mlab = mlabfun("FT9 4", res_4))
addpoly(es_overall, row = -2, mlab = mlabfun("Overall", es_overall))
```

References (as appearing in the supplementary material)

1. Ackerman SJ, Sullivan EM, Beusterien KM, Natter HM, Gelinas DF, Patrick DL. Cost effectiveness of recombinant human insulin-like growth factor I therapy in patients with ALS. PharmacoEconomics. 1999; 15(2):179-95.

2. Adelman EE, Albert SM, Rabkin JG, Del Bene ML, Tider T, O'Sullivan I. Disparities in perceptions of distress and burden in ALS patients and family caregivers. Neurology. 2004; 62(10):1766-70.

3. Akerblom Y, Zetterberg L, Larsson BJ, Nyholm D, Nygren I, Asenlof P. Pain, disease severity and associations with individual quality of life in patients with motor neuron diseases. BMC palliative care. 2021; 20(1):154.

4. Conroy E, Velez-Gomez B, O'Brien D, Heverin M, Hardiman O, McDermott C, Galvin M. IMPACT-ALS: summary of results from a European survey of people living with ALS. Amyotrophic Lateral Sclerosis and Frontotemporal Degeneration. 2023; 24(7-8):641EP-50.

5. Gebrehiwet P, Meng L, Rudnicki SA, Sarocco P, Wei J, Wolff AA, et al. Health utilities and quality-adjusted life years for patients with amyotrophic lateral sclerosis receiving reldesemtiv or placebo in FORTITUDE-ALS. Journal of medical economics. 2023; 26(1):488‐93.

6. Green C, Kiebert G, Murphy C, Mitchell JD, O'Brien M, Burrell A, Leigh PN. Patients' health-related quality-of-life and health state values for motor neurone disease/amyotrophic lateral sclerosis. Quality of Life Research. 2003; 12(5):565EP-74.

7. Kiebert GM, Green C, Murphy C, Mitchell JD, O'Brien M, Burrell A, Leigh PN. Patients' health-related quality of life and utilities associated with different stages of amyotrophic lateral sclerosis. Journal of the Neurological Sciences. 2001; 191(1-2):87EP-93.

8. Hecht M, Hillemacher T, Grasel E, Tigges S, Winterholler M, Heuss D, et al. Subjective experience and coping in ALS. Amyotrophic Lateral Sclerosis and Other Motor Neuron Disorders. 2002; 3(4):225EP-31.

9. Helleman J, Johnson B, Holdom C, Hobson E, Murray D, Steyn FJ, et al. Patient perspectives on digital healthcare technology in care and clinical trials for motor neuron disease: an international survey. Journal of Neurology. 2022; 269(11):6003EP-13.

10. Jones AR, Jivraj N, Balendra R, Murphy C, Kelly J, Thornhill M, et al. Health utility decreases with increasing clinical stage in amyotrophic lateral sclerosis. Amyotrophic Lateral Sclerosis and Frontotemporal Degeneration. 2014; 15(3-4):285EP-91.

11. Maksymowicz-Sliwinska A, Lule D, Nieporecki K, Ciecwierska K, Ludolph AC, Kuzma-Kozakiewicz M. Attitudes of caregivers towards prolonging and shortening life in advanced stages of amyotrophic lateral sclerosis. Folia neuropathologica. 2023; 61(4):349EP-59.

12. Moore A, Young CA, Hughes DA. Health Utilities and Costs for Motor Neurone Disease. Value in health : the journal of the International Society for Pharmacoeconomics and Outcomes Research. 2019; 22(11):1257-65.

13. Raheja D, Stephens HE, Lehman E, Walsh S, Yang C, Simmons Z. Patient-reported problematic symptoms in an ALS treatment trial. Amyotrophic lateral sclerosis & frontotemporal degeneration. 2016; 17(3‐4):198‐205.

14. Rudnicki SA. Factors influencing a patient's decision regarding riluzole: An early experience. Journal of the Neurological Sciences. 1997; 152(SUPPL. 1):S80EP-S1.

15. Sandstedt P, Littorin S, Crode Widsell G, Johansson S, Gottberg K, Ytterberg C, et al. Caregiver experience, health-related quality of life and life satisfaction among informal caregivers to patients with amyotrophic lateral sclerosis: A cross-sectional study. Journal of clinical nursing. 2018; 27(23-24):4321EP-30.

16. Seo J, Saurkar S, Fernandez GS, Das A, Goutman SA, Heidenreich S. Preferences of Patients with Amyotrophic Lateral Sclerosis for Intrathecal Drug Delivery: Choosing between an Implanted Drug-Delivery Device and Therapeutic Lumbar Puncture. Patient. 2024; 17(2):161EP-77.

17. Stenson K, Fecteau TE, O'Callaghan L, Bryden P, Mellor J, Wright J, et al. Health-related quality of life across disease stages in patients with amyotrophic lateral sclerosis: results from a real-world survey. Journal of Neurology. 2024; 271(5):2390EP-404.

18. Tarlarini C, Greco LC, Lizio A, Gerardi F, Sansone VA, Lunetta C. Changes in taste perception in ALS patients with gastrostomy feeding tubes and the impact on their quality-of-life. Amyotrophic Lateral Sclerosis and Frontotemporal Degeneration. 2017; 18(Supplement 2):313.

19. Thakore N, Lapin B, Pioro E. Health utility declines with advancing Fine'til 9 stage of ALS. Neurology. 2019; 92(15 Supplement 1).

20. Tobin K, Maguire S, Corr B, Normand C, Hardiman O, Galvin M. Discrete choice experiment for eliciting preference for health services for patients with ALS and their informal caregivers. BMC health services research. 2021; 21(1):213.

21. Trail M, Nelson ND, Van JN, Appel SH, Lai EC. Major stressors facing patients with amyotrophic lateral sclerosis (ALS): A survey to identify their concerns and to compare with those of their caregivers. Amyotrophic Lateral Sclerosis and Other Motor Neuron Disorders. 2004; 5(1):40EP-5.

22. Van Eijk RPA, Van Den Berg LH, Lu Y. Cultivating Patient Preferences in ALS Clinical Trials: Reliability and Prognostic Value of the Patient-Ranked Order of Function. Neurology. 2024; 103(2):e209502.

23. van Eijk RPA, van den Berg LH, Lu Y. Composite endpoint for ALS clinical trials based on patient preference: patient-Ranked Order of Function (PROOF). Journal of neurology, neurosurgery, and psychiatry. 2022; 93(5):539‐46.

24. Wagner KN, Nagaraja HN, Allain DC, Quick A, Kolb SJ, Roggenbuck J. Patients with sporadic and familial amyotrophic lateral sclerosis found value in genetic testing. Molecular genetics & genomic medicine. 2018; 6(2):224EP-9.

25. Wei QQ, Hou Y, Chen Y, Ou R, Cao B, Zhang L, et al. Health-related quality of life in amyotrophic lateral sclerosis using EQ-5D-5L. Health and quality of life outcomes. 2021; 19(1):181.

26. Karam SG, Zhang Y, Pardo-Hernandez H, Siebert U, Koopman L, Noyes J, et al. ROBVALU: a tool for assessing risk of bias in studies about people’s values, utilities, or importance of health outcomes. bmj. 2024; 385.

27. Viechtbauer W. Conducting meta-analyses in R with the metafor package. Journal of statistical software. 2010; 36(3):1-48.

28. Saeed YA, Phoon A, Bielecki JM, Mitsakakis N, Bremner KE, Abrahamyan L, et al. A systematic review and meta-analysis of health utilities in patients with chronic hepatitis C. Value in Health. 2020; 23(1):127-37.

29. Borenstein M, Hedges LV, Higgins JPT, Rothstein HR. Introduction to Meta-Analysis. Chichester, UK: John Wiley & Sons; 2009.

30. Higgins JP, Thomas J, Chandler J, Cumpston M, Li T, Page MJ, Welch VA. Cochrane handbook for systematic reviews of interventions. John Wiley & Sons; 2019.

31. Wan X, Wang W, Liu J, Tong T. Estimating the sample mean and standard deviation from the sample size, median, range and/or interquartile range. BMC medical research methodology. 2014; 14:1-13.
